# S100A10-ANXA2 tetramer inhibition hampers hepatic stellate cell activation in human MASLD organoids

**DOI:** 10.1038/s44321-026-00464-y

**Published:** 2026-06-10

**Authors:** Miranda Türkal, Christine Maeder, Marta Correia de Sousa, Margot Fournier, Sanae El-Harane, Monika Gjorgjieva, Michelangelo Foti, Pierre Maechler, Etienne Delangre

**Affiliations:** 1 Department of Cell Physiology and Metabolism, Faculty of Medicine, University of Geneva, Geneva, Switzerland; 2 Faculty of Medicine, Diabetes Center, University of Geneva, Geneva, Switzerland; 3 Department of Medicine, Faculty of Medicine, University of Geneva, Geneva, Switzerland

**Keywords:** Digestive System, Metabolism

## Abstract

Metabolic dysfunction-Associated Steatotic Liver Disease (MASLD), initiated by the pathological lipid accumulation within hepatocytes, can progress towards Metabolic dysfunction-Associated SteatoHepatitis (MASH) characterized by inflammation and fibrosis. Hepatic fibrosis is the strongest predictor of liver-related mortality, yet effective antifibrotic therapies remain limited, calling for identification of new molecular targets. Our previous work identified S100A10 as a MASLD promoter, suggesting that its association with AnnexinA2 (ANXA2) within the S100A10-ANXA2 heterotetramer (A2t) might promote hepatic fibrosis. Here, we inhibited A2t using its inhibitor, A2ti-1, in human hepatic stellate cells (LX-2) and in human multilineage liver organoids (HLOs) modeling MASLD. In LX-2, A2ti-1 reduced α-SMA protein levels and expression of profibrotic genes, indicating direct suppression of stellate cell activation. In HLOs, A2ti-1 significantly reduced fibrosis by lowering α-SMA levels, collagen deposition, and profibrotic gene expression, without altering steatosis. Mechanistically, A2ti-1 inhibited hepatic stellate cell activation through a SMAD-independent mechanism involving reduced STAT3 phosphorylation. These findings identify the S100A10-ANXA2 tetramer as a new regulator of hepatic stellate cells activation and highlight its inhibition as a promising antifibrotic strategy in MASH.

## Synopsis

Human liver organoids (HLOs) bridge the gap between conventional 2D in vitro systems and animal models. Here, MASLD modelling organoids are employed to elucidate the role of S100A10/ANXA2 tetramer in hepatic stellate cell activation and liver fibrosis.

- Hepatic cell diversity and key functions are recapitulated in HLOs derived from embryonic stem cells.
- Key pathological features of MASLD/MASH are reproduced leveraging HLOs.
- Hepatic fibrosis establishment and progression are impaired by S100A10/ANXA2 inhibition via a direct stellate cell-dependent mechanism.
- Hepatic stellate cell activation is fostered by S100A10/ANXA2 through a SMAD-independent pathway.

## Abstract (web summary)

Human liver organoids (HLOs) bridge the gap between conventional 2D in vitro systems and animal models. Here, MASLD modelling organoids are employed to elucidate the role of S100A10/ANXA2 tetramer in hepatic stellate cell activation and liver fibrosis.

## Graphical abstract

**Figure:**
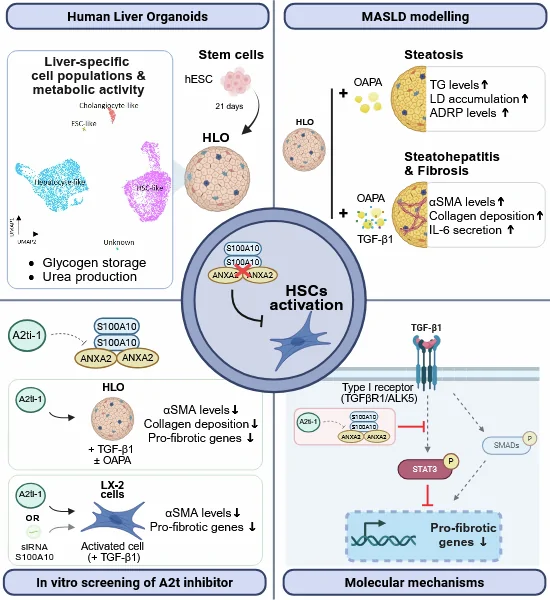

**Figure:**
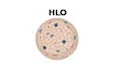

The paper explained **Problem**

Metabolic dysfunction Associated Steatotic Liver Disease (MASLD) has emerged as a major global health issue, with steatosis affecting 30% of the worldwide population while the treatment of hepatic fibrosis remains a major clinical challenge. The absence of reliable preclinical models that faithfully recapitulate the human pathology has contributed to the limited availability of effective therapeutic options to reverse liver fibrosis. In that regard, the contribution of S100A10/ANXA2 tetramer in hepatic stellate cell activation has been investigated using Human Liver Organoids (HLOs) and the LX-2 cell line.

**Results**

Using a 21-days differentiation process of embryonic stem cells, we generated multilineage HLOs exhibiting key liver functions, including lipid and amino acid metabolism, urea synthesis or glycogen storage. Diverse stimuli were tested to establish MASLD/MASH-HLOs, recapitulating triglyceride accumulation, stellate cell activation and collagen deposition. We evaluated the effect of A2ti-1, a small-molecule inhibitor of S100A10/ANXA2 heterotetramerization. Treatment of multilineage HLOs with A2ti-1 reduced hepatic stellate cell activation and subsequent fibrosis deposition in both preventive and curative settings. Finally, a direct effect of A2ti-1 on TGF-β1-induced hepatic stellate cells activation through a non-canonical, SMAD-independent pathway was demonstrated.

**Impact**

The use of S100A10/ANXA2 inhibitors such as A2ti-1 opens new avenues for the treatment of hepatic fibrosis and may potentially be extended to other fibrotic diseases. These findings also provide new evidence supporting the value of multilineage organoids in preclinical research, helping animal use and overcome interspecies differences.

## Introduction

Metabolic dysfunction-Associated Steatotic Liver Disease (MASLD), previously referred as Non-Alcoholic Fatty Liver Disease (NAFLD), affects more than 30% of the world adult population (Miao et al, 2024), making it the most prevalent cause of chronic liver diseases. MASLD begins with ectopic lipid accumulation in the hepatocytes and, if persistent, promotes hepatocyte damage, inflammation, and fibrosis; hallmarks of Metabolic dysfunction-Associated SteatoHepatitis (MASH) (Li et al, 2024). Following hepatocyte death and the release of pro-inflammatory signals, including TGF-β1, hepatic stellate cells (HSCs) are activated. Once activated, they express α-smooth muscle actin (α-SMA), and acquire a myofibroblast phenotype, representing the key source of fibrotic extracellular matrix (Hernandez-Gea and Friedman, 2011). Progressive fibrosis can lead to cirrhosis and substantially increase the risk of hepatocellular carcinoma (HCC), which is the third leading cause of cancer-related mortality worldwide (Sung et al, 2021). Importantly, fibrosis stage is the strongest predictor of liver-related morbidity and mortality, and clinical evidence indicates that once advanced fibrosis develops, disease progression becomes largely irreversible with poor therapeutic responsiveness (Dulai et al, 2017; Soon and Wee, 2021).

Although recently FDA-approved thyroid receptor ß-agonist (resmetirom) and glucagon-like peptide-1 receptor agonists (GLP-1RAs) have demonstrated therapeutic promises in MASLD, their efficacy in fibrosis resolution remains variable, and concerns regarding long-term safety have been raised (Harrison et al, 2024; Wang et al, 2025). Thus, identifying new molecular drivers of HSC activation and fibrosis progression remains a priority.

In this context, we recently identified S100A10 as a driver of MASLD/MASH pathophysiology (Delangre et al, 2025). Hepatocyte-specific downregulation of S100A10 in diet-induced or genetic mouse model of MASLD highlighted a role for S100A10 in hepatic steatosis and fibrosis development (Delangre et al, 2025), although the underlying mechanisms are unresolved. S100A10 forms the heterotetrameric complex A2t with Annexin A2 (ANXA2) and most of its described functions are attributed to this tetrameric complex (Okura et al, 2023). A2t is involved in several physiological processes, including plasminogen activation, membrane repair/vesicle fusion, and macrophage activation/recruitment (Lim and Hajjar, 2021; Okura et al, 2023; Swisher et al, 2010), while its potential role in MASLD/MASH pathogenesis is unknown. In parallel, ANXA2 has been linked to hepatic fibrosis development in vivo in the CCl_4_ model (Yang et al, 2017) and in diet-based MASH rodent models (Wang et al, 2022) through hepatocyte-dependent mechanisms. However, whether the S100A10-ANXA2 tetramer has a direct effect on hepatic stellate cells remains to be investigated.

Based on our recent findings in hepatocyte-specific S100A10 knockdown mice (Delangre et al, 2025), and the reported profibrotic role of ANXA2 in MASH (Wang et al, 2022; Yang et al, 2017), we hypothesized that S100A10-ANXA2 heterotetramer could control fibrosis development in MASLD/MASH, and that pharmacological disruption of this complex could represent a therapeutic strategy. To this end, we used selective S100A10-ANXA2 tetramer inhibitor (A2ti-1) that prevents protein-protein interaction between S100A10 and ANXA2 (Koh et al, 2024; Patton et al, 2025; Reddy et al, 2012; Weng et al, 2023; Woodham et al, 2015).

Studying liver pathologies requires an appropriate disease modelling system that can accurately mimic the complex physiology and pathophysiology of the liver. Unfortunately, clinically meaningful progress in this field has been hampered by the limited physiologically relevant models. While traditional 2D-cultured cell lines lack the hetero- and multicellular interactions (Kaur et al, 2023), rodent models may fail to fully recapitulate human metabolic and fibrotic responses due to interspecies differences (Delire et al, 2015). In this regard, recent generation of multilineage human liver organoids (HLOs) derived from human pluripotent stem cells opens new perspectives (Hess et al, 2023; Osonoi and Takebe, 2024; Ouchi et al, 2019). Organoids bridge the gap between traditional 2D cell systems and in vivo models, by incorporating multiple liver cell types, recapitulating aspects of 3D liver architecture and function, and retaining the human genetic background (Kaur et al, 2023; Shao et al, 2025).

In this study, we investigated the role of S100A10-ANXA2 tetramer in MASLD/MASH pathogenesis and evaluated the therapeutic potential of its pharmacological disruption. First, we examined the impact of A2ti-1 on TGF-β1 induced activation of an HSC line. Then, we leveraged HLOs to model steatosis, fibrosis, and steatohepatitis on a human genetic background to evaluate the effects of A2t inhibition across disease stages. Finally, we explored the signalling pathways underlying A2ti-1 mediated anti-fibrotic effects in HSCs. Together, this approach uncovered the role of the S100A10-ANXA2 tetramer as a molecular driver of fibrosis, opening new avenues for translational potential through its targeted disruption.

## Results

### Disruption of the S100A10-Annexin A2 tetramer (A2t) attenuates TGF-β1-induced fibrotic activation in hepatic stellate cells

**Figure EV1:**
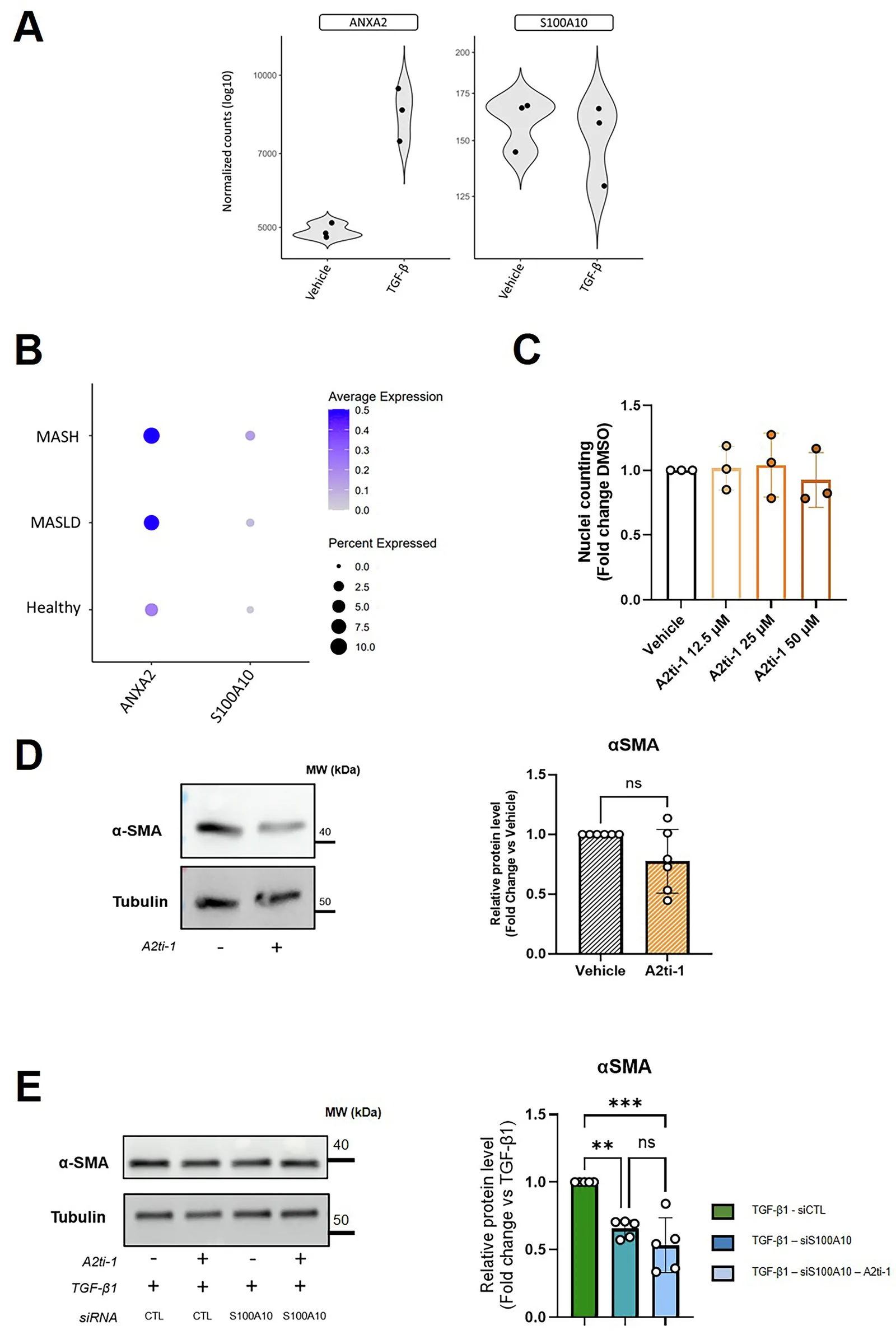
The expression of ANXA2 and S100A10 in human hepatic stellate cells (HSCs) in vitro and in vivo. (**A**) ANXA2 and S100A10 expression in primary human HSCs with or without TGF-β stimulation. Violin plots show normalized RNA-seq counts for ANXA2 and S100A10 in primary human HSCs reflecting the relative abundance of ANXA2 and S100A10 transcripts. Human HSCs cultured under solvent (Vehicle) or treated with TGF-β (10 ng/ml) for 24 h (Data ref: GEO GSE253493, Ma et al. 2024). Individual dots represent biological replicates (*n* = 3 per condition). Expression values are shown on a log10 scale. (**B**) Expression of ANXA2 and S100A10 in human hepatic stellate cells in vivo. Dot plots showing expression of ANXA2 and S100A10 in human HSCs across disease conditions (Healthy, MASLD, MASH), derived from single-nucleus RNA-sequencing data (Data ref: GEO GSE244832, 2025 (Kim et al, 2025)). Each dot represents the aggregated expression within the HSC population for a given condition. Dot size indicates the percentage of HSCs expressing the gene, while dot colour reflects average normalized expression level. (**C**) Automatic nuclei counting of LX-2 cells (*n* = 3) treated with increasing concentrations of A2ti-1 (12.5, 25 or 50 µM) for 2 days, measured on ImageXpress. Data are expressed as fold change versus Vehicle condition. Triplicates have been performed for each biological replicate (*n* = 3). (**D**) Representative Western blot (left) and quantification (right) of α-SMA levels in LX-2 cells treated with or without A2ti-1 (50 μM) for 2 days (*n* = 6). Tubulin is used as loading control. (**E**) Representative Western blot (left) and quantification (right) of α-SMA protein levels in LX-2 cells transfected 3 days with siRNA control (siCTL) or siRNA direct against S100A10 (siS100A10) treated with or without A2ti-1 (50 μM) and TGF-β1 (5 ng/ml) for 2 days (*n* = 5). Tubulin is used as loading control. Data are presented as mean ± SD, expressed as fold change relative to the indicated control where applicable. Statistical significance was assessed using Mann–Whitney test or one-way ANOVA followed by Tukey’s multiple comparisons test. ∗∗*P* < 0.01, ∗∗∗*P* < 0.001, ns=not significant. “*n*” represents the number of independent experiments, performed at different passages.

Fibrosis represents the key transition point at which MASLD becomes progressively hardly reversible (Dulai et al, 2017). Activated hepatic stellate cells are the principal source of extracellular matrix deposit in the fibrotic liver. S100A10 and ANXA2 expressions in HSCs and their regulation during activation or disease progression remains insufficiently characterized. Here, we re-analyzed a publicly available RNA-seq dataset (Data ref: GEO GSE253493, 2024 (Ma et al, 2024)) of primary human HSCs cultured under basal conditions or stimulated with TGF-β1. ANXA2 transcripts were abundantly expressed in human primary HSCs and increased upon TGF-β1 activation (Fig. EV1A). In contrast, S100A10 transcripts were detected at low levels and not significantly affected by TGF-β1 stimulation. To assess their expression in vivo, we additionally analyzed a human single-nucleus RNA-sequencing (snRNA-seq) dataset comprising liver samples from healthy individuals and patients suffering from MASLD or MASH (Data ref: GEO GSE244832, 2025 (Kim et al, 2025)). Here again, ANXA2 was consistently detected in HSCs among all subjects, with modest expression that tended to increase with disease severity (Fig. EV1B). S100A10 was present in a smaller fraction of HSCs, although at lower levels, detectable in both health and disease states, substantiating its expression in human HSCs. Given that both ANXA2 and S100A10 are expressed, we hypothesized that the ANXA2-S100A10 tetramer (A2t) may play a functional role in HSC activation and fibrogenic responses.

**Figure 1:**
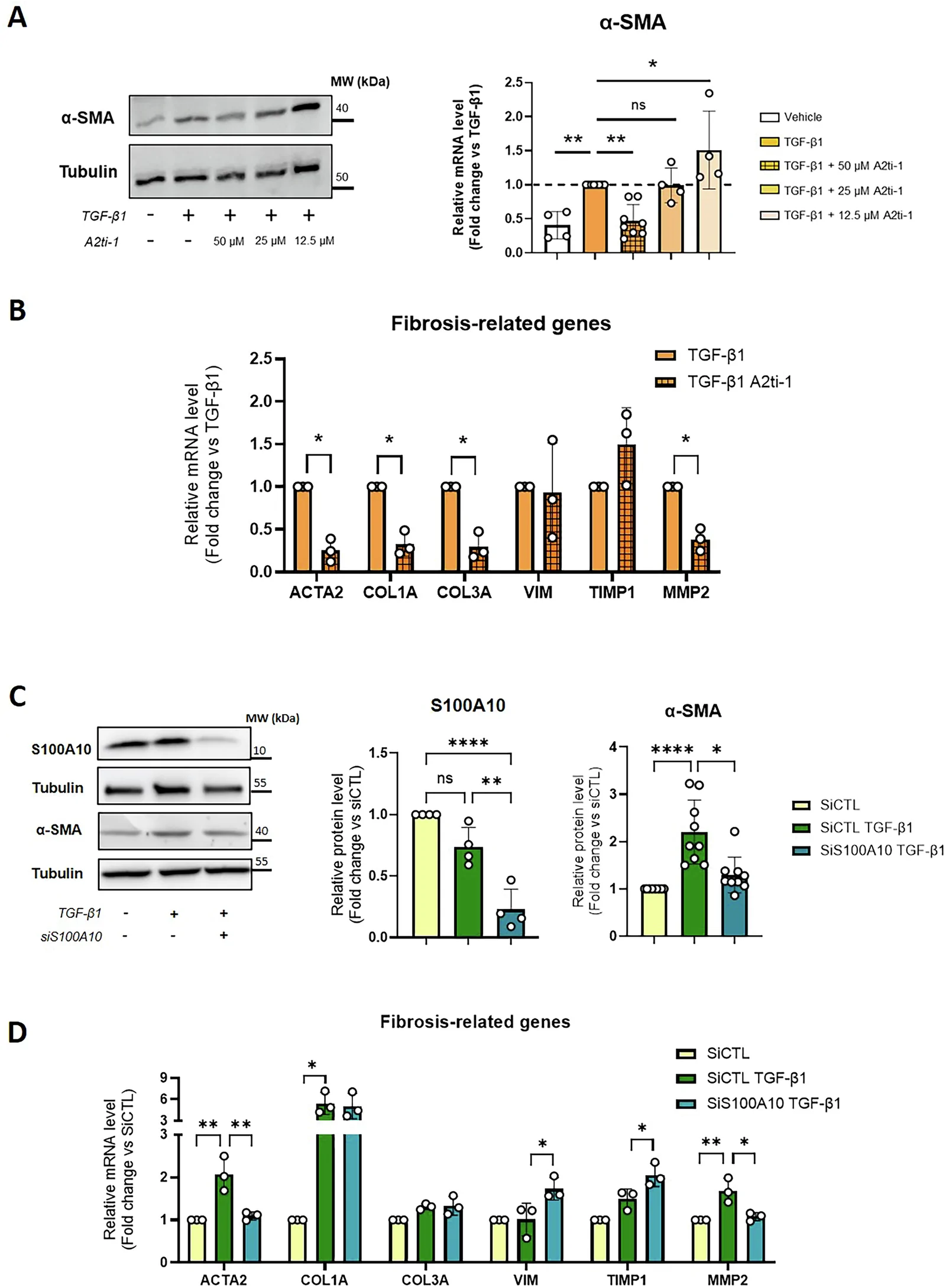
Disruption of the S100A10-Annexin A2 tetramer attenuates TGF-β1 induced fibrotic activation in hepatic stellate cells. (**A**) Representative Western blot images (left) and quantification (right) of α-SMA protein levels in LX-2 cells treated with TGF-β1 (5 ng/ml) for 2 days in the absence or presence of A2ti-1 (12.5, 25 or 50 μM). Tubulin served as a loading control (*n* = 4–8). (**B**) Relative mRNA level of fibrosis-related genes (ACTA2, COL1A1, COL3A1, VIM, TIMP1, MMP2) in TGF-β1 (5 ng/ml) treated LX-2 cells with or without A2ti-1 (50 μM) treatment (*n* = 3). Cyclophilin A and TBP were used as housekeeping genes. Technical triplicates have been performed for the qPCR. (**C**) Representative Western blot images (left) and quantification (right) of S100A10 and α-SMA protein levels following transfection with control siRNA (siCTL) or S100A10-targeting siRNA (siS100A10) in TGF-β1 (5 ng/ml) treated LX-2 cells (*n* = 4 for S100A10 and *n* = 9 for α-SMA protein quantifications, respectively). Tubulin served as a loading control. (**D**) Relative mRNA level of fibrosis-related genes (ACTA2, COL1A1, COL3A1, VIM, TIMP1, MMP2) in TGF-β1 (5 ng/ml) stimulated LX-2 cells transfected with siCTL or siS100A10 (*n* = 3). Cyclophilin A and TBP were used as housekeeping genes. Technical triplicates have been performed for the qPCR. Data are presented as mean ± SD, expressed as fold change relative to the indicated control where applicable. Statistical significance was assessed using unpaired two-tailed Student’s *t* test with Welch’s correction, one-way ANOVA followed by Sidak’s multiple comparisons test or Kruskal–Wallis followed by Dunn’s multiple comparisons when data did not pass normality assumptions. “*n*” represents the number of independent experiments, performed at different passages. ∗*P* < 0.05, ∗∗*P* < 0.01, ∗∗∗∗*P* < 0.0001. ns not significant. Source data are available online for this figure.

To test this hypothesis, we selectively disrupted A2t using the small molecule inhibitor A2ti-1 (Koh et al, 2024; Patton et al, 2025; Reddy et al, 2012; Weng et al, 2023; Woodham et al, 2015), or achieved siRNA-based S100A10 silencing in the human HSC line LX-2. A dose-response study was performed for A2ti-1 showing no significant cytotoxicity (Fig. EV1C). We first assessed the HSC activation by measuring α-Smooth Muscle Actin (α-SMA) levels, a marker of HSC activation (Fig. 1A) (Washington et al, 2000). As witnessed by the reduction of α-SMA protein levels, 50 µM A2ti-1 significantly attenuated TGF-β1-driven HSC activation. Lower doses of A2ti-1 did not alter the phenotype, 12.5 µM showing an unexpected enhancement of α-SMA levels (Fig. 1A). Background induction of α-SMA in LX-2 cells, without addition of exogenous TGF-β1, also tended to be reduced by 50 μM A2ti-1 (Fig. EV1D). In addition, A2ti-1 treatment reduced the expression of profibrotic genes ACTA2, COL1A1, COL3A1 and MMP2 (Fig. 1B), although mRNA levels of VIM and TIMP1 was not significantly altered.

To validate the role of S100A10 in HSC activation, we performed efficient knockdown of S100A10. This resulted in a ~50% reduction in α-SMA protein levels in TGF-β1 activated LX-2 cells, limiting its level to that of control conditions (Fig. 1C). qPCR analysis confirmed reduced ACTA2 and MMP2 mRNA levels upon S100A10 knockdown, while COL1A1 and COL3A1 were unchanged (Fig. 1D). These results reveal slight differences between S100A10 gene silencing on one hand and A2t pharmacological disruption on the other, none of them resulting in complete ablation of the A2t complex resulting in residual background activation. Of note, the on-target effect of A2ti-1 was further assessed by co-treating LX-2 cells with siRNAs and A2ti-1 together, without significant cumulative effects on α-SMA protein levels (Fig. EV1E).

Taken together, these results revealed that S100A10-Annexin A2 tetramer contributes to fibrotic activation of HSCs. Moreover, disrupting A2t efficiently prevents TGF-β1 induced HSC activation, highlighting its role in fibrosis progression.

### Human liver organoids (HLOs) recapitulate key features of hepatic cell diversity and function

**Figure 2:**
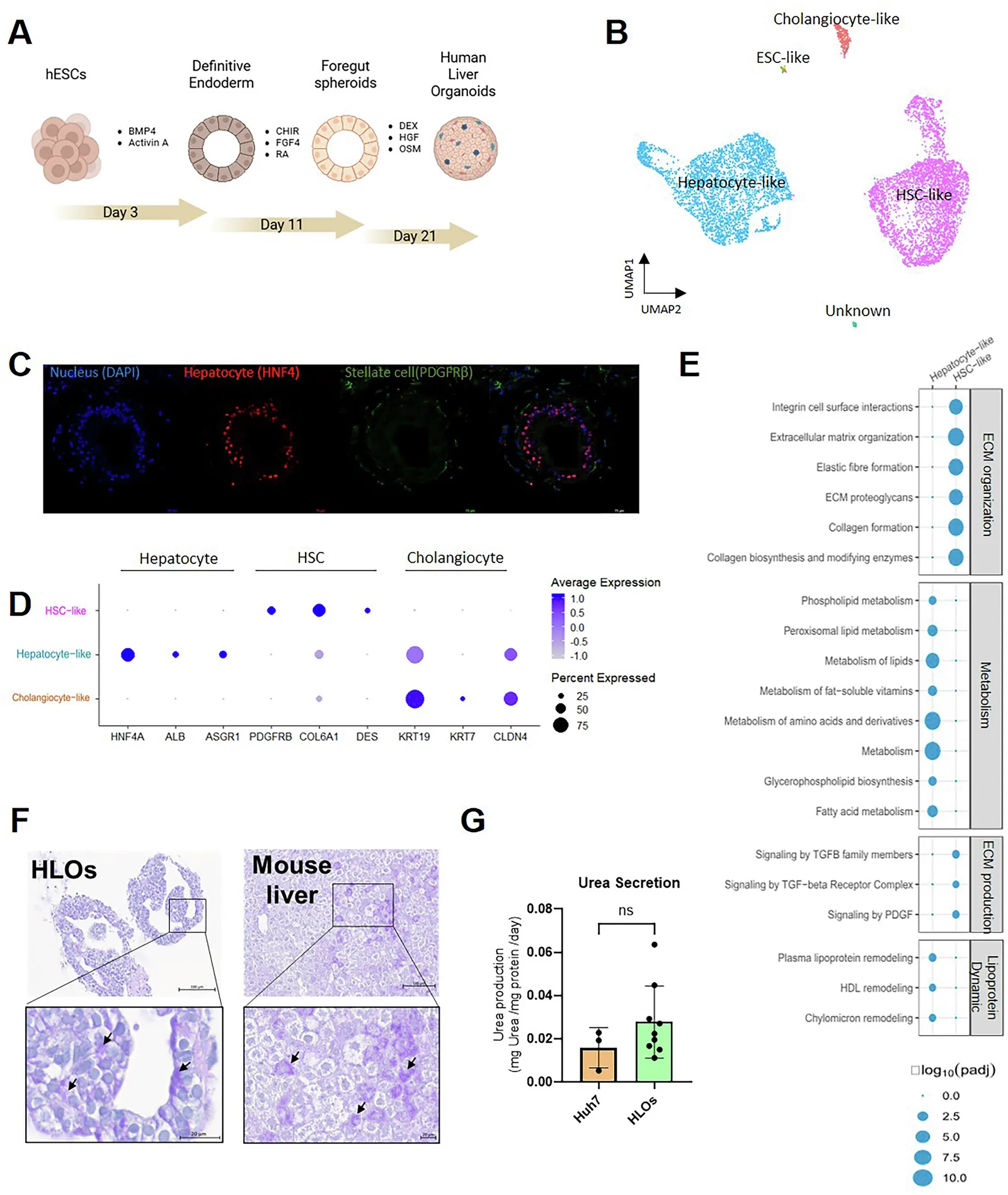
Human Liver Organoids (HLOs) recapitulate key features of hepatic cell diversity and function. (**A**) Schematic representation of the stepwise differentiation protocol for HLO generation. Human embryonic stem cells (hESCs) were first differentiated into definitive endoderm and subsequently to foregut, from which liver organoids were formed. (**B**) Single-cell RNA sequencing (scRNA-seq) analysis of day 21 HLOs. UMAP plots show clustering of distinct cell populations, annotated based on known marker genes (Hess et al, 2023). (**C**) Representative confocal images of day 21 HLOs stained for HNF4α (red, hepatocyte-like cells), PDGFRβ (green, stellate-like cells) and Hoechst (blue, nuclei). Images were taken with Stellaris confocal microscope (Leica) at ×40 magnification. (**D**) Dot plot showing the expression of canonical liver cell-type marker genes across clusters identified from scRNA-seq. Circle size represents the proportion of cells within each cluster expressing the gene, while circle colour indicates normalized average expression levels. (**E**) Dot plot showing selected Reactome pathways significantly enriched in hepatocyte- and HSC-like cells. The y-axis lists the pathways grouped by functional categories, while the x-axis represents the two main cell populations. The size of each circle corresponds to the log10 adjusted *P* value, reflecting the significance of enrichment. (**F**) Representative Periodic acid-Schiff (PAS) staining in HLOs (left) and mouse liver (right) histological sections. Arrows indicate glycogen-positive (dark purple) regions. Images were taken with AxioScan Z.1 slide scanner (Zeiss) at 200x magnification. (**G**) Urea quantification from day 21 HLO supernatants (*n* = 9) compared to Huh7 hepatoma cell line (*n* = 3). Data are normalized to the amount of protein. Technical duplicates have been performed for this assay. Data are shown as mean ± SD. ns = no significance, determined by unpaired two-tailed Student’s *t* test. “*n*” represents the number of independent experiments, corresponding to independent HLOs differentiation or independent Huh7 passages. Source data are available online for this figure.

**Figure EV2:**
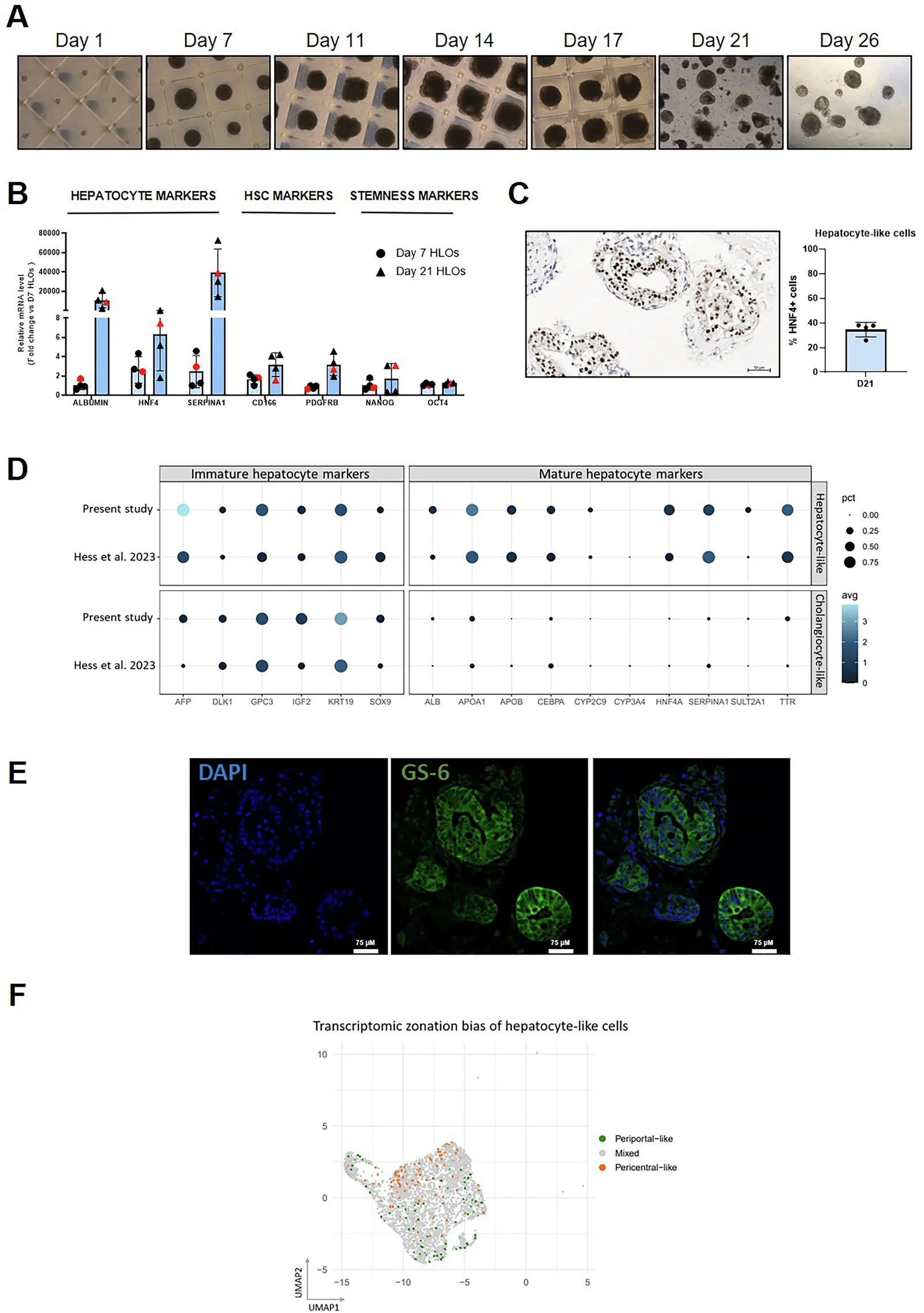
Generation and characterization of human liver organoids. (**A**) Representative bright-field images (5X) showing different stages of HLO differentiation from human embryonic stem cells (hESCs). (**B**) Quantitative PCR (qPCR) analysis of different cell types and progenitor marker gene expression in Day 7 HLOs and Day 21 HLOs (*n* = 4). Expression values are shown as fold change relative to Day 7 HLOs. The red data point indicates the HLO sample used for single-cell RNA sequencing. Cyclophilin A and TBP was used as housekeeping genes. (**C**) Representative picture (left) and quantification (right) of HNF4 staining performed in HLOs after 21 days of differentiation (*n* = 4). Data are expressed as percentage of HNF4-positive cells on total number of cells. Images were taken at ×200 magnification. (**D**) Single-cell transcriptomic comparison of hepatocyte-like cells at day 21. Dot plot comparing the expression of representative immature/hepatoblast (AFP, DLK1, GPC3, IGF2, KRT19, SOX9) and mature hepatocyte markers (ALB, APOA1, APOB, CEBPA, CYP2C9, CYP3A4, HNF4A, SERPINA1, SULT2A1, TTR) in hepatocyte-like clusters from D21 HLOs generated in present study and from a previously published D21 HLO scRNA-seq dataset (Data ref: GEO GSE207889, 2023) (Hess et al, 2023). Cholangiocyte-like cells are included to demonstrate that hepatocyte markers are not ubiquitously expressed in the dataset. Dot size indicates the fraction of cells with detectable expression (expression >0), and colour indicates the mean log-normalized expression across hepatocyte-like cells. Datasets were analysed separately. One limitation of this comparison is that the datasets may not be normalized identically as we used the provided normalized values from Hess et al 2023. (**E**) Representative confocal images of Day 21 HLOs stained for GS-6 (green, pericentral hepatocyte marker) and Hoechst (blue, nuclei). Images were taken with Stellaris confocal microscope (Leica) at ×400 magnification. (**F**) UMAP visualization of hepatocyte-like cells coloured according to transcriptional zonation bias. A continuous porto-central coordinate was computed from single-cell RNA-seq data using module scores for periportal (zone 1) and pericentral (zone 3) gene signatures derived from human liver datasets (MacParland et al, 2018). The zonation axis represents the standardized difference between pericentral and periportal scores. Cells were classified using a stringent threshold ( ± 1.75 SD) into periportal-like (green), pericentral-like (orange), or mixed (grey) populations. Data in (**B**, **C**) are presented as mean ± SD. “*n*” corresponds to an independent human liver organoid (HLO) batch.

Because A2ti-1 attenuated fibrotic activation in LX-2 cells, we next sought to evaluate its effects in a more physiologically relevant multicellular system that better mimics the structural and cellular complexity of the human liver. To this end, we first generated HLOs through stepwise differentiation of human embryonic stem cells (hESCs) using a modified version of a well-established protocol (Figs. 2A and  EV2A) (Correia de Sousa et al, 2024; Ouchi et al, 2019). We implemented an air–liquid interface culture to optimize the nutrition and gas exchange of cultured HLOs (El Harane et al, 2025) (Fig. EV2A).

To determine the cellular composition of the HLOs, single-cell RNA sequencing was performed (scRNA-seq). UMAP analysis revealed three major distinct clusters corresponding to respectively hepatocyte-like cells, hepatic stellate cells (HSC)-like and cholangiocytes-like cells (Fig. 2B). Cell identities were assigned using ScType annotations from the published day 21 HLO reference dataset (Data ref: GEO GSE207889, 2023), which was selected by Hess et al (Hess et al, 2023), as the most robust annotation method after comparing marker-based, CellTypist, and ScType classifications.

To ensure that the sample used for scRNA-seq analysis was representative, we performed quantitative PCR (qPCR) analysis of key hepatic differentiation markers across multiple HLO preparations. The expression profile of the HLOs selected for sequencing (indicated in red) was consistent with the other differentiations, confirming its relevance as a representative sample (Fig. EV2B). HNF4α staining of D21 HLOs in four independent batches also underscores the batch-to-batch reproducibility regarding the hepatocyte-like population (Fig. EV2C). Additionally, we compared hepatocyte-like cells from D21 HLOs with those from a previously published D21 HLO scRNA-seq study (Hess et al, 2023 (Hess et al, 2023)) by examining established immature/hepatoblast and mature hepatocyte marker expression (Fig. EV2D). Both datasets showed concurrent expression of mature markers (e.g., ALB, HNF4A, APOB) and fetal/immature markers (e.g., AFP, IGF2), indicating a comparable intermediate hepatocyte-like maturation state at this stage. While minor quantitative differences in expression levels and in the fraction of expressing cells were observed, the overall transcriptional profiles were highly similar between studies. Although HLOs used in the present study express mature hepatocyte-specific genes, residual expression of the progenitor-associated marker KRT19 indicates that hepatocyte-like cells retain features of developmental immaturity at day 21.

Cluster identities were further confirmed by immunofluorescent staining of HNF4α (hepatocyte marker) and PDGFRβ (HSC marker) (Fig. 2C). Furthermore, hepatocyte-like cells exhibited small zonation features, expressing the pericentral marker glutamine synthetase (GS-6) (Fig. EV2E). Despite detectable GS expression, hepatocyte-like cells predominantly exhibited a mixed transcriptional pattern (Fig. EV2F). This indicates that most hepatocyte-like cells lack a strong transcriptional program toward either periportal or pericentral programs, consistent with incomplete zonation rather than mature spatial segregation.

Marker gene analysis showed that hepatocyte-like cells were enriched in hepatocyte markers such as HNF4A, ALB and ASGR1 (Fig. 2D). The HSC-like cluster expressed HSC markers including PDGFRB, COL6A1 and DES. Lastly, cholangiocyte-like cluster exhibited enrichment in cholangiocyte markers KRT19, KRT7, and CLDN4 (Fig. 2D). Pathway enrichment analysis highlighted metabolic differentiation in the hepatocyte-like cluster, including free fatty acid (FFA) and amino acid/vitamin metabolisms, as well as lipoprotein remodelling. Moreover, HSC-like clusters were enriched in components of ECM organization, collagen formation and TGF-β signalling processes (Fig. 2E; Dataset EV1). In addition to their liver-like transcriptional profiles, HLOs exhibited some of the key liver functions. Periodic Acid Schiff (PAS) staining of HLOs revealed glycogen storage patterns similar to those observed in mouse liver tissue (Fig. 2F). Finally, urea production from HLOs was comparable to that of the widely used Huh7 hepatoma cell line, indicating successful induction of hepatocyte fundamental function (Fig. 2G).

Together, these results demonstrate that HLOs contained specialized hepatocyte-, HSC-, and cholangiocyte-like cell populations with appropriate lineage transcriptional programs and liver-specific metabolic activity.

### Hallmarks of MASLD progression can be induced in human liver organoids

**Figure 3:**
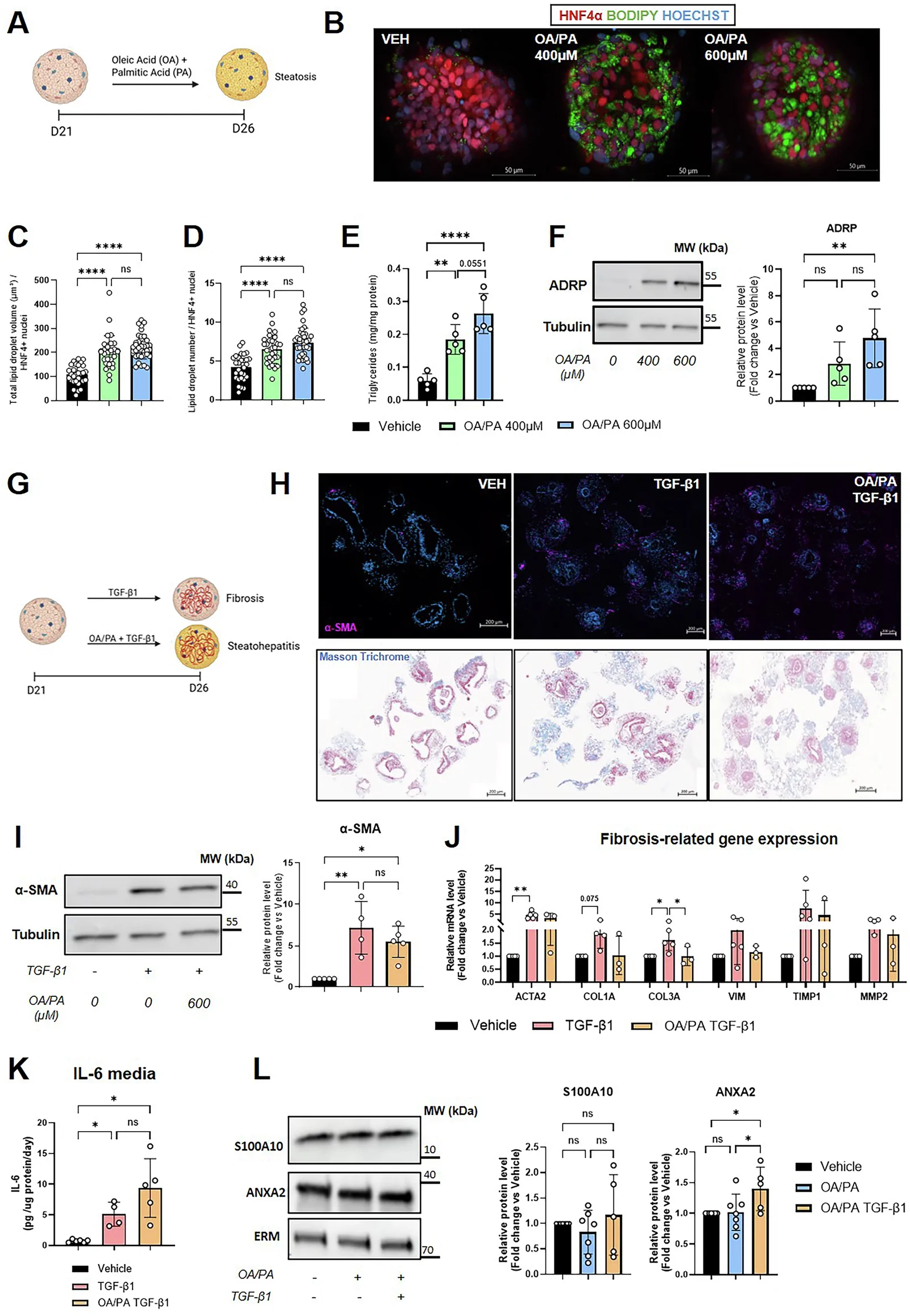
Hallmarks of MASLD progression can be induced in human liver organoids. (**A**) Schematic representation of steatosis induction protocol in HLOs by oleic and palmitic acid treatment for 5 days. (**B**) Representative images of BODIPY staining (green), Hoechst staining (blue) and HNF4 immunofluorescence (red) in HLOs treated 5 days with vehicle (left), OA/PA (400 μM, middle), or OA/PA (600 μM, right). (**C**) Quantification of total lipid droplet (LD) volume per HNF4α⁺ nucleus (hepatocytes) in HLOs treated 5 days with vehicle, OA/PA (400 μM), or OA/PA (600 μM). Every dot represents an HLO. Three independent experiments corresponding to 3 HLOs batches have been performed. (**D**) Quantification of lipid droplet number per HNF4α⁺ nucleus (hepatocytes) per HLO. Every dot represents an HLO. Three independent experiments corresponding to 3 HLOs batches have been performed. (**E**) Intracellular Triglyceride content measured in HLOs treated 5 days with vehicle (*n* = 5), OA/PA 400 µM (*n* = 5) or OA/PA 600 µM (*n* = 5). Three technical replicates have been performed for this set of data. (**F**) Representative Western blot images (left) and quantification (right) of ADRP protein level in HLOs treated 5 days with vehicle (*n* = 5), OA/PA 400 μM (*n* = 5) or OA/PA 600 μM (*n* = 5). Tubulin was used as a loading control. (**G**) Schematic representation of fibrosis or steatohepatitis induction protocol in HLOs following 5 days treatment with TGF-β1 (20 ng/ml) or OA/PA (600 µM) + TGF-β1 (20 ng/ml). (**H**) Representative pictures of α-SMA immunofluorescence (upper panel) or Masson Trichrome coloration (lower panel) performed in HLOs treated 5 days with vehicle (left) TGF-β1 (20 ng/ml, middle) or OA/PA (600 µM) + TGF-β1 (20 ng/ml) (right). Images were taken with AxioScan Z.1 slide scanner (Zeiss) at ×20 magnification. (**I**) Representative Western blot images (left) and quantification (right) of α-SMA protein level measured in HLOs treated 5 days with vehicle (*n* = 5), TGF-β1 (20 ng/ml, *n* = 4), and OA/PA (600 µM) + TGF-β1 (20 ng/ml) (*n* = 5). Tubulin is used as a loading control. (**J**) Relative mRNA level of fibrosis-related gene expression in HLOs treated with TGF-β1 (20 ng/ml) (*n* = 5) with or OA/PA (400 μM/200 μM) + TGF-β1 (20 ng/ml) (*n* = 3) for 5 days. Fibrosis-related genes are shown as fold change relative to vehicle (*n* = 5). Cyclophilin A and TBP were used as housekeeping genes. Technical triplicates have been performed for the qPCR. (**K**) ELISA measurement of IL-6 levels in supernatants of HLOs treated with vehicle (*n* = 6), TGF-β1(20 ng/ml) (*n* = 4) or OA/PA (600 µM) + TGF-β1(20 ng/ml) (*n* = 5). (**L**) Representative Western blot images (left) and quantification (right) of S100A10 and ANXA2 protein level measured in HLOs treated 5 days with vehicle (*n* = 7), (600 µM) (*n* = 7), and OA/PA (600 µM) + TGF-β1 (20 ng/ml) (*n* = 7). ERM is used as a loading control. Data are represented as mean ± SD and expressed as fold change to respective control when indicated. ∗*P* < 0.05, ∗∗*P* < 0.01, ∗∗∗∗*P* < 0.0001, ns = not significant. Statistical significance was determined by one-way ANOVA followed by Sidak’s or Dunnett’s multiple comparisons test or Kruskal–Wallis followed by Dunn’s multiple comparisons when data did not pass normality assumptions. With the exception of (**C**, **D**), each ‘*n*’ corresponds to an independent human liver organoid (HLO) batch. Source data are available online for this figure.

To establish in vitro MASLD modelling in a multicellular context, we leveraged the multilineage nature of HLOs to induce steatosis, fibrosis, and steatohepatitis. For steatosis modelling, HLOs were exposed to various concentrations of oleic and palmitic acids (OA/PA; 400 or 600 µM in 2:1 ratio) for 5 days (Fig. 3A). BODIPY staining of neutral lipids showed a robust increase in lipid droplet accumulation in HLO’s hepatocytes (Fig. 3B–D) upon exposure to 400 or 600 µM OA/PA. These results were further substantiated by the observed increase in triglycerides and ADRP (Perilipin 2) protein levels (a lipid droplet-associated protein) within HLOs treated with OA/PA (Fig. 3E,F). Finally, the more potent effect of the 600 µM OA/PA condition was selected for subsequent experiments.

To model more advanced stages of MASLD, which include inflammation and fibrosis, HLOs were next treated with either TGF-β1 alone (fibrosis model) or in combination with OA/PA (steatohepatitis model) for 5 days (Fig. 3G). α-SMA was significantly upregulated at the protein level in both TGF-β1 and OA/PA TGF-β1 treated groups, as shown by immunofluorescent staining and western blot analysis (Fig. 3H,I). Masson’s trichrome coloration further revealed enhanced collagen deposition in these groups (Fig. 3H), uncovering end-stage fibrosis development. Interestingly, under OA/PA TGF-β1 conditions, ACTA2/α-SMA was the only fibrotic marker induced at the transcriptional level, whereas canonical extracellular matrix genes such as COL1A1 and COL3A1 were not upregulated (Fig. 3J). TIMP1 and MMP2 displayed modest upward trends without reaching significance. This difference in the responses is substantiated by the weaker Masson’s trichrome coloration in the OA/PA TGF-β1 condition as compared to the TGF-β1 condition.

Activated HSCs and damaged hepatocytes can secrete pro-inflammatory cytokines which contribute to the inflammatory environment in MASH (Carter and Friedman, 2022). Accordingly, IL-6 secretion was assessed in the culture supernatants of HLOs, revealing a significant increase in IL-6 release following TGF-β1 and OA/PA TGF-β1 treatment (Fig. 3K). Notably, the total protein levels of S100A10 and ANXA2 in whole-HLO lysates were not altered in OA/PA treated conditions. However, OA/PA TGF-β1 treatment slightly increased ANXA2 protein level (Fig. 3L), aligned with literature (Wang et al, 2022; Zhang et al, 2010) and human datasets (Fig. EV1).

Collectively, these results suggest that HLOs are able to recapitulate key pathological features of MASLD/MASH, including steatosis, activation of hepatic stellate cells, collagen deposition, and IL-6 secretion. Therefore, HLOs provide a suitable multicellular system to evaluate the therapeutic potential of disrupting the S100A10-Annexin A2 tetramer.

### A2ti-1 treatment reduces fibrotic development in MASLD/MASH HLOs

**Figure 4:**
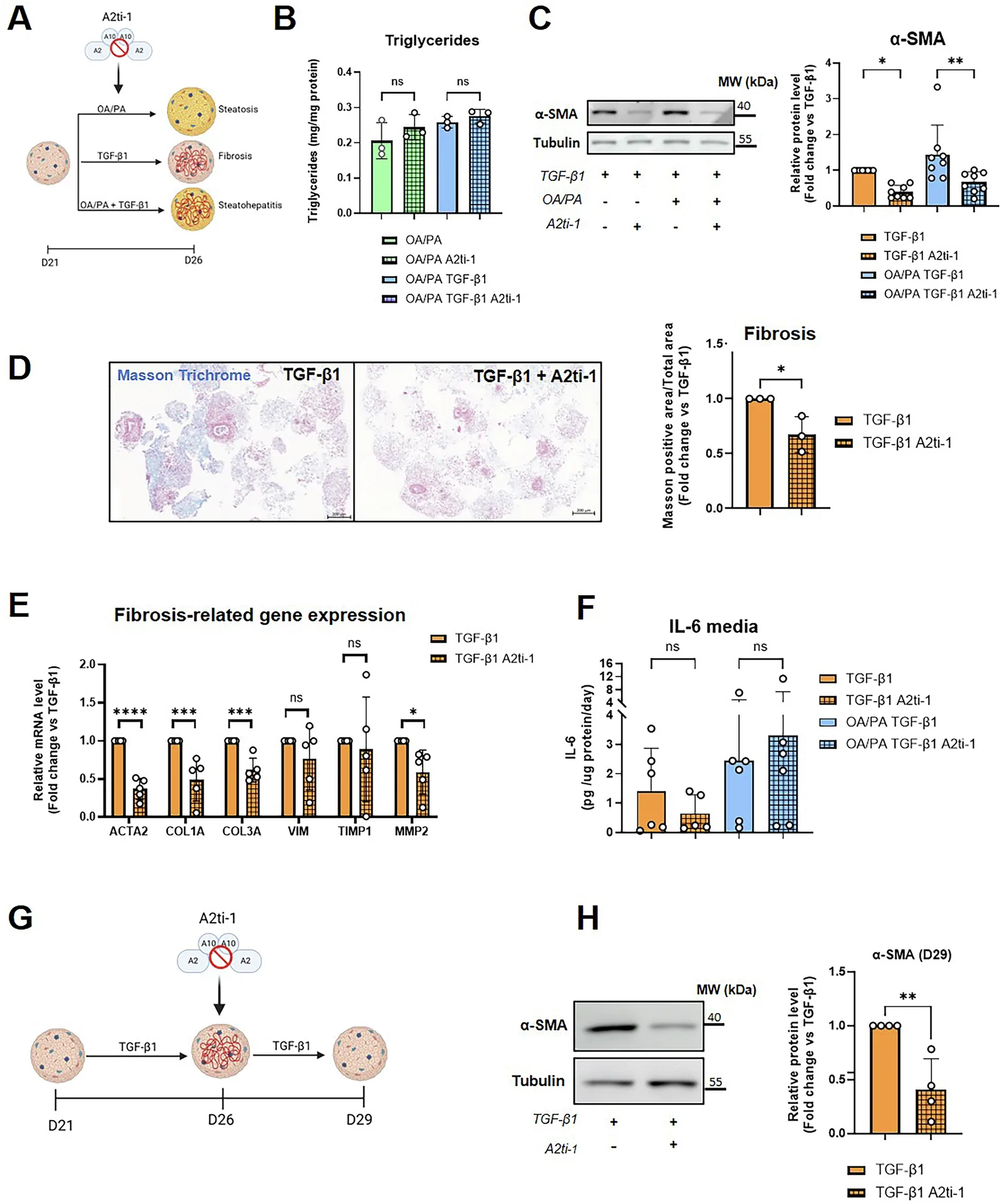
A2ti-1 treatment reduces fibrotic development in MASLD/MASH HLOs. (**A**) Schematic representation of MASH modelling HLOs and the A2ti-1 intervention strategy. (**B**) Intracellular triglyceride content measured in HLOs treated for 5 days with OA/PA (600 μM) (*n* = 3), OA/PA (600 μM) + A2ti-1 (50 μM) (*n* = 3), OA/PA (600 μM) + TGF-β1 (20 ng/ml) (*n* = 3) and OA/PA (600 μM) + TGF-β1 (20 ng/ml) + A2ti-1 (50 μM) (*n* = 3). Three technical replicates have been performed for this set of data. (**C**) Representative western blot (left) and quantification (right) of α-SMA protein level measured in HLOs treated for 5 days with TGF-β1 (20 ng/ml) (*n* = 8), TGF-β1(20 ng/ml) + A2ti-1 (50 μM) (*n* = 8), OA/PA (600 μM) + TGF-β1 (20 ng/ml) (*n* = 8) and OA/PA (600 μM) + TGF-β1 (20 ng/ml) + A2ti-1 (50 μM) (*n* = 8). Tubulin was used as a loading control. (**D**) Representative Masson Trichrome images of HLO treated for 5 days with TGF-β1 (20 ng/ml) (*n* = 3, left) and TGF-β1 (20 ng/ml) + A2ti-1 (50 μM) (*n* = 3, right). Images were taken with AxioScan Z.1 slide scanner (Zeiss) at ×20 magnification. Images were analyzed with QuPath and Masson’s trichrome-positive areas were delineated using pixel thresholding, and results were expressed as coverage of Masson-positive area over total area of the HLO cuts. (**E**) Relative mRNA level of fibrosis-related genes (ACTA2, COL1A1 and COL3A1, VIM, TIMP1, MMP2) measured by RT-qPCR in HLOs treated for 5 days with TGF-β1 (20 ng/ml) (*n* = 5), TGF-β1 + A2ti-1 (50 μM) (*n* = 5), OA/PA (600 μM) + TGF-β1 (20 ng/ml) (*n* = 5) and OA/PA (600 μM) + TGF-β1 (20 ng/ml) + A2ti-1 (50 μM) (*n* = 5). Cyclophilin A and TBP were used as housekeeping genes. Technical triplicates have been performed for the qPCR. (**F**) IL-6 secretion measured by ELISA in supernatants from HLOs treated for 5 days with TGF-β1 (20 ng/ml) (*n* = 6), TGF-β1 (20 ng/ml) + A2ti-1 (50 μM) (*n *= 6), OA/PA (600 μM) + TGF-β1 (20 ng/ml) (*n* = 7) and OA/PA (600 μM) + TGF-β1 (20 ng/ml) + A2ti-1 (50 μM) (*n* = 6). Technical duplicates have been performed for this assay. (**G**) Schematic representation of therapeutic intervention design in fibrotic HLOs with A2ti-1. First, fibrosis is induced for 5 days with TGF-β1, then A2ti-1 is added for additional 3 days in the presence of TGF-β1. (**H**) Representative Western blot (left) and quantification (right) of α-SMA protein level measured in HLOs treated 8 days with TGF-β1 (20 ng/ml) (*n* = 4) with or without 3 days treatment with A2ti-1 (50 μM) (*n* = 4). Tubulin is used as loading control. Data are presented as mean ± SD, expressed as fold change relative to the indicated control when applicable. Statistical significance was assessed using unpaired two-tailed Student’s *t* test, one-way ANOVA followed by Sidak’s multiple comparisons test, or Kruskal–Wallis followed by Dunn’s multiple comparisons test when data did not pass normality assumptions (**C** and Fig. 3F). ∗*P* < 0.05, ∗∗*P* < 0.01, ∗∗∗*P* < 0.001, ∗∗∗∗*P* < 0.0001, ns = not significant. Each ‘*n*’ corresponds to an independent human liver organoid (HLO) batch. Source data are available online for this figure.

**Figure EV3:**
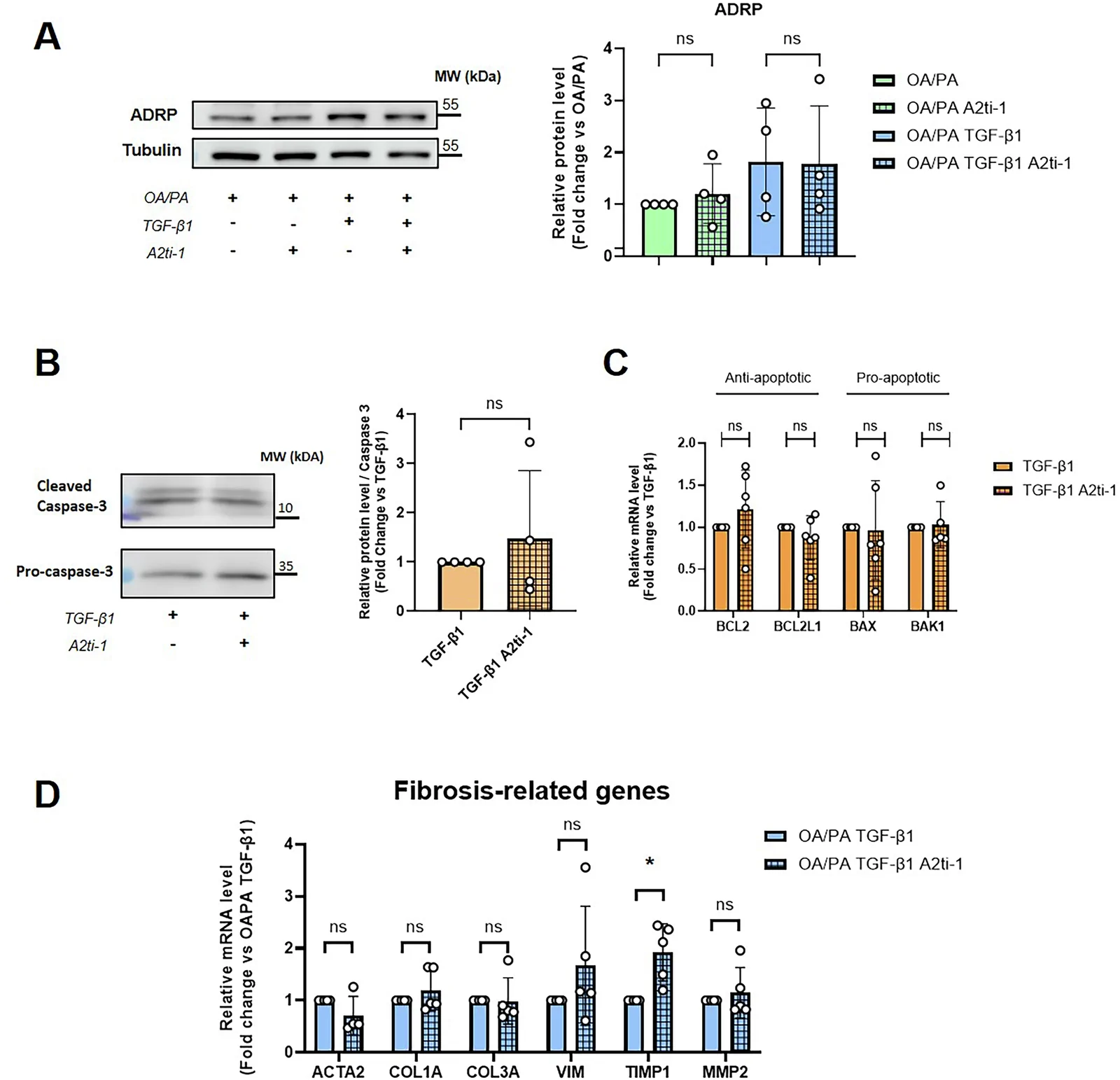
Effect of A2ti-1 on steatosis and steatohepatitis modelling HLOs. (**A**) Representative Western blot (left) and quantification (right) of ADRP protein level in HLOs treated with OA/PA (600 μM) ± TGF-β1 (20 ng/ml) and/or A2ti-1 (50 μM). Tubulin was used as a loading control (*n* = 4 for each group). (**B**) Representative Western blot (left) and quantification (right) of cleaved caspase 3 protein level in HLOs treated with TGF-β1 (20 ng/ml) with or without A2ti-1 (50 μM). Data are normalized on total caspase 3 levels (*n* = 4 for each group). (**C**) qPCR analysis of anti-apoptotic (BCL2 and BCL2L1) and pro-apoptotic genes (BAX and BAK1) in TGF-β1 (20 ng/ml) treated HLOs with or without A2ti-1 (50 μM). Cyclophilin A and TBP was used as housekeeping genes (*n* = 6 for each group). (**D**) qPCR analysis of fibrosis-related genes (ACTA2, COL1A1, COL3A1, VIM, TIMP1, MMP2) in OA/PA (600 μM) + TGF-β1 (20 ng/ml) treated HLOs ± A2ti-1 (50 μM). Cyclophilin A and TBP was used as housekeeping genes (*n* = 5 for each group). Technical triplicates have been performed for the qPCR. Data are presented as mean ± SD, expressed as fold change relative to the indicated control when applicable. Statistical significance was assessed using one-way ANOVA followed by Tukey’s multiple comparisons test and unpaired *t* test or Mann–Whitney test when data did not pass normality assumptions. ∗∗*P* < 0.01, ns = not significant. “*n*” corresponds to an independent human liver organoid (HLO) batch.

To assess the role of S100A10-ANXA2 tetramer in MASLD progression, we induced steatosis or fibrosis individually as well as steatohepatitis and simultaneously treated such MASLD modelling HLOs with A2ti-1 (Fig. 4A). Triglyceride content (Fig. 4B) and ADRP protein level (Fig. EV3A) remained unchanged upon A2ti-1 treatment, suggesting that functional A2t is not required for lipid accumulation in HLOs.

We next evaluated the effects of A2ti-1 on fibrosis development. Consistent with our observations in LX-2 cells, A2ti-1 treatment markedly reduced α-SMA protein levels in both TGF-β1 and OA/PA TGF-β1-treated HLOs (Fig. 4C), without noticeable pro-apoptotic effects (Fig. EV3B,C). Moreover, Masson’s trichrome staining showed reduced collagen deposition following A2ti-1 treatment in TGF-β1 exposed HLOs (Fig. 4D). Consistently, qPCR analysis of fibrosis associated genes revealed significant downregulation of ACTA2, COL1A1, COL3A1 and MMP2 transcripts in TGF-β1 treated HLOs following A2ti-1 treatment, while VIM and TIMP1 expression remained unchanged (Fig. 4E). Conversely, in HLOs steatohepatitis modelling, A2ti-1 treatment did not significantly affect expression of most of the profibrotic genes, except for an increase in TIMP1 expression (Fig. EV3D). ELISA measurement of IL-6 secretion showed no effect of A2ti-1 on cytokine release (Fig. 4F).

Because A2ti-1 could prevent fibrosis induction, we next tested its potential to reverse an established fibrotic feature. To this end, HLOs were first treated with TGF-β1 for 5 days, followed by an additional 3-day treatment with A2ti-1 in the continued presence of TGF-β1 (Fig. 4G). Remarkably, A2ti-1 administered after fibrosis induction significantly reduced α-SMA protein levels (Fig. 4H).

Overall, this set of data demonstrates that A2ti-1 can counter established fibrosis without altering lipid storage or IL-6 secretion. Importantly, A2ti-1 showed efficiency both in preventing fibrosis induction and in reversing induced fibrotic markers, highlighting its therapeutic potential for fibrosis resolution in MASLD/MASH.

### A2ti-1 suppresses TGF-β1 induced stellate cell activation through a SMAD-independent axis

**Figure 5:**
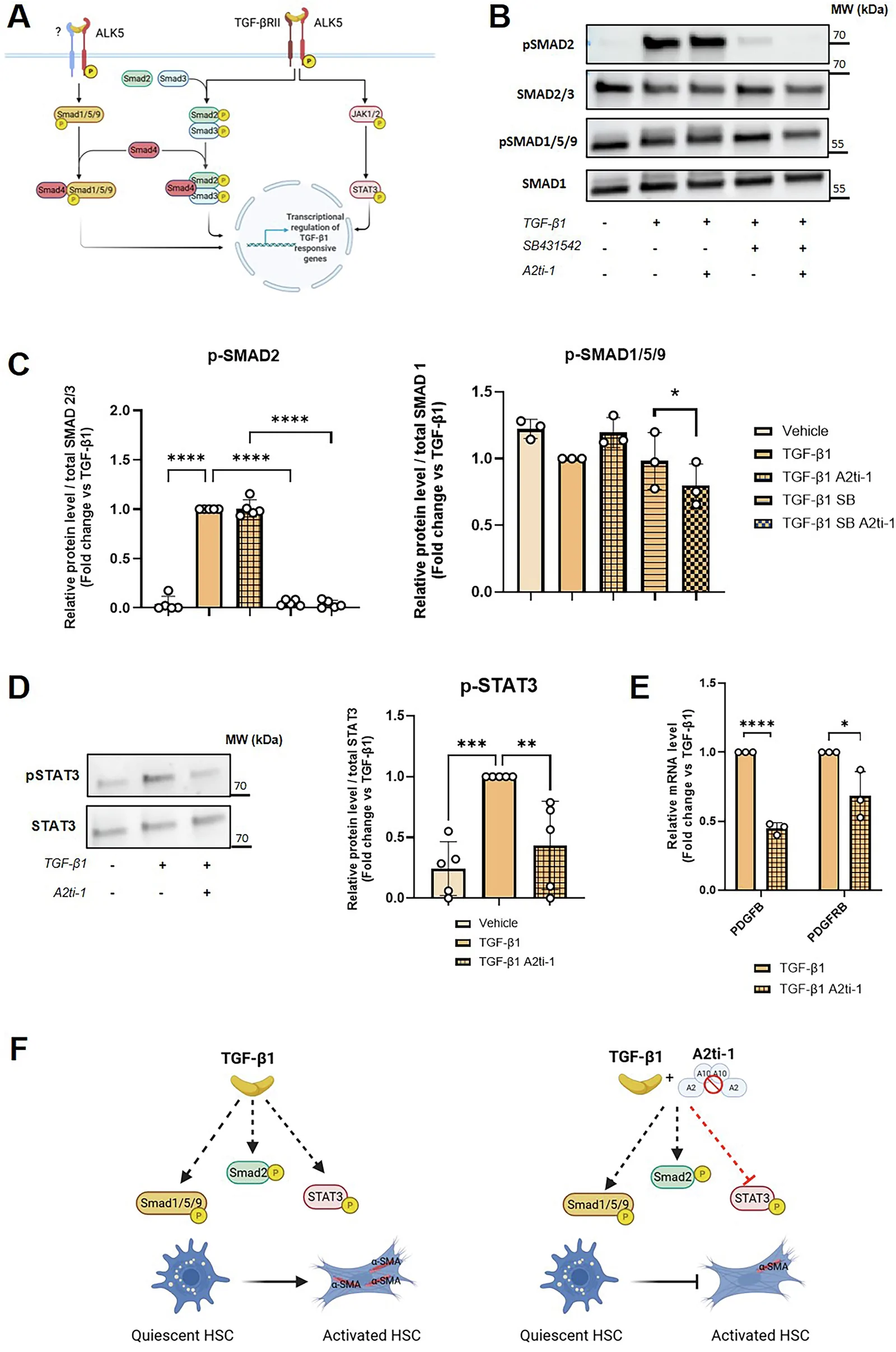
A2ti-1 suppresses TGF-β1 induced stellate cell activation through a SMAD-independent axis. (**A**) Schematic representation of TGF-β1 signalling pathways. (**B**, **C**) Western blot images (**B**) and quantification (**C**) of phosphorylated SMAD2 (pSMAD2) relative to total SMAD2/3 (*n* = 5) and phosphorylated SMAD1/5/9 (pSMAD1/5/9) relative to total SMAD1 (*n* = 3) in LX-2 cells treated with TGF-β1 (5 ng/ml) for 2 days in the presence or absence of A2ti-1 (50 μM) or SB431542 (5 μM). (**D**) Western blot images (left) and quantification (right) of phosphorylated STAT3 (pSTAT3) relative to total STAT3 in LX-2 cells treated with TGF-β1 (5 ng/ml) in the presence or absence of A2ti-1 (50 μM) for 2 days (*n* = 5). (**E**) Relative mRNA level of PDGFB and PDGFRB of TGF-β1 (5 ng/ml) activated LX-2 cells in the presence or absence of A2ti-1 (50 μM) (*n* = 3). Cyclophilin A and TBP were used as housekeeping genes. Technical triplicates have been performed for the qPCR. (**F**) Schematic illustration of signalling events downstream of TGF-β1 stimulation in LX-2 cells before and after A2ti-1 treatment. TGF-β1 induces phosphorylation of SMADs and STAT3 (dashed lines indicate simplified signalling steps). A2ti-1 does not affect SMAD phosphorylation but inhibits STAT3 activation. Created with BioRender.com. Data are presented as mean ± SD, expressed as fold change relative to the indicated control where applicable. Statistical significance was assessed using one-way ANOVA followed by Sidak’s multiple comparisons or unpaired two-tailed Student’s *t* test. ∗*P* < 0.05, ∗∗*P* < 0.01, ∗∗∗*P* < 0.001, ∗∗∗∗*P* < 0.0001, ns = not significant. “*n*” represents the number of independent experiments, performed at a different passage. Source data are available online for this figure.

Based on the observed anti-fibrotic effect of A2ti-1 on HLOs, we next investigated its potential interaction with TGF-β1 signalling specifically on stellate cells using LX-2 cell line. TGF-β1 signalling is initiated upon ligand binding to its type II receptor (TGFβR2), which recruits and phosphorylates the type I receptor (TGFβR1/ALK5), see Fig. 5A. The activated type I receptor then phosphorylates receptor-regulated SMAD2 and SMAD3. In addition, several studies have shown that TGF-β1 can also induce phosphorylation of SMAD1/5/9 through mechanisms involving the ALK5 receptor (Daly et al, 2008; Ramachandran et al, 2018). Upon phosphorylation, SMADs form complexes with SMAD4 and translocate into the nucleus to regulate transcription of TGF-β responsive genes (Weiss and Attisano, 2013). Beyond SMAD proteins, TGF-β1 also activates several non-canonical signalling cascades. Among these, the JAK/STAT3 signalling pathway plays a critical role in cell proliferation, myofibroblast differentiation, ECM production and α-SMA expression (Deng et al, 2024; Finnson et al, 2020) (Fig. 5A).

**Figure EV4:**
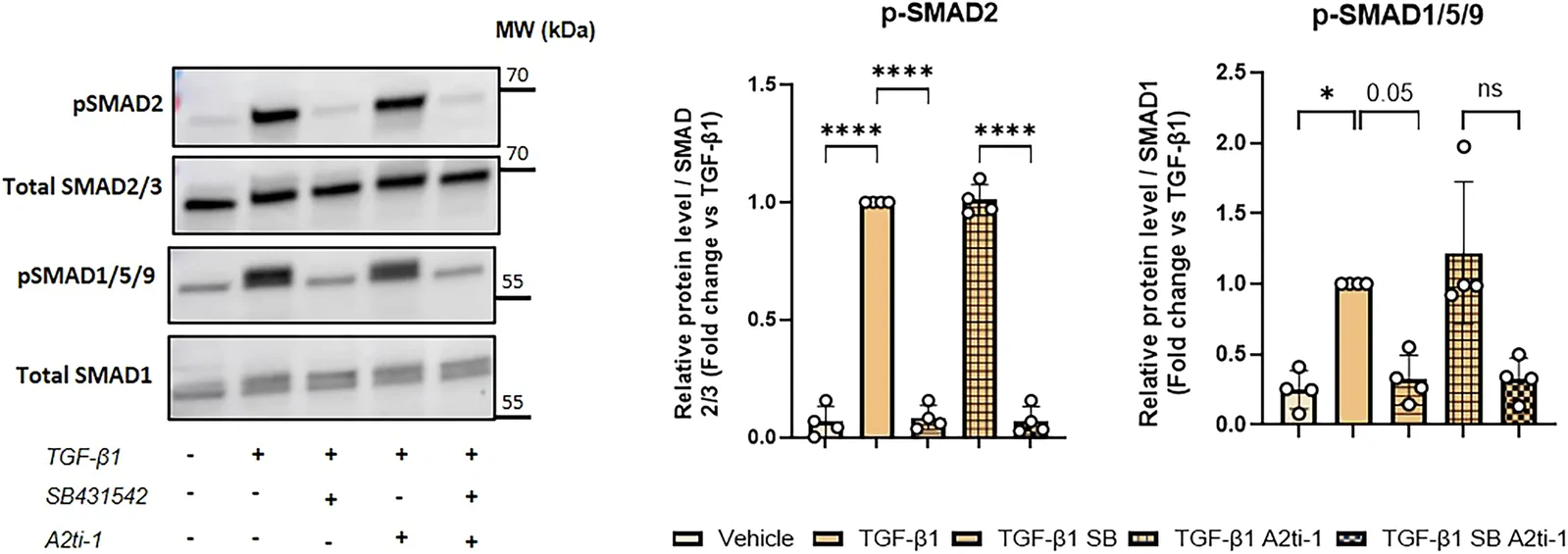
Phosphorylation of SMAD proteins after 1 h TGF-β1 stimulation. Western blot images (left) and quantification (right) of phosphorylated SMAD2 (pSMAD2) relative to total SMAD2/3 and phosphorylated SMAD1/5/9 (pSMAD1/5/9) relative to total SMAD1 in LX-2 cells treated with TGF-β1 (5 ng/ml) for 1 h in the presence or absence of A2ti-1 (50 μM) or SB431542 (5 μM). Data are presented as mean ± SD, expressed as fold change relative to the vehicle. Statistical significance was assessed one-way ANOVA followed by Sidak’s multiple comparisons test, or Kruskal–Wallis followed by Dunn’s multiple comparisons test when data did not pass normality assumptions. ∗*P* < 0.05, ∗∗*P* < 0.01, ∗∗∗∗*P *< 0.0001, ns = not significant (*n* = 4 for each group, “*n*” represents the number of independent experiments, performed at a different passage).

We first investigated the effects of A2ti-1 on SMAD signalling. Western blot analysis showed that 48 h of TGF-β1 treatment significantly increased pSMAD2, an effect completely blocked by the ALK5 inhibitor SB431542 (Fig. 5B,C). In contrast, A2ti-1 did not alter pSMAD2 levels (Fig. 5B,C). We next assessed phosphorylation of SMAD1/5/9 under the same conditions. However, 48 h of TGF-β1 exposure did not significantly change its phosphorylation status, nor did SB431542 modify this parameter (Fig. 5B,C). A2ti-1 slightly but significantly reduced its phosphorylation. Because SMAD1/5/9 activation can be transient (Daly et al, 2008; Ramachandran et al, 2018), we evaluated shorter TGF-β1 stimulation. After 1 h of TGF-β1 treatment, phosphorylation state of SMAD2 but then also of SMAD1/5/9 was markedly increased and effectively suppressed by SB431542, confirming ALK5 signalling dependency. Consistent with the 48 h exposure, A2ti-1 did not prevent SMAD2 nor SMAD1/5/9 phosphorylation induced by 1 h of TGF-β1 treatment (Fig. EV4).

Given the lack of effect of A2ti-1 on SMAD signalling, we next examined non-canonical TGF-β1 pathways. Previous studies have reported an association between ANXA2 and STAT3 (Rocha et al, 2018; Wang et al, 2015); therefore, we focused on the TGF-β-STAT3 axis. Treatment with TGF-β1 for 48 h markedly increased STAT3 phosphorylation, which was significantly reduced by A2ti-1 (Fig. 5D). To further support our findings regarding the effect of A2ti-1 on non-canonical TGF-β1 signaling, we analyzed downstream transcriptional target of STAT3. Platelet-derived growth factor subunit B (PDGFB) was shown to be reduced following STAT3 depletion in TGF-β1 stimulated LX-2 cells (Tang et al, 2017). In our study, A2ti-1 treatment significantly decreased PDGFB mRNA levels in activated LX-2 cells. Additionally, we observed a reduction in the expression of its receptor PDGFRB under the same conditions (Fig. 5E).

Collectively, these findings suggest that A2ti-1 suppresses TGF-β1 induced profibrotic responses in hepatic stellate cells by modulating at least the TGF-β1-STAT3 axis, independently of SMAD signalling (Fig. 5F).

## Discussion

The incidence and prevalence of MASLD have increased dramatically in recent decades. Yet, only a small fraction of preclinical findings ever progressed successfully into early-phase clinical trials (Miao et al, 2024; Wu et al, 2025). Effective therapies targeting MASLD, particularly its fibrotic stage, remain limited due to the complex and multifactorial nature of the disease and the lack of physiologically relevant preclinical human in vitro models. In this regard, organoid technology has emerged as a powerful platform for liver fibrosis modelling (Hess et al, 2023; Hurrell et al, 2020) and anti-fibrotic compound screening (Hurrell et al, 2020; Wu et al, 2023), which positions human liver organoids (HLOs) as a valuable system for preclinical investigations. We therefore employed a combined approach, using HLOs to assess the role of the S100A10-ANXA2 tetramer (A2t) in liver fibrosis and LX-2 human stellate cells to dissect the underlying molecular mechanisms and potential of the A2t inhibitor, A2ti-1.

Our study shows that pharmacologic disruption of A2t tetramer using A2ti-1 reduces TGF-β1 induced hepatic stellate cell (HSC) activation. Consistently, genetic downregulation of S100A10 in LX-2 cells partially reproduced the anti-fibrotic effects obtained with A2ti-1, pointing to the key role played by S100A10 in HSC activation. Using human embryonic stem cell derived multilineage HLOs to model progressive stages of MASLD/MASH, we further demonstrated that A2t inhibition selectively mitigates fibrosis without altering lipid accumulation or inflammatory cytokine IL-6 release. Mechanistically, these effects occur without altering canonical TGF-β-SMAD signalling, while being associated with suppression of the STAT3 phosphorylation. Together, our findings underscore the functional relevance of A2t tetramer in MASLD/MASH development and identify A2t disruption as a promising anti-fibrotic strategy.

S100A10 primarily functions as part of a heterotetramer complex composed of two Annexin A2 (ANXA2) and two S100A10 (Okura et al, 2023). Hepatocyte-derived ANXA2 itself has been implicated in liver fibrosis (Zhang et al, 2010), promoting HSC activation through indirect mechanisms, including the participation of von Willebrand factor (vWF) (Yang et al, 2017), and osteopontin secretion by hepatocytes (Wang et al, 2022). Altogether, these observations raise the possibility that S100A10’s profibrotic function may rely on its interaction with ANXA2 within the heterotetramer complex. We previously uncovered S100A10 as an inducer of MASLD progression where AAV-mediated hepatic silencing of S100A10 significantly reduced steatosis in both diet-induced and genetic mouse models (Delangre et al, 2025). However, its molecular partner required for fibrosis development remains to be identified. In the present study, disruption of S100A10-ANXA2 tetramer in MASLD modelling HLOs by A2ti-1 resumed fibrosis without affecting lipid accumulation. These findings suggest that previously attributed role for S100A10 in promoting steatosis (Delangre et al, 2025) may be independent of its tetramerization with ANXA2. In addition, we previously observed that hepatic S100A10 knockdown also reduced fibrosis in a diet-based MASH model (Delangre et al, 2025). It remains unclear whether the antifibrotic effect of S100A10 silencing resulted indirectly from reduced steatosis or reflected a direct effect on HSCs. Here, we observed that A2ti-1 directly attenuated activation of LX-2 cells, indicating that S100A10 may contribute to MASLD pathogenesis through two distinct mechanisms: promoting steatosis in hepatocytes, while cooperating with ANXA2 to drive fibrogenesis in HSC. Further mechanistic studies should investigate this putative dual function.

Noteworthy, we observed a difference in the A2ti-1 mediated repression of fibrosis-related genes under either TGF-β1 or OA/PA TGF-β1 treatments, the latter being less responsive to A2ti-1. In the absence of A2ti-1, baseline transcriptional state of fibrosis-related genes showed a partial fibrotic transcriptional response under combined OA/PA TGF-β1 treatment compared to TGF-β1 alone that induced a fully activated program of extracellular matrix production. Thus, the apparent discrepancy reflects distinct baseline fibrotic states. These observations are consistent with recent single-cell transcriptomic analyses of HLOs reported by Hess et al (Hess et al, 2023) which showed that OA (500 μM) treatment alone reduces COL1A1 expression and collagen deposition, whereas PA (500 μM) primarily induces inflammatory rather than fibrotic transcriptional programs. Hess et al showed that OA exerts anti-fibrotic effects even in the presence of metabolic stress, aligning with evidence showing that OA-rich lipid environments attenuate fibrosis progression (Frendi et al, 2025; Hong et al, 2018). In our experimental design, the OA/PA mix was applied at a ratio (400 µM OA/200 µM PA) that likely preserved the anti-fibrotic influence of unsaturated OA while limiting PA-driven fibrotic gene induction. This might explain why OA/PA TGF-β1 selectively induces α-SMA/ACTA2, reflecting cytoskeletal activation, without fully engaging a collagen-producing transcriptional program. Under these conditions, A2ti-1 can still reduce α-SMA protein levels, while exerting limited effects on collagen gene expression.

TGF-β1 is a central driver of HSC activation, acting through both canonical SMAD-dependent and non-canonical pathways (Dewidar et al, 2019). Here, A2ti-1 does not alter SMAD phosphorylation, indicating that its antifibrotic activity is not mediated by the canonical TGF-β-SMAD axis. This suggests that the S100A10-ANXA2 tetramer contributes to HSC activation through SMAD-independent signalling mechanisms. Beyond the SMAD cascade, TGF-β1 can also trigger JNK, p38 MAPK, NF-κB, PI3K/AKT, and JAK/STAT signalling, orchestrating diverse cellular responses, including survival, proliferation, metabolism, and differentiation (Deng et al, 2024; Ramachandran et al, 2018). Among non-canonical TGF-β pathways, JAK/STAT signalling is of particular interest. Indeed, STAT3 activation promotes HSC proliferation, myofibroblast differentiation, collagen deposition, and liver fibrosis both in vitro and in vivo (Chakraborty et al, 2017; Levy and Darnell, 2002; Liu et al, 2013; Meng et al, 2012; Neeli et al, 2004; Tang et al, 2017). Furthermore, ANXA2 has been previously linked to STAT3 activation in several conditions. ANXA2 directly interacts with STAT3, thereby enhancing its transcriptional activity, particularly in epithelial-mesenchymal transition (EMT) (Wang et al, 2015). In breast cancer cells, phosphorylated ANXA2 induces STAT3 phosphorylation and transcriptional activation promoting EMT (Wang et al, 2015). Similarly, studies on rectal cancer cells have demonstrated that TGF-β1 induces ANXA2 phosphorylation that, through engagement of the Src/ANXA2/STAT3 signalling axis, favours TGF-β1 driven EMT (Rocha et al, 2018). In MASH, STAT3 functions as a transcription factor for ANXA2, binding to its promoter and upregulating its expression (Feng et al, 2022). These studies point to a context-dependent regulatory loop between ANXA2 and STAT3. In addition, a recent study demonstrated that administration of A2ti-1 in a mouse model of acute kidney injury significantly attenuates renal damages by reducing STAT3 phosphorylation (Chen et al, 2025). In agreement with these reports, our data demonstrate that A2ti-1 treatment markedly reduces STAT3 phosphorylation in activated LX-2 cells, supporting the hypothesis that its antifibrotic effects are mediated, at least partially, by the suppression of STAT3 signalling. Previous work reported that PDGFB expression is suppressed by downregulation of STAT3 in TGF-β1 activated LX-2 cells (Tang et al, 2017). Here, we showed that both PDGFB and its receptor PDGFRB are significantly downregulated in LX-2 cells after A2ti-1 treatment, further supporting the role of A2ti-1 in modulating the STAT3 signalling pathway. Importantly, PDGFB/PDGFRB axis is not only a target of STAT3 but also a potent upstream activator of STAT3 signalling (Kim et al, 2012; Neeli et al, 2004; Tang et al, 2017). Accordingly, A2ti-1 treatment reduces the expression of both PDGFB and PDGFRB. This dual action indicates that A2ti-1 may exert antifibrotic effects through two complementary mechanisms. First, it could directly suppress TGF-β1 induced α-SMA expression and collagen production. Second, it could disrupt the PDGFB/PDGFRB-STAT3 positive feedback loop, reducing both autocrine and paracrine amplification of STAT3 signalling. Further studies are needed to determine whether A2t directly affects STAT3 activation or modulates upstream kinases within this pathway. Together, these mechanistic insights suggest that the S100A10-ANXA2 tetramer is not only a structural component of HSC activation but also a dynamic signalling node.

Of note, the HLOs produced in this study lack resident immune cells. IL-6 production in this system likely reflects stress responses by non-immune cells. In this context, A2ti-1 selectively suppresses fibrogenic HSC activation without broadly attenuating stress-associated cytokine production IL-6. Here, we observed that A2ti-1 hampered STAT3 phosphorylation without affecting the canonical TGF-β1 SMAD-dependent pathway. Our hypothesis is that IL-6 secretion would be more dependent on SMAD pathway rather than STAT3, arguing for an A2t independent effect. Once secreted, IL-6, initiates signaling pathways relying on JAK/STAT (Kishimoto, 2005). Therefore, even if the amount of secreted IL-6 is unchanged upon A2ti-1 treatment, downstream deleterious signaling should be reduced.

Incidentally, our study draws attention to the therapeutic potential of A2ti-1 in liver fibrosis, although further in vivo investigations should be conducted. Notably, A2ti-1 not only exerts preventative effects but also revers established fibrosis in TGF-β1 treated HLOs. Pharmacological inhibition of STAT3 is an emerging therapeutic strategy, with several STAT3 targeting agents in ongoing or completed early-phase clinical trials, including TTI-101 (solid tumours) and danvatirsen (hematologic malignancies) as well as feasibility study testing STA-21 in psoriasis (Chakraborty et al, 2017; Li et al, 2015; Miyoshi et al, 2011; Reilley et al, 2018; Tsimberidou et al, 2025). In this context, selective disruption of the S100A10-ANXA2 tetramer may represent an alternative strategy of dampening STAT3-driven fibrogenic signalling and A2ti-1 as a promising candidate for MASLD/MASH pharmacology. Importantly, inhibition of the STAT3 signalling axis, without affecting the canonical SMAD-dependent pathway, would preserve physiological functions of TGF-β reducing potential side effects.

The antifibrotic effects of A2ti-1 observed in HLOs strengthen the translational relevance of targeting the S100A10-ANXA2 tetramer in a human multicellular context. While HLOs offer advantages over traditional 2D and animal models (Delire et al, 2015; Kaur et al, 2023), current systems remain developmentally immature and lack immune and vascular compartments, which limits the modelling of inflammatory and stromal interactions in advanced MASLD (Andrews and Kriegstein, 2022; Osonoi and Takebe, 2024). Future incorporation of these components will enhance the utility of HLOs for fibrosis research and therapeutic testing.

Overall, our study uncovers the role for the S100A10-Annexin A2 tetramer in hepatic fibrosis. Targeting this tetramer to inactivate hepatic stellate cells could offer a compelling strategy to combat liver fibrosis. Nevertheless, further studies should assess the pharmacokinetics, specificity, and safety profile of A2ti-1 and evaluate its therapeutic efficacy in vivo.

## Methods

**Table:** Reagents and tools table

Reagent/resource	Reference or source	Identifier or catalog number
**Experimental models**		
LX-2 cells	Gift from Francesco Negro laboratory	N/A
HS-420 cells, BAG-hES-IMP-0046	Gift from Karl-Heinz Krause laboratory (El Harane et al, 2025). Originally from Karolinska Institute, Stockholm, Sweden	N/A
**Antibodies**		
S100A10	Abcam	ab76472
Annexin A2	Cell Signalling	8235S
pSTAT3	Cell Signalling	9145
STAT3	Santa Cruz	sc482
pSMAD2	GeneTex	GTX133614
SMAD2/3	GeneTex	GTX111123
pSMAD1/5/9	Cell Signalling	13820
SMAD1	Cell Signalling	9743
ADRP	Progen	610102
ERM	Cell Signalling	3142
Caspase 3	Cell Signalling	14220
Cleaved-caspase 3	Cell Signalling	9664
Tubulin	Cell Signalling	2128
α-SMA	Gift from Marie-Luce Bochaton Piallat laboratory	
HNF4-Alexa647	Abcam	Ab217073
PDGFRβ	Abcam	Ab32570
Glutamine Synthetase	Merck	MAB302
HNF4	Santa Cruz	Sc-8987
**Oligonucleotides and other sequence-based reagents**		
Primers	Microsynth	Appendix Table S1
siRNA Control	Qiagen	1027310
siRNA anti-human S100A10	Qiagen	SI03246670
**Software**		
QuantStudio 5 Real-Time PCR System	Applied Biosystems	N/A
Fusion Evolution-Capt Edge software.	Vilber	N/A
MATLAB R2024a	Correia de Sousa et al, 2024	N/A
QPath version 0.5.1 and 0.6.0	Open source	N/A
Cellpose 3.0	Open source	
Cell Ranger version 7.2.0	10x Genomics	N/A
BD FACSDiva software (version 8.0.2)	BD Biosciences	N/A
**Other**		
Accutase	Gibco	A1110501
StemFlex medium	Gibco	A3349401
Penicillin-Streptomycin	Gibco	15140122
Rock inhibitor	Tocris	Y27632
RPMI-1640 medium	Gibco	61870010
BMP4	R&D Biotechne	314-BP-010
Activin A	R&D Biotechne	338-AC-050
KSR	Gibco	A3181502
DMEM/F12 medium	Gibco	12634010
GlutaMax	Gibco	35050038
B27	Gibco	17504044
N2	Gibco	17502048
Gentamycin/Amphotericin	Gibco	R01510
FGF4	PeproTech	100-31
CHIR99021	Miltenyi	130-106-539
Matrigel	Corning	356237
Retinoic acid	Sigma	R2625
HCM	Lonza	CC-3198
HGF	PeproTech	100-39H
Dexamethasone	Sigma	D4902
Oncostatin M	PeproTech	300-10
TGF-β1	PeproTech	100-21
A2ti-1	MCE	HY-136465
DMEM 4.5 g/l glucose	Gibco	61965-026
SB431542	Sigma	S4317
HiPerFect Transfection Reagent kit	Qiagen	301704
TRIzol	Invitrogen	15596026
High-Capacity cDNA Reverse Transcription Kit	Applied Biosystems	4368814
PowerUp™ SYBR™ Green Master Mix	Applied Biosystems	A25742
BCA protein assay kit	Pierce Biotechnology	23225
Nitrocellulose membranes	Amersham	RPN303D
ECL Prime Substrate	Amersham	RPN22232
Glycerol Lysis Solution	Promega	J3160
Kit Triglycerides FS	DiaSys	157109910021
BODIPY	Molecular probes	D3922
Hoechst	ThermoFisher Scientific	33342
DAB kit	Abcam	ab64238
IL-6 ELISA kit	R&D System	D6050B
Urea Assay Kit	BioAssay Systems	DIUR-100
TrypLE Express	Gibco	12604013
70 μm MACS SmartStrainer	Militenyi	130-110-916
Qubit fluorometer	ThermoFisher Scientific	
Tapestation (DNA High sensitivity chip)	Agilent Technologies	

### Methods and protocols

#### Human liver organoids (HLO)

##### Culture, differentiation and maturation

Human embryonic stem cells (hESC) (HS420, BAG-hES-IMP-0046, Karolinska Institute, Stockholm, Sweden) were differentiated into HLOs using a well-established method with slight modifications (Correia de Sousa et al, 2024; Thompson and Takebe, 2020). When reached 70–80% confluency, hESCs colonies were detached by Accutase (Gibco, A1110501) and the cell pellet was resuspended in StemFlex medium (Gibco, A3349401) supplemented with 1% penicillin-streptomycin (PS) (Gibco, 15140122) and 10 µM of Rock inhibitor (Tocris, Y27632). Approximately 100 cells per well were seeded in air/liquid interface microwells (Airliwells) (El Harane et al, 2025). The next day, Rock inhibitor was removed from the medium. In order to induce definitive endoderm development, medium was changed to RPMI-1640 (Gibco, 61870010) supplemented with PS, 50 ng/mL bone morphogenetic protein 4 (BMP4, R&D Biotechne, 314-BP-010) and 100 ng/mL activin A (R&D Biotechne, 338-AC-050). 100 ng/mL of activin A (R&D Biotechne, 338-AC-050) and 0.2% KnockOut Serum Replacement (KSR, A3181502, Gibco) were added to RPMI-1640 on day 2, and 100 ng/mL of activin A (338-AC-050, R&D Biotechne) and 2% KSR (Gibco, A3181502) were added on day 3. The medium was switched to advanced DMEM/F12 (Gibco, 12634010) from day 4 to day 6, supplemented with 0.1 mM Hepes, GlutaMax (Gibco, 35050038), 1× B27 (Gibco, 17504044), 1× N2 (Gibco, 17502048), 1x Gentamycin/Amphotericin (Gibco, R01510), 500 ng/mL fibroblast growth factor 4 (FGF4, PeproTech, 100-31), 3 µM CHIR99021 (Miltenyi, 130-106-539), and changed every day. On day 7, 20% Matrigel (Corning, 356237) and 2 µM retinoic acid (RA, Sigma, R2625) were added to the advanced DMEM/F12 supplemented with B27 and N2, changed every other day. After total of 4 days of RA treatment, the media was switched to Hepatocyte Culture media (HCM, Lonza, CC-3198) supplemented with 10 ng/mL Hepatocyte Growth Factor (HGF, PeproTech, 100-39H), 100 nM Dexamethasone (Sigma, D4902), 20 ng/mL Oncostatin M (Peprotech, 300-10) (complete HCM medium), and 20% Matrigel. The medium was changed every 3 days. After being carefully removed from Airliwells on day 17, HLOs were maintained in ultralow attachment plates suspended in complete HCM medium with 10% Matrigel until the end of the experiments (day 21, 26, or 29).

##### Steatosis, inflammation and fibrosis induction in HLOs

At the end of the HLO differentiation (D21), disease modeling was initiated using defined treatments. To model steatosis, HLOs were treated for 5 days with either 400 μM or 600 μM of oleic acid/palmitic acid (OA/PA) mixture in a 2:1 ratio. Oleate and palmitate were complexed to BSA (Thompson and Takebe, 2020).

For fibrosis modeling, HLOs were treated with 20 ng/mL of TGF-β1 recombinant protein (Peprotech, 100-21). To induce a steatohepatitis phenotype, HLOs were co-treated with 600 μM OA/PA and 20 ng/mL TGF-β1. All treatments were carried out for 5 days, with medium refreshed after 2 days. To investigate the effect of A2ti-1 in MASLD/MASH modeling HLOs, HLOs were treated with either 50 µM A2ti-1 (MCE, HY-136465) or 0.5% DMSO-control concurrently with the above-described stimulants for the indicated time period. The concentration of A2ti-1 used in this study was selected based on published data indicating effective biological activity without reported cytotoxicity in cultured cells at concentrations up to 100 μM (Weng et al, 2023; Woodham et al, 2015). For therapeutic modeling, fibrosis was first induced in HLOs by 5 days of TGF-β1 (20 ng/mL) treatment, then 50 µM A2ti-1 were added to the medium for an additional 3 days. At the end of each experiment, conditioned media were collected and sequentially centrifuged at 300×*g* for 5 min and 2000×*g* for 5 min, and the supernatants were stored at −80 °C for subsequent analyses. HLOs were washed twice with DPBS (−/−) and stored at −80 °C for protein and mRNA analyses or fixed in 4% paraformaldehyde.

#### Culture and treatments of human hepatic stellate cell line LX-2

##### Culture

LX-2 cell line (a kind gift from Prof. Francesco Negro, University of Geneva) was cultured in 4.5 g/l glucose Dulbecco’s modified Eagle’s medium (DMEM) without pyruvate (Gibco, 61965-026), supplemented with 2% fetal bovine serum (FBS) and 1% PS. For all the experiments, 200,000 cells were seeded in six-well plates. Cells were used between passages 10 and 30 following routine anti-mycoplasma treatment.

##### Pharmacological treatments

To test the effect of A2ti-1 on TGF-β1-SMADs and TGF-β1-STAT3 pathways, LX-2 cells exposed to TGF-β1 (5 ng/ml) and treated with TGFβR1 inhibitor SB431542 (5 µM, Sigma-Aldrich, S4317) and/or A2ti-1 (12.5, 25 or 50 µM) or 0.5% DMSO (vehicle) for 2 days. To investigate the phosphorylation status of SMADs, LX-2 cells were serum-deprived for 48 h in DMEM 4.5 g/l glucose + 1% P/S, followed by a 1 h pre-treatment with SB431542 (5 µM, Sigma-Aldrich, S4317) and/or A2ti-1 50 µM or 0.5% DMSO (vehicle). Subsequently, LX-2 cells were activated with 5 ng/mL TGF-β1 for 1 h. Following A2ti-1 treatment, nuclei were stained with DAPI and automatically detected on ImageXpress system.

##### Transfection

For downregulation of S100A10, LX-2 cells were transfected with siRNA during 72 h (20 nmol of siRNA using the HiPerFect Transfection Reagent kit (Qiagen, 301704). Control siRNA (AllStars Neg.Control siRNA, Qiagen, 1027310) and anti-human S100A10 siRNA (Qiagen, SI03246670) were used.

#### RNA extraction and RT-qPCR

Total RNA from LX-2 cells and HLOs was extracted using TRIzol (Invitrogen, 15596026) following the standard TRIzol-chloroform-isopropanol extraction protocol. RNA concentrations were measured using NanoDrop spectrophotometer (Thermo Scientific). Complementary DNA (cDNA) was synthesized from 0.5 or 1 µg total RNA using the High-Capacity cDNA Reverse Transcription Kit (Applied Biosystems™, 4368814) according to the supplier’s instructions. Quantitative real-time PCR (qPCR) was carried out using PowerUp™ SYBR™ Green Master Mix (Applied Biosystems™, A25742) on a QuantStudio 5 Real-Time PCR System with the associated data analysis software. Primer sequences are provided below. Relative gene expression was quantified using the 2^−ΔΔCt method. Primers used are listed in Appendix Table S1.

#### Western blot

Total proteins from LX-2 cells and HLOs were extracted using RIPA buffer (50 mM Tris-HCl, pH 6.8, 100 mM DTT, 2%SDS, 0.1% bromophenol blue, 10% glycerol). Lysates were centrifuged at 12,000×*g* for 10 min, and the supernatants were collected. Protein concentration was measured using BCA protein assay kit (Pierce Biotechnology, 23225). The extracted proteins (5–10 µg) were separated by 5-20% gradient sodium dodecyl sulfate-polyacrylamide gel electrophoresis (SDS-PAGE) and transferred onto nitrocellulose membranes (Amersham, RPN303D). The membranes were blocked with 5% non-fat dry milk in TBS-T (0.1% Tween-20) and incubated overnight at 4 °C with the S100A10 (1/2000, Abcam, ab76472), AnnexinA2 (1/2000, Cell Signalling, 8235S), pSTAT3 (1/1000, Cell Signalling, 9145), STAT3 (1/1000, Santa Cruz, sc482), pSMAD2 (1/1000, GeneTex, GTX133614), SMAD2/3 (1/10000, GeneTex, GTX111123), pSMAD1/5/9 (1/1000, Cell Signaling, 13820), SMAD1 (1/1000, Cell Signaling, 9743), ADRP (1/1000, Progen, 610102), ERM ((Ezrin/Radixin/Moesin), 1/1000, Cell Signaling, 3142), Pro-caspase 3 (1/1000, Cell Signaling, 14220), Cleaved-caspase 3 (1/1000, Cell Signaling, 9664), Tubulin (1/5000, Cell Signaling, 2128) and α-SMA (kind gift from Prof. Marie-Luce Bochaton-Piallat, University of Geneva). After washing with 0.1% TBS-T, membranes were incubated with corresponding secondary antibodies for 1 h at room temperature. Protein bands were visualized using ECL Prime Substrate (Amersham, RPN22232) and detected with the FUSION imaging system (Vilber, France). Signal intensities were quantified using Fusion Evolution-Capt Edge software.

#### Triglyceride quantification

HLOs were lysed in Glycerol Lysis Solution (Promega, J3160) using sonication. Triglyceride concentrations were quantified by a colorimetric assay (Kit Triglycerides FS, DiaSys, 157109910021), and normalized to total protein content.

#### 3D immunofluorescence and Bodipy staining of lipid droplets

##### Staining

HLOs were fixed with 4% paraformaldehyde (PFA) at room temperature for 2 h. Following fixation, samples were incubated overnight at 4 °C with HNF4-Alexa647 antibody (1:100, Abcam, ab217073) in PBS containing 0.5% Triton X-100 and 1% BSA as blocking buffer. The next day, HLOs were washed three times with PBS, and stained for neutral lipids using BODIPY (1 µg/mL, Molecular Probes, D3922), counterstained with Hoechst (1 µg/mL, ThermoFisher Scientific, 33342) for 15 minutes. Imaging was performed using a Nikon A1R Spectral confocal microscope (Nikon).

##### Analysis and quantification

For HLO image analysis, 3D confocal stacks were processed using a dedicated framework developed in MATLAB R2024a (The MathWorks) (Correia de Sousa et al, 2024), Nikon. nd2 files were imported via the Bio-Formats package (Linkert et al, 2010). Organoid reconstruction was performed by computing the voxel-wise maximum across all available normalized channels. A 3D Gaussian filter was then applied, followed by global image segmentation using Otsu’s method. Potential holes in the resulting binary images were subsequently filled. Nuclei (labeled with Hoechst and/or HNF4) and lipid droplets were restored (denoised or deblurred) and segmented using Cellpose 3.0 (Stringer and Pachitariu, 2025; Stringer et al, 2021) with its pre-trained “nuclei” and “cyto3” models, respectively. Lipid droplets were assigned to specific cell nuclei based on the shortest centroid-to-centroid Euclidean distance. Finally, results were quantified in terms of total lipid droplet volume and total lipid droplet number in HNF4-positive nuclei. Custom code used for semi-automatic quantification analyses is available from the corresponding author upon request.

#### Histology and immunostainings of slides

##### Embedding

After 2 h of fixation in 4% PFA, HLOs were embedded in 3% bactoagar prior to paraffin embedding. HLOs were sectioned at 5μm thickness using a microtome.

##### Colorations

Periodic Acid-Schiff (PAS) and Masson’s Trichrome coloration were performed by the Histology Core Facility of the University of Geneva.

##### Immunofluorescence

Paraffin sections were deparaffinized in Histoclear and rehydrated through a graded ethanol series (100%, 100%, 95%, 70%) followed by distilled water. Antigen retrieval was performed in citrate buffer (pH 6.0) for 30 min. Sections were then blocked and permeabilized in PBS containing 10% normal goat serum (NGS), 1% BSA, and 0.2% Triton X-100 for 1 h at room temperature, followed by overnight incubation at 4 °C with primary antibodies: HNF4-Alexa647 antibody (1:100, Abcam, ab217073), PDGFRβ antibody (1:100, abcam, ab32570), Glutamine Synthetase antibody (GS-6) (1:250, Merck, MAB302), anti-αSMA in 1% BSA in PBS. The next day, after washing steps, slides were incubated with appropriate secondary antibody at room temperature for 1 h Following immunofluorescence labelling, slides were mounted and imaged using either an AxioScan Z.1 slide scanner (Zeiss) or a Stellaris confocal microscope (Leica).

##### Immunohistochemistry

Following deparaffinization, hydration and antigen retrieval described in the “immunofluorescence” section, samples were incubated in 3% H_2_O_2_ for 15 min. Blocking was performed in 10% normal goat serum, followed by overnight incubation at 4 °C with primary antibody anti-HNF4 (1:100, Santa Cruz, sc-8987). After appropriate HRP-conjugated secondary antibody incubation, DAB-based revelation was performed (Abcam, ab64238).

##### Semi-automatic quantification using QuPath

Whole-slide images were analyzed using QuPath (version 0.5.1 or version 0.6.0 [https://doi.org/10.1038/s41598-017-17204-5]) through various semi-automated tools. Manual curation was subsequently conducted to validate and refine the automated detections. For the quantification of fibrosis, Masson’s trichrome-positive regions were identified using pixel-based thresholding, and the results were expressed as the proportion of Masson-positive area relative to the total HLO area.

Custom code used for semi-automatic quantification analyses is available from the corresponding author upon request.

#### IL-6 ELISA

At the end of each treatment, conditioned medium of HLOs was collected and sequentially centrifuged at 300×*g* for 5 min and 2000×*g* for 10 min at 4 °C to remove cellular debris. Interleukin-6 (IL-6) levels were quantified using an ELISA kit (R&D System, D6050B) according to the manufacturer’s instructions and normalized to total protein content.

#### Measurement of urea production

At the end of the HLO differentiation protocol (D21), medium of HLOs was collected and sequentially centrifuged at 300×*g* for 5 min and 2000×*g* for 10 min at 4 °C. To measure the urea concentration, Urea Assay Kit (BioAssay Systems, DIUR-100) was used according to the manufacturer’s instructions and normalized to total protein content and day of culture. Media collected from Huh-7 cells were used as positive controls.

#### Single-cell RNA sequencing

##### Sample preparation and sequencing

On D21, HLOs from three wells of an ultra-low attachment 24-well plate were collected and washed 2× with DPBS (−/−). The HLOs were then dissociated using TrypLE Express (Gibco, 12604013) for 15 min at 37 °C. Following dissociation, the cell suspension was passed through a 70 μm MACS SmartStrainer (Miltenyi, 130-110-916) to obtain a single-cell suspension. Cells were collected in 1% BSA in DMEM, centrifuged at 300×*g* for 5 minutes, and resuspended again in 1% BSA in DMEM. Viable cells were gated with flow cytometry (BD FACSDiva software (version 8.0.2)) using double staining. Dead cells were eliminated with Draq7, while alive cells were sorted with Hoechst. Cell counting was performed using a Tali™ Image-Based Cytometer. Subsequently, cells were sent to iGE3 Genomics Platform of University of Geneva for processing.

The 10× Genomics Chromium equipment and the 3′v3.1 reagent kit were used to create single-cell RNA-Seq libraries in accordance with the manufacturer’s instructions. A Qubit fluorometer (ThermoFisher Scientific) and a Tapestation (DNA High sensitivity chip - Agilent Technologies) were used for library quantification and quality evaluation. Libraries were sequenced on an Illumina NovaSeq 6000 for paired-end 28–90 reads.

##### Analysis

Single-cell RNA-seq data were processed using Cell Ranger version 7.2.0 with human genome GRCh38-2020-A. Filtered gene quantification matrix were processed with Seurat package in R, filtering out genes expressed in fewer than 3 cells and cells with fewer than 200 detected genes. During the quality control, cells with less than 40,000 UMIs or less than 20% mitochondrial transcripts were included. Doublets were identified and removed using scDblFinder. PCA and UMAP dimensionality reduction was performed using 500 most variable genes, and cells clusters were identified using first 10 principal components at a resolution of 0.5. Cell type annotation was performed using a pre-trained classifier derived from the annotated day 21 HLO dataset (Data ref: GEO GSE207889, 2023), as described by Hess et al (Hess et al, 2023). Predicted cell types were assigned based on the highest classification score, and clusters were relabeled when the predicted identity of cells within the cluster exceeded 85%. Then markers of each cluster were identified with FindAllMarkers () method of Seurat package (adjusted *P* value ≤ 0.05, log₂FC > 0.5, and expression in ≥5% of cells in either group).

Human gene symbols were further mapped to Ensembl IDs (release 110) using a table generated using Biomart service from Ensemble.org Pathway enrichment analysis was then conducted against Reactome and Gene Ontology databases for each gene set using hypergeometric tests. Only pathways with adjusted *P* value ≤ 0.05 and at least 10 overlapping genes were considered significant.

To benchmark hepatocyte maturation in the present study, we compared the expression of representative immature/hepatoblast and mature hepatocyte markers with Data ref: GEO GSE207889, 2023 (Hess et al, 2023), of day 21 HLOs. The reference dataset was analyzed independently using the processed, log-normalized expression values provided by the original study. A curated set of immature/hepatoblast markers (AFP, DLK1, GPC3, IGF2, KRT19, and SOX9) and mature hepatocyte markers (ALB, APOA1, APOB, CEBPA, CYP2C9, CYP3A4, HNF4A, SERPINA1, SULT2A1, and TTR) was used to assess hepatocyte differentiation status. For each dataset, mean expression levels and the fraction of cells with detectable expression (expression > 0) were calculated for hepatocyte-like clusters. Cholangiocyte-like clusters were included as a control population to confirm lineage specificity of hepatocyte markers. Datasets were analyzed separately to avoid cross-dataset normalization artifacts. As a limitation, differences in normalization strategies between datasets may affect direct quantitative comparisons; therefore, the analysis focuses on relative expression patterns and marker consistency rather than absolute expression values.

To assess hepatocyte spatial zonation, a continuous porto-central zonation axis was computed based on established periportal and pericentral gene expression signatures from (MacParland et al, 2018). For each hepatocyte, module scores for periportal and pericentral programs were calculated using Seurat’s AddModuleScore function, which computes the average expression of a gene set relative to control genes matched for expression level. A continuous zonation coordinate was then defined as the standardized difference between pericentral and periportal module scores:

$${{{\rm{Zonation\; axis}}}}={{{\rm{scale}}}}({{{\rm{Pericentral\; score}}}}-{{{\rm{Periportal\; score}}}})$$

To categorize cells into periportal-like, pericentral-like, or intermediate states, a strict data-driven threshold was applied to the continuous zonation axis. The optimal threshold ( ± 1.75 standard deviations) was selected empirically to capture only cells exhibiting strong transcriptional bias toward either program while minimizing misclassification of intermediate cells.

##### Re-analysis of GSE244832 and GSE253493 datasets for S100A10 and ANXA2 expression

Publicly available single-nucleus RNA-sequencing (snRNA-seq) data from human liver samples were obtained from Data ref: GEO GSE244832, 2024. This dataset comprises snRNA-seq profiles from 18 human liver samples including Healthy, MASLD, and MASH disease states. The gene-cell count matrix was imported into R and analysed using the Seurat package (v4). Gene expression data were normalized using Seurat’s NormalizeData function. Highly variable genes were identified using the variance-stabilizing transformation (“vst”) method, selecting the top 2000 variable genes. Data were scaled, and principal component analysis (PCA) was performed using the variable gene set. Hepatic stellate cells (HSCs) were identified based on expression of canonical marker genes derived from the Panglao database. Marker expression was assessed using feature plots and dot plots, including established HSC markers (LRAT, RBP1, CYGB, PDGFRB, DES, COL1A1, NGFR). Within the HSC population, expression of ANXA2 and S100A10 was examined across disease conditions (Healthy, MASLD, and MASH) which were treated as categorical variables and ordered accordingly.

Bulk RNA-sequencing data from primary human HSCs were obtained from Data ref: GEO GSE253493, 2024. This dataset comprises RNA-seq profiles from cultured primary human HSCs treated with vehicle (solvent control) or TGF-β (10 ng/mL) for 24 h, with three biological replicates per condition. Raw gene-level count tables were imported into R and merged across samples based on Ensembl gene identifiers. Differential expression analysis and normalization were performed using the DESeq2 package (v3.1.0). Size factors and dispersion estimates were calculated using the default DESeq2 workflow, and normalized counts were extracted for downstream visualization. Ensembl gene identifiers were mapped to human gene symbols using the org.Hs.eg.db annotation package, after removal of Ensembl version numbers. Normalized expression levels of ANXA2 and S100A10 were extracted and visualized across conditions using violin plots overlaid with individual sample values. Normalized counts reflect library size-adjusted RNA abundance and are displayed on a log₁₀ scale.

### Graphics

Figures and Synopsis have been generated with BioRender.com.

### Data representation and statistics

All the data are presented as mean ± standard deviation (SD). Normal distribution was tested using the Shapiro–Wilk normality test. Sample size was adjusted for each experiment depending on the variability of the assay. Outliers test was performed using the ROUT method (*Q*  =  1%). Quantification of Masson’s trichrome staining in HLO sections was performed in a blinded manner, with the evaluator unaware of sample identity or treatment group. Statistical significances were assessed using unpaired two-tailed Student’s *t* test, Mann–Whitney, one-way ANOVA followed by appropriate multiple comparisons test, or Kruskal–Wallis. Statistical significance was declared when ∗*P* < 0.05, ∗∗*P* < 0.01, ∗∗∗*P* < 0.001, ∗∗∗∗*P* < 0.0001, ns = not significant and analyses were performed using GraphPad Prism 10 Software (GraphPad Software, San Diego, CA, USA). Exact *P* values are provided in Appendix Table S2.

### Ethics **statement**

The Geneva Health Head Office granted ethical authority to utilize Embryonic Stem Cells (ESCs) HS420 cells (authorization number R-FP-S-2-0028), and the procedure was carried out in accordance with Swiss norms on research using ESCs.

## Supplementary information


Appendix
Peer Review File
Source data Fig. 1
Source data Fig. 2
Source data Fig. 3
Source data Fig. 4
Source data Fig. 5
Dataset EV1
Expanded View Figures


## Data Availability

Raw data for single-cell RNA sequencing are available at Gene Expression Omnibus (GEO) under the accession GSE313271. The source data of this paper are collected in the following database record: biostudies:S-SCDT-10_1038-S44321-026-00464-y.
