# Author Correction: S100A10-ANXA2 tetramer inhibition hampers hepatic stellate cell activation in human MASLD organoids

**DOI:** 10.1038/s44321-026-00512-7

**Published:** 2026-08-26

**Authors:** Miranda Türkal, Christine Maeder, Marta Correia de Sousa, Margot Fournier, Sanae El-Harane, Monika Gjorgjieva, Michelangelo Foti, Pierre Maechler, Etienne Delangre

**Affiliations:** 1 Department of Cell Physiology and Metabolism, Faculty of Medicine, University of Geneva, Geneva, Switzerland; 2 Faculty of Medicine, Diabetes Center, University of Geneva, Geneva, Switzerland; 3 Department of Medicine, Faculty of Medicine, University of Geneva, Geneva, Switzerland

**Correction to:** *EMBO Molecular Medicine* (2026) 18:2920–2945. https://doi.org/10.1038/s44321-026-00464-y | Published online 10 June 2026

The authors contacted the journal after identifying a typographical error in an author’s name in the published article.

**The 8th author’s name is corrected**.

The 8th author’s name is corrected from: Pierre Maecher

To: (Changes in bold.)

**Pierre Maechler**

