# Supplementary material for: S100A10-ANXA2 tetramer inhibition hampers hepatic stellate cell activation in human MASLD organoids
Source: EMBO Mol Med. 2026 Jun 10;18(7):2920–45. doi: 10.1038/s44321-026-00464-y (PMC13365249; doi:10.1038/s44321-026-00464-y)
Supplement: Supplementary file 1 — Appendix [file 44321_2026_464_MOESM1_ESM.pdf]

Appendix to “S100A10-ANXA2 TETRAMER INHIBITION HAMPERS HEPATIC STELLATE CELL ACTIVATION IN HUMAN MASLD ORGANOIDS”

Appendix Table S1…………………………………………………………………………..**2**

Appendix Table S2…………………………………………………………………………..**3**

Appendix Table S1: List of primers used for qPCR

Name	Forward Sequence (5′-3′)	Reverse sequence (5′-3′)
ACTA2	TGATCACCATCGGAAATGAA	CGGCTTCATCGTATTCCTGT
COL1A	AACATGACCAAAAACCAAAAGTG	CATTGTTTCCTGTGTCTTCTGG
COL3A	GGAAACACTGGTGGACAGATTC	CTGGAGAGAAGTCGAAGGAAT
PDGFB	AATGCTGAGCGACCACTCCAT	TCGGGTCATGTTCAAGTCCAGC
PDGFRB	TGATGCCGAGGAACTATTCATCT	TTTCTTCTCGTGCAGTGTCAC
VIM	GCCCTAGACGAACTGGGTC	GGCTGCAACTGCCTAATGAG
MMP2	CTTCCA AGTCTGGAGCGATGT	TACCGTCAA AGGGGTATCCAT
TIMP1	AGACACCAGAGAACCCACCA	TTTGCAGGGGATGGATAAAC
CYCA	ATGGTCAACCCCACCGTGT	TCTGCTGTCTTTGGGACCTTGTC
TBP	GCCCGAAACGCCGAATATA	CGTGGCTCTCTTATCCTCATGA
BCL2	GCCCTGTGGATGACTGAGTA	GCCAAACTGAGCAGAGTCTT
BCL2L1	GCCAGGCCTTCAACCACTAT	ACCATTCGTGGGTGGTCTTC
BAX	CCCGAGAGGTCTTTTTCCGAG	CCAGCCCATGATGGTTCTGAT
BAK1	GTTTTCCGCAGCTACGTTTTT	GCAGAGGTAAGGTGACCATCTC

Appendix Table S2: p-values determined for each panel

Figure	Comparison	P-value	Test
1A	TGF-β1 vs. Vehicle	0.0065	One-Way ANOVA followed by Sidak's multiple comparisons
	TGF-β1 vs. TGF-β1 + A2ti1 50 µM	0.0029	One-Way ANOVA followed by Sidak's multiple comparisons
	TGF-β1 vs. TGF-β1 + A2ti-1 25 µM	>0.9999	One-Way ANOVA followed by Sidak's multiple comparisons
	TGF-β1 vs. TGF-β1 + A2ti-1 12.5 µM	0.0216	One-Way ANOVA followed by Sidak's multiple comparisons
1B	ACTA2	0.0107	Unpaired t-test – Welch's correction
	COL1A	0.0145	Unpaired t-test – Welch's correction
	COL3A	0.0164	Unpaired t-test – Welch's correction
	VIM	0.8638	Unpaired t-test – Welch's correction
	TIMP1	0.1823	Unpaired t-test – Welch's correction
	MMP2	0.0148	Unpaired t-test – Welch's correction
1C	S100A10<br>siCTL vs. siCTL + TGF-β1	0.0601	One-Way ANOVA followed by Sidak's multiple comparisons
	S100A10<br>siCTL vs. siS100A10 + TGF-β1	<0.0001	One-Way ANOVA followed by Sidak's multiple comparisons
	S100A10<br>siCTL + TGF-β1 vs. siS100A10 + TGF-β1	0.0011	One-Way ANOVA followed by Sidak's multiple comparisons
	α-SMA<br>siCTL vs. siCTL + TGF-β1	<0.0001	Kruskal-Wallis followed by Dunn's multiple comparisons
	α-SMA<br>siCTL + TGF-β1 vs. siS100A10 + TGF-β1	0.0310	Kruskal-Wallis followed by Dunn's multiple comparisons
1D	ACTA2<br>siCTL vs siCTL + TGF-β1	0.0032	One-Way ANOVA followed by Sidak's multiple comparisons
	ACTA2<br>siCTL + TGF-β1 vs. siS100A10 + TGF-β1	0.0051	One-Way ANOVA followed by Sidak's multiple comparisons
	COL1A<br>siCTL vs siCTL + TGF-β1	0.0146	One-Way ANOVA followed by Sidak's multiple comparisons
	COL1A<br>siCTL + TGF-β1 vs. siS100A10 + TGF-β1	0.9405	One-Way ANOVA followed by Sidak's multiple comparisons
	COL3A<br>siCTL vs siCTL + TGF-β1	0.0565	One-Way ANOVA followed by Sidak's multiple comparisons
	COL3A<br>siCTL + TGF-β1 vs. siS100A10 + TGF-β1	0.9772	One-Way ANOVA followed by Sidak's multiple comparisons

	VIM<br><br>siCTL vs siCTL + TGF-β1	0.9988	One-Way ANOVA followed by Sidak's multiple comparisons
	VIM<br><br>siCTL + TGF-β1 vs. siS100A10 + TGF-β1	0.0330	One-Way ANOVA followed by Sidak's multiple comparisons
	TIMP1<br><br>siCTL vs siCTL + TGF-β1	0.0532	One-Way ANOVA followed by Sidak's multiple comparisons
	TIMP1<br><br>siCTL + TGF-β1 vs. siS100A10 + TGF-β1	0.0313	One-Way ANOVA followed by Sidak's multiple comparisons
	MMP2<br><br>siCTL vs siCTL + TGF-β1	0.0059	One-Way ANOVA followed by Sidak's multiple comparisons
	MMP2<br><br>siCTL + TGF-β1 vs. siS100A10 + TGF-β1	0.0104	One-Way ANOVA followed by Sidak's multiple comparisons
2G	Huh7 vs. HLOs	0.1460	Unpaired t-test
3C	Vehicle vs. OA/PA 400 µM	<0.0001	Kruskal-Wallis followed by Dunn's multiple comparisons
	Vehicle vs. OA/PA 600 µM	<0.0001	Kruskal-Wallis followed by Dunn's multiple comparisons
	OA/PA 400 µM vs. OA/PA 600 µM	0.3368	Kruskal-Wallis followed by Dunn's multiple comparisons
3D	Vehicle vs. OA/PA 400 µM	<0.0001	One-Way ANOVA followed by Sidak's multiple comparisons
	Vehicle vs. OA/PA 600 µM	<0.0001	One-Way ANOVA followed by Sidak's multiple comparisons
	OA/PA 400 µM vs. OA/PA 600 µM	0.1839	One-Way ANOVA followed by Sidak's multiple comparisons
3E	Vehicle vs. OA/PA 400 µM	0.0027	One-Way ANOVA followed by Sidak's multiple comparisons
	Vehicle vs. OA/PA 600 µM	<0.0001	One-Way ANOVA followed by Sidak's multiple comparisons
	OA/PA 400 µM vs. OA/PA 600 µM	0.0551	One-Way ANOVA followed by Sidak's multiple comparisons
3F	Vehicle vs. OA/PA 400 µM	0.1570	One-Way ANOVA followed by Sidak's multiple comparisons
	Vehicle vs. OA/PA 600 µM	0.0088	One-Way ANOVA followed by Sidak's multiple comparisons
	OA/PA 400 µM vs. OA/PA 600 µM	0.1570	One-Way ANOVA followed by Sidak's multiple comparisons
3I	Vehicle vs. TGF-β1	0.0025	One-Way ANOVA followed by Sidak's multiple comparisons
	Vehicle vs. OA/PA + TGF-β1	0.0144	One-Way ANOVA followed by Sidak's multiple comparisons
	TGF-β1 vs. OA/PA + TGF-β1	0.5612	One-Way ANOVA followed by Sidak's multiple comparisons

3J	ACTA2<br>Vehicle vs. TGF-β1	0.0046	One-Way ANOVA followed by Sidak's multiple comparisons
	ACTA2<br>Vehicle vs. OA/PA + TGF-β1	0.0885	One-Way ANOVA followed by Sidak's multiple comparisons
	ACTA2<br>TGF-β1 vs. OA/PA + TGF-β1	0.5842	One-Way ANOVA followed by Sidak's multiple comparisons
	COL1A<br>Vehicle vs. TGF-β1	0.0752	One-Way ANOVA followed by Sidak's multiple comparisons
	COL1A<br>Vehicle vs. OA/PA + TGF-β1	0.9967	One-Way ANOVA followed by Sidak's multiple comparisons
	COL1A<br>TGF-β1 vs. OA/PA + TGF-β1	0.1114	One-Way ANOVA followed by Sidak's multiple comparisons
	COL3A<br>Vehicle vs. TGF-β1	0.0231	One-Way ANOVA followed by Sidak's multiple comparisons
	COL3A<br>Vehicle vs. OA/PA + TGF-β1	>0.9999	One-Way ANOVA followed by Sidak's multiple comparisons
	COL3A<br>TGF-β1 vs. OA/PA + TGF-β1	0.0465	One-Way ANOVA followed by Sidak's multiple comparisons
	VIM<br>Vehicle vs. TGF-β1	0.1967	One-Way ANOVA followed by Sidak's multiple comparisons
	VIM<br>Vehicle vs. OA/PA + TGF-β1	0.9618	One-Way ANOVA followed by Sidak's multiple comparisons
	VIM<br>TGF-β1 vs. OA/PA + TGF-β1	0.3988	One-Way ANOVA followed by Sidak's multiple comparisons
	TIMP1<br>Vehicle vs. TGF-β1	0.2172	One-Way ANOVA followed by Sidak's multiple comparisons
	TIMP1<br>Vehicle vs. OA/PA + TGF-β1	0.6783	One-Way ANOVA followed by Sidak's multiple comparisons
	TIMP1<br>TGF-β1 vs. OA/PA + TGF-β1	0.7651	One-Way ANOVA followed by Sidak's multiple comparisons
	MMP2<br>Vehicle vs. TGF-β1	0.0932	One-Way ANOVA followed by Sidak's multiple comparisons
	MMP2<br>Vehicle vs. OA/PA + TGF-β1	0.3734	One-Way ANOVA followed by Sidak's multiple comparisons
	MMP2<br>TGF-β1 vs. OA/PA + TGF-β1	0.6977	One-Way ANOVA followed by Sidak's multiple comparisons
3K	Vehicle vs. TGF-β1	0.0497	Brown-Forsythe and Welch One-Way ANOVA followed by Dunnett's multiple comparisons
	Vehicle vs. OA/PA + TGF-β1	0.0390	Brown-Forsythe and Welch One-Way ANOVA followed by Dunnett's multiple comparisons
	TGF-β1 vs. OA/PA + TGF-β1	0.2846	Brown-Forsythe and Welch One-Way ANOVA followed by Dunnett's multiple comparisons
3L	S100A10<br>Vehicle vs. OA/PA	0.7799	One-Way ANOVA followed by Sidak's multiple comparisons
	S100A10<br>Vehicle vs. OA/PA + TGF-β1	0.8223	One-Way ANOVA followed by Sidak's multiple comparisons

	S100A10<br>OA/PA vs. OA/PA + TGF-β1	0.4602	One-Way ANOVA followed by Sidak's multiple comparisons
	ANXA2<br>Vehicle vs. OA/PA	0.9926	One-Way ANOVA followed by Sidak's multiple comparisons
	ANXA2<br>Vehicle vs. OA/PA + TGF-β1	0.0405	One-Way ANOVA followed by Sidak's multiple comparisons
	ANXA2<br>OA/PA vs. OA/PA + TGF-β1	0.0498	One-Way ANOVA followed by Sidak's multiple comparisons
4B	OA/PA vs. OA/PA + A2ti-1	0.3686	One-Way ANOVA followed by Sidak's multiple comparisons
	OA/PA + TGF-β1 vs. OA/PA + TGF-β1 + A2ti-1	0.8092	One-Way ANOVA followed by Sidak's multiple comparisons
4C	TGF-β1 vs. TGF-β1 + A2ti-1	0.0235	One-Way ANOVA followed by Sidak's multiple comparisons
	OA/PA + TGF-β1 vs. OA/PA + TGF-β1 + A2ti-1	0.0032	One-Way ANOVA followed by Sidak's multiple comparisons
4D	TGF-β1 vs. TGF-β1 + A2ti-1	0.0257	Unpaired t-test – Welch's correction
4E	ACTA2<br>TGF-β1 vs. TGF-β1 + A2ti-1	<0.0001	Unpaired t-test – Welch's correction
	COL1A<br>TGF-β1 vs. TGF-β1 + A2ti-1	0.0006	Unpaired t-test – Welch's correction
	COL3A<br>TGF-β1 vs. TGF-β1 + A2ti-1	0.0001	Unpaired t-test – Welch's correction
	VIM<br>TGF-β1 vs. TGF-β1 + A2ti-1	0.2393	Unpaired t-test – Welch's correction
	TIMP1<br>TGF-β1 vs. TGF-β1 + A2ti-1	0.7288	Unpaired t-test – Welch's correction
	MMP2<br>TGF-β1 vs. TGF-β1 + A2ti-1	0.0135	Unpaired t-test – Welch's correction
4F	TGF-β1 vs. TGF-β1 + A2ti-1	0.8063	Kruskal-Wallis followed by Dunn's multiple comparisons
	OA/PA + TGF-β1 vs. OA/PA + TGF-β1 + A2ti-1	>0.9999	Kruskal-Wallis followed by Dunn's multiple comparisons
4H	TGF-β1 vs. TGF-β1 + A2ti-1	0.0059	Unpaired t-test – Welch's correction
5C (left)	Vehicle vs. TGF-β1	<0.0001	One-Way ANOVA followed by Sidak's multiple comparisons
	TGF-β1 vs. TGF-β1 SB	<0.0001	One-Way ANOVA followed by Sidak's multiple comparisons
	TGF-β1 vs. TGF-β1 A2ti.1	0.8188	One-Way ANOVA followed by Sidak's multiple comparisons
	TGF-β1 A2ti.1 vs. TGF-β1 SB A2ti.1	<0.0001	One-Way ANOVA followed by Sidak's multiple comparisons
5C (right)	Vehicle vs. TGF-β1	0.2462	One-Way ANOVA followed by Sidak's multiple comparisons
	TGF-β1 vs. TGF-β1 SB	0.9998	One-Way ANOVA followed by Sidak's multiple comparisons
	TGF-β1 vs. TGF-β1 A2ti.1	0.3569	One-Way ANOVA followed by Sidak's multiple comparisons

	TGF-β1 A2ti.1 vs. TGF-β1 SB A2ti.1	0.0175	One-Way ANOVA followed by Sidak's multiple comparisons
5D	Vehicle vs. TGF-β1	0.0008	One-Way ANOVA followed by Sidak's multiple comparisons
	TGF-β1 vs. TGF-β1 A2ti.1	0.0068	One-Way ANOVA followed by Sidak's multiple comparisons
5E	PDGFB<br>TGF-β1 vs. TGF-β1 A2ti.1	<0.0001	Unpaired t-test
	PDGFRB<br>TGF-β1 vs. TGF-β1 A2ti.1	0.0356	Unpaired t-test
EV1C	Vehicle vs. A2ti-1 12.5 µM	0.9993	One-Way ANOVA followed by Sidak's multiple comparisons
	Vehicle vs. A2ti-1 25 µM	0.9912	One-Way ANOVA followed by Sidak's multiple comparisons
	Vehicle vs. A2ti-1 50 µM	0.9489	One-Way ANOVA followed by Sidak's multiple comparisons
EV1D	Vehicle vs. A2ti-1 50 µM	0.0939	Unpaired t-test – Welch's correction
EV1E	TGF-β1 + siCTL vs. TGF-β1 + siS100A10	0.0022	One-Way ANOVA followed by Sidak's multiple comparisons
	TGF-β1 + siCTL vs. TGF-β1 + siS100A10 + A2ti-1	0.0002	One-Way ANOVA followed by Sidak's multiple comparisons
	TGF-β1 + siS100A10 vs. TGF-β1 + siS100A10 + A2ti-1	0.2956	One-Way ANOVA followed by Sidak's multiple comparisons
EV3A	OAPA vs. OAPA A2ti-1	0.9239	One-Way ANOVA followed by Sidak's multiple comparisons
	OAPA + TGF-β1 vs. OAPA + TGF-β1 + A2ti-1	0.9965	One-Way ANOVA followed by Sidak's multiple comparisons
EV3B	TGF-β1 vs. TGF-β1 A2ti.1	0.5103	Unpaired t-test – Welch's correction
EV3C	BCL2	0.3084	Unpaired t-test – Welch's correction
	BCL2L1	0.3034	Unpaired t-test – Welch's correction
	BAX	0.8922	Unpaired t-test – Welch's correction
	BAK1	0.6825	Mann-Whitney test
EV3D	ACTA2	0.3143	Mann-Whitney test
	COL1A	0.6825	Mann-Whitney test
	COL3A	0.1270	Mann-Whitney test
	VIM	0.2625	Unpaired t-test – Welch's correction
	TIMP1	0.0197	Unpaired t-test – Welch's correction
	MMP2	0.5128	Unpaired t-test – Welch's correction
EV4 (left)	Vehicle vs. TGF-β1	<0.0001	One-Way ANOVA followed by Sidak's multiple comparisons
	TGF-β1 vs. TGF-β1 SB	<0.0001	One-Way ANOVA followed by Sidak's multiple comparisons
	TGF-β1 vs. TGF-β1 A2ti-1	0.9978	One-Way ANOVA followed by Sidak's multiple comparisons
	TGF-β1 A2ti-1 vs. TGF-β1 SB A2ti-1	<0.0001	One-Way ANOVA followed by Sidak's multiple comparisons

EV4 (right)	Vehicle vs. TGF-β1	0.0108	Kruskal-Wallis followed by Dunn's multiple comparisons test
	TGF-β1 vs. TGF-β1 SB	0.0557	Kruskal-Wallis followed by Dunn's multiple comparisons test
	TGF-β1 vs. TGF-β1 A2ti-1	>0.9999	Kruskal-Wallis followed by Dunn's multiple comparisons test
	TGF-β1 A2ti-1 vs. TGF-β1 SB A2ti-1	0.1910	Kruskal-Wallis followed by Dunn's multiple comparisons test
