# Supplementary figures and images for: S100A10-ANXA2 tetramer inhibition hampers hepatic stellate cell activation in human MASLD organoids
Source: EMBO Mol Med. 2026 Jun 10;18(7):2920–45. doi: 10.1038/s44321-026-00464-y (PMC13365249; doi:10.1038/s44321-026-00464-y)

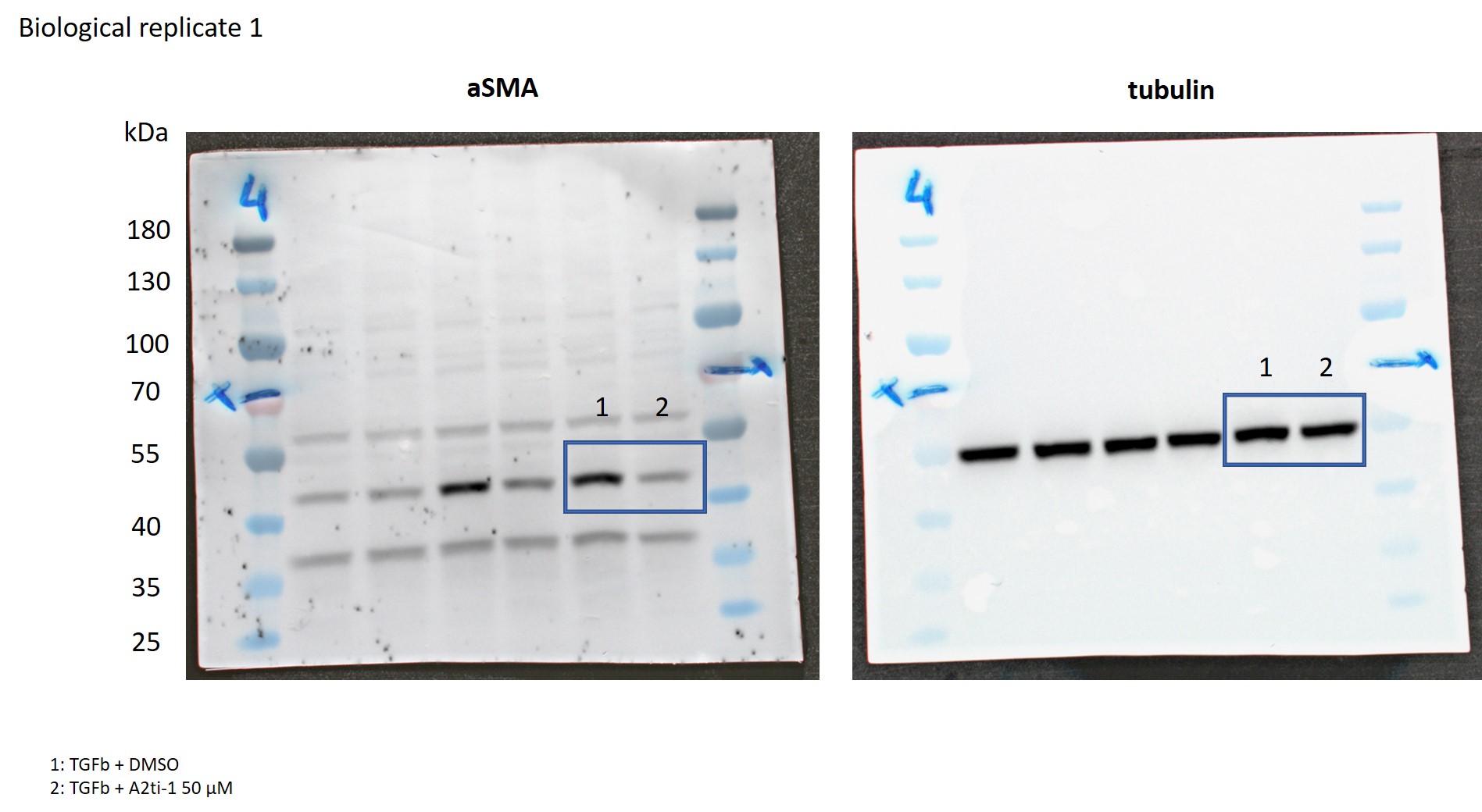

Supplement: Supplementary file 3 — Source data Fig. 1 [file 44321_2026_464_MOESM3_ESM.zip › Figure 1/1A/Replicate 1.jpg]

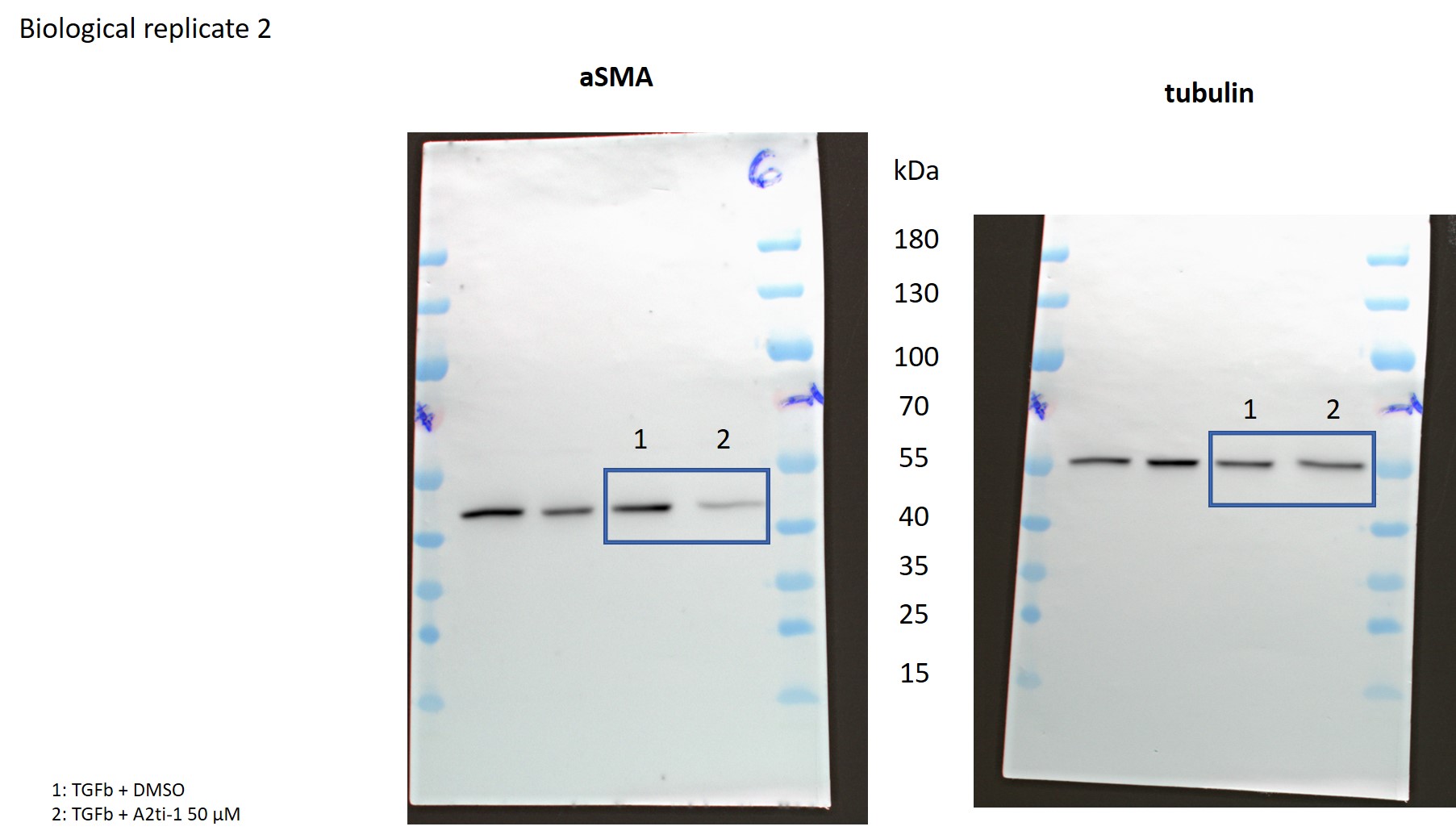

Supplement: Supplementary file 3 — Source data Fig. 1 [file 44321_2026_464_MOESM3_ESM.zip › Figure 1/1A/Replicate 2.jpg]

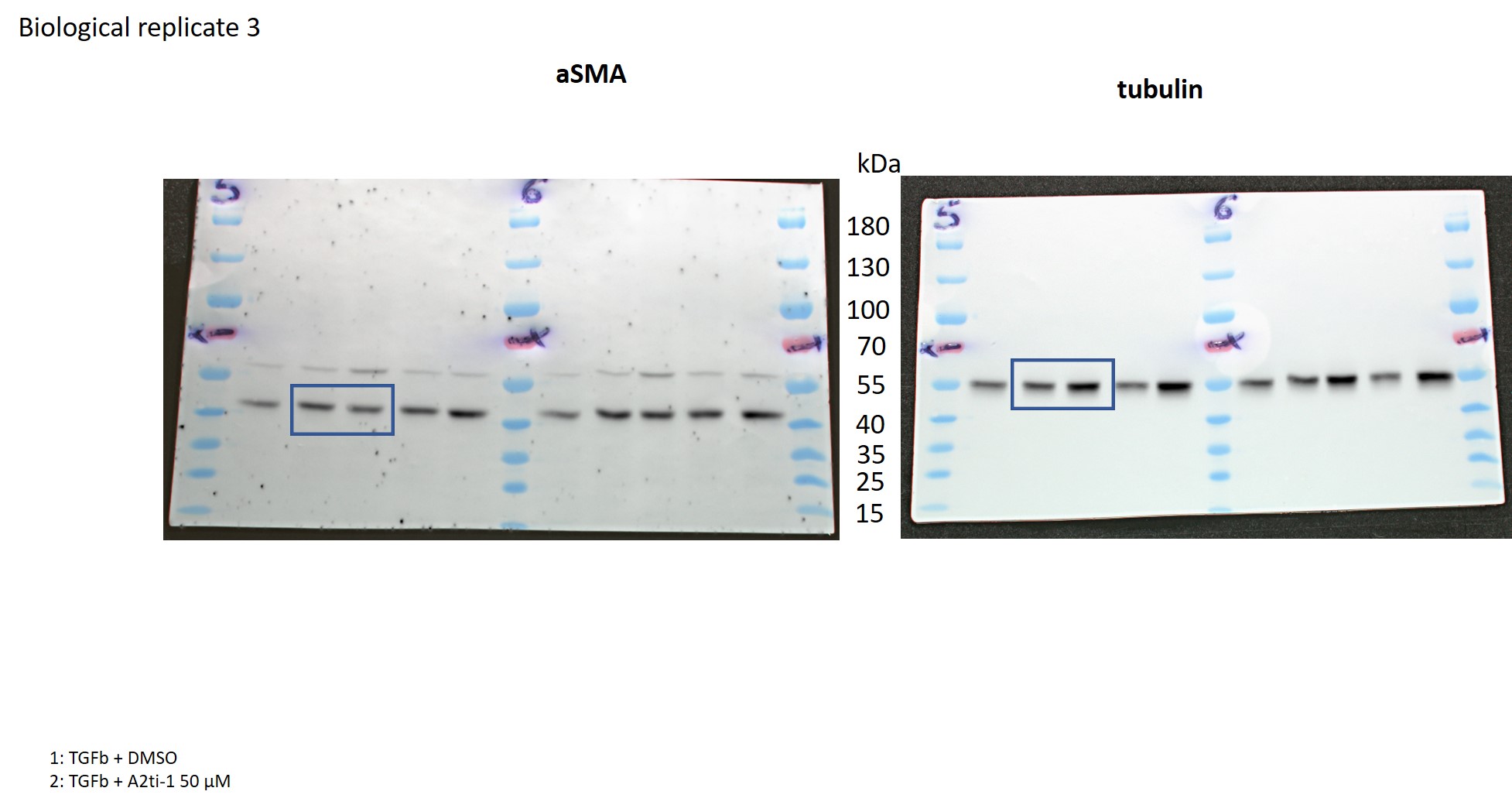

Supplement: Supplementary file 3 — Source data Fig. 1 [file 44321_2026_464_MOESM3_ESM.zip › Figure 1/1A/Replicate 3.jpg]

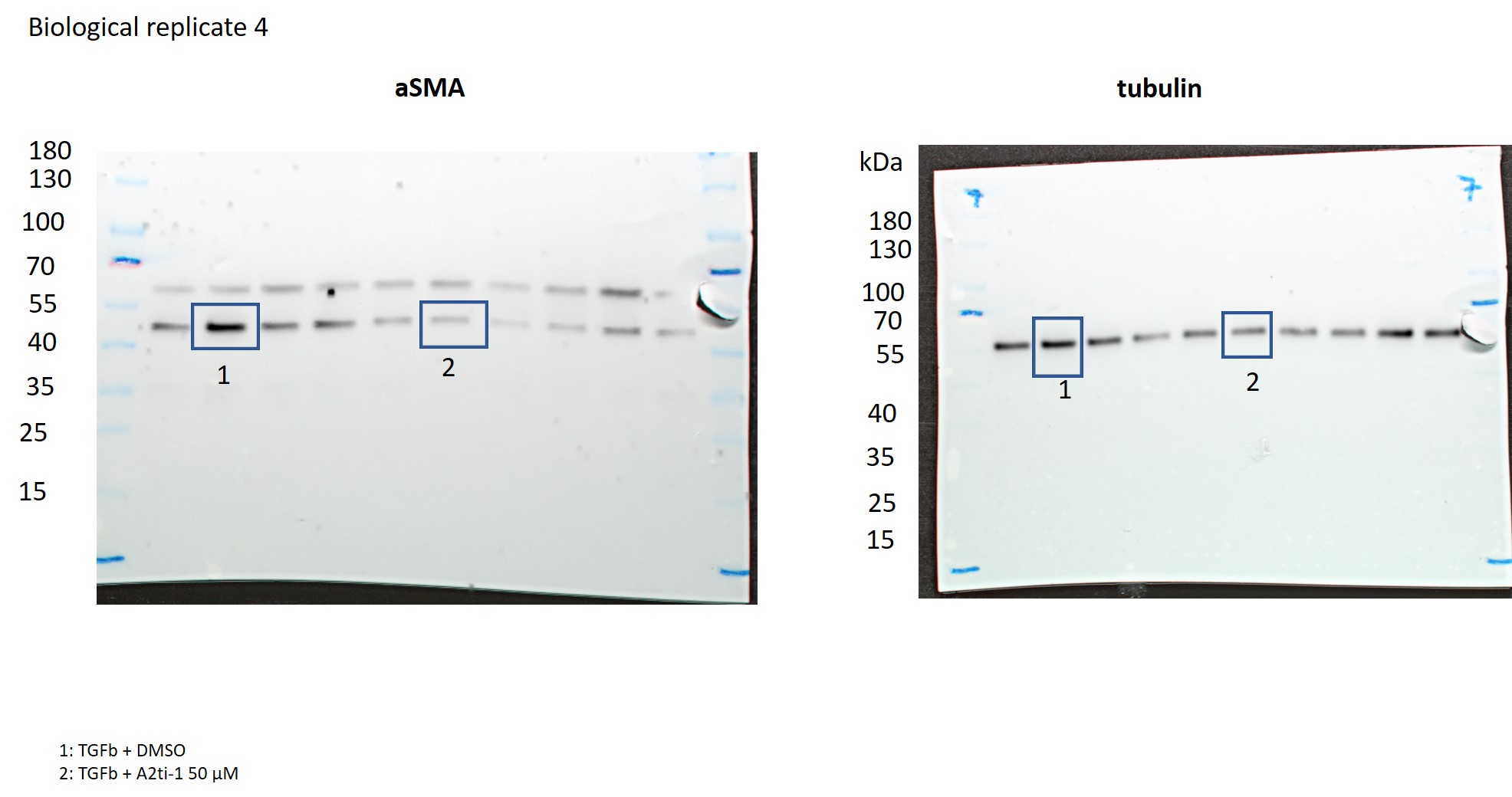

Supplement: Supplementary file 3 — Source data Fig. 1 [file 44321_2026_464_MOESM3_ESM.zip › Figure 1/1A/Replicate 4.jpg]

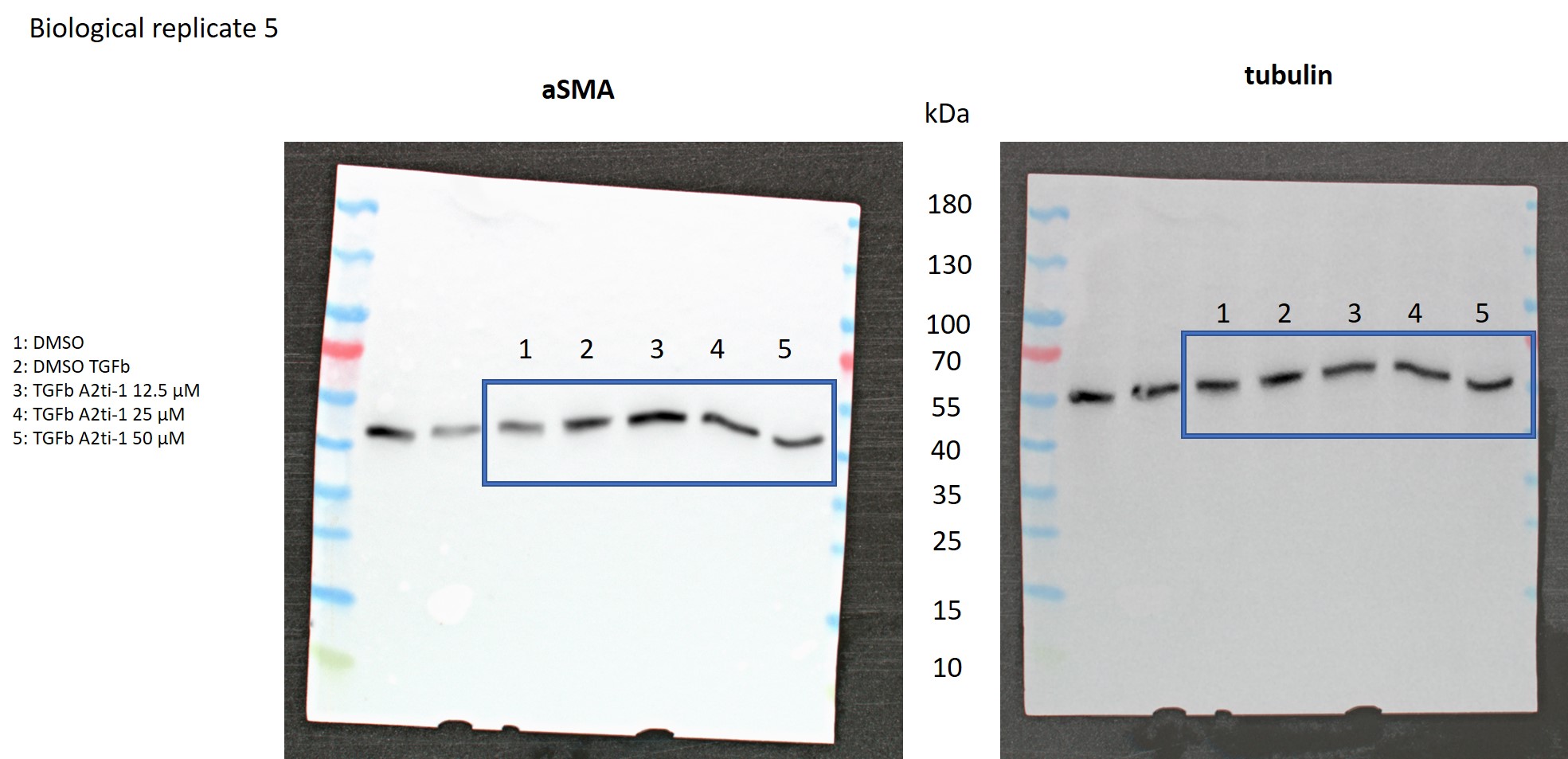

Supplement: Supplementary file 3 — Source data Fig. 1 [file 44321_2026_464_MOESM3_ESM.zip › Figure 1/1A/Replicate 5.jpg]

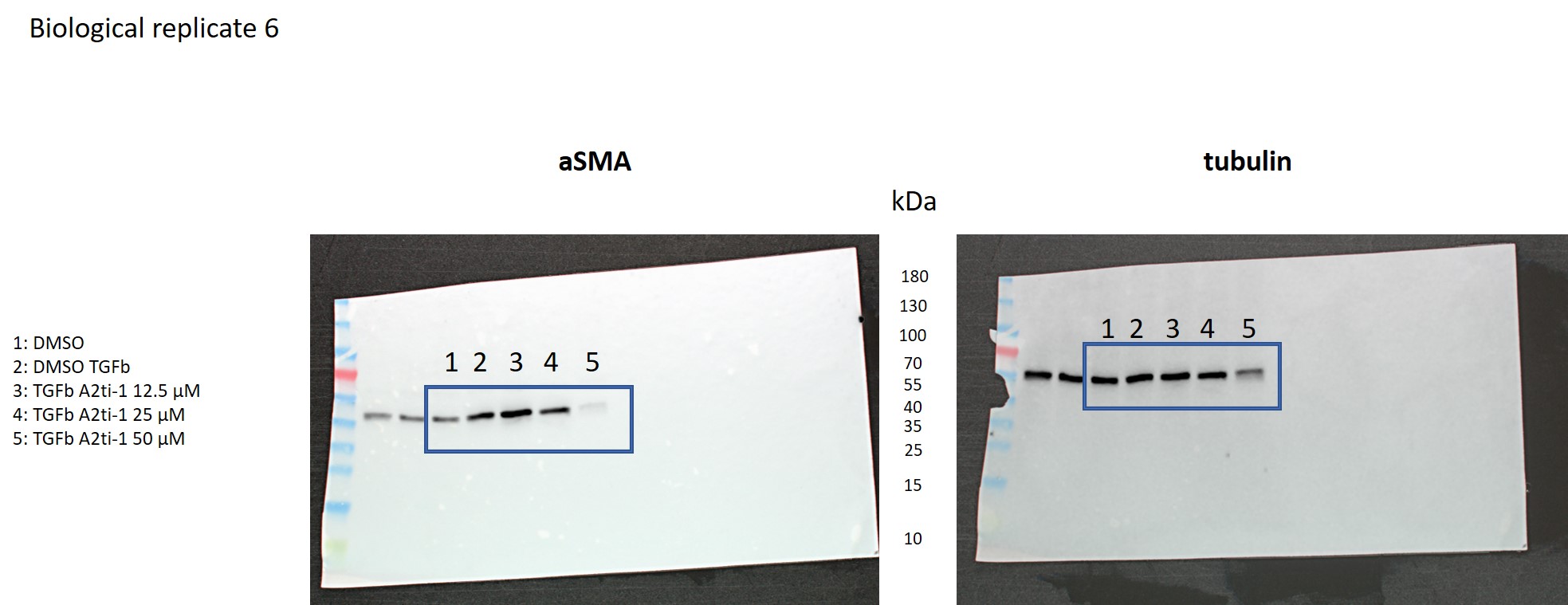

Supplement: Supplementary file 3 — Source data Fig. 1 [file 44321_2026_464_MOESM3_ESM.zip › Figure 1/1A/Replicate 6.jpg]

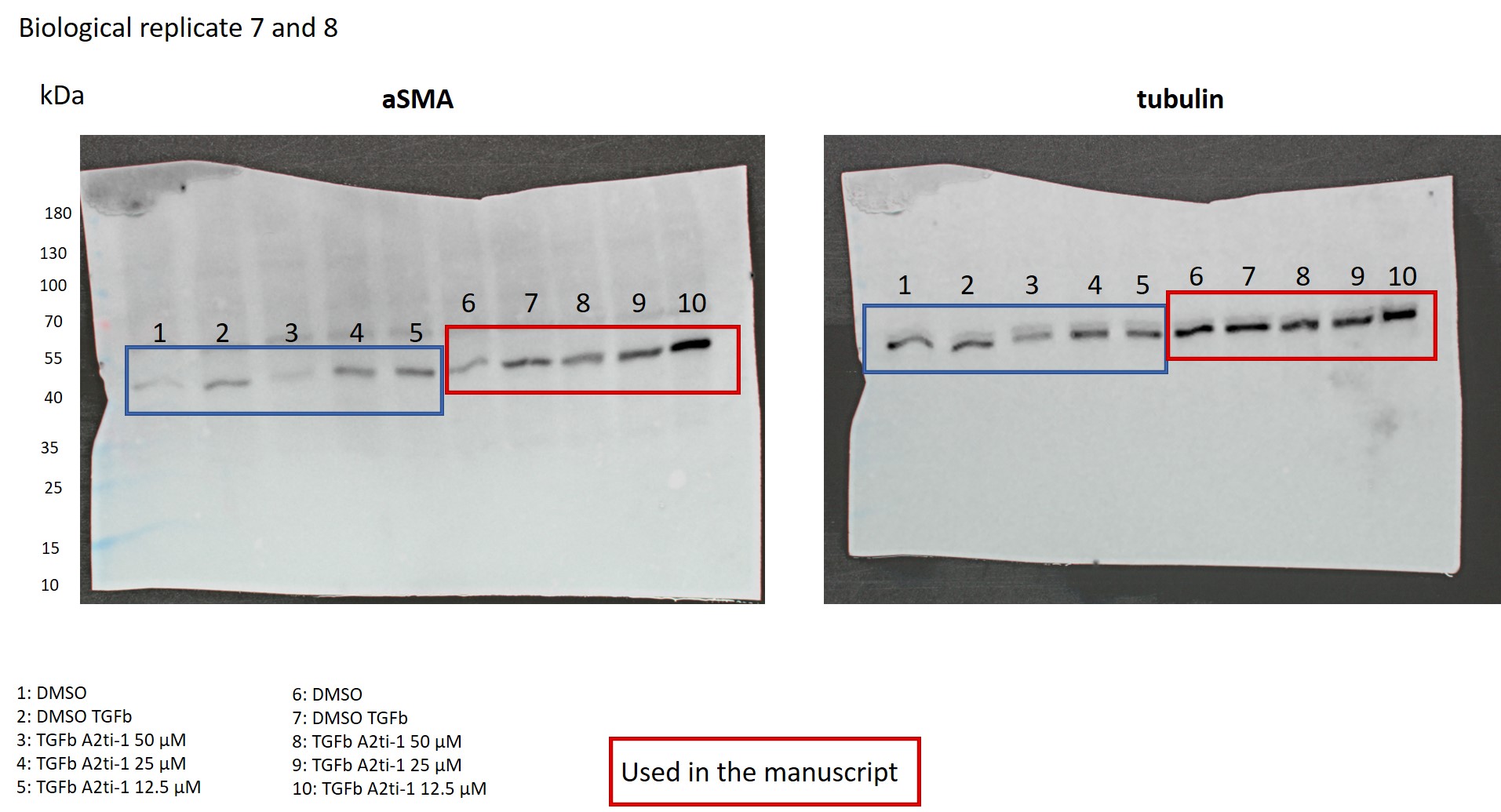

Supplement: Supplementary file 3 — Source data Fig. 1 [file 44321_2026_464_MOESM3_ESM.zip › Figure 1/1A/Replicate 7+8.jpg]

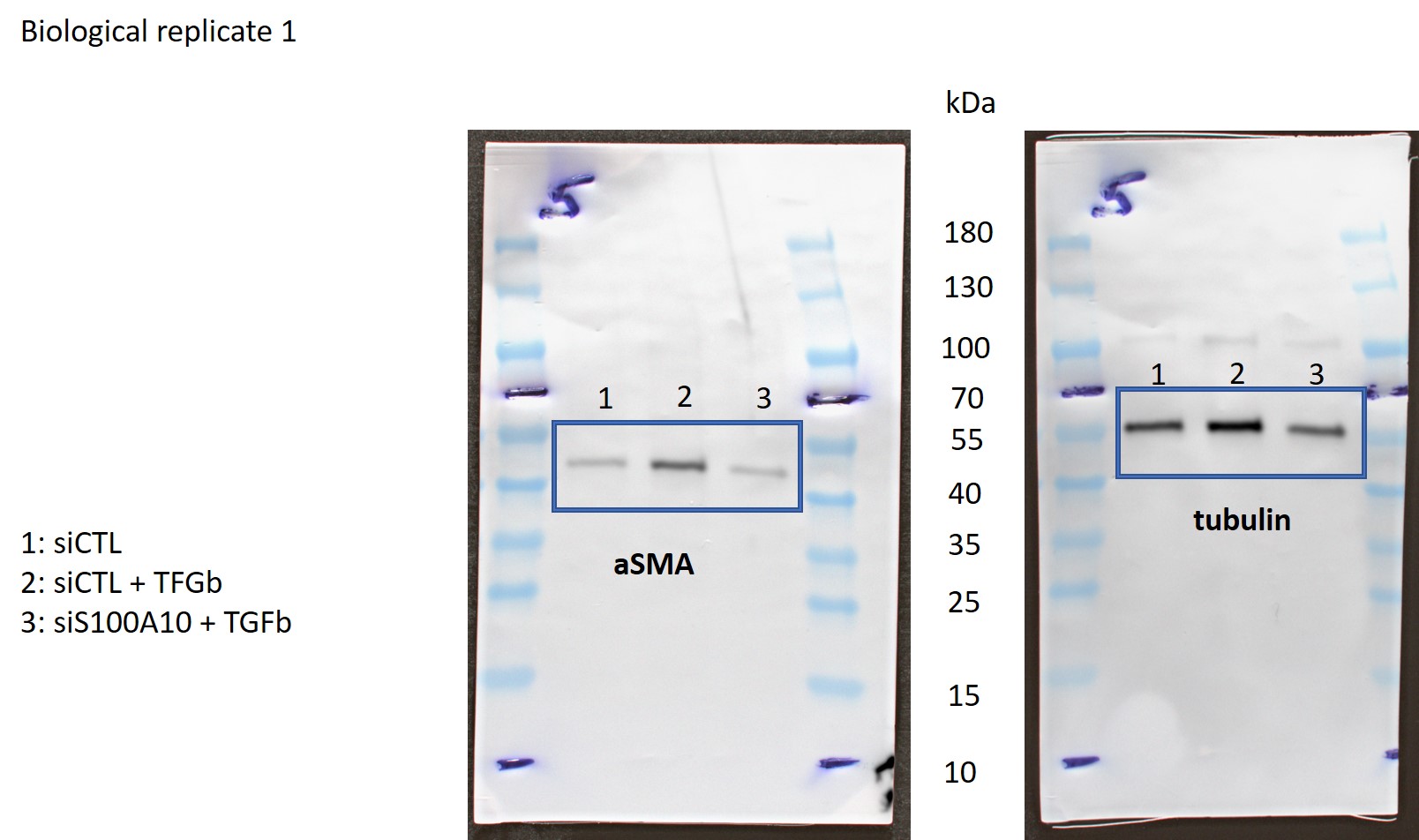

Supplement: Supplementary file 3 — Source data Fig. 1 [file 44321_2026_464_MOESM3_ESM.zip › Figure 1/1C/Replicate 1 - aSMA.jpg]

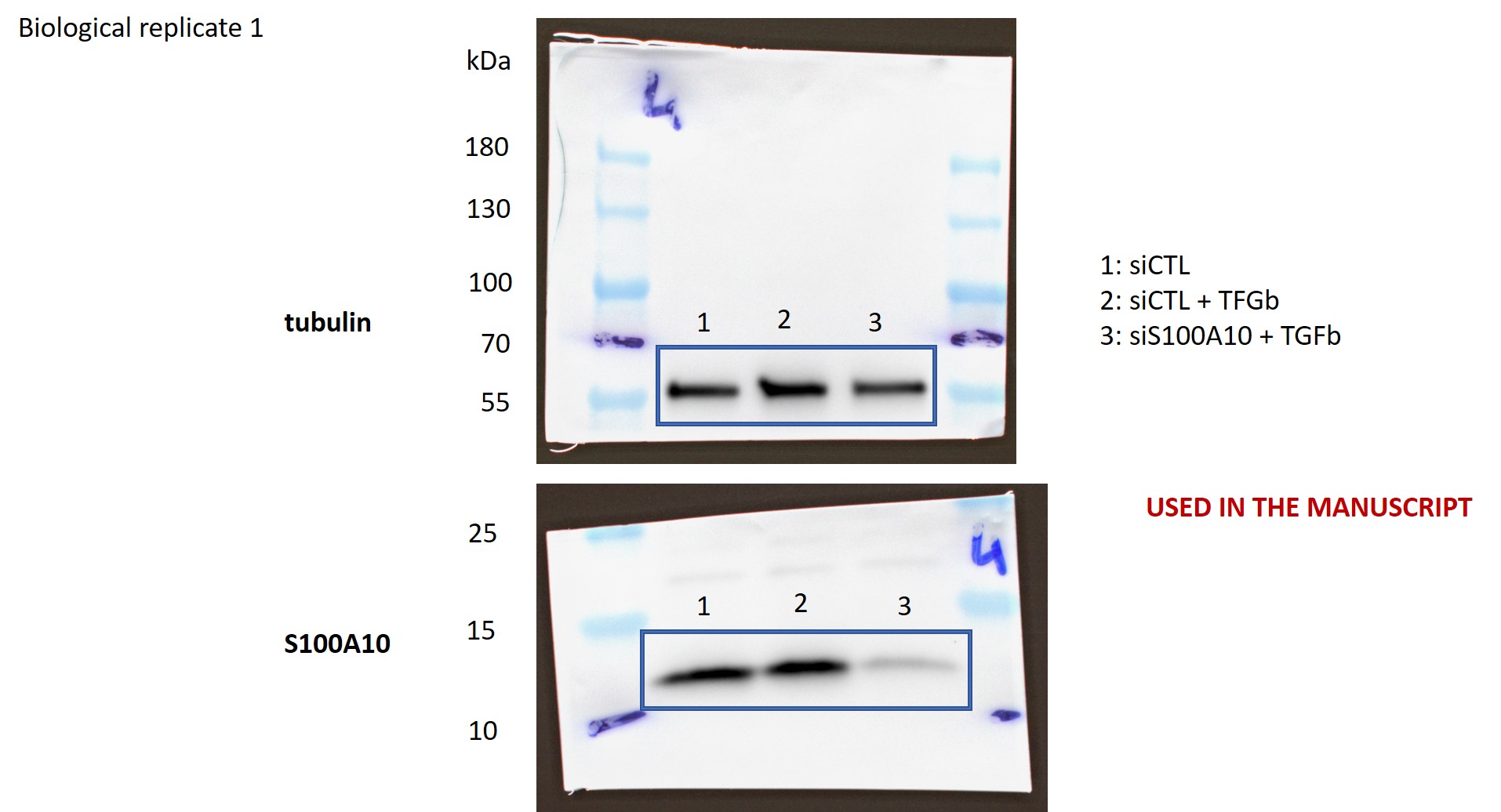

Supplement: Supplementary file 3 — Source data Fig. 1 [file 44321_2026_464_MOESM3_ESM.zip › Figure 1/1C/Replicate 1 - S100A10.jpg]

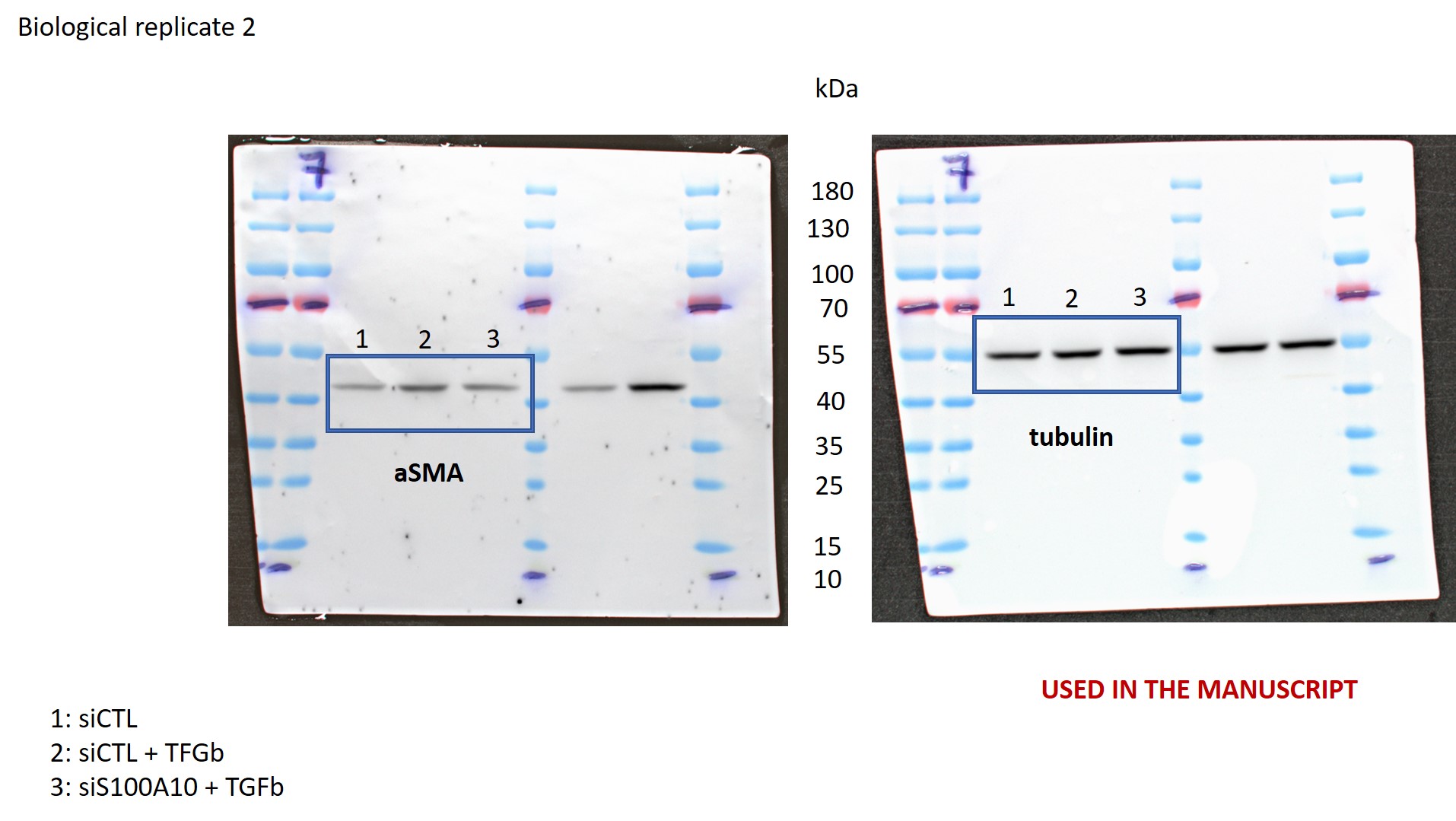

Supplement: Supplementary file 3 — Source data Fig. 1 [file 44321_2026_464_MOESM3_ESM.zip › Figure 1/1C/Replicate 2 - aSMA.jpg]

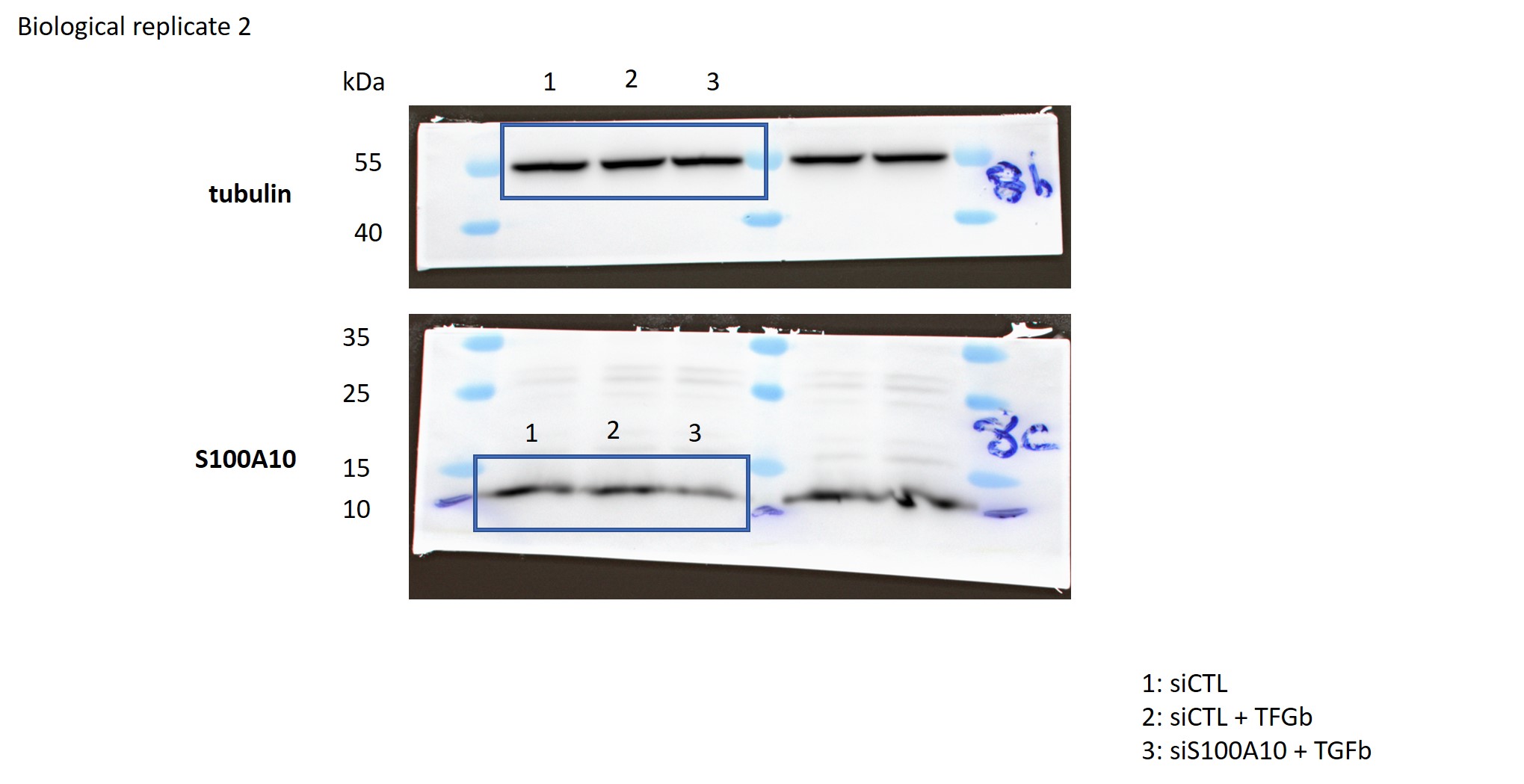

Supplement: Supplementary file 3 — Source data Fig. 1 [file 44321_2026_464_MOESM3_ESM.zip › Figure 1/1C/Replicate 2 - S100A10.jpg]

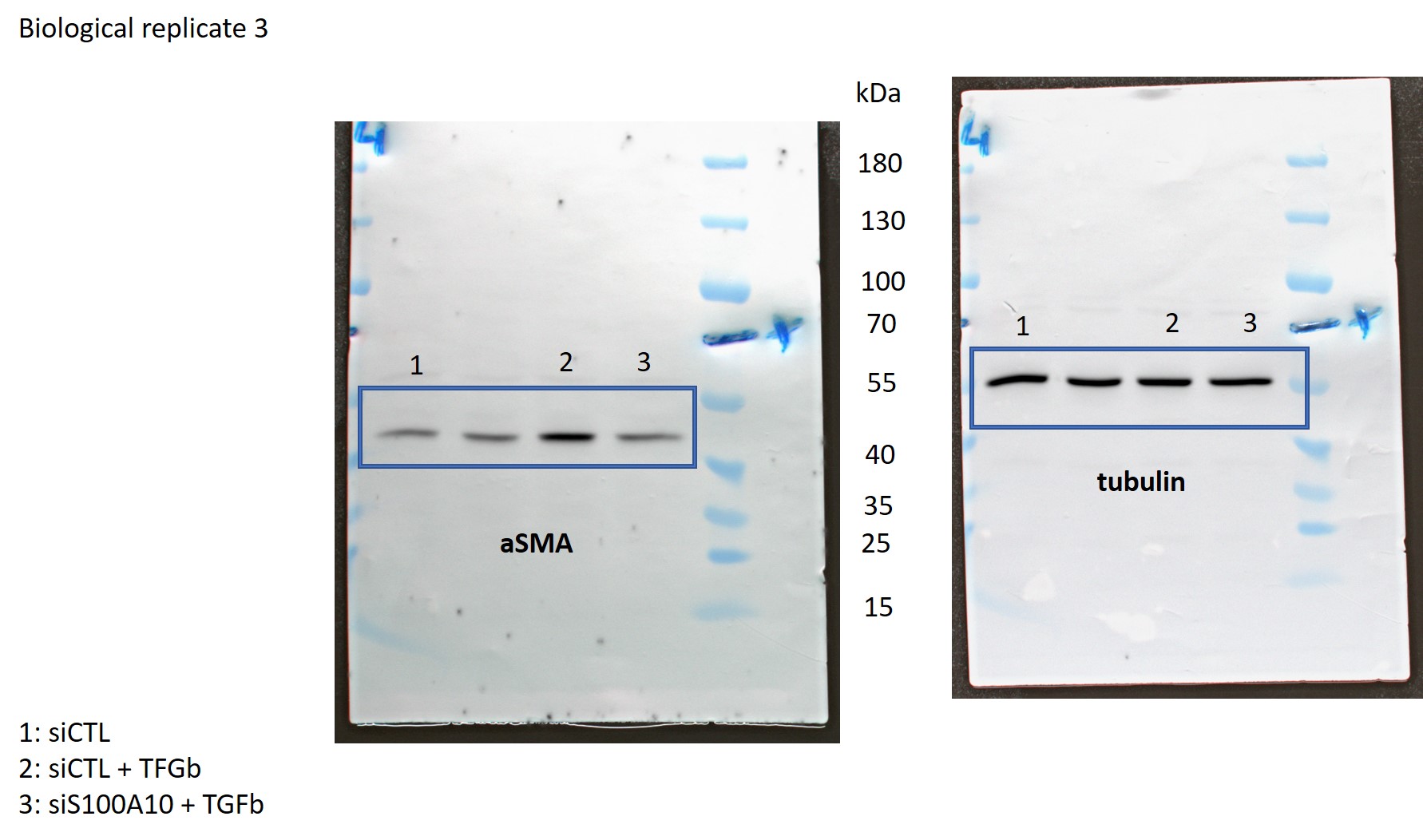

Supplement: Supplementary file 3 — Source data Fig. 1 [file 44321_2026_464_MOESM3_ESM.zip › Figure 1/1C/Replicate 3 - aSMA.jpg]

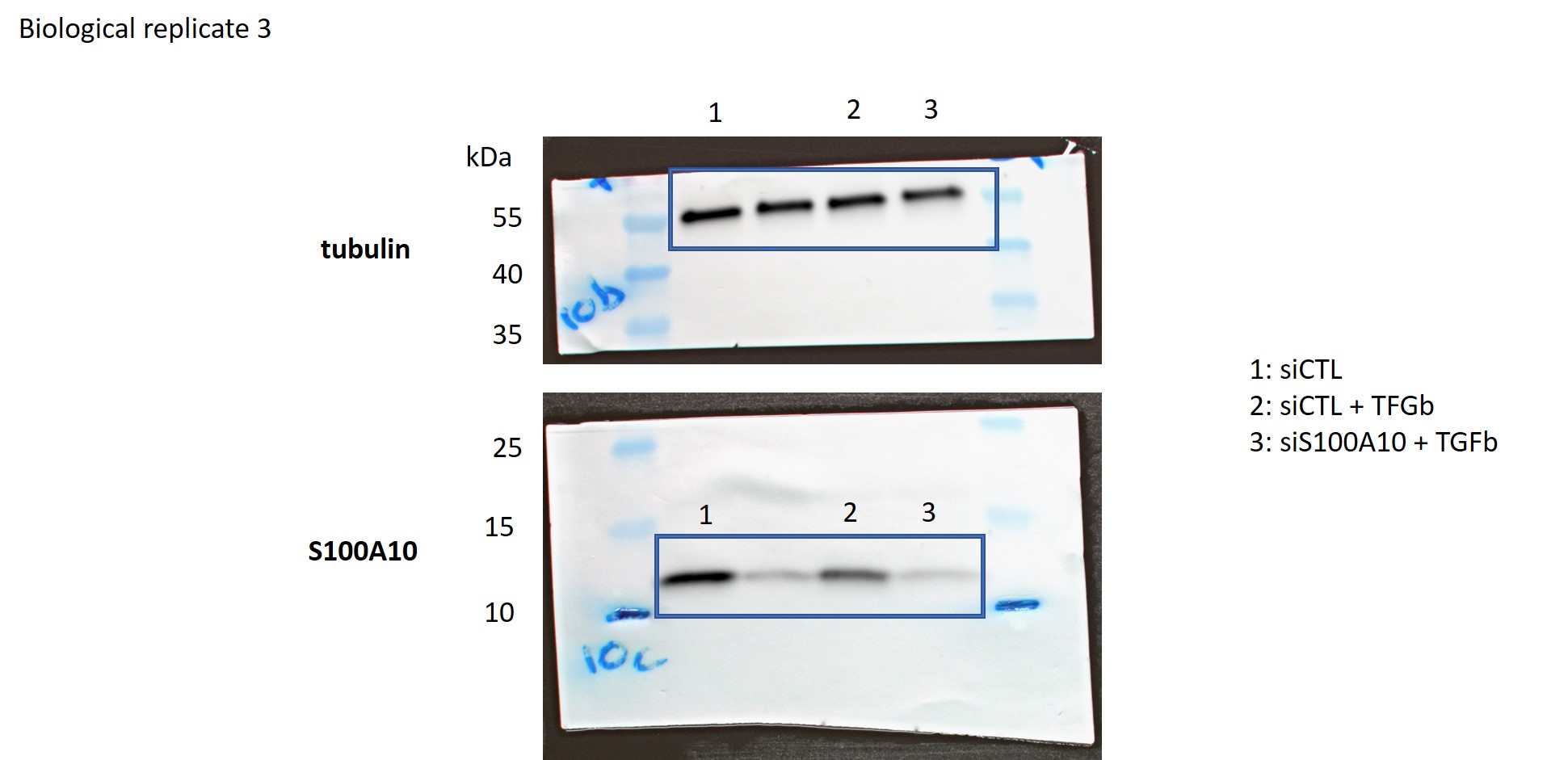

Supplement: Supplementary file 3 — Source data Fig. 1 [file 44321_2026_464_MOESM3_ESM.zip › Figure 1/1C/Replicate 3 - S100A10.jpg]

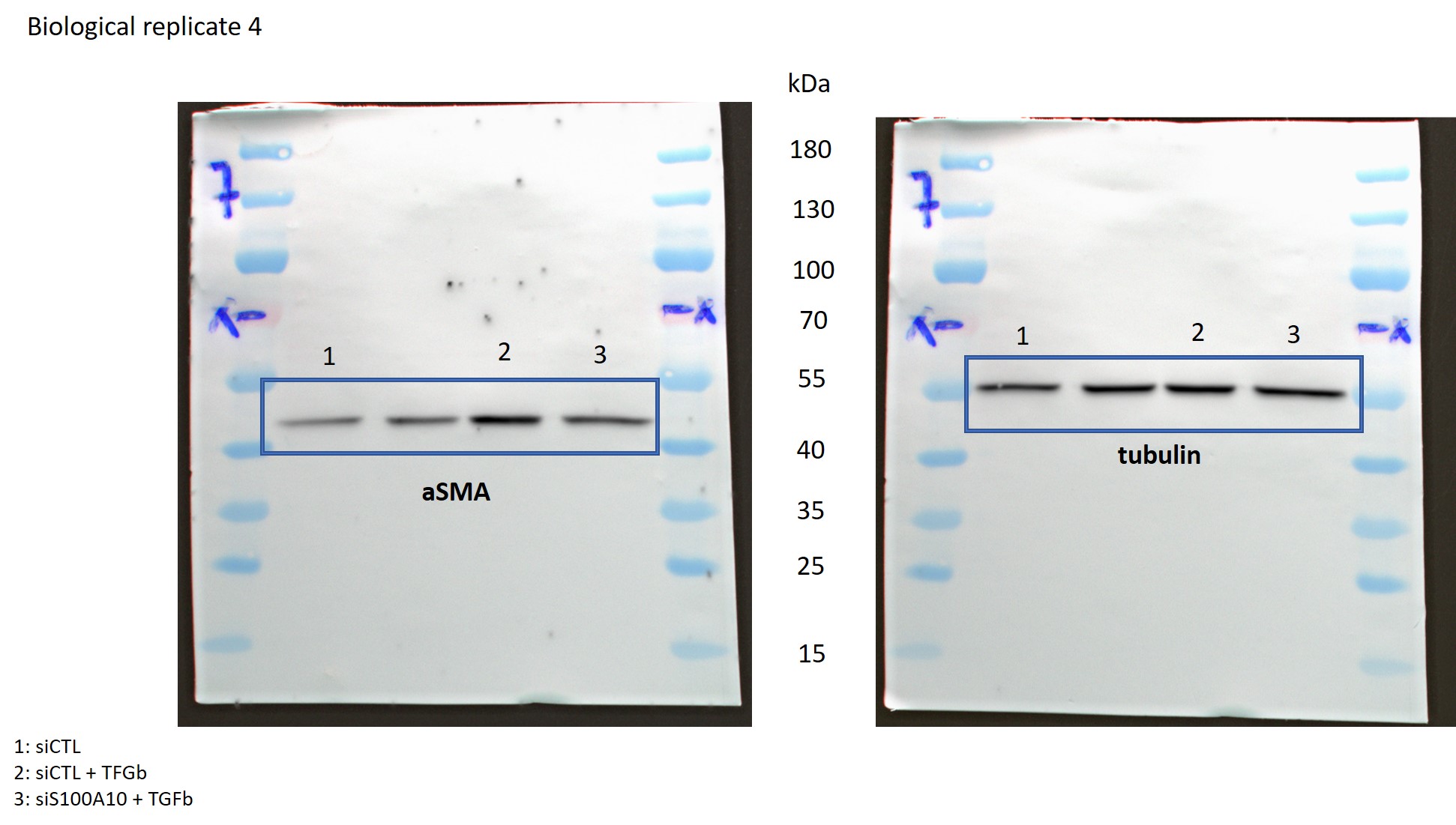

Supplement: Supplementary file 3 — Source data Fig. 1 [file 44321_2026_464_MOESM3_ESM.zip › Figure 1/1C/Replicate 4 - aSMA.jpg]

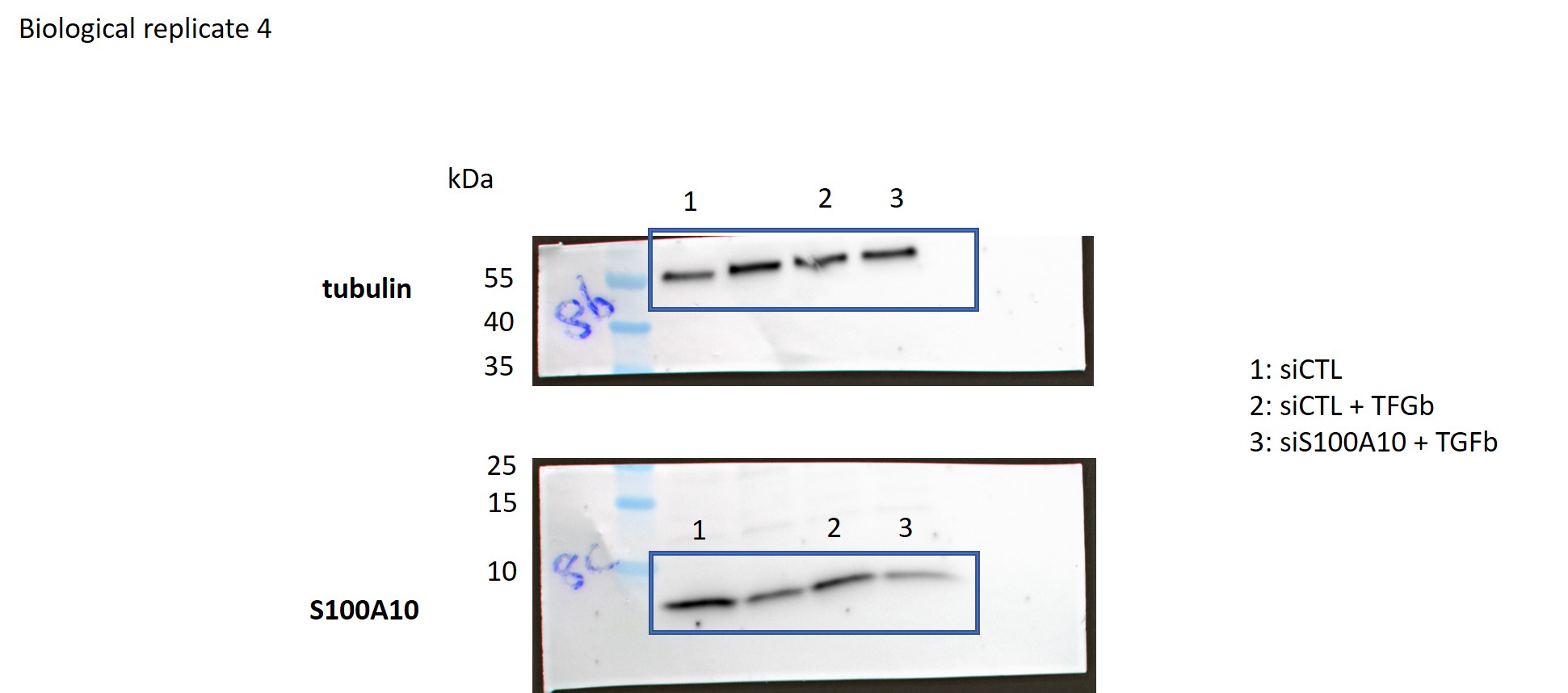

Supplement: Supplementary file 3 — Source data Fig. 1 [file 44321_2026_464_MOESM3_ESM.zip › Figure 1/1C/Replicate 4 - S100A10.jpg]

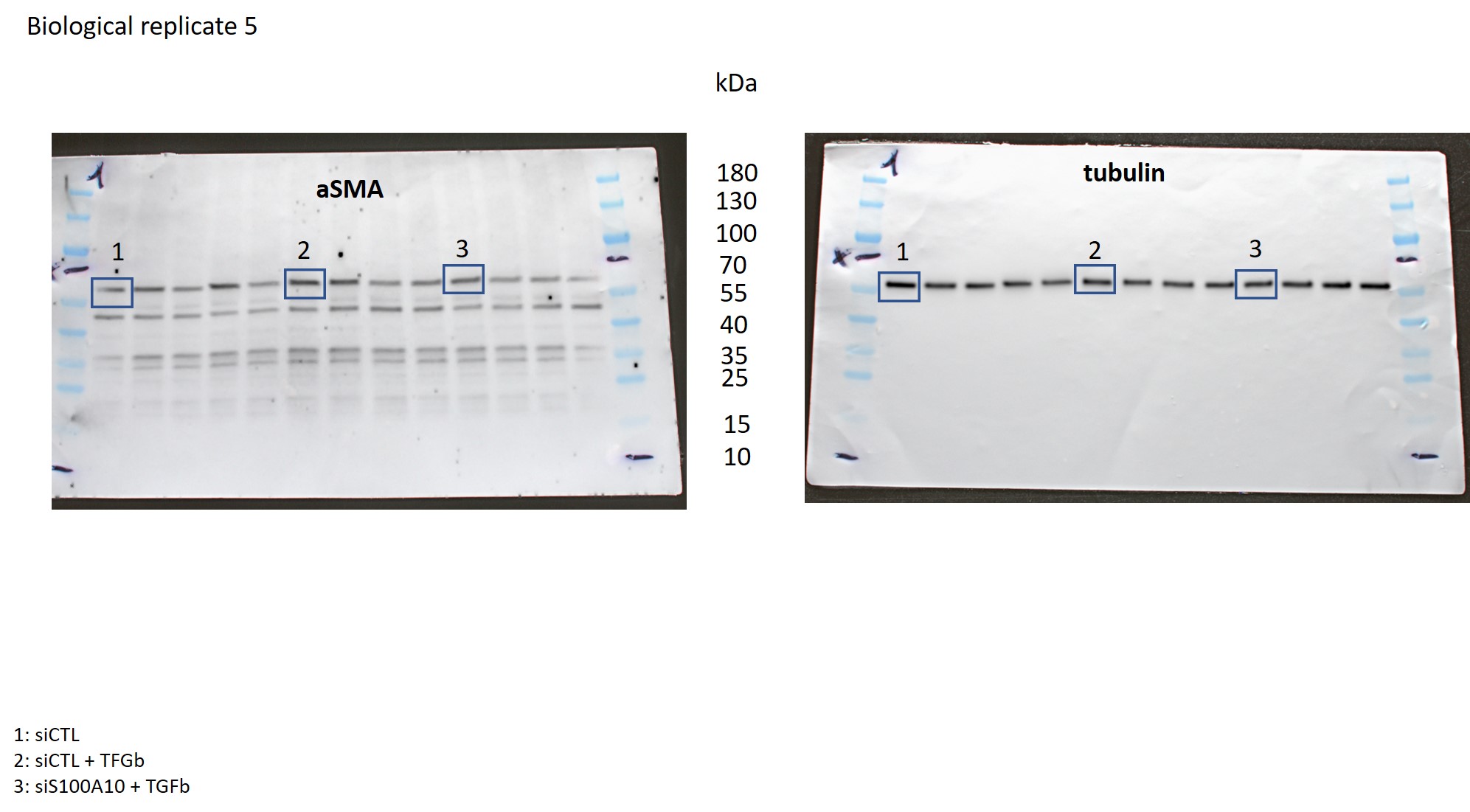

Supplement: Supplementary file 3 — Source data Fig. 1 [file 44321_2026_464_MOESM3_ESM.zip › Figure 1/1C/Replicate 5 - aSMA.jpg]

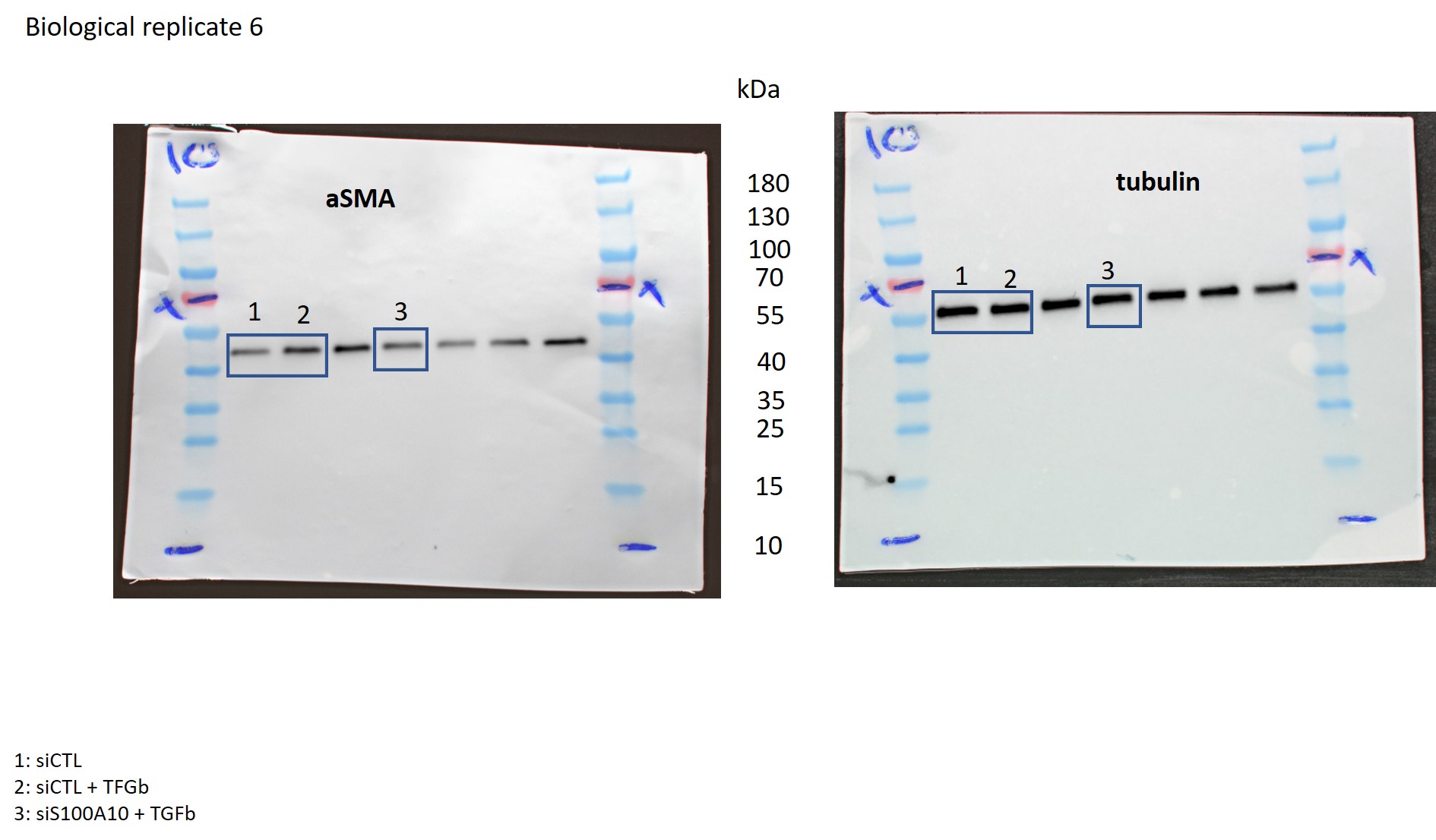

Supplement: Supplementary file 3 — Source data Fig. 1 [file 44321_2026_464_MOESM3_ESM.zip › Figure 1/1C/Replicate 6 - aSMA.jpg]

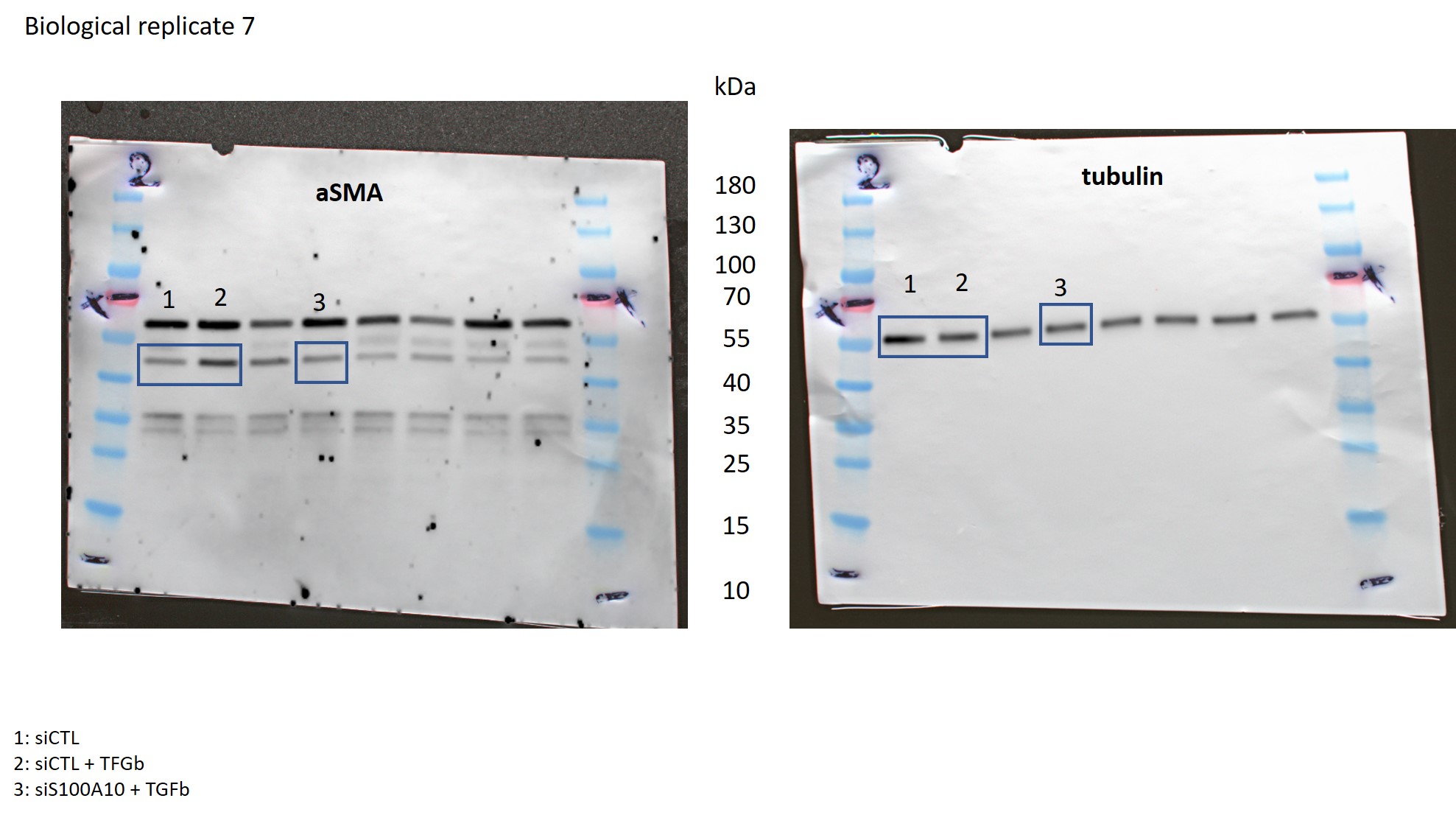

Supplement: Supplementary file 3 — Source data Fig. 1 [file 44321_2026_464_MOESM3_ESM.zip › Figure 1/1C/Replicate 7 - aSMA.jpg]

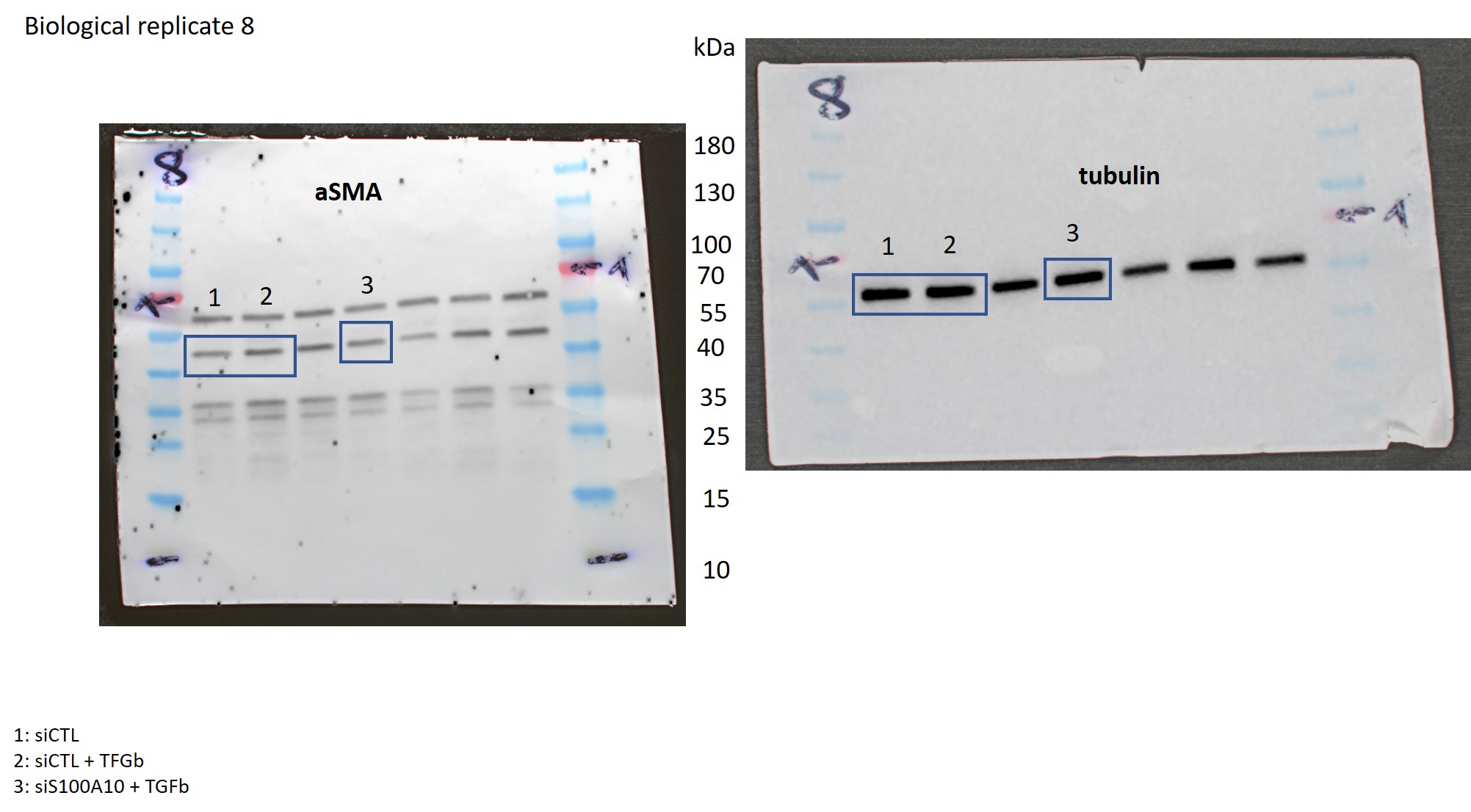

Supplement: Supplementary file 3 — Source data Fig. 1 [file 44321_2026_464_MOESM3_ESM.zip › Figure 1/1C/Replicate 8 - aSMA.jpg]

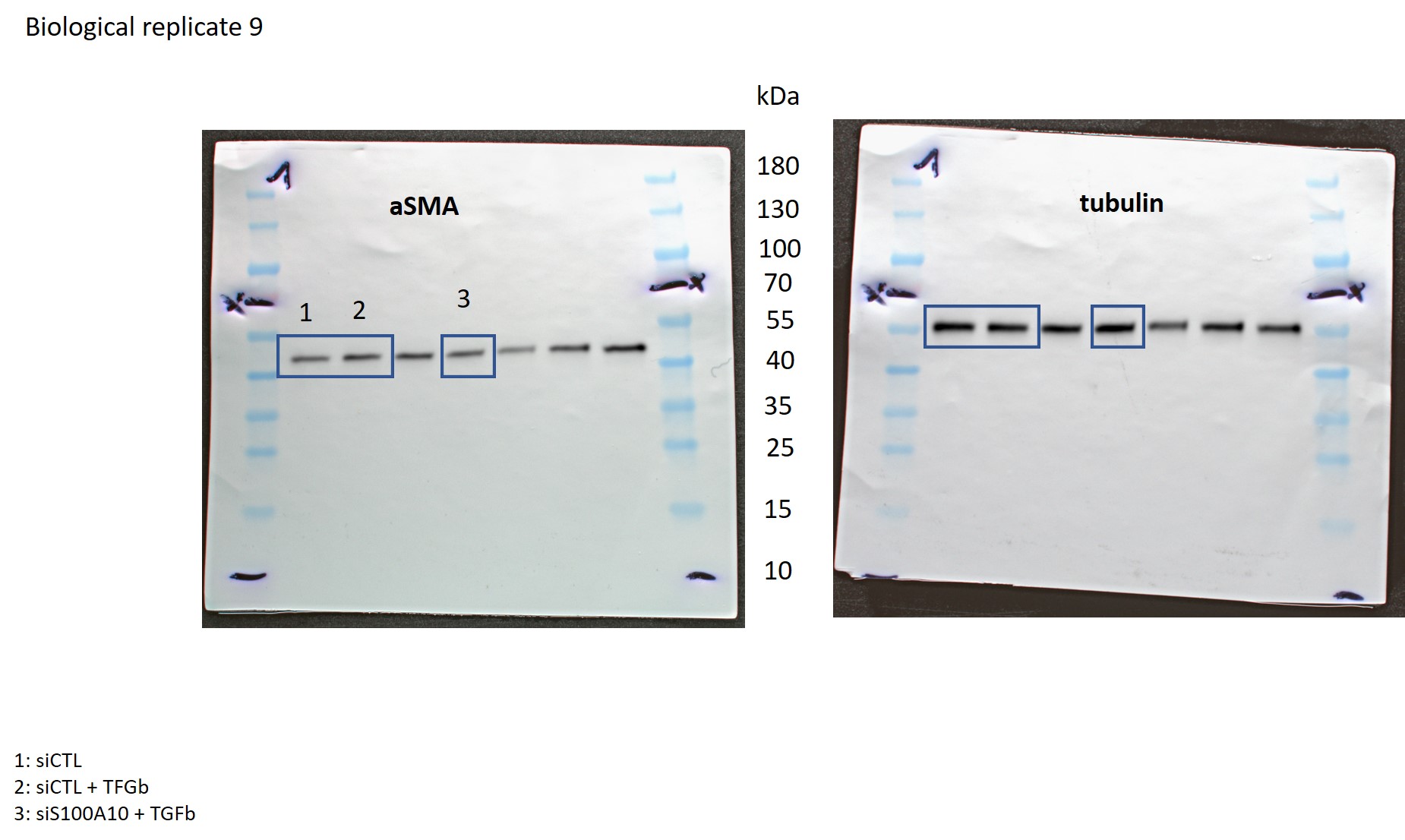

Supplement: Supplementary file 3 — Source data Fig. 1 [file 44321_2026_464_MOESM3_ESM.zip › Figure 1/1C/Replicate 9 - aSMA.jpg]

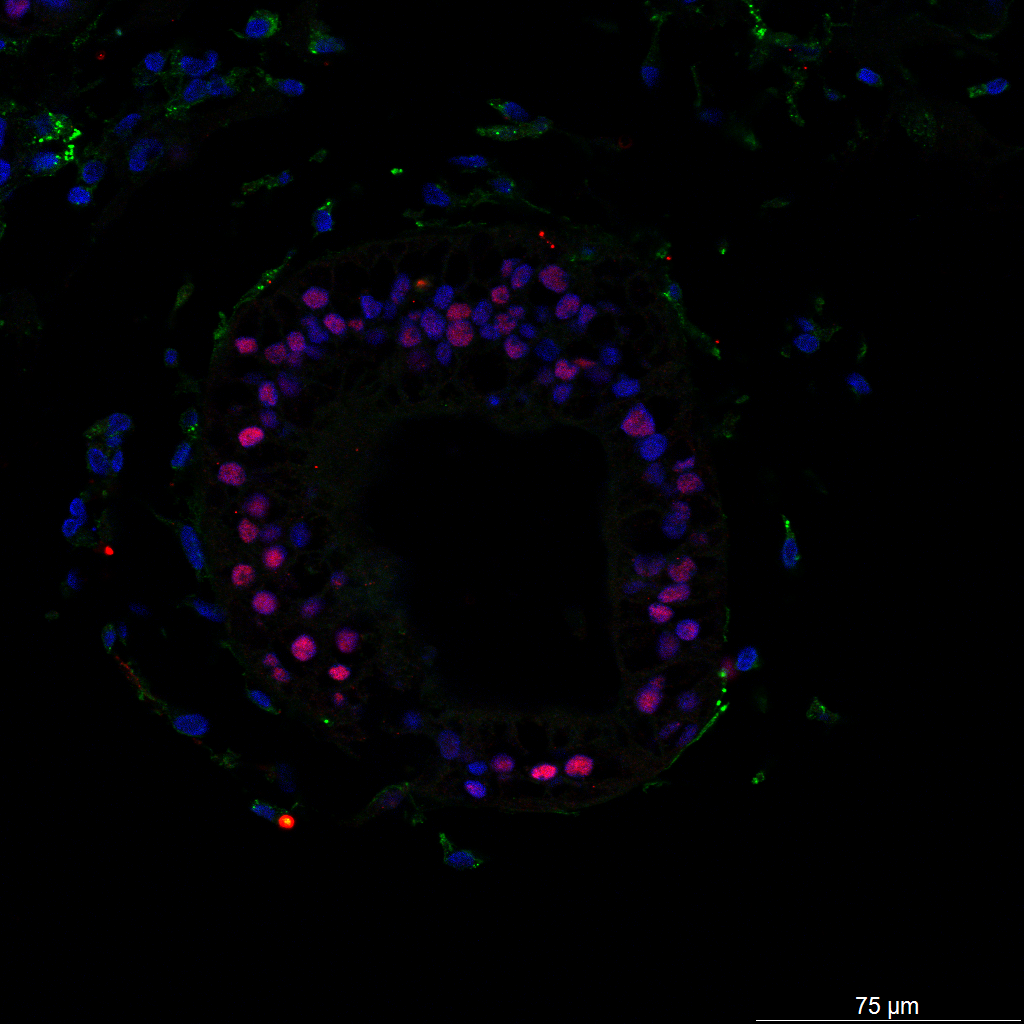

Supplement: Supplementary file 4 — Source data Fig. 2 [file 44321_2026_464_MOESM4_ESM.zip › Figure 2/Figure 2C/Figure_2C_Image_Merged.tif]

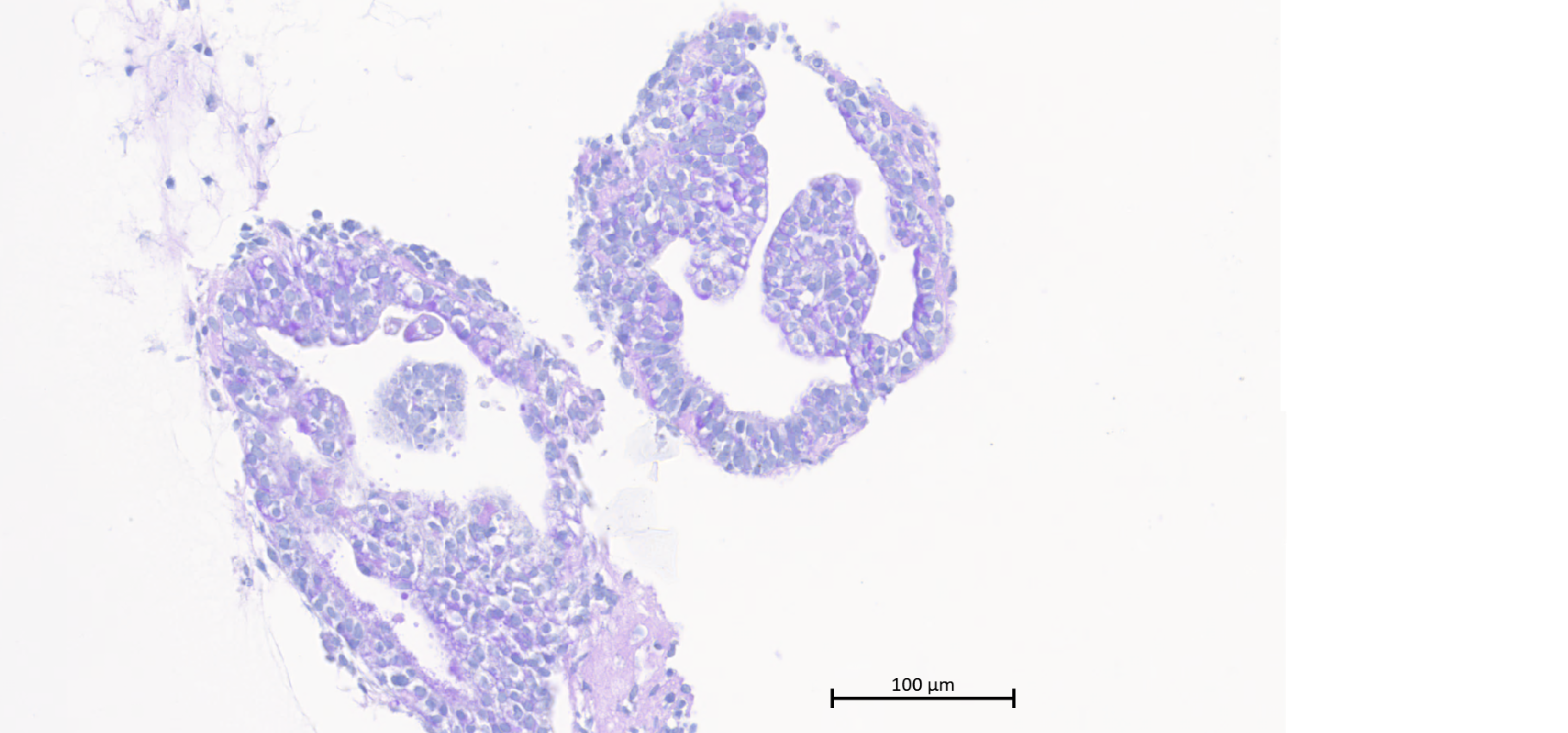

Supplement: Supplementary file 4 — Source data Fig. 2 [file 44321_2026_464_MOESM4_ESM.zip › Figure 2/Figure 2F/Figure_2F_MT_Picture_HLOs.png]

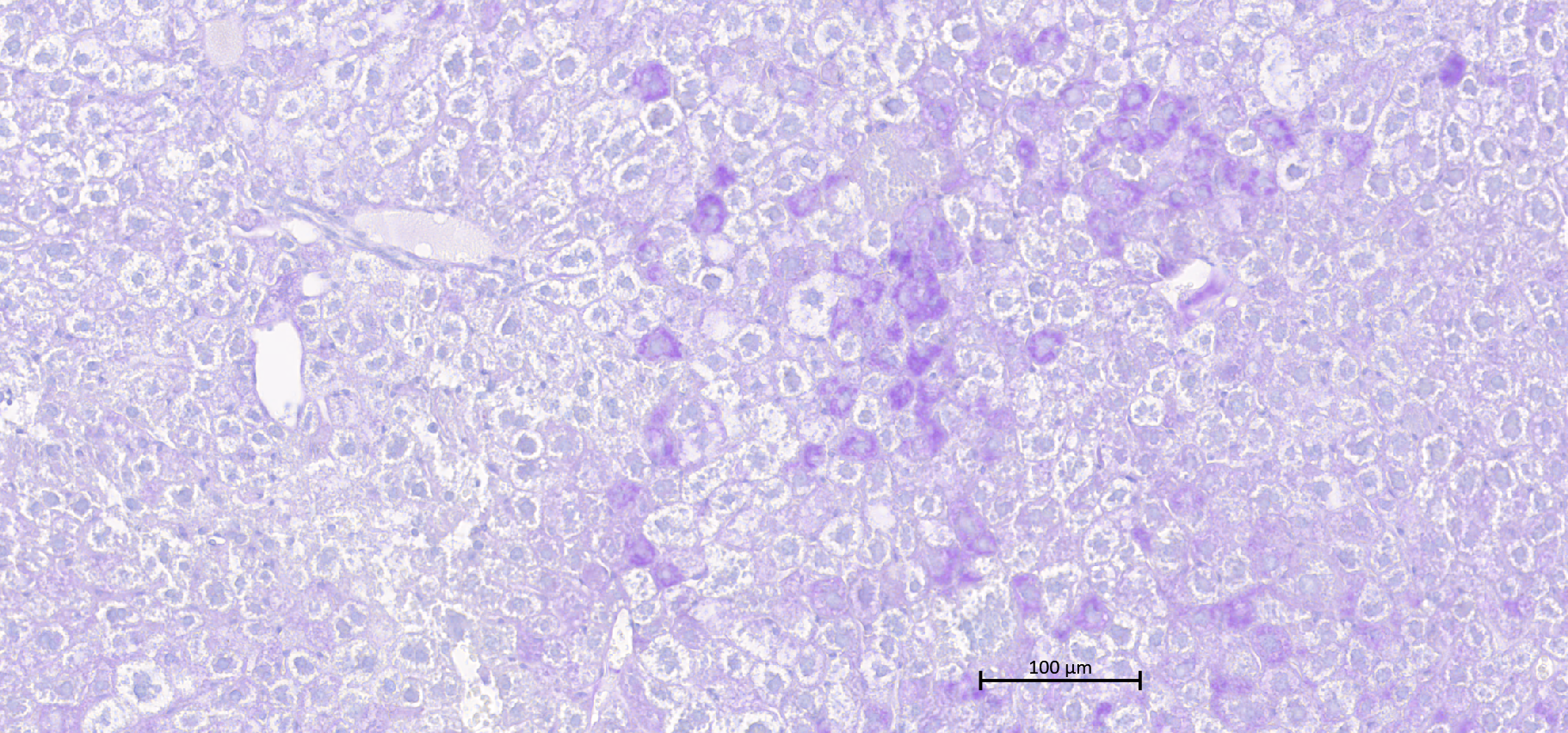

Supplement: Supplementary file 4 — Source data Fig. 2 [file 44321_2026_464_MOESM4_ESM.zip › Figure 2/Figure 2F/Figure_2F_MT_Picture_Liver.png]

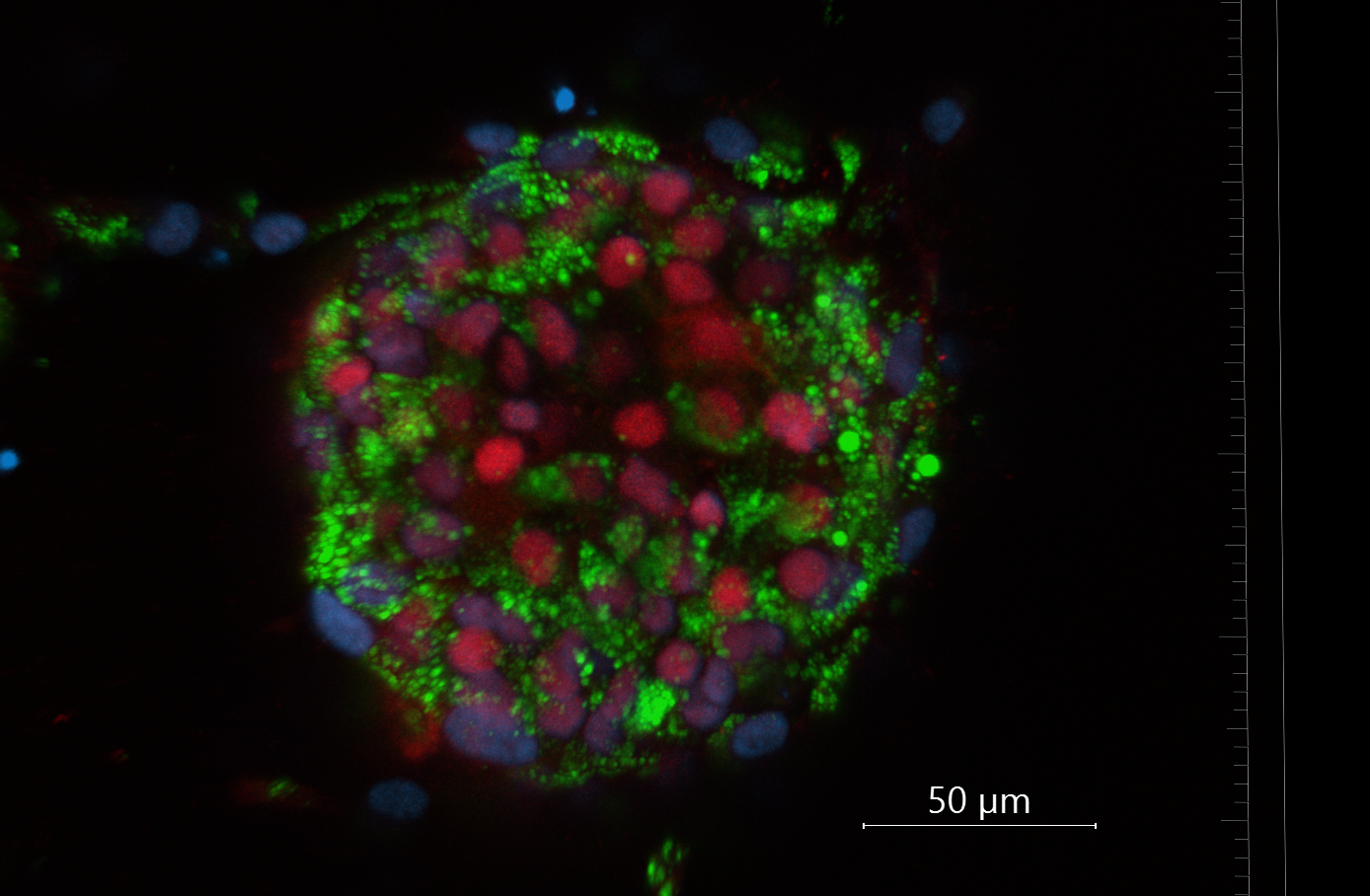

Supplement: Supplementary file 5 — Source data Fig. 3 [file 44321_2026_464_MOESM5_ESM.zip › Figure 3/Figure 3B/Figure_3B_Picture_MT_OAPA400.png]

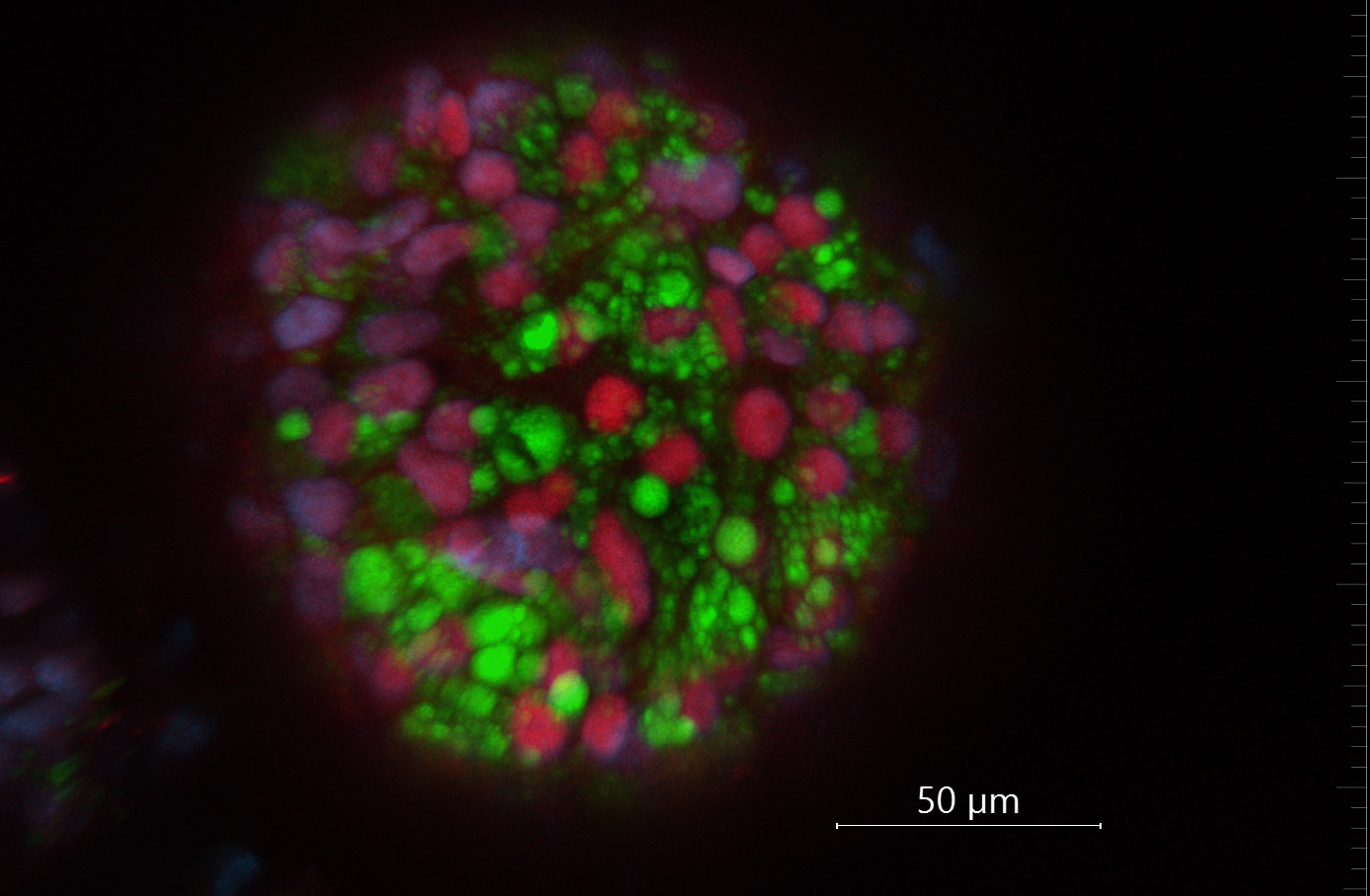

Supplement: Supplementary file 5 — Source data Fig. 3 [file 44321_2026_464_MOESM5_ESM.zip › Figure 3/Figure 3B/Figure_3B_Picture_MT_OAPA600.png]

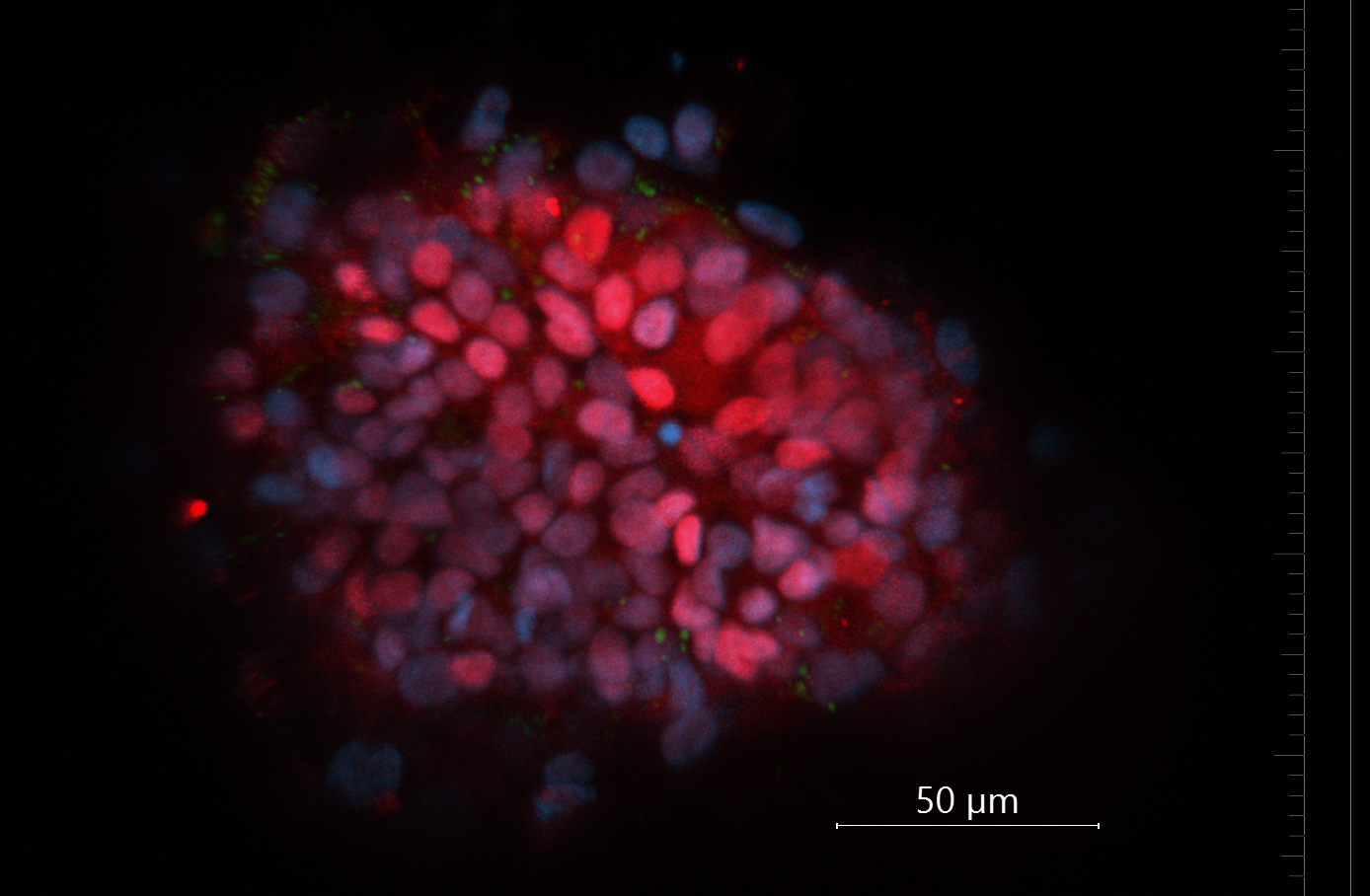

Supplement: Supplementary file 5 — Source data Fig. 3 [file 44321_2026_464_MOESM5_ESM.zip › Figure 3/Figure 3B/Figure_3B_Picture_MT_Vehicle.png]

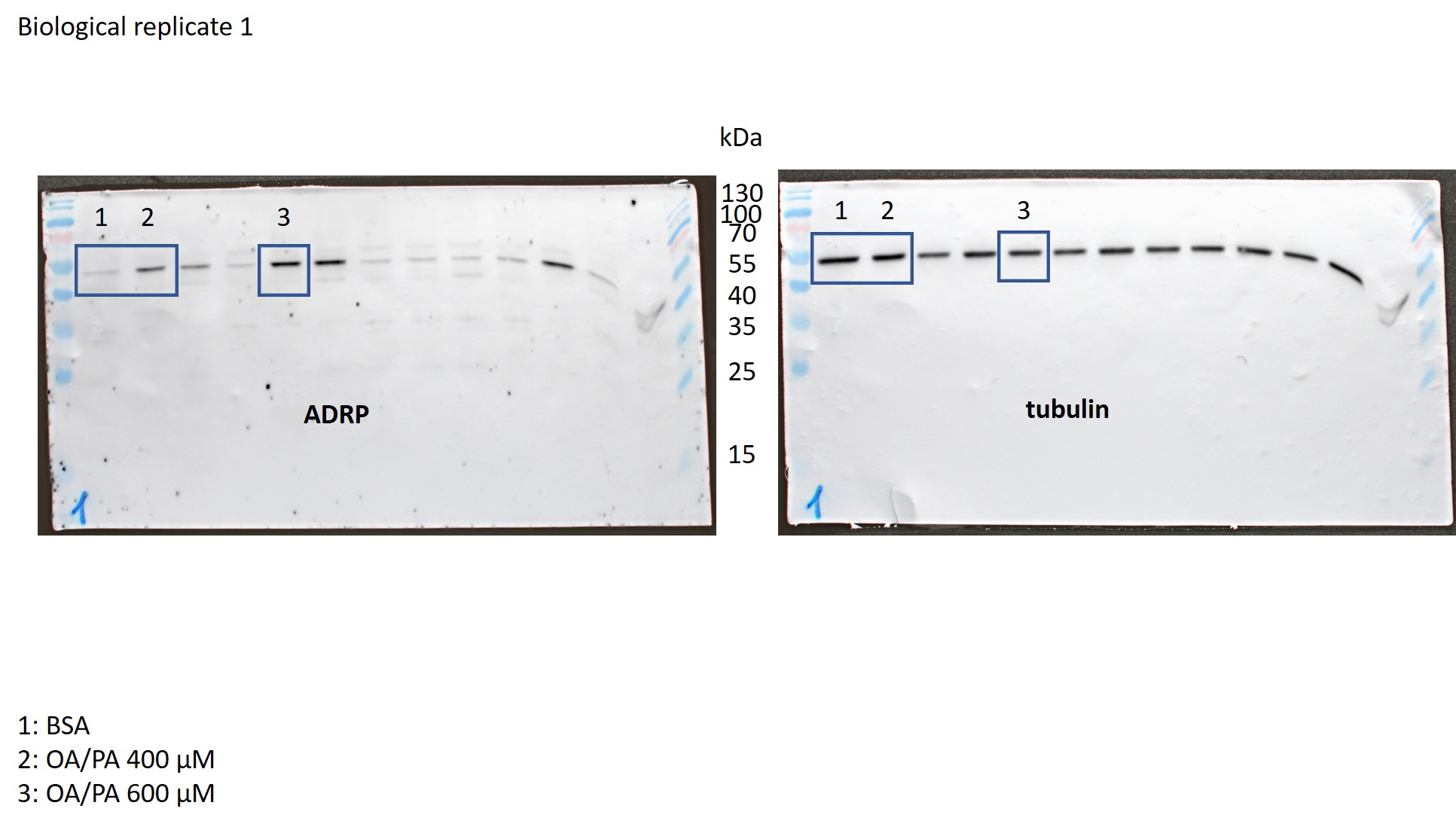

Supplement: Supplementary file 5 — Source data Fig. 3 [file 44321_2026_464_MOESM5_ESM.zip › Figure 3/Figure 3F/Replicate 1 - ADRP.jpg]

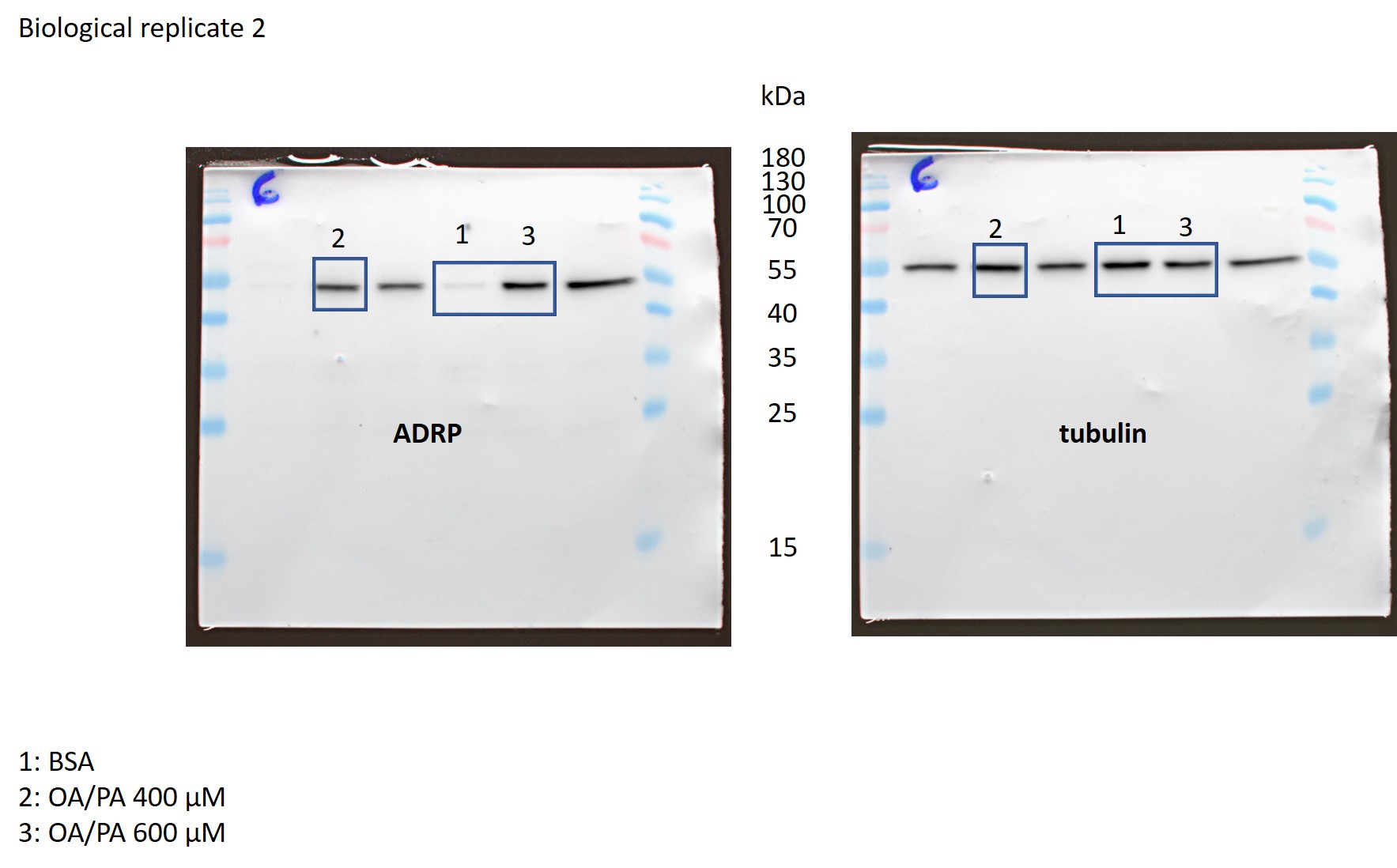

Supplement: Supplementary file 5 — Source data Fig. 3 [file 44321_2026_464_MOESM5_ESM.zip › Figure 3/Figure 3F/Replicate 2 - ADRP.jpg]

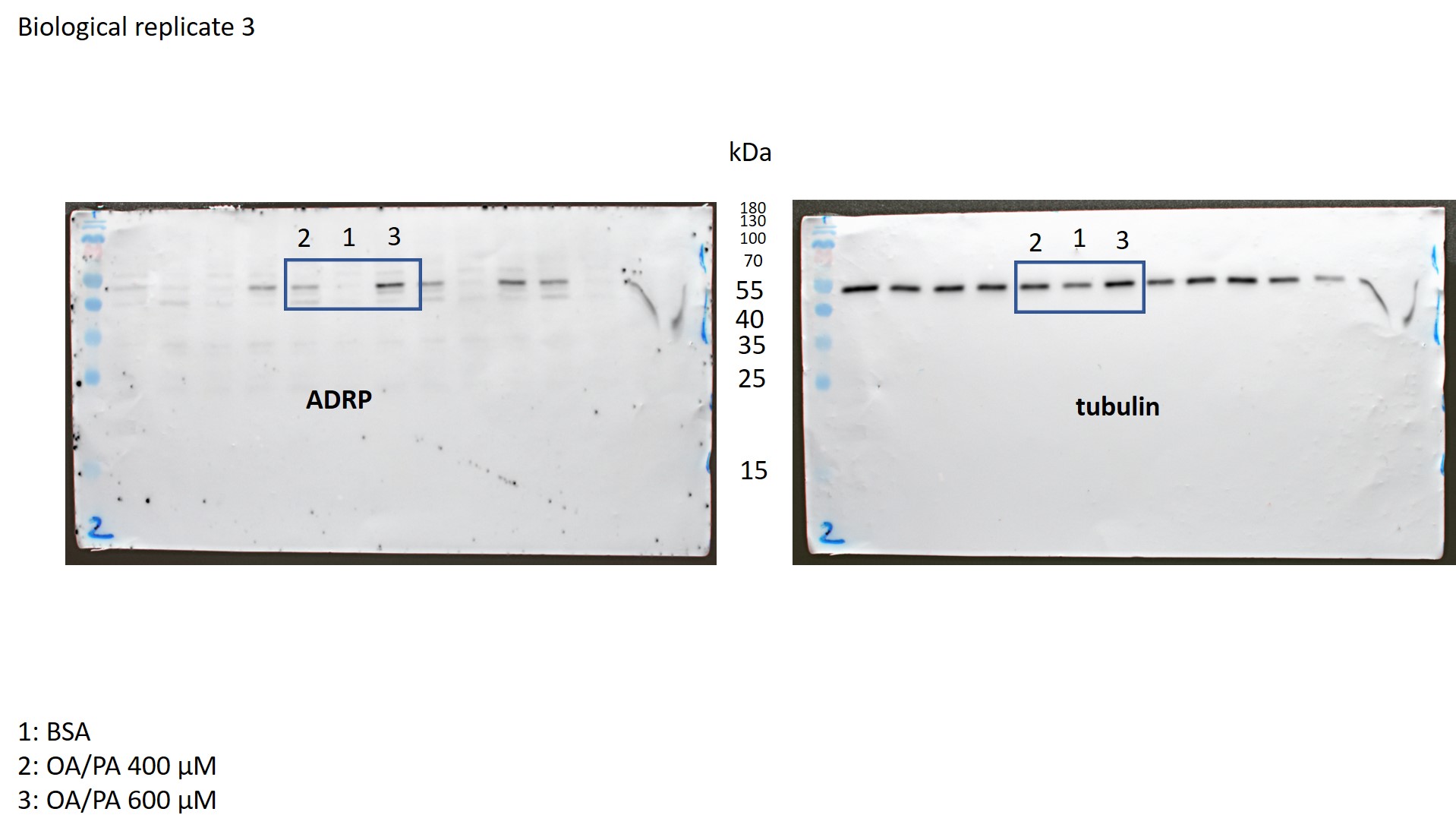

Supplement: Supplementary file 5 — Source data Fig. 3 [file 44321_2026_464_MOESM5_ESM.zip › Figure 3/Figure 3F/Replicate 3 - ADRP.jpg]

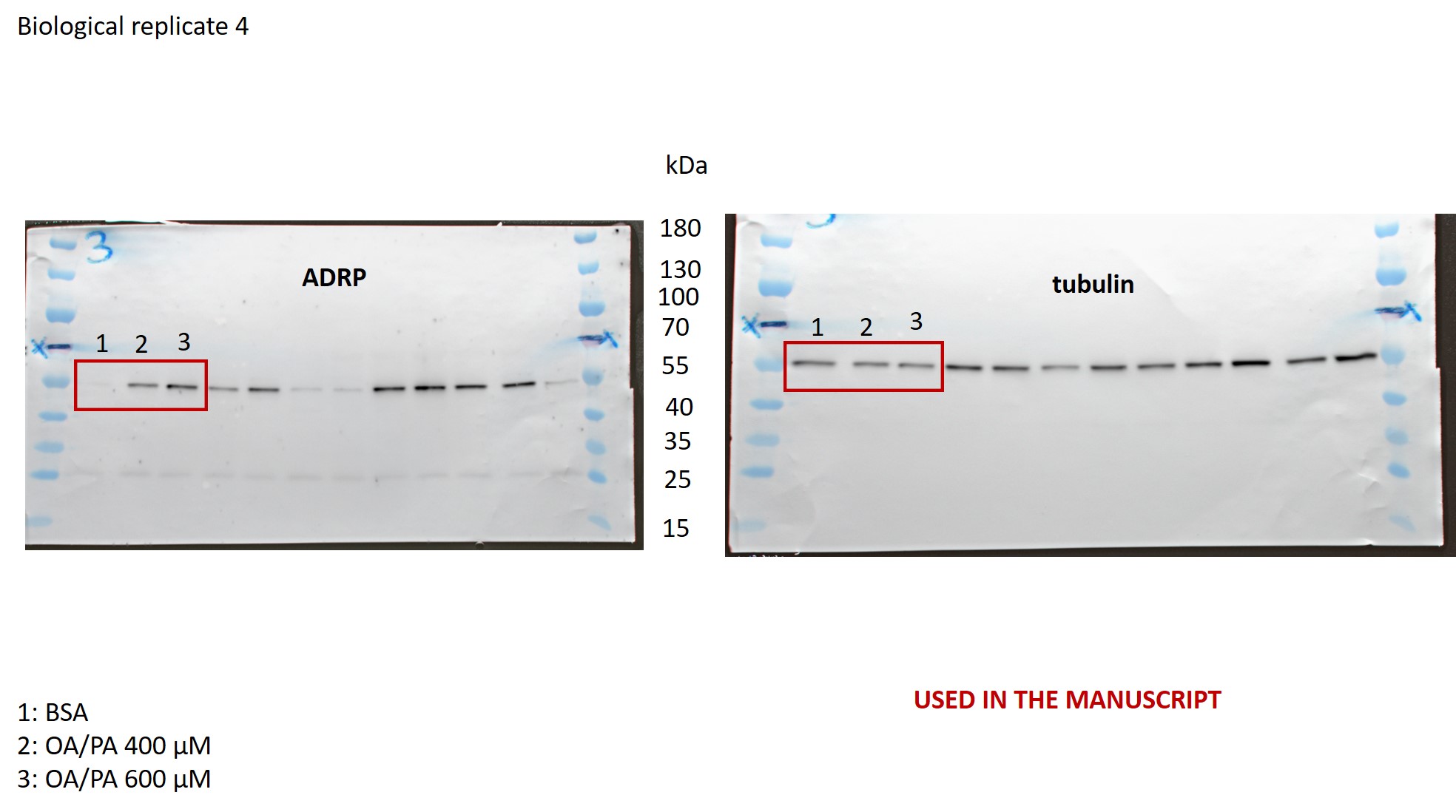

Supplement: Supplementary file 5 — Source data Fig. 3 [file 44321_2026_464_MOESM5_ESM.zip › Figure 3/Figure 3F/Replicate 4 - ADRP.jpg]

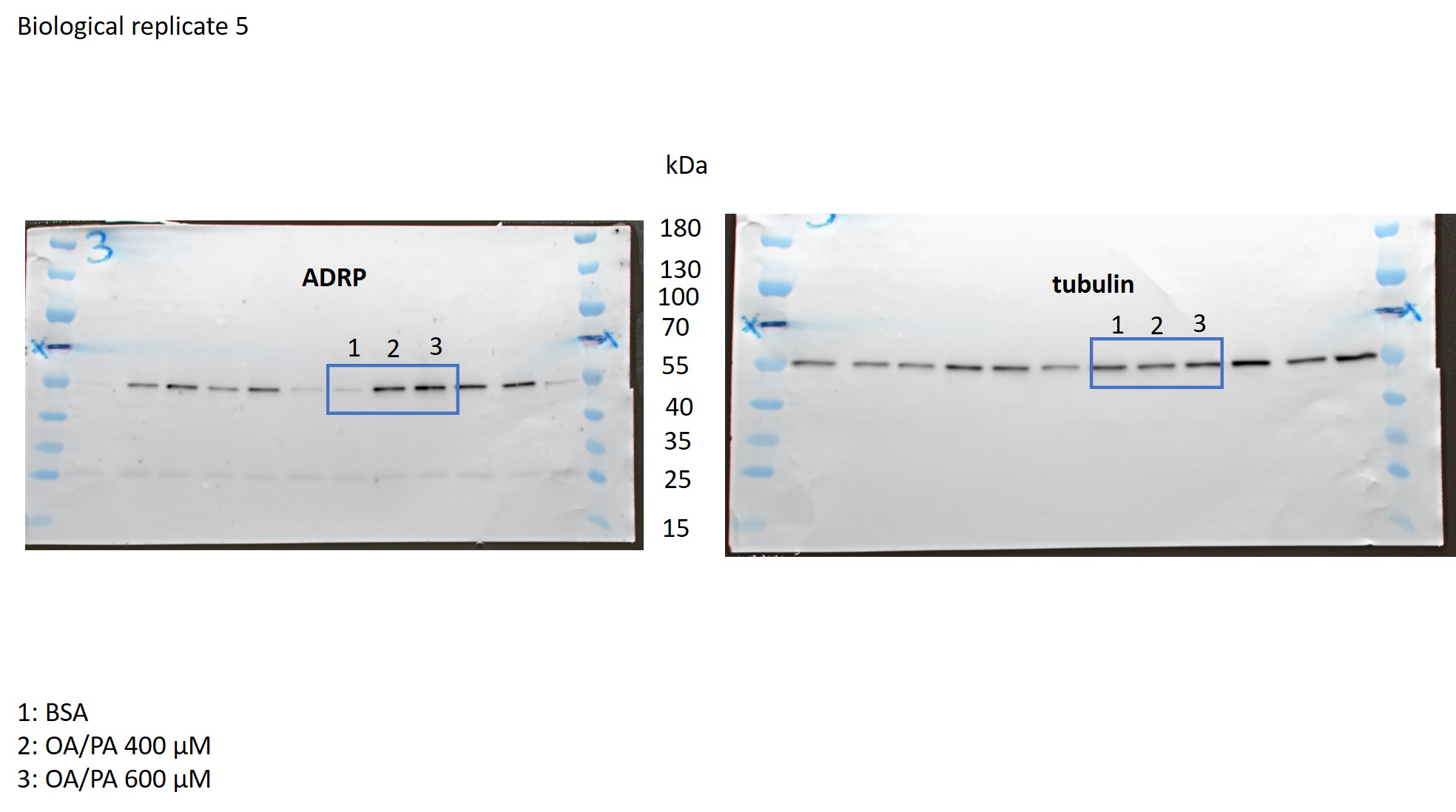

Supplement: Supplementary file 5 — Source data Fig. 3 [file 44321_2026_464_MOESM5_ESM.zip › Figure 3/Figure 3F/Replicate 5 - ADRP.jpg]

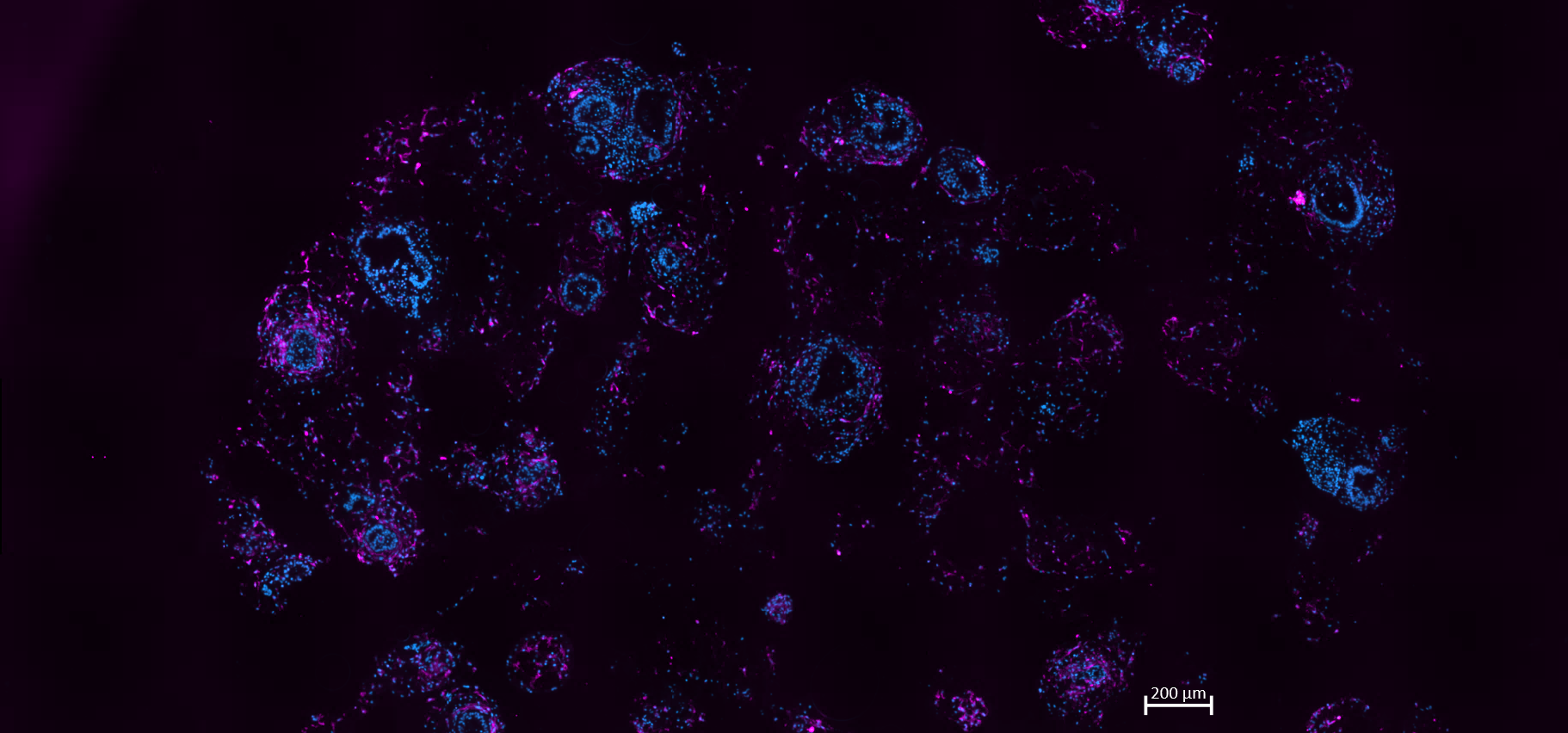

Supplement: Supplementary file 5 — Source data Fig. 3 [file 44321_2026_464_MOESM5_ESM.zip › Figure 3/Figure 3H/Figure_3H_MT_aSMA_Staining_HLOs_OAPA_TGFb1.png]

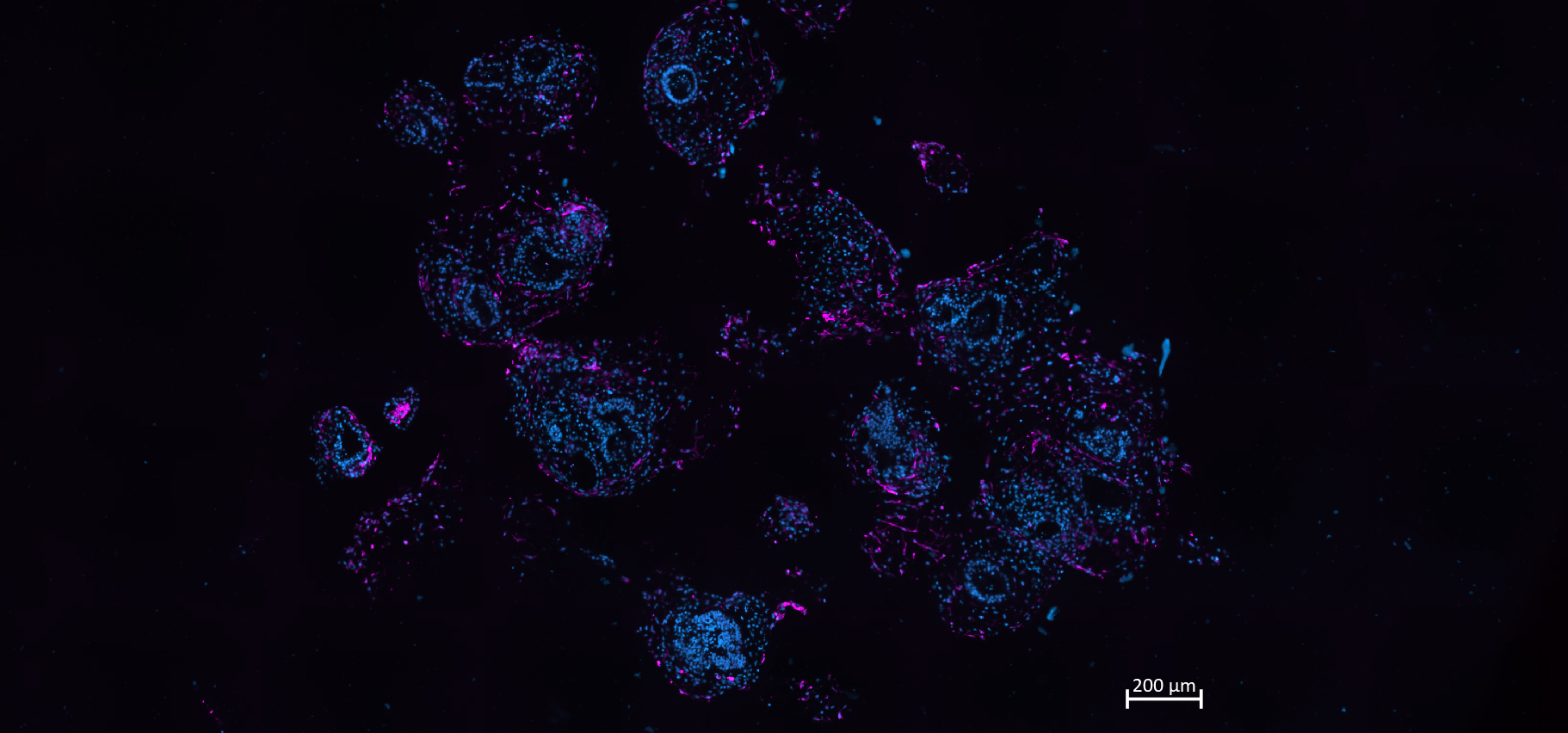

Supplement: Supplementary file 5 — Source data Fig. 3 [file 44321_2026_464_MOESM5_ESM.zip › Figure 3/Figure 3H/Figure_3H_MT_aSMA_Staining_HLOs_TGFb1.png]

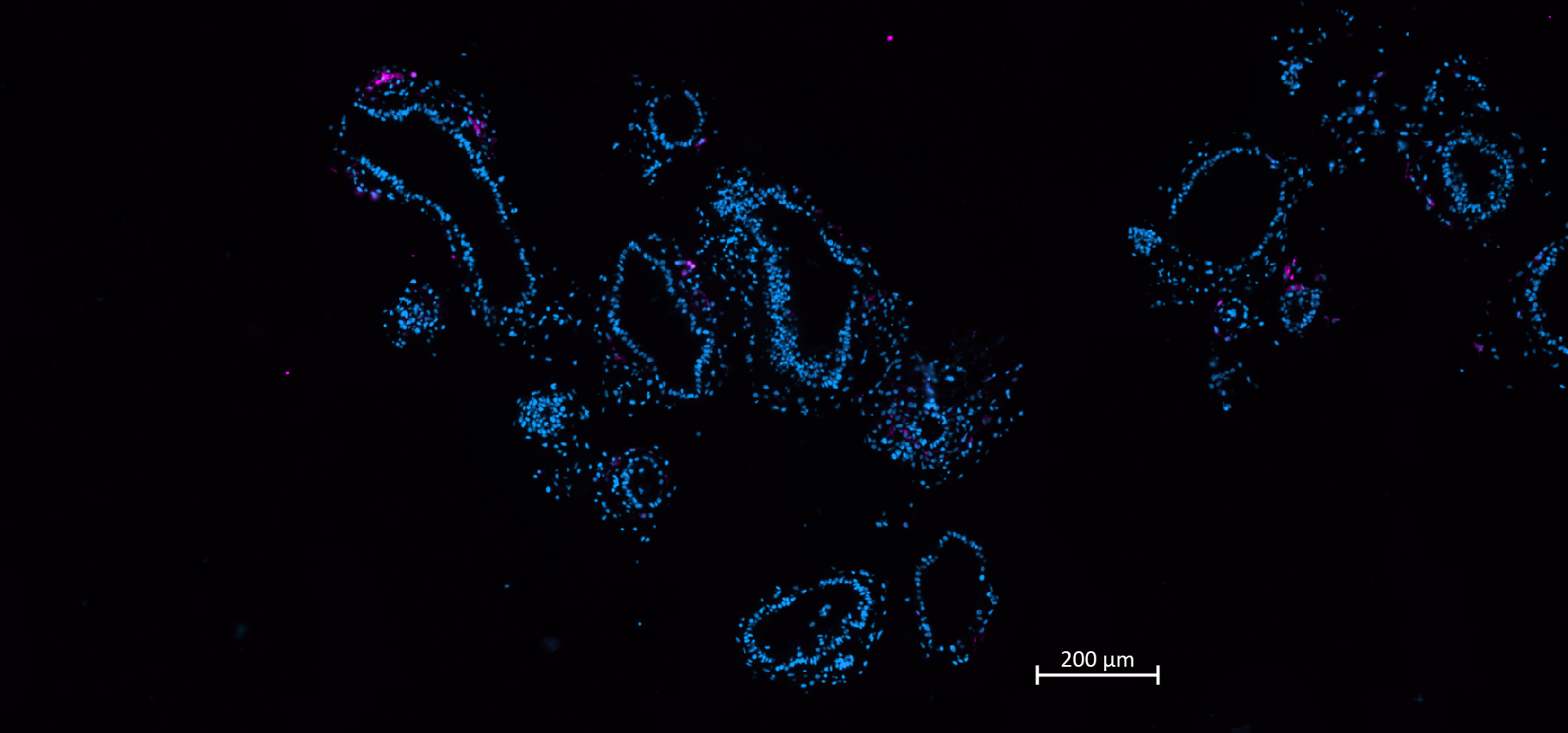

Supplement: Supplementary file 5 — Source data Fig. 3 [file 44321_2026_464_MOESM5_ESM.zip › Figure 3/Figure 3H/Figure_3H_MT_aSMA_Staining_HLOs_VEH.png]

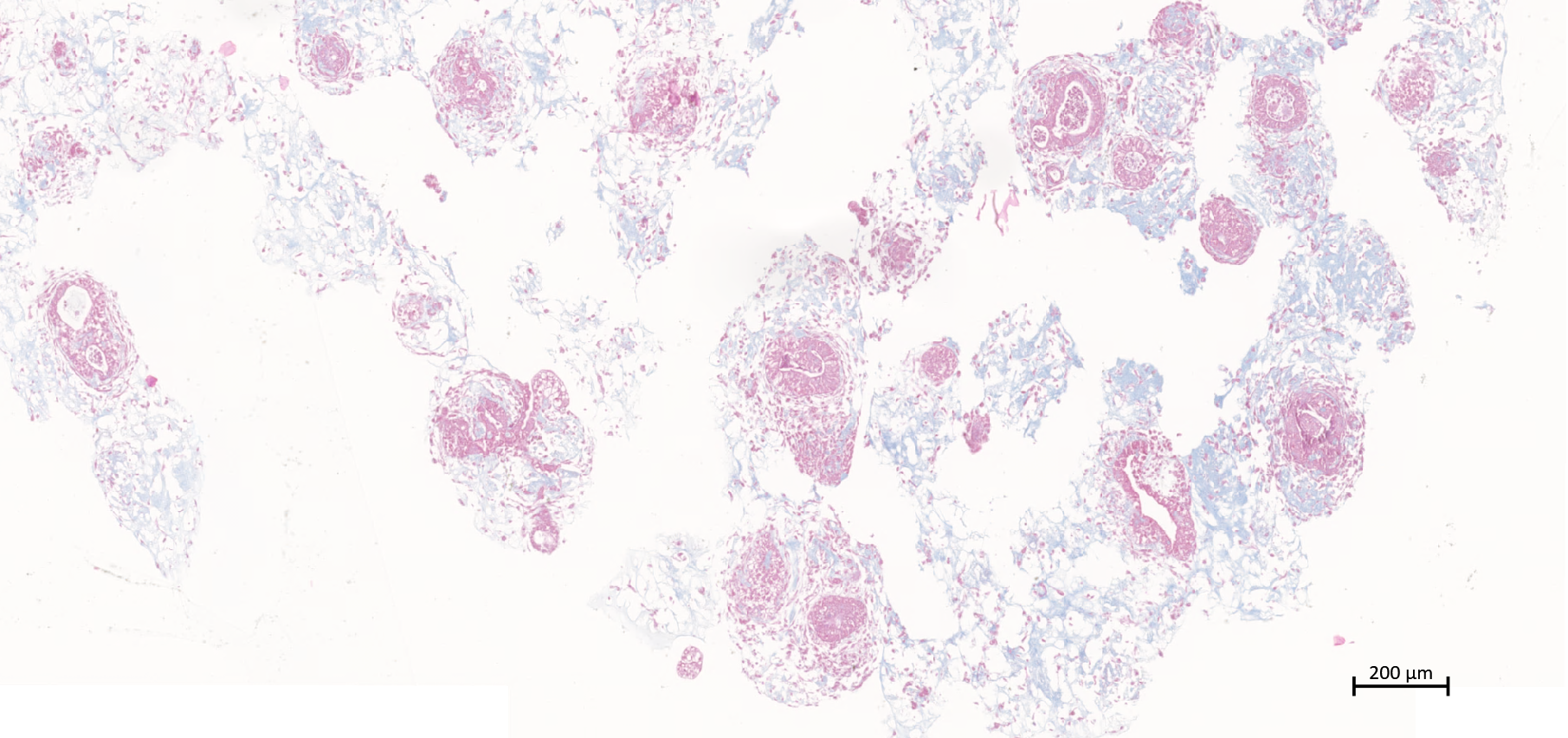

Supplement: Supplementary file 5 — Source data Fig. 3 [file 44321_2026_464_MOESM5_ESM.zip › Figure 3/Figure 3H/Figure_3H_MT_Masson_HLOs_OAPA_TGFb1.png]

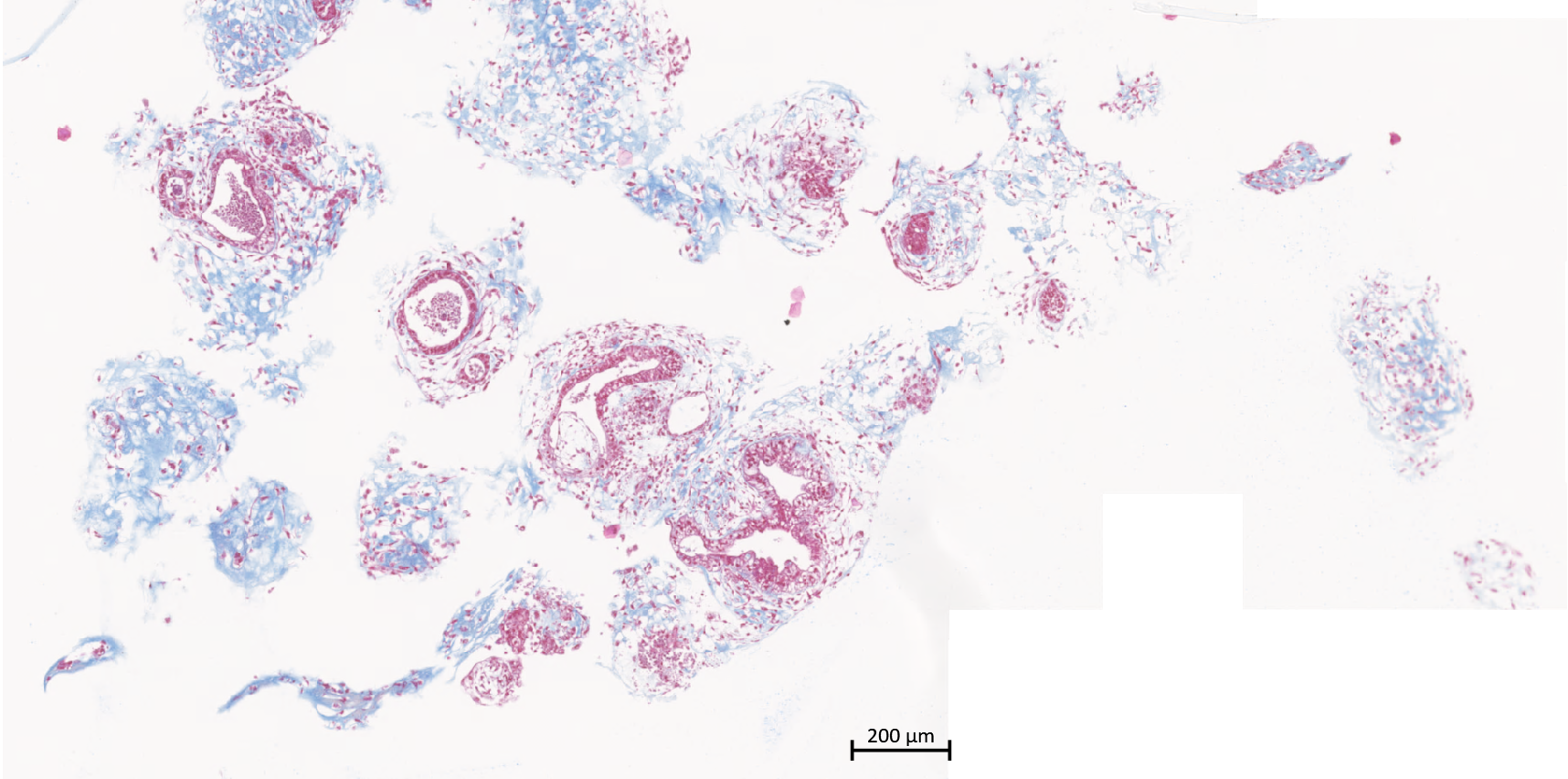

Supplement: Supplementary file 5 — Source data Fig. 3 [file 44321_2026_464_MOESM5_ESM.zip › Figure 3/Figure 3H/Figure_3H_MT_Masson_HLOs_TGFb1.png]

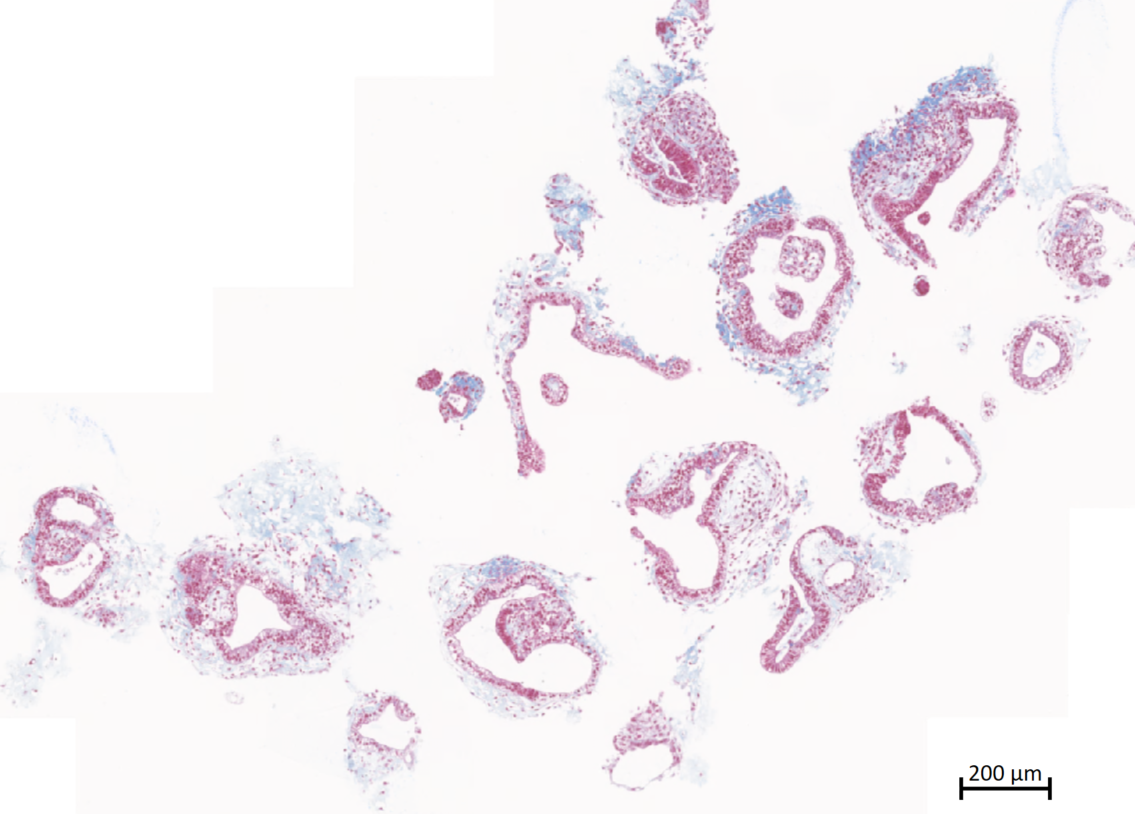

Supplement: Supplementary file 5 — Source data Fig. 3 [file 44321_2026_464_MOESM5_ESM.zip › Figure 3/Figure 3H/Figure_3H_MT_Masson_HLOs_VEH.png]

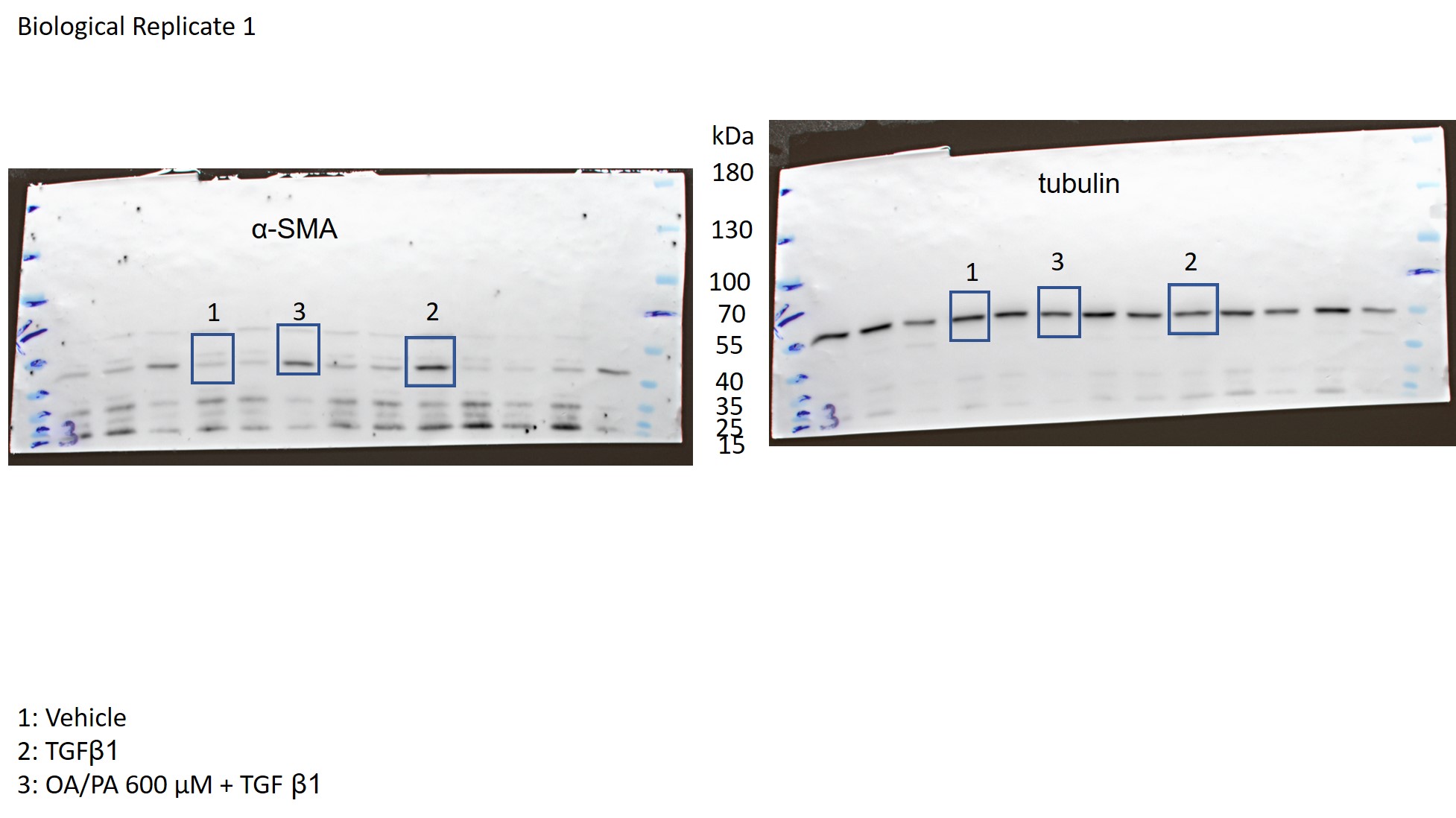

Supplement: Supplementary file 5 — Source data Fig. 3 [file 44321_2026_464_MOESM5_ESM.zip › Figure 3/Figure 3I/Replicate 1 - aSMA.jpg]

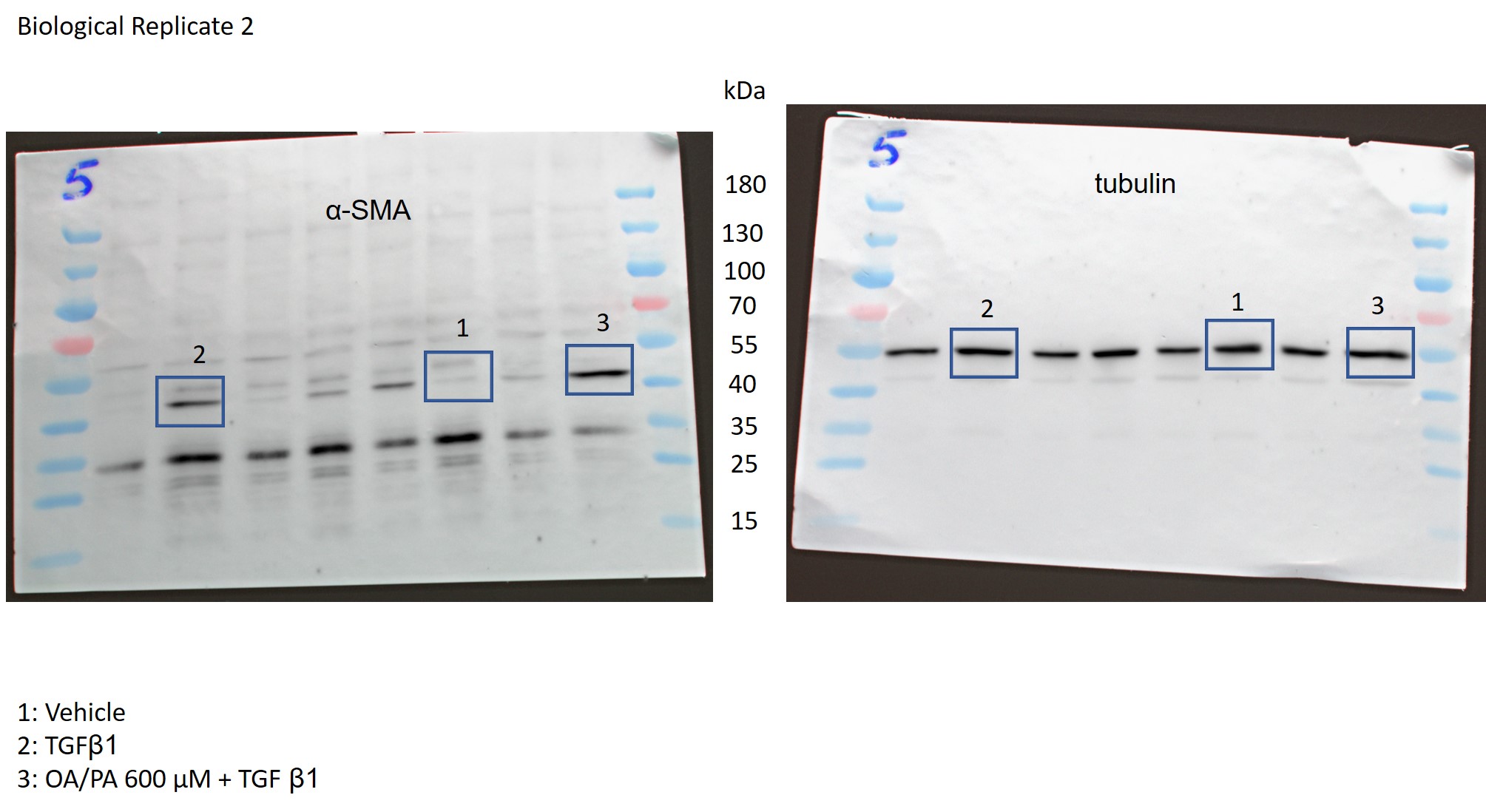

Supplement: Supplementary file 5 — Source data Fig. 3 [file 44321_2026_464_MOESM5_ESM.zip › Figure 3/Figure 3I/Replicate 2 - aSMA.jpg]

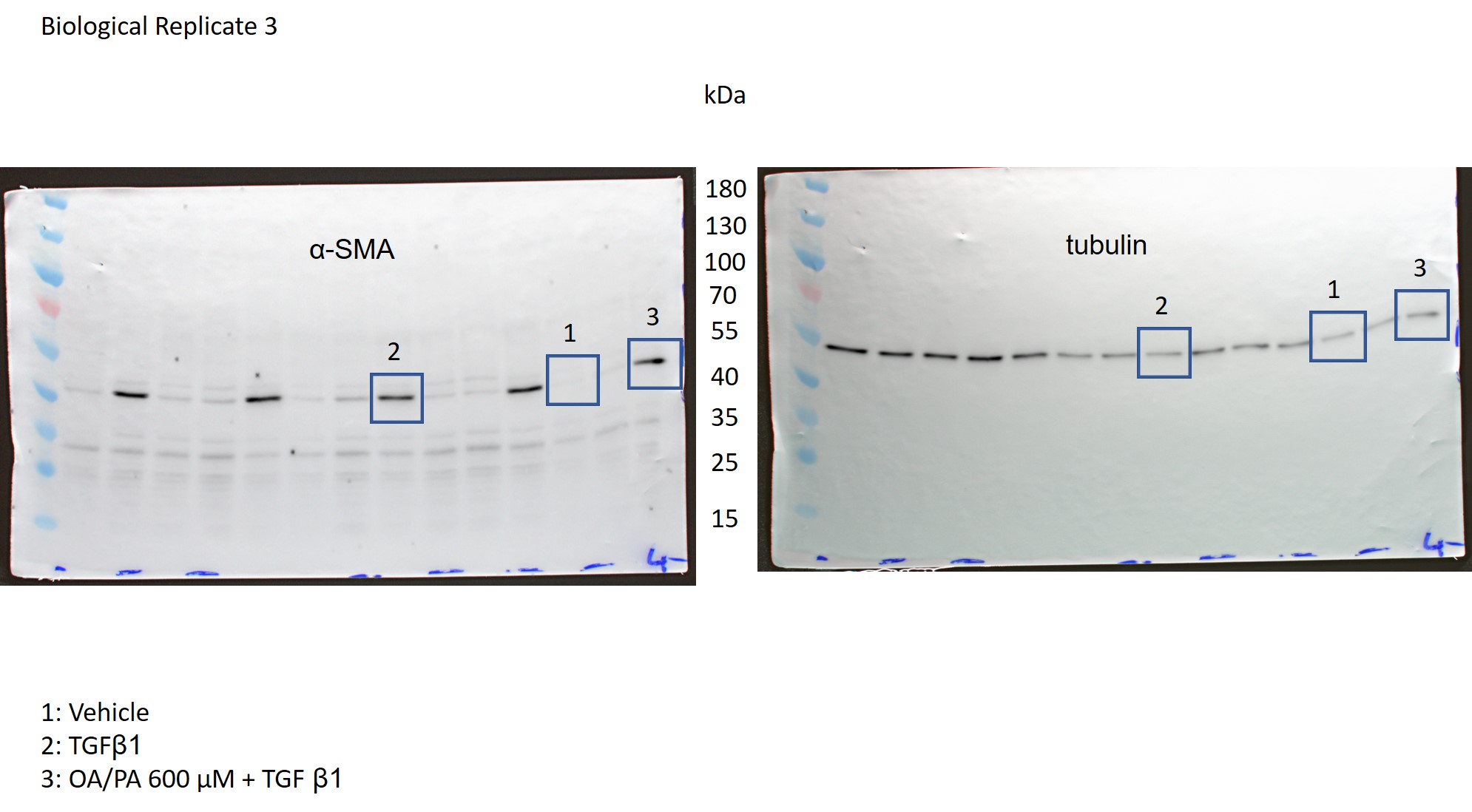

Supplement: Supplementary file 5 — Source data Fig. 3 [file 44321_2026_464_MOESM5_ESM.zip › Figure 3/Figure 3I/Replicate 3 - aSMA.jpg]

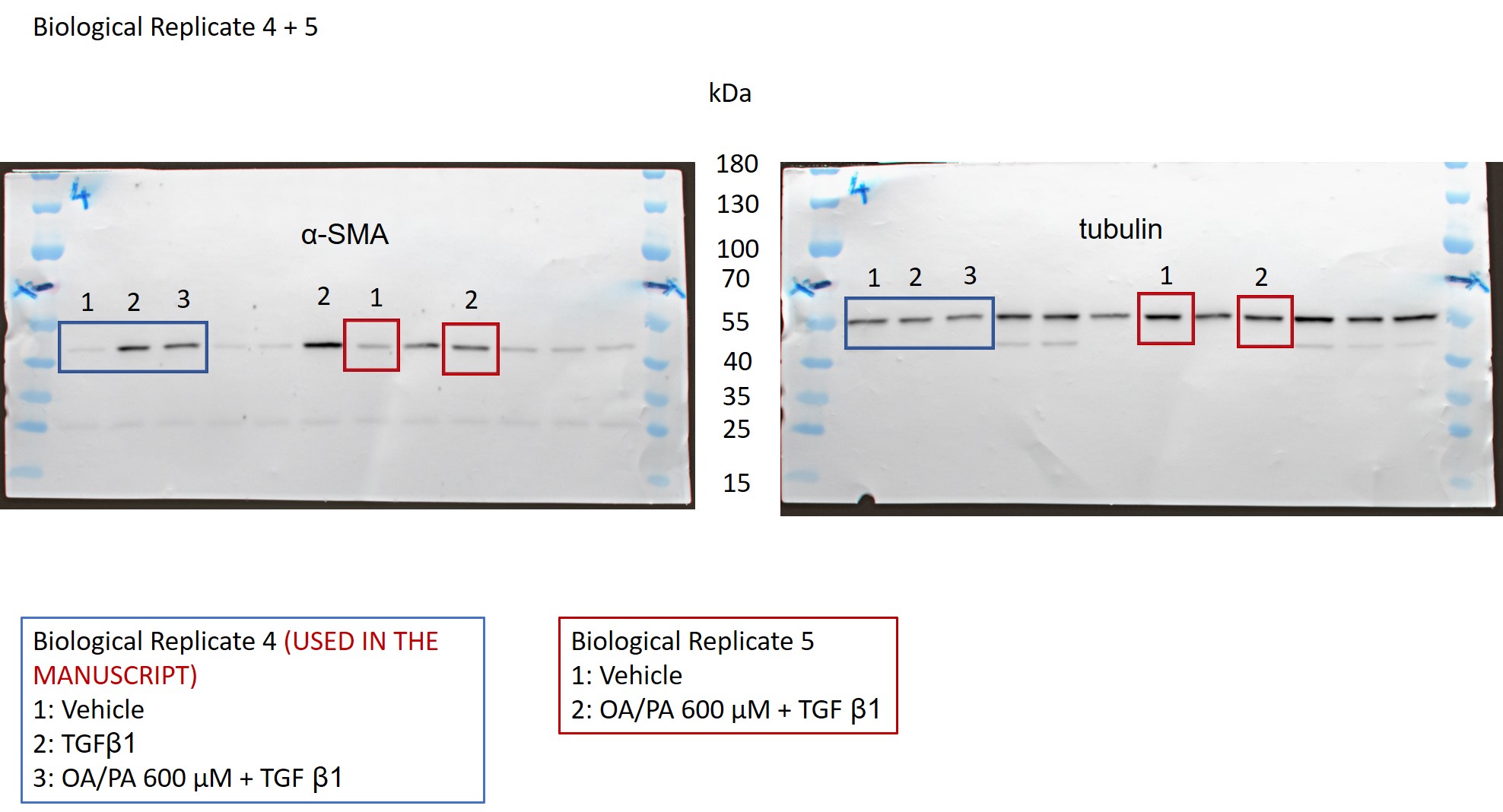

Supplement: Supplementary file 5 — Source data Fig. 3 [file 44321_2026_464_MOESM5_ESM.zip › Figure 3/Figure 3I/Replicate 4+5 - aSMA.jpg]

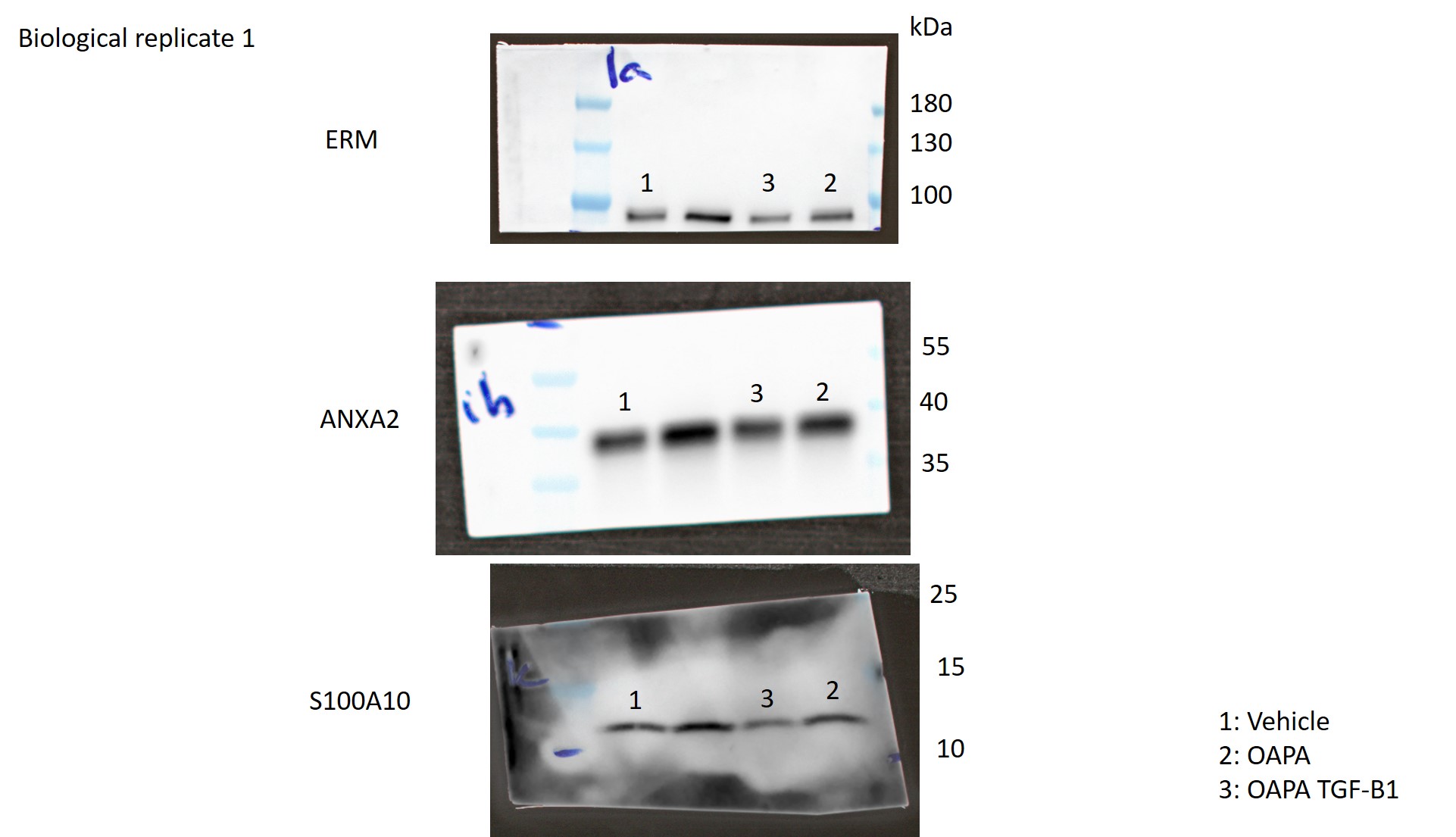

Supplement: Supplementary file 5 — Source data Fig. 3 [file 44321_2026_464_MOESM5_ESM.zip › Figure 3/Figure 3L/Replicate 1.jpg]

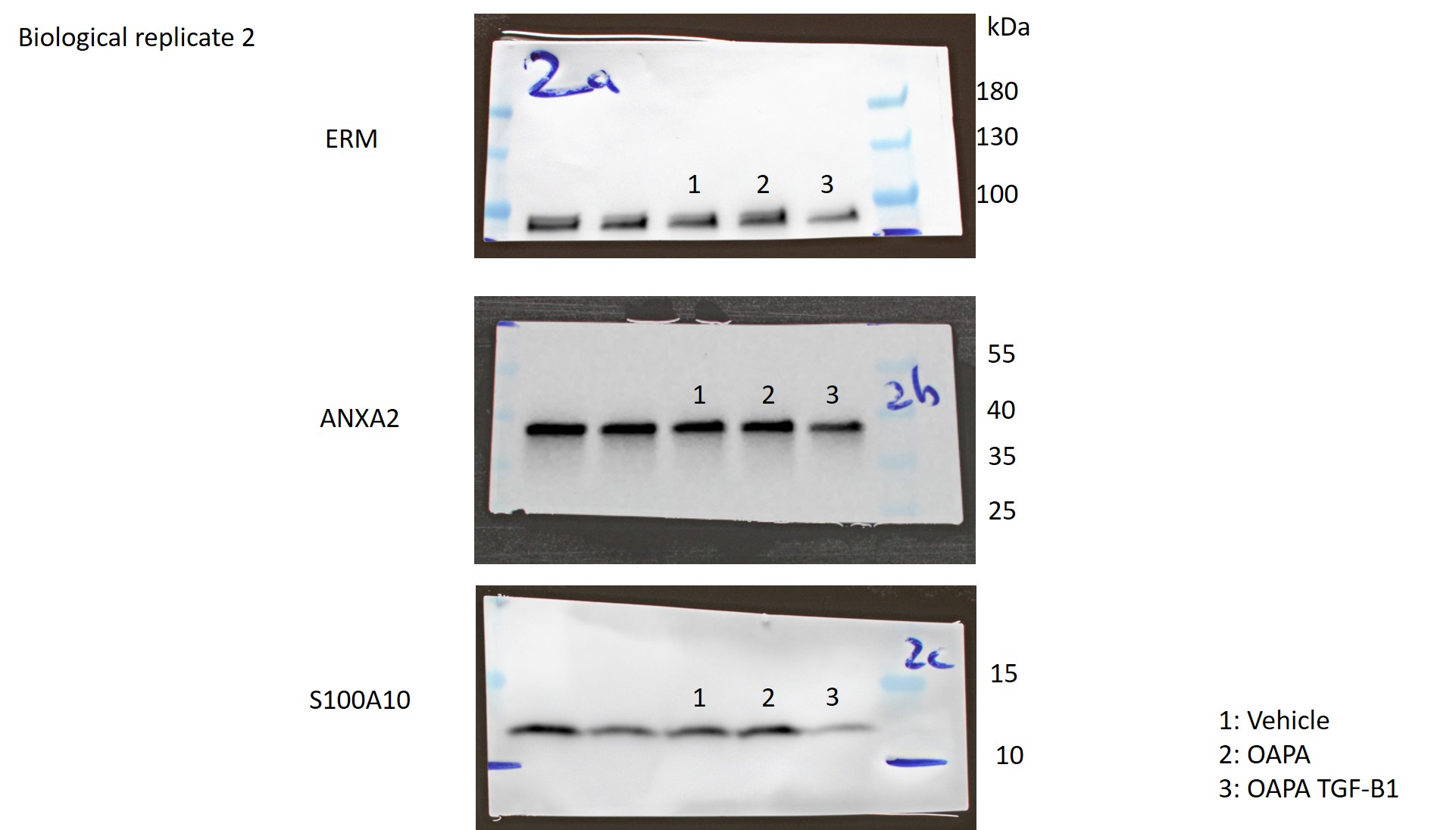

Supplement: Supplementary file 5 — Source data Fig. 3 [file 44321_2026_464_MOESM5_ESM.zip › Figure 3/Figure 3L/Replicate 2.jpg]

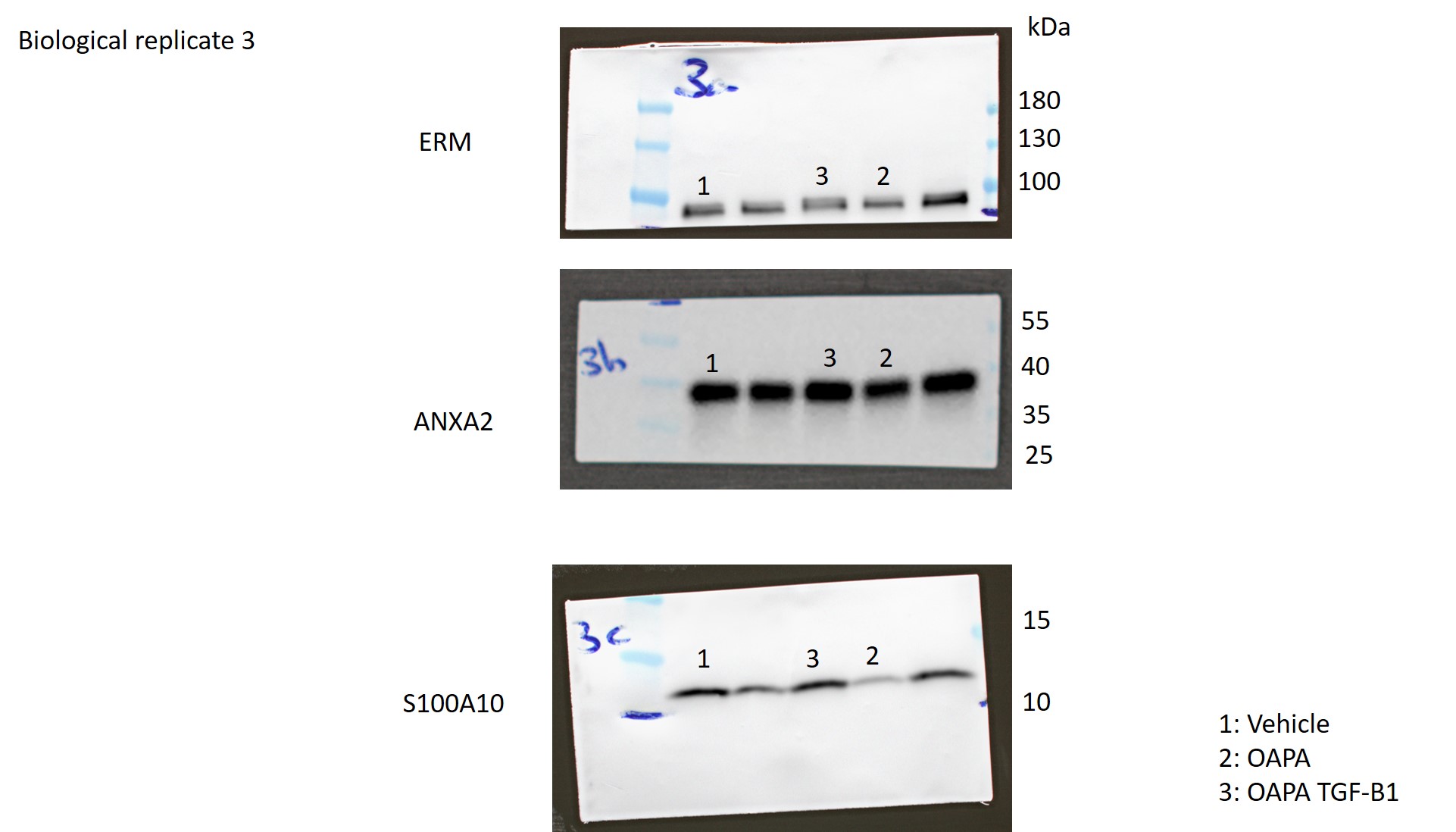

Supplement: Supplementary file 5 — Source data Fig. 3 [file 44321_2026_464_MOESM5_ESM.zip › Figure 3/Figure 3L/Replicate 3.jpg]

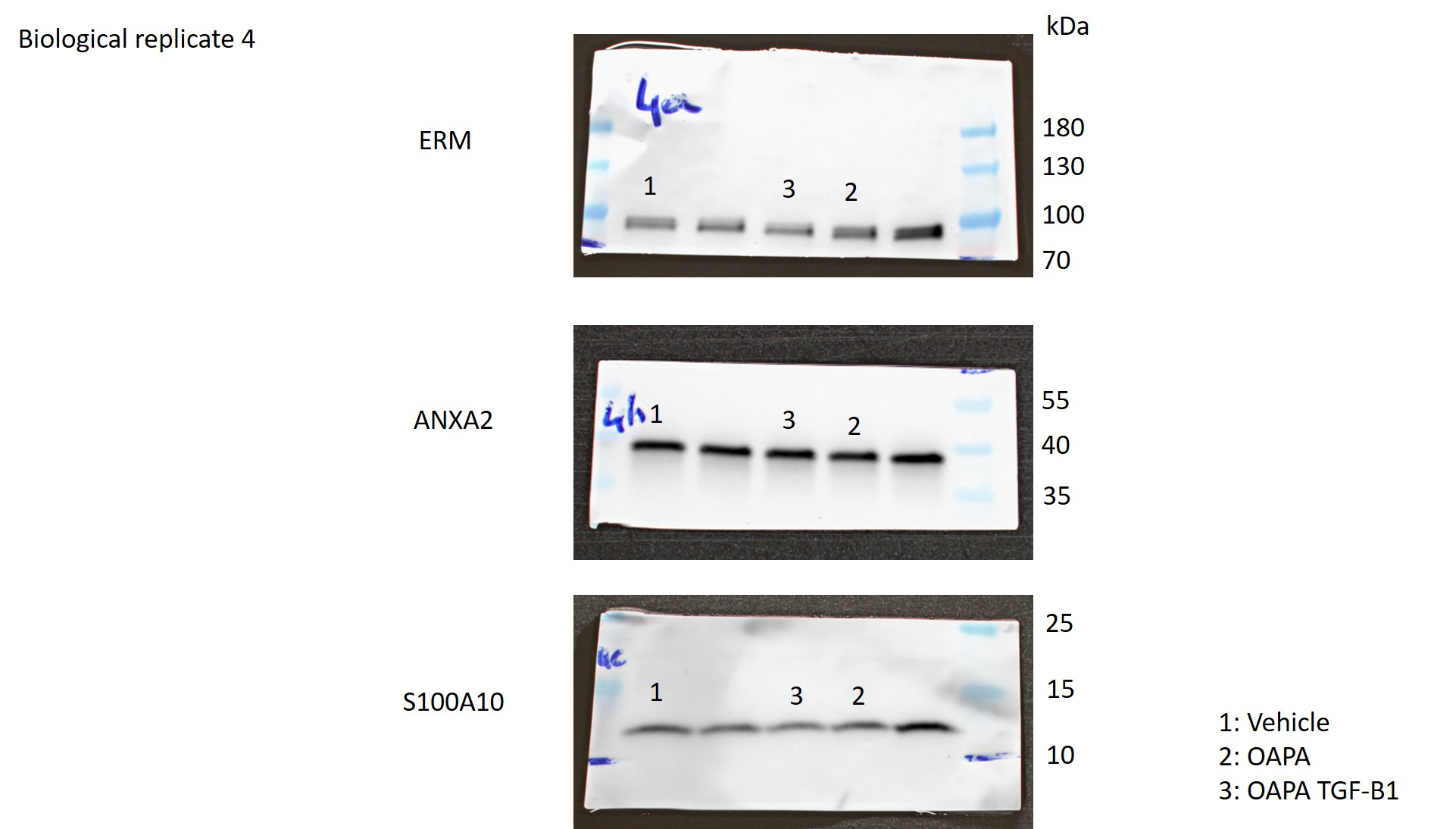

Supplement: Supplementary file 5 — Source data Fig. 3 [file 44321_2026_464_MOESM5_ESM.zip › Figure 3/Figure 3L/Replicate 4.jpg]

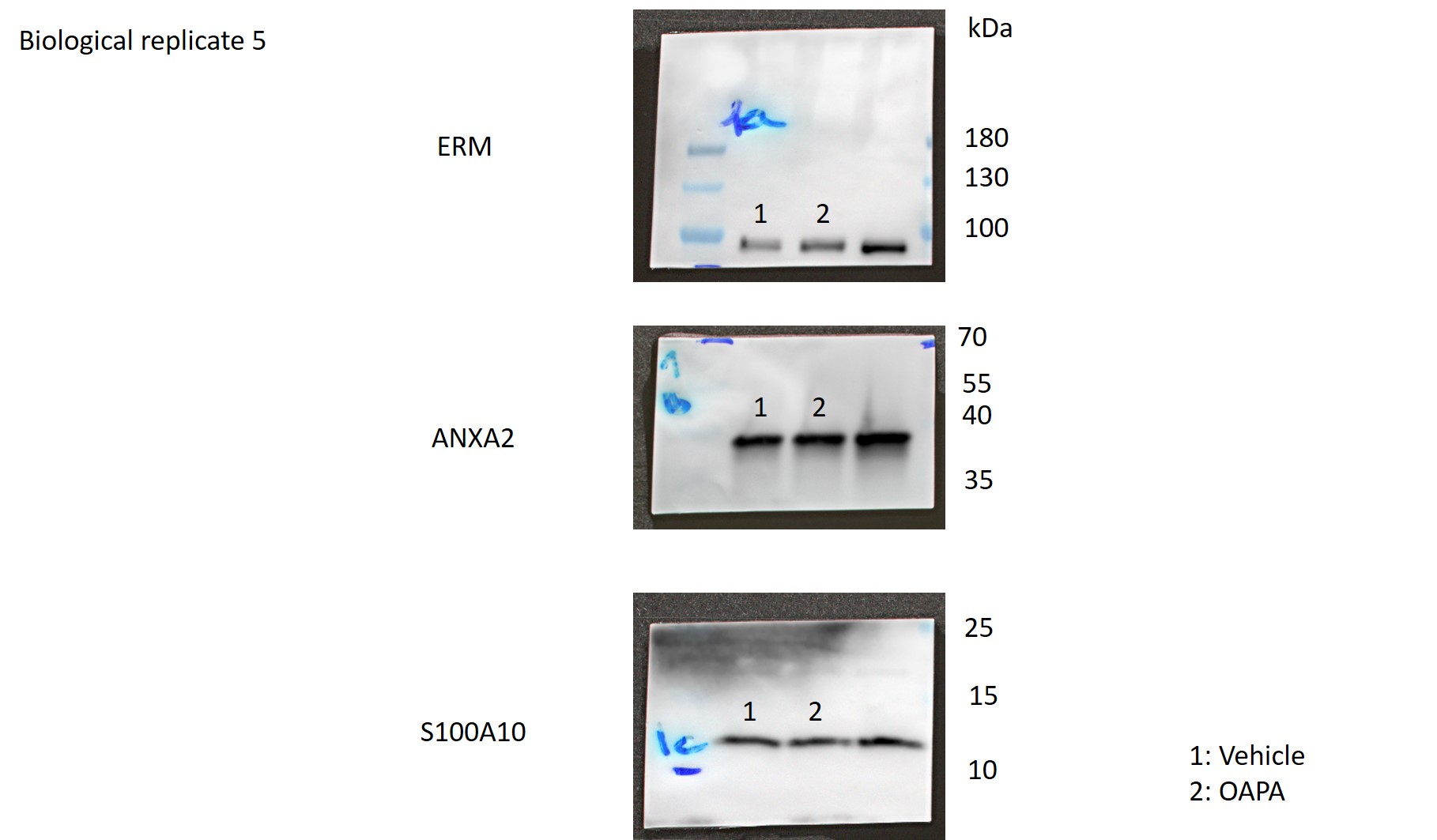

Supplement: Supplementary file 5 — Source data Fig. 3 [file 44321_2026_464_MOESM5_ESM.zip › Figure 3/Figure 3L/Replicate 5.jpg]

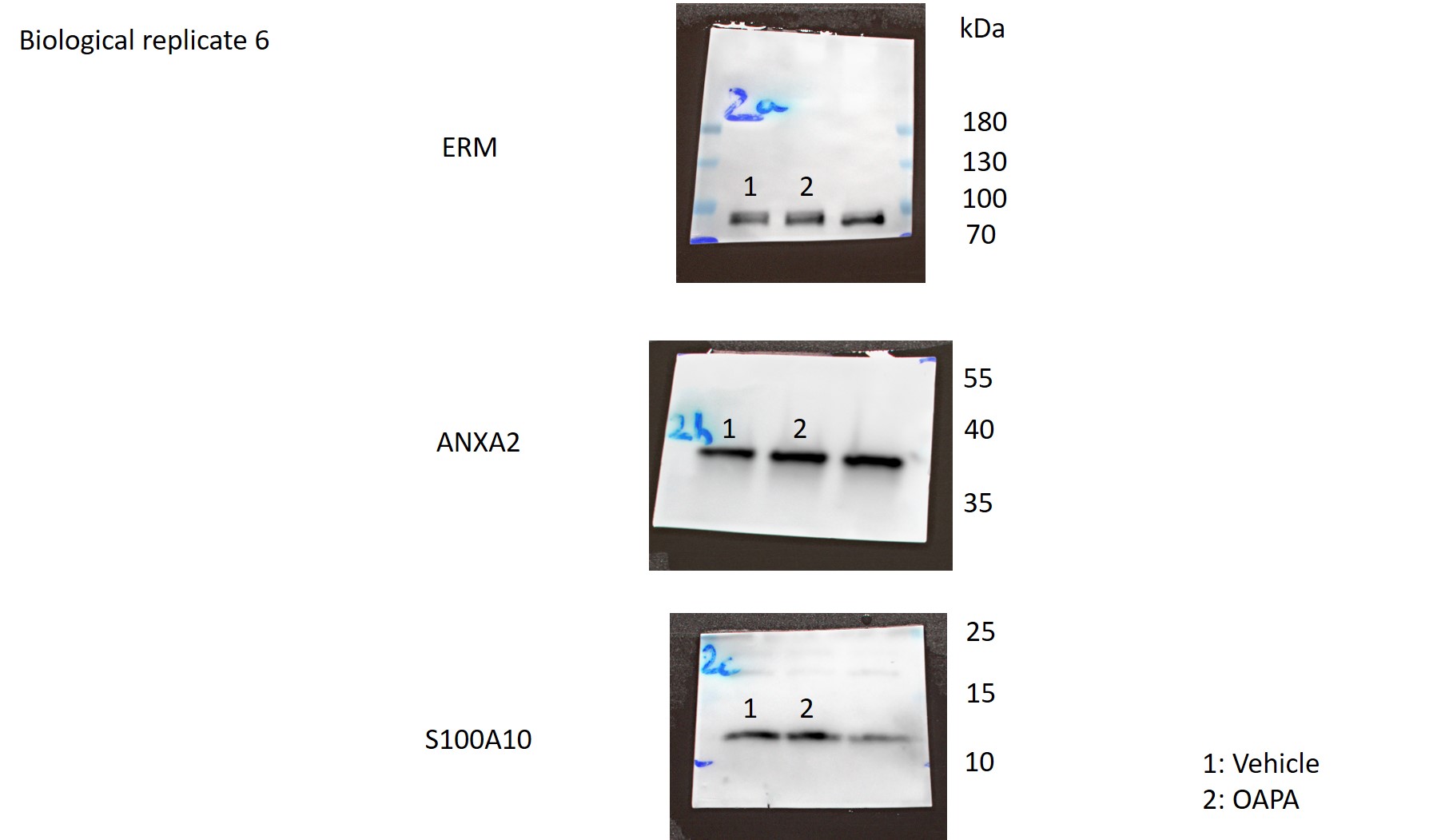

Supplement: Supplementary file 5 — Source data Fig. 3 [file 44321_2026_464_MOESM5_ESM.zip › Figure 3/Figure 3L/Replicate 6.jpg]

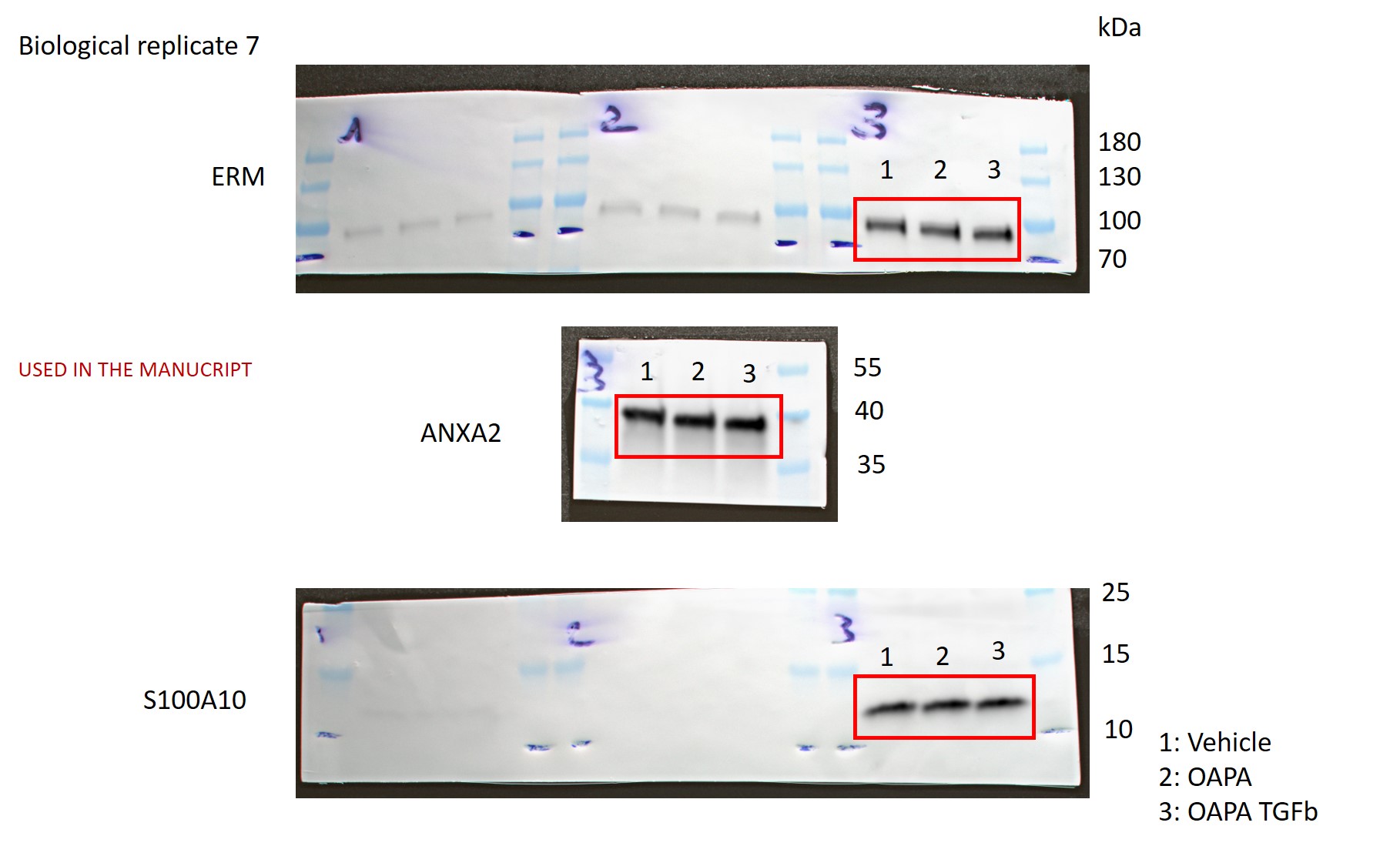

Supplement: Supplementary file 5 — Source data Fig. 3 [file 44321_2026_464_MOESM5_ESM.zip › Figure 3/Figure 3L/Replicate 7.jpg]

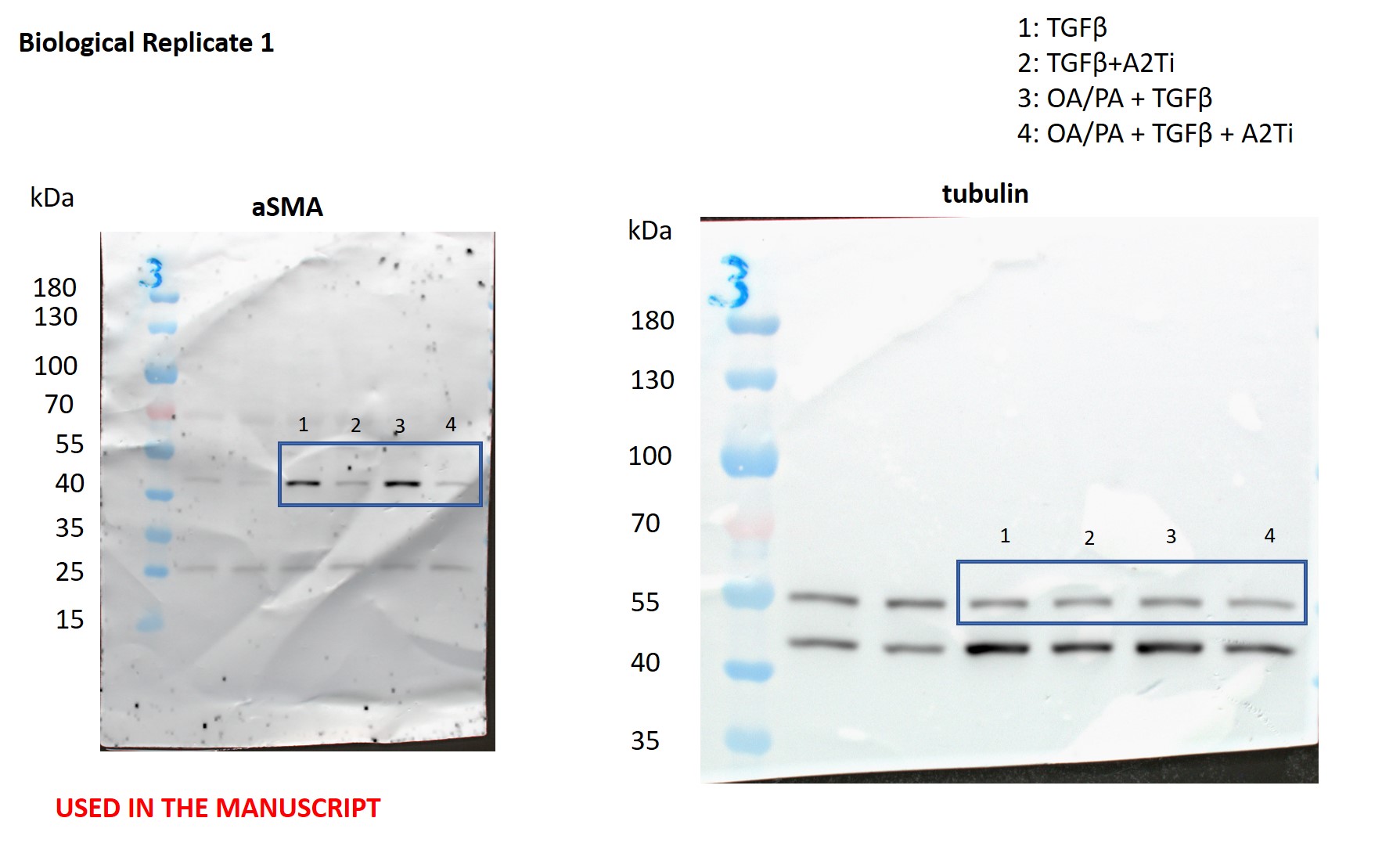

Supplement: Supplementary file 6 — Source data Fig. 4 [file 44321_2026_464_MOESM6_ESM.zip › Figure 4/Figure_4C/Replicate 1.jpg]

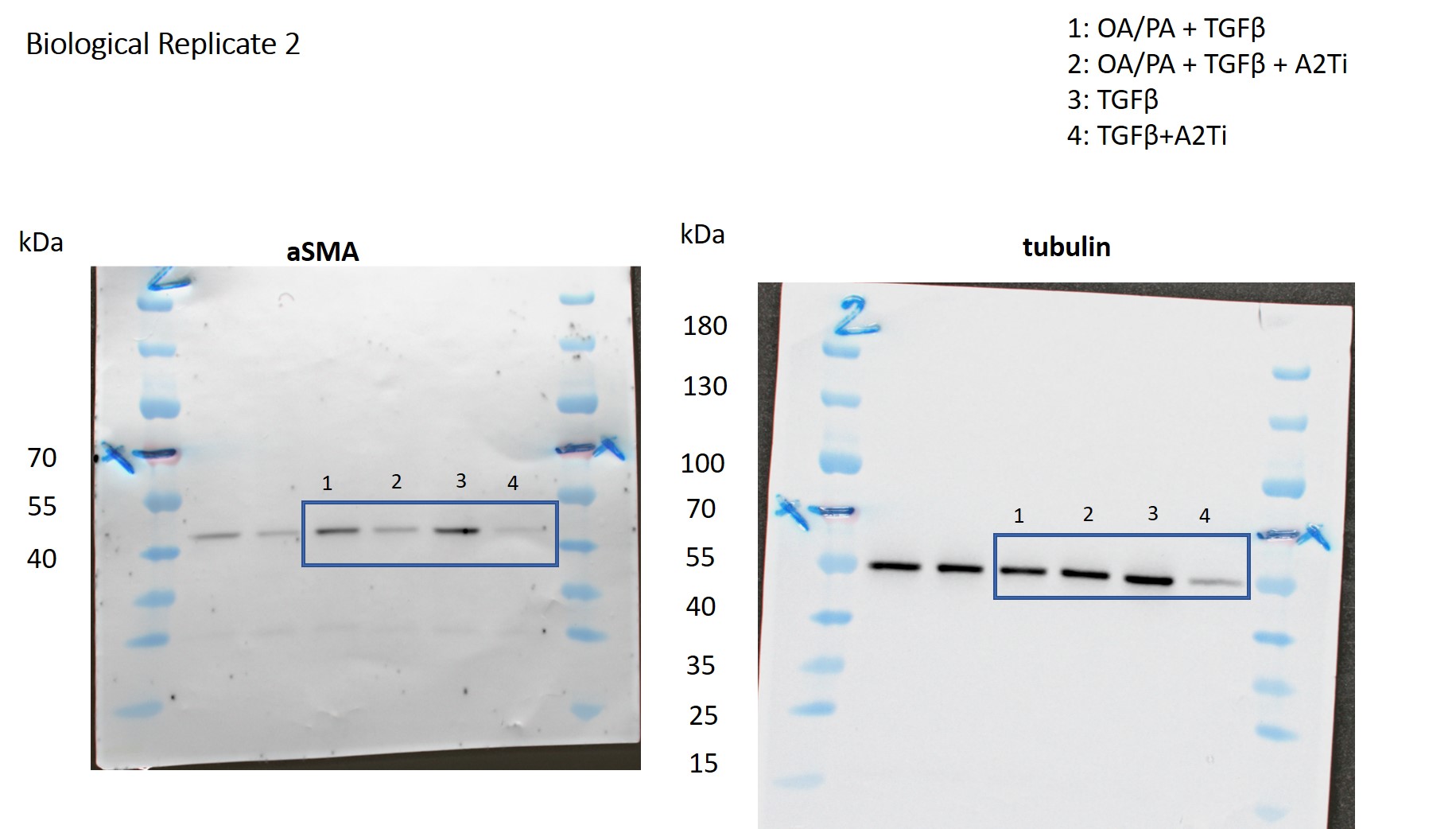

Supplement: Supplementary file 6 — Source data Fig. 4 [file 44321_2026_464_MOESM6_ESM.zip › Figure 4/Figure_4C/Replicate 2.jpg]

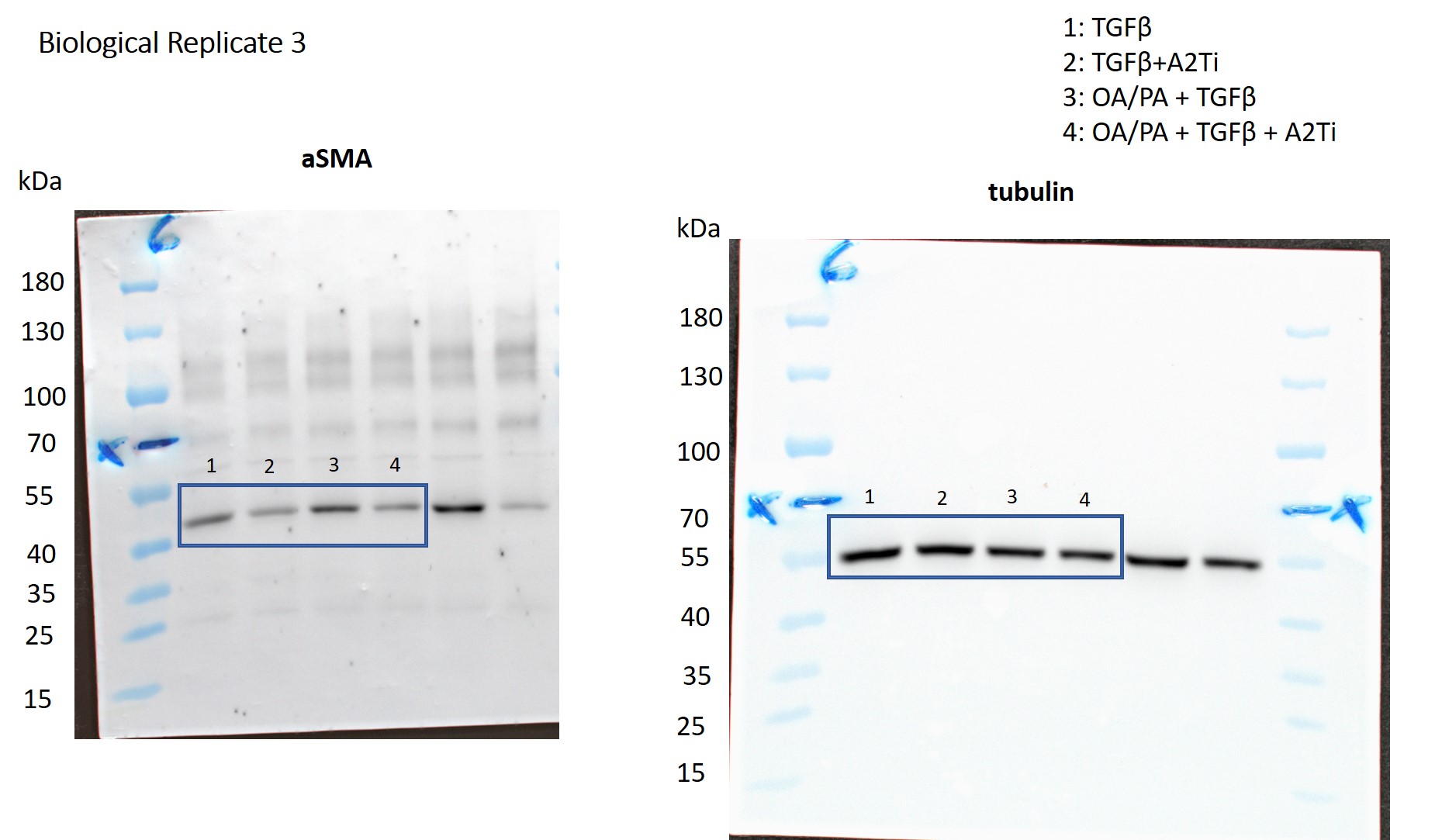

Supplement: Supplementary file 6 — Source data Fig. 4 [file 44321_2026_464_MOESM6_ESM.zip › Figure 4/Figure_4C/Replicate 3.jpg]

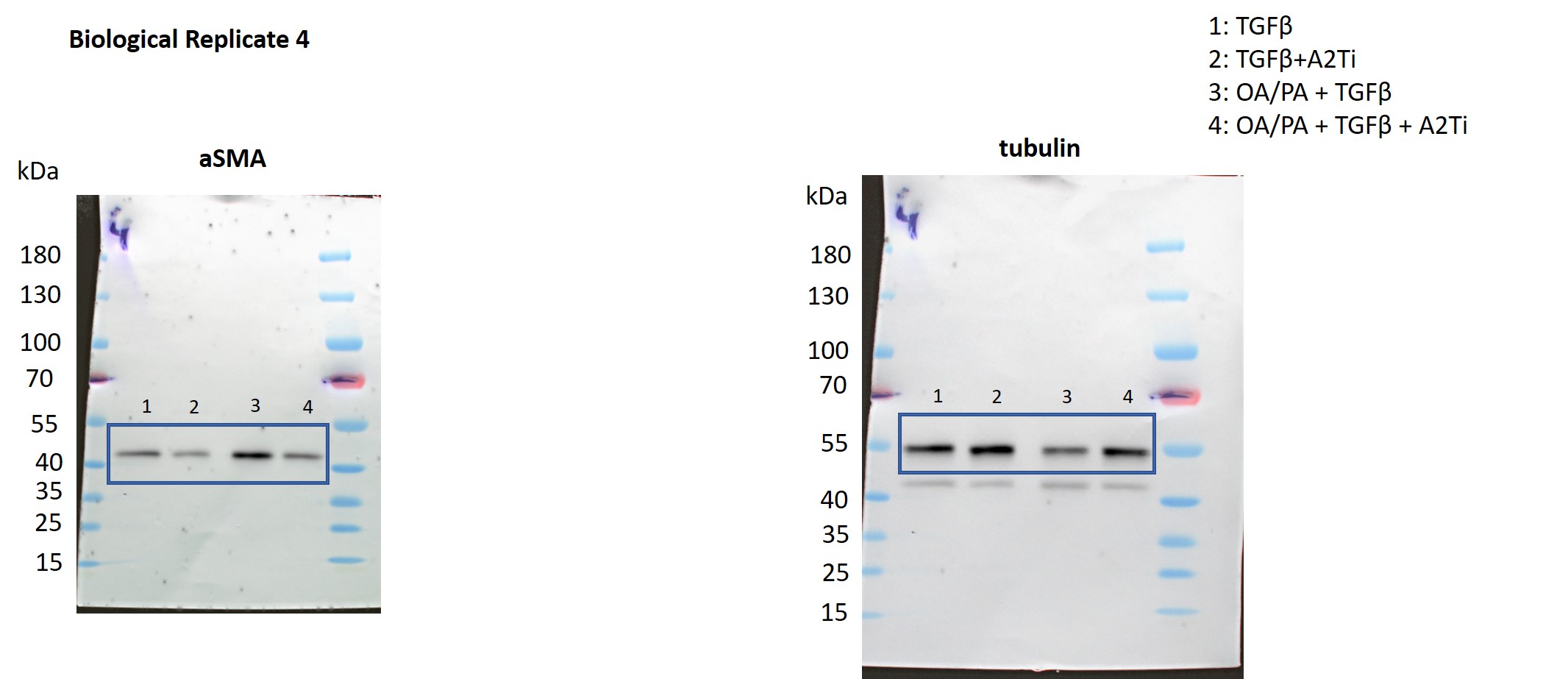

Supplement: Supplementary file 6 — Source data Fig. 4 [file 44321_2026_464_MOESM6_ESM.zip › Figure 4/Figure_4C/Replicate 4.jpg]

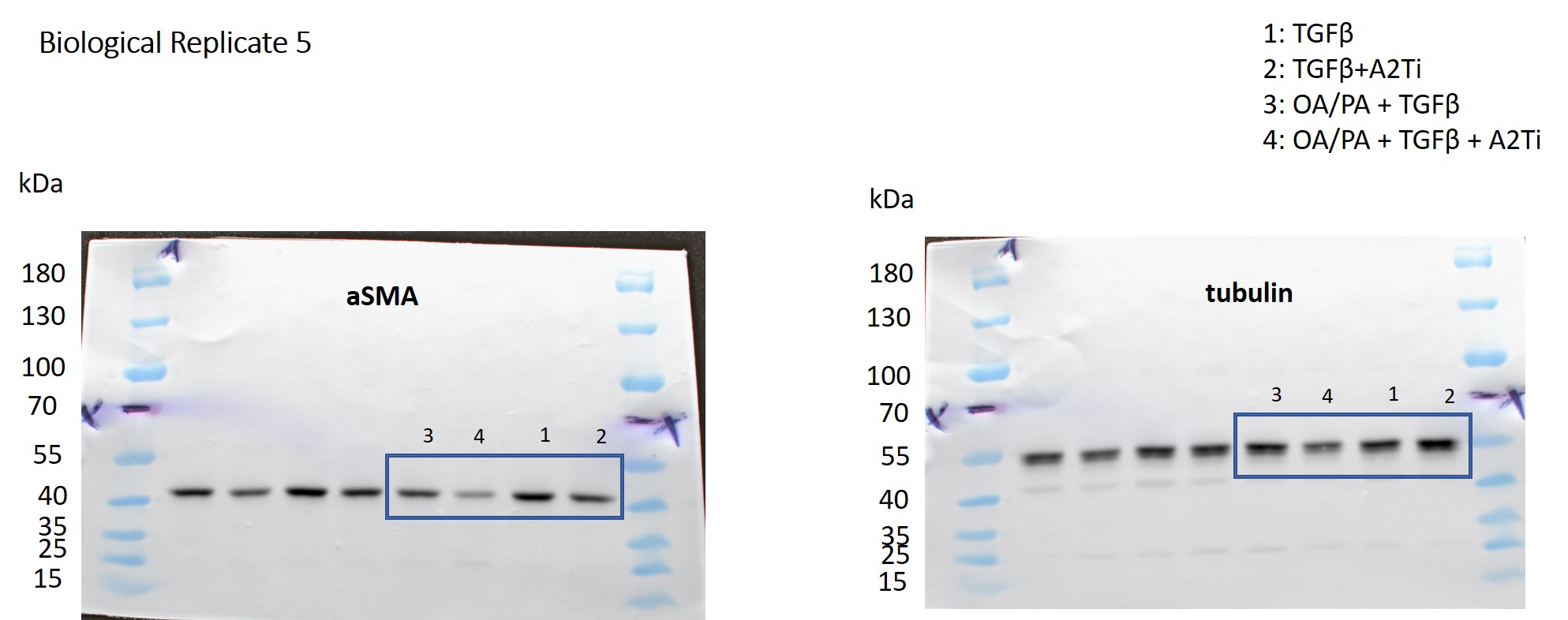

Supplement: Supplementary file 6 — Source data Fig. 4 [file 44321_2026_464_MOESM6_ESM.zip › Figure 4/Figure_4C/Replicate 5.jpg]

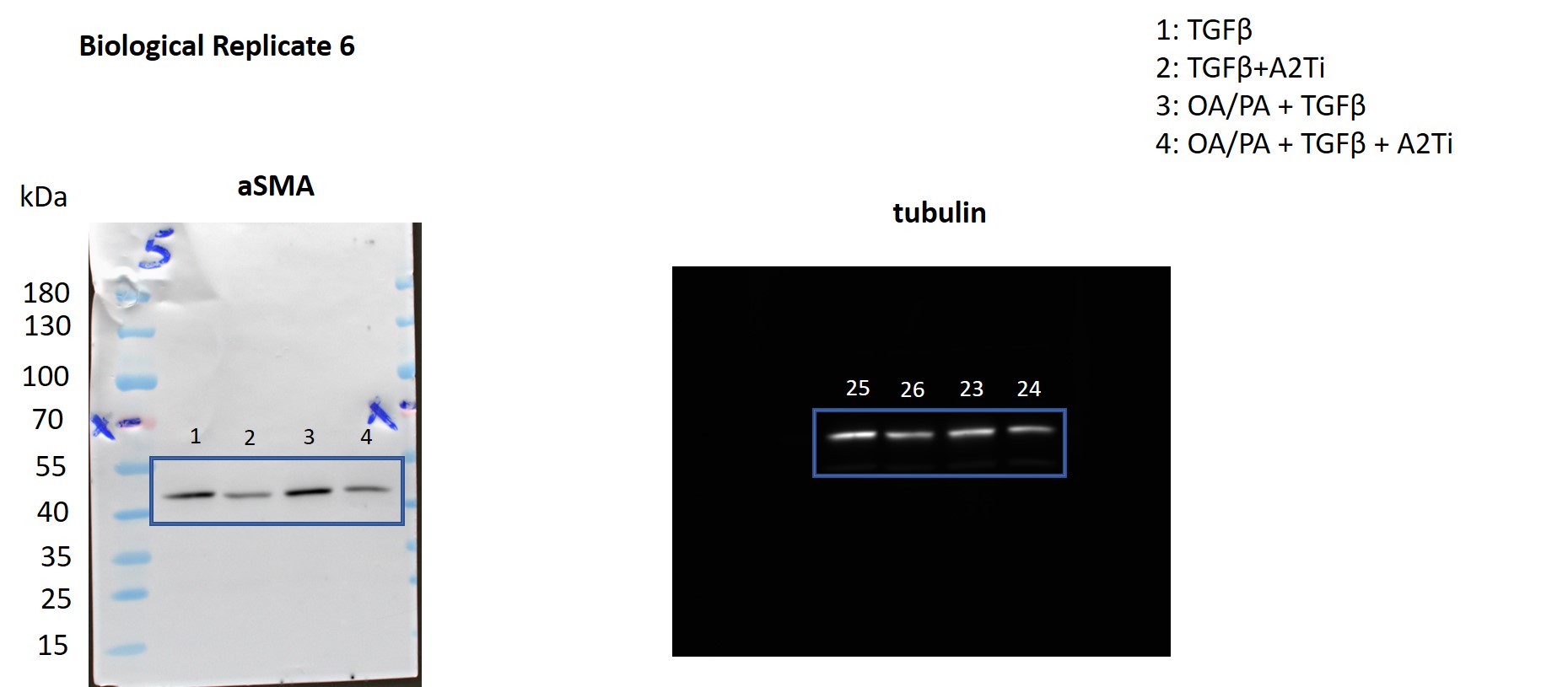

Supplement: Supplementary file 6 — Source data Fig. 4 [file 44321_2026_464_MOESM6_ESM.zip › Figure 4/Figure_4C/Replicate 6.jpg]

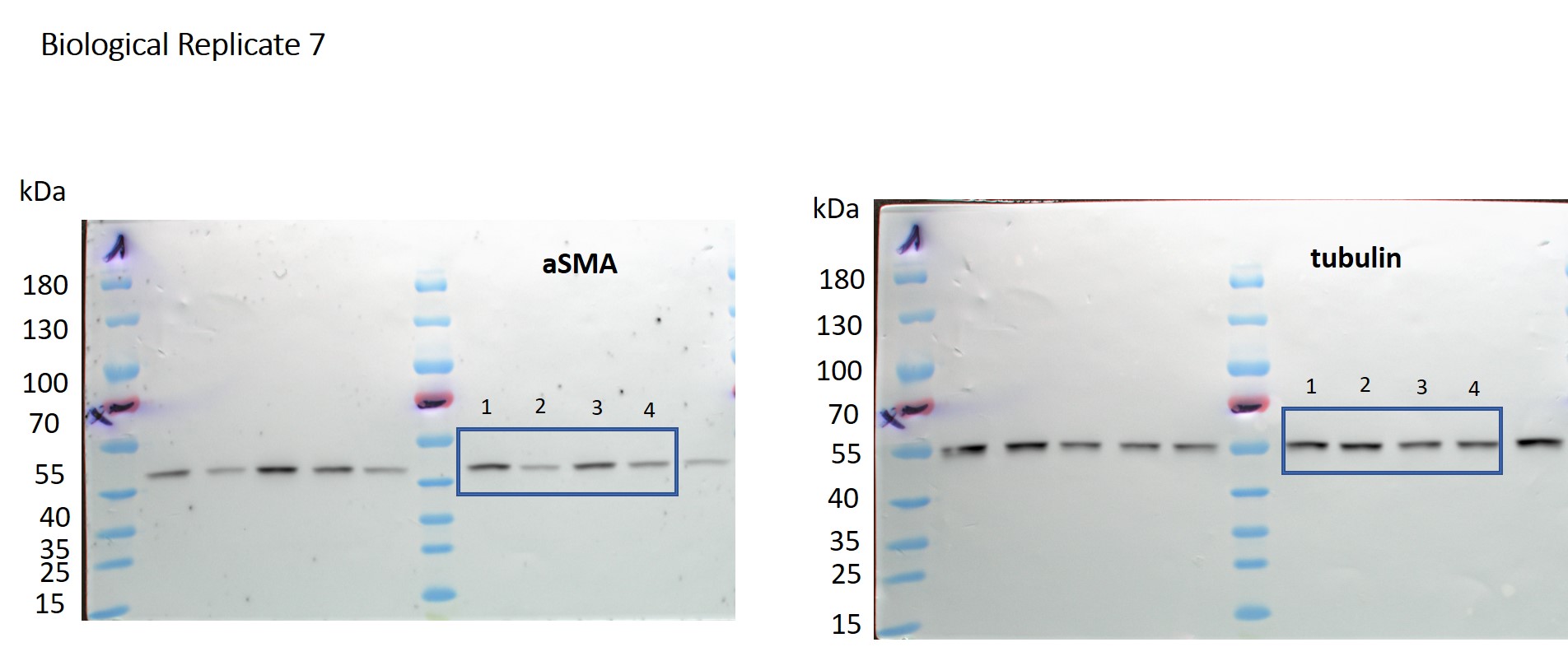

Supplement: Supplementary file 6 — Source data Fig. 4 [file 44321_2026_464_MOESM6_ESM.zip › Figure 4/Figure_4C/Replicate 7.jpg]

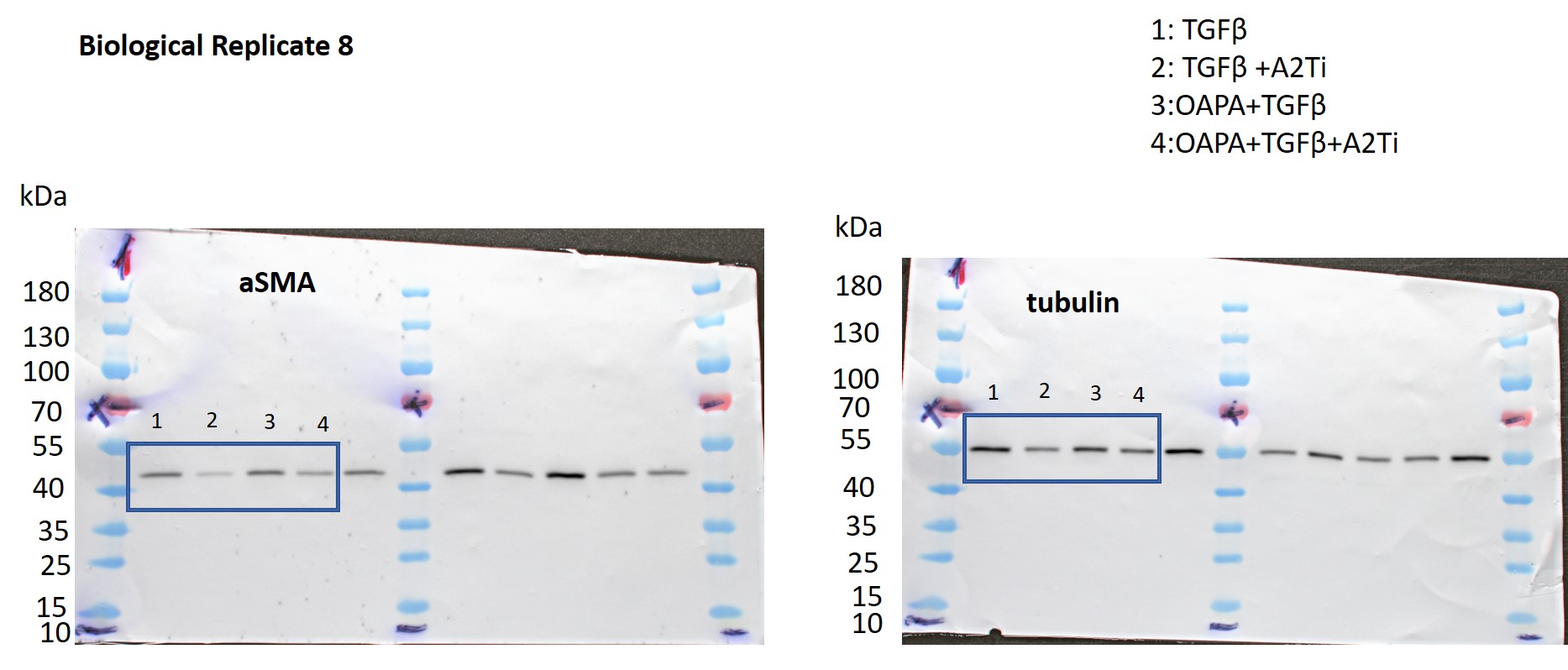

Supplement: Supplementary file 6 — Source data Fig. 4 [file 44321_2026_464_MOESM6_ESM.zip › Figure 4/Figure_4C/Replicate 8.jpg]

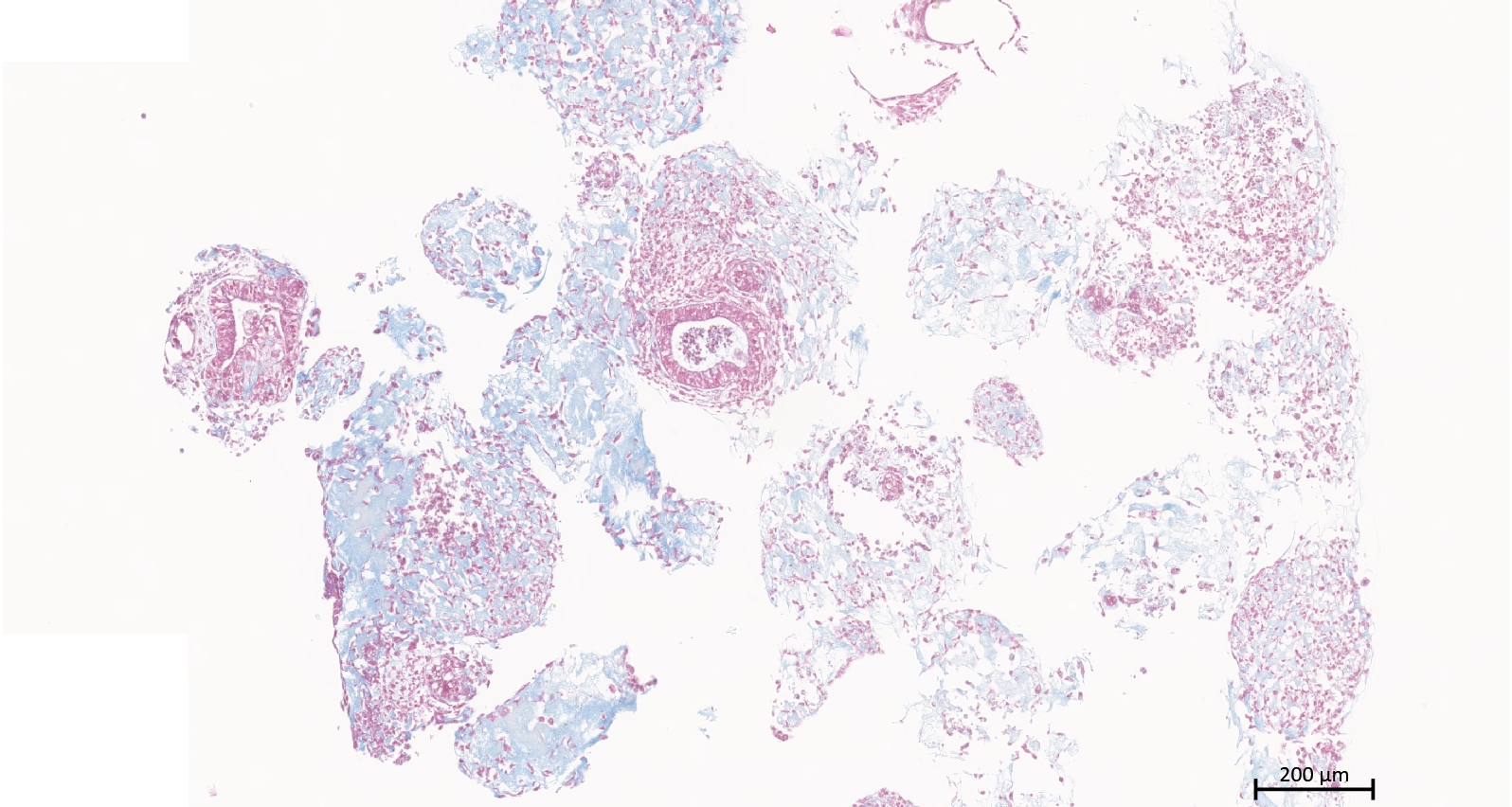

Supplement: Supplementary file 6 — Source data Fig. 4 [file 44321_2026_464_MOESM6_ESM.zip › Figure 4/Figure_4D/Figure_4D_TGFB1.jpeg]

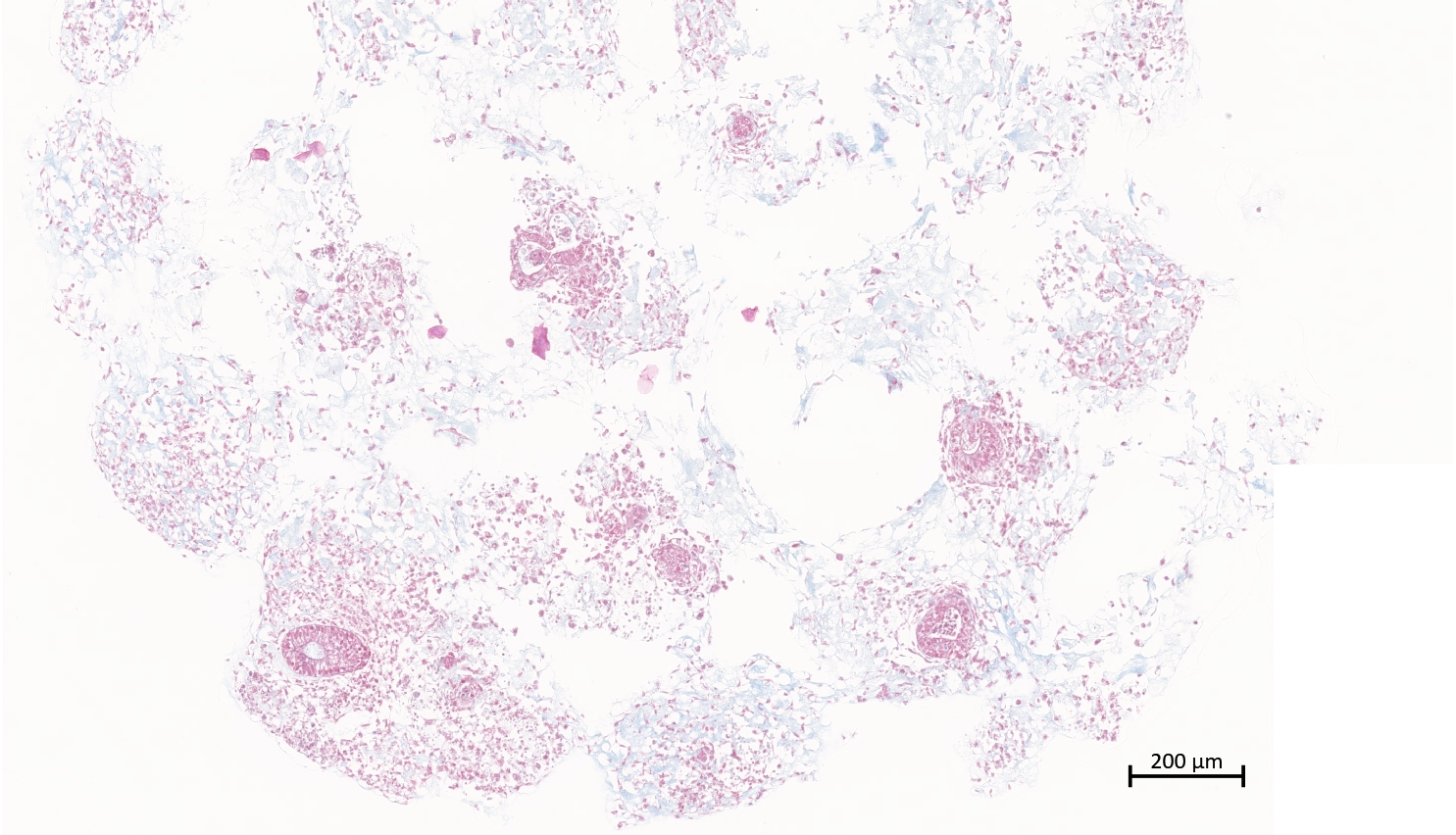

Supplement: Supplementary file 6 — Source data Fig. 4 [file 44321_2026_464_MOESM6_ESM.zip › Figure 4/Figure_4D/Figure_4D_TGFB1_A2ti.jpeg]

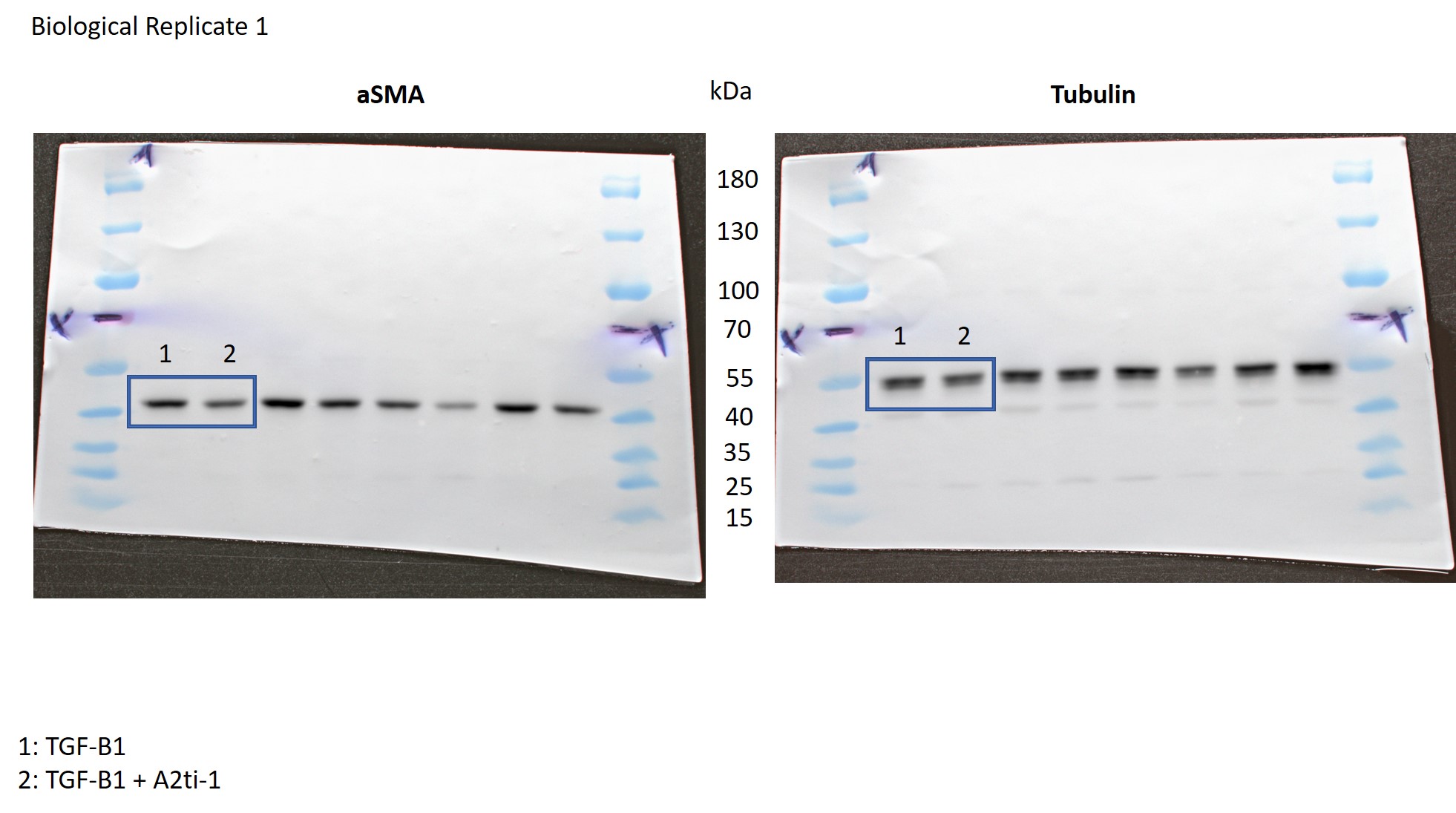

Supplement: Supplementary file 6 — Source data Fig. 4 [file 44321_2026_464_MOESM6_ESM.zip › Figure 4/Figure_4H/Replicate 1.jpg]

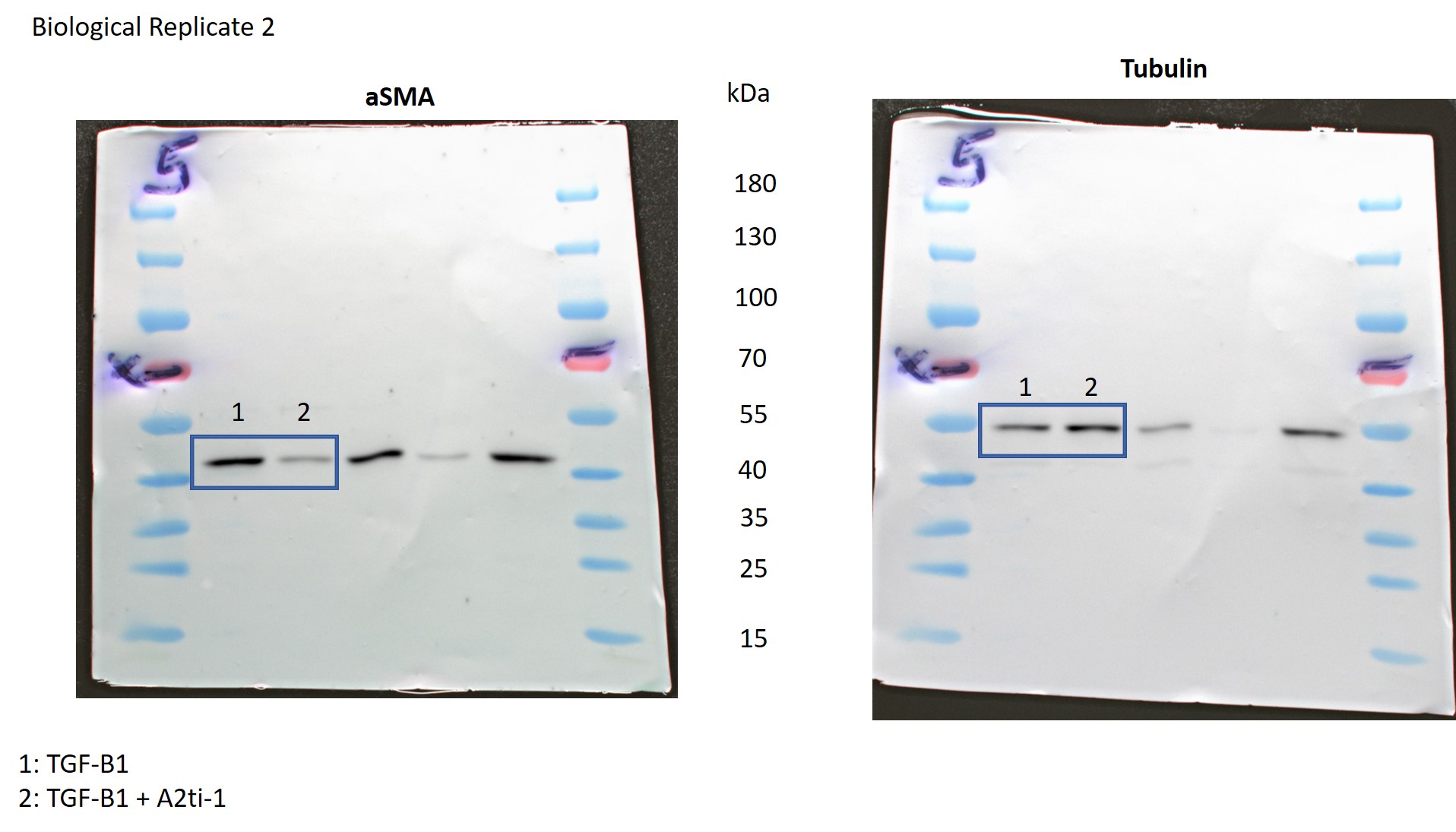

Supplement: Supplementary file 6 — Source data Fig. 4 [file 44321_2026_464_MOESM6_ESM.zip › Figure 4/Figure_4H/Replicate 2.jpg]

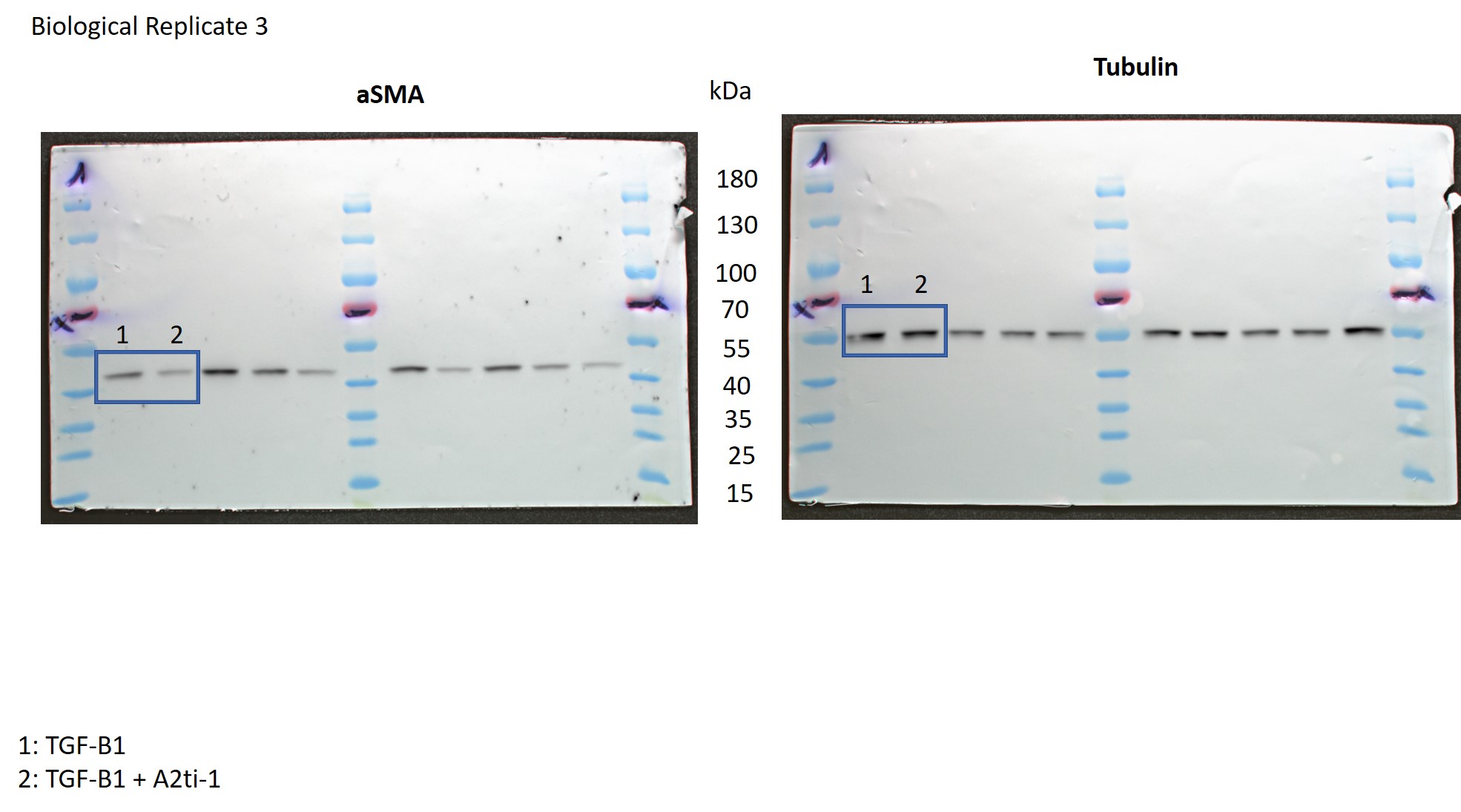

Supplement: Supplementary file 6 — Source data Fig. 4 [file 44321_2026_464_MOESM6_ESM.zip › Figure 4/Figure_4H/Replicate 3.jpg]

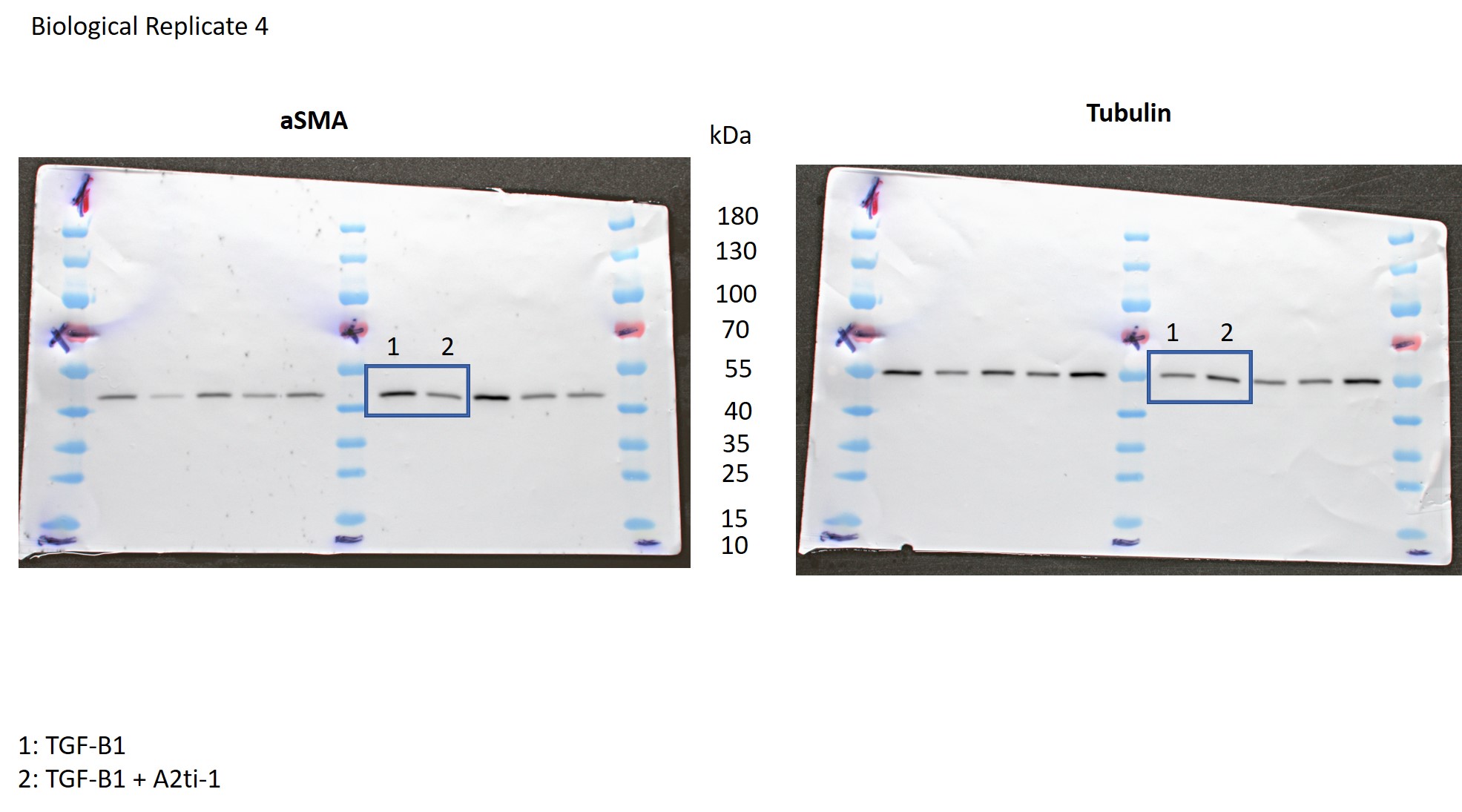

Supplement: Supplementary file 6 — Source data Fig. 4 [file 44321_2026_464_MOESM6_ESM.zip › Figure 4/Figure_4H/Replicate 4.jpg]

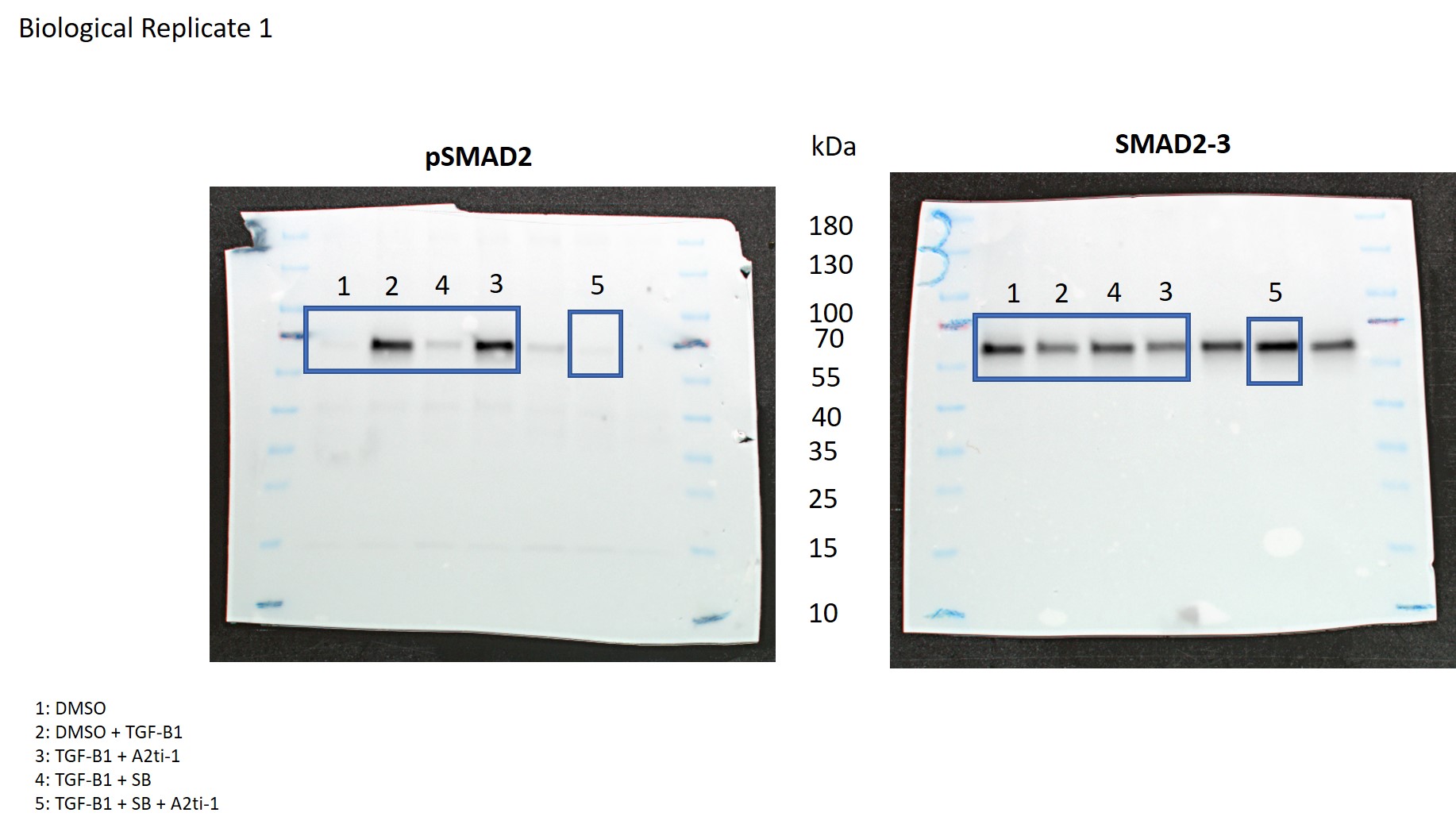

Supplement: Supplementary file 7 — Source data Fig. 5 [file 44321_2026_464_MOESM7_ESM.zip › Figure 5/Figure 5B_5C/Replicate 1 - pSMAD2-SMAD2.jpg]

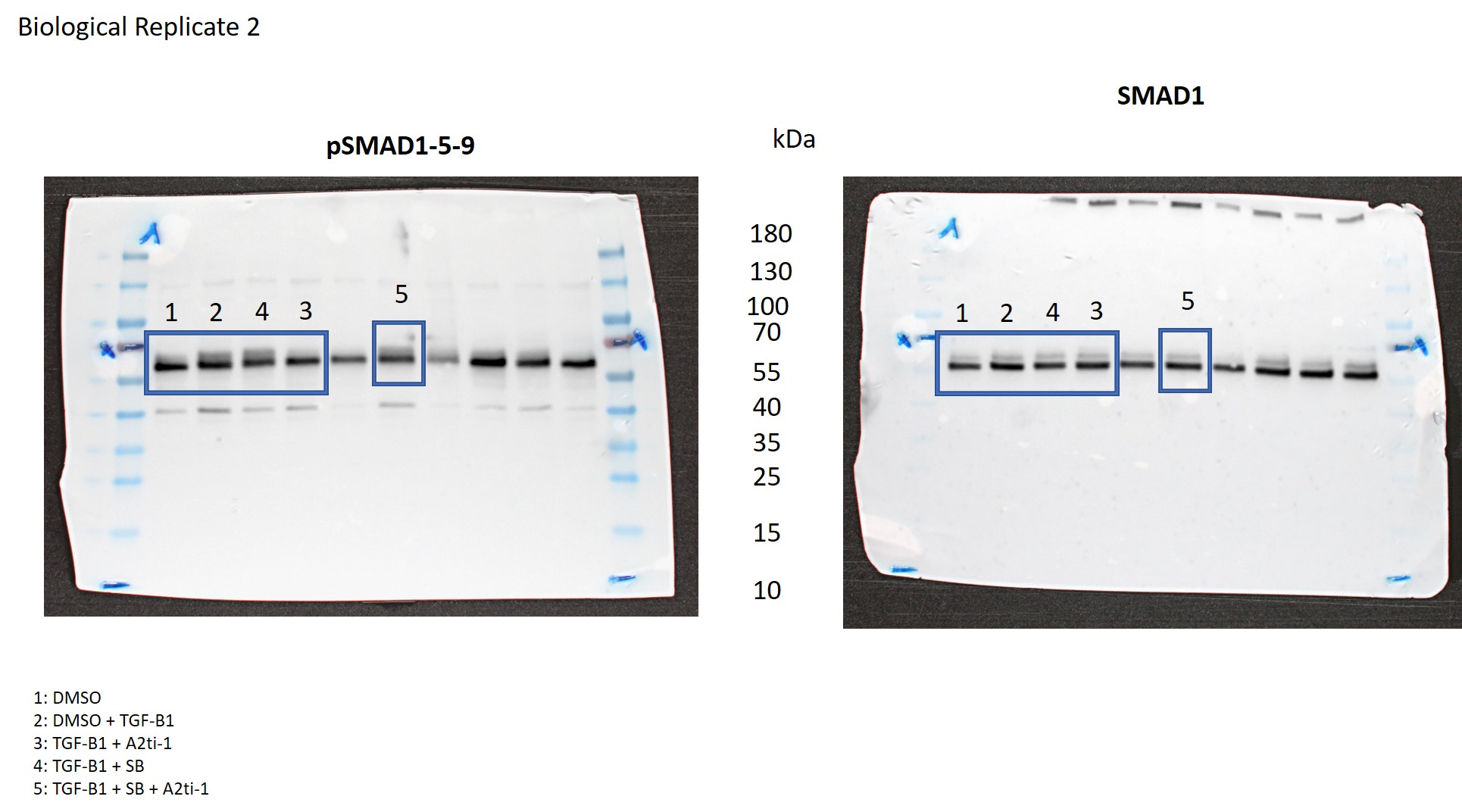

Supplement: Supplementary file 7 — Source data Fig. 5 [file 44321_2026_464_MOESM7_ESM.zip › Figure 5/Figure 5B_5C/Replicate 2 - pSMAD1-SMAD1.jpg]

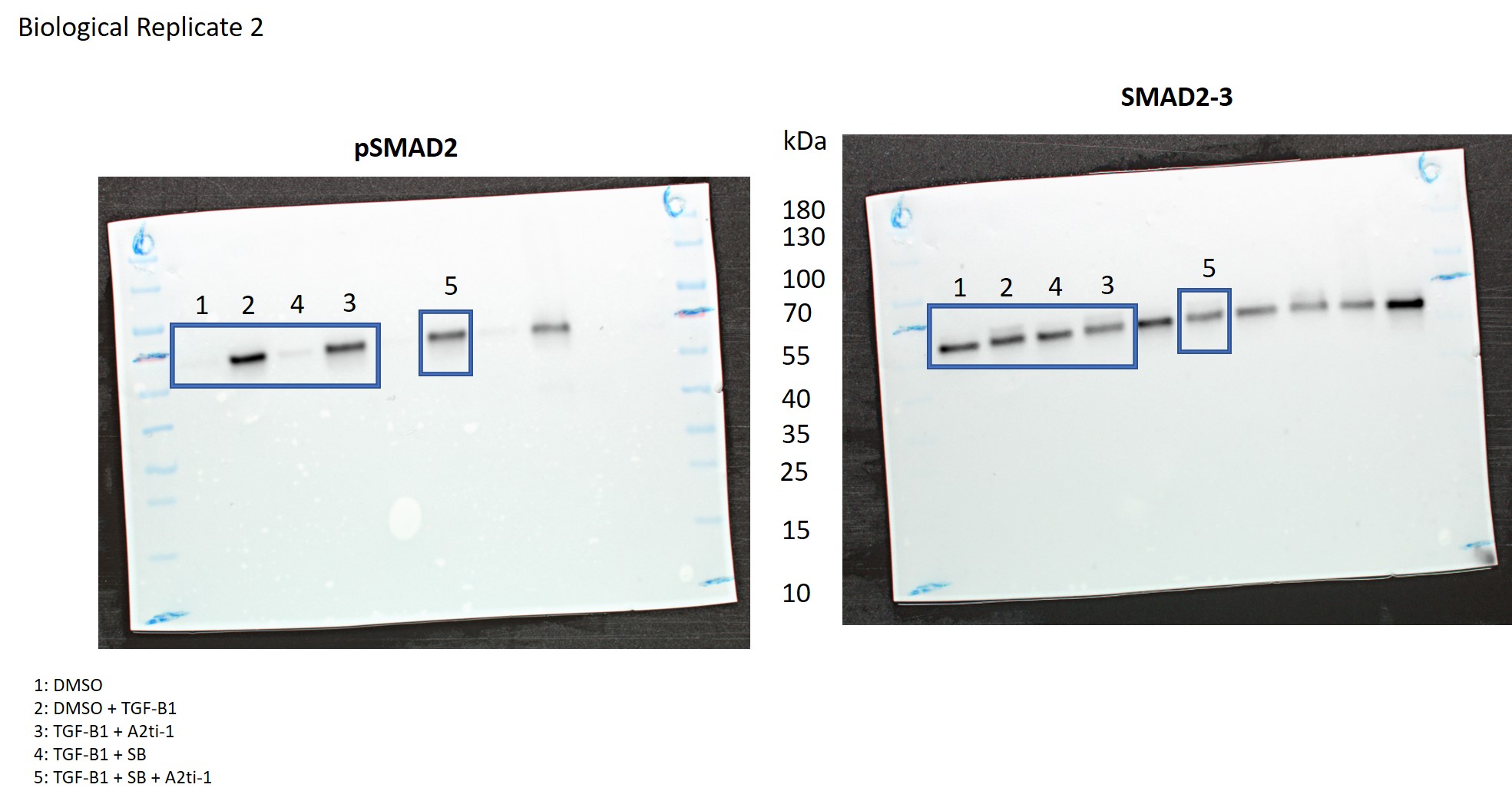

Supplement: Supplementary file 7 — Source data Fig. 5 [file 44321_2026_464_MOESM7_ESM.zip › Figure 5/Figure 5B_5C/Replicate 2 - pSMAD2-SMAD2.jpg]

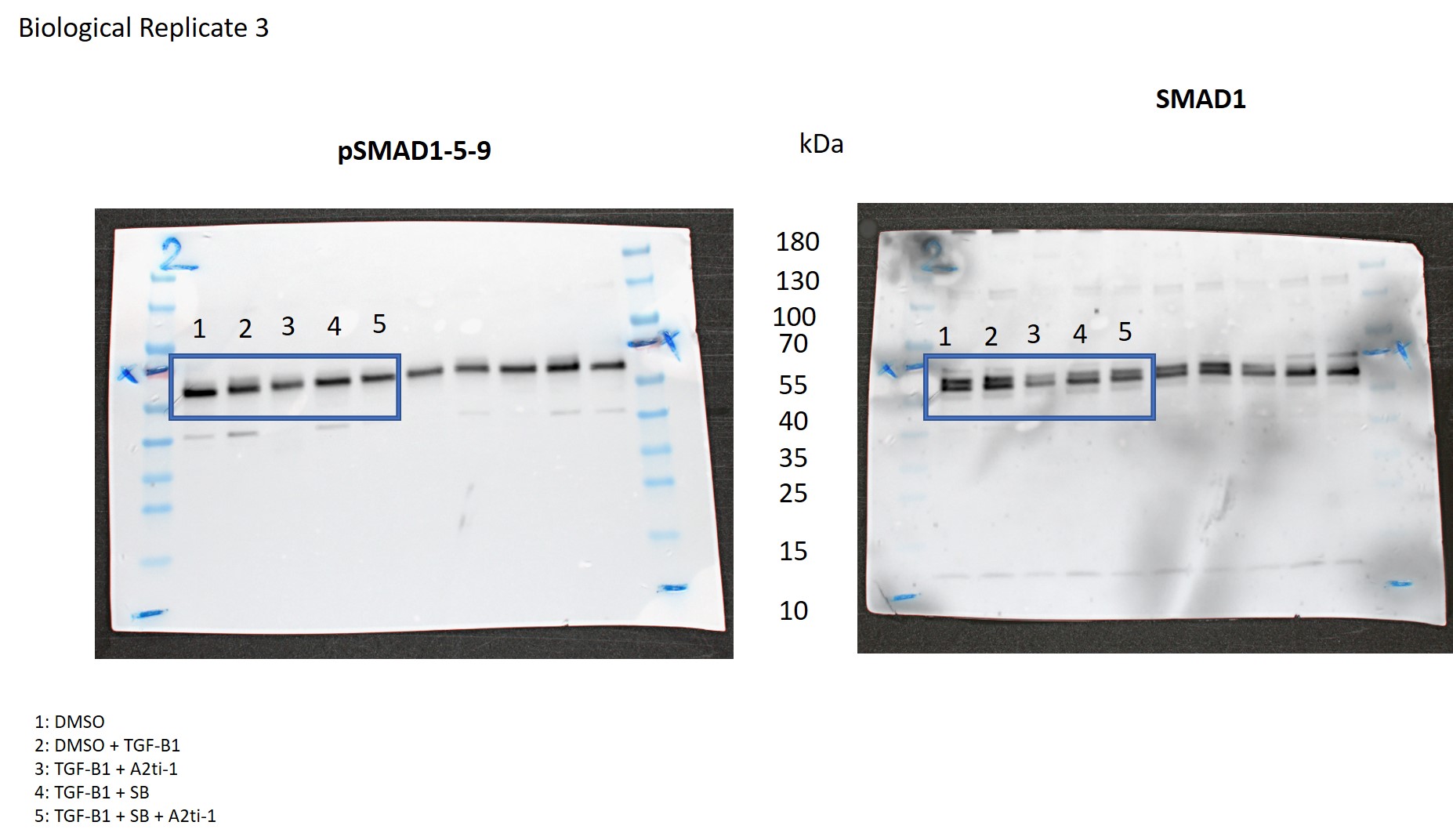

Supplement: Supplementary file 7 — Source data Fig. 5 [file 44321_2026_464_MOESM7_ESM.zip › Figure 5/Figure 5B_5C/Replicate 3 - pSMAD1-SMAD1.jpg]

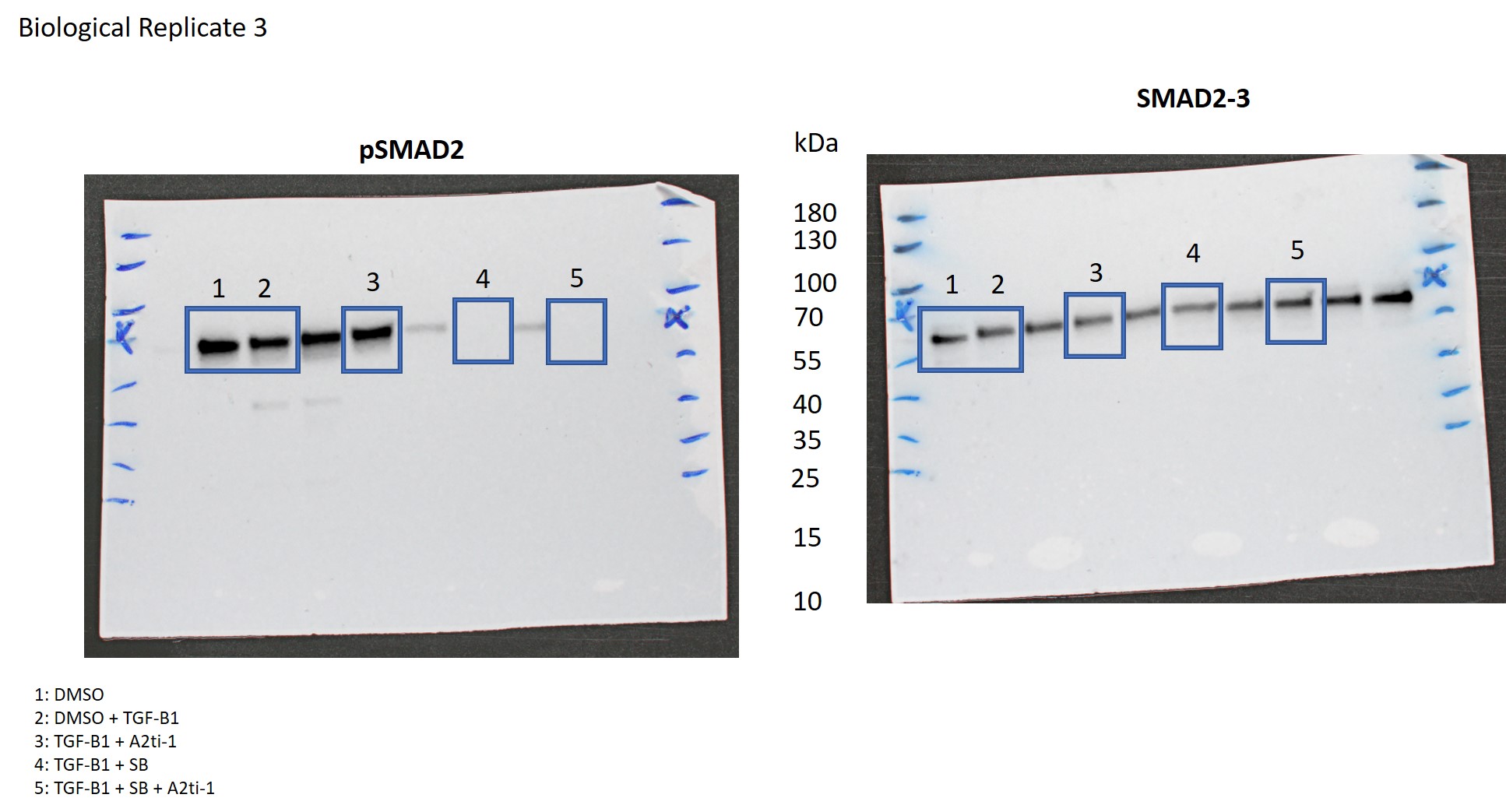

Supplement: Supplementary file 7 — Source data Fig. 5 [file 44321_2026_464_MOESM7_ESM.zip › Figure 5/Figure 5B_5C/Replicate 3 - pSMAD2-SMAD2.jpg]

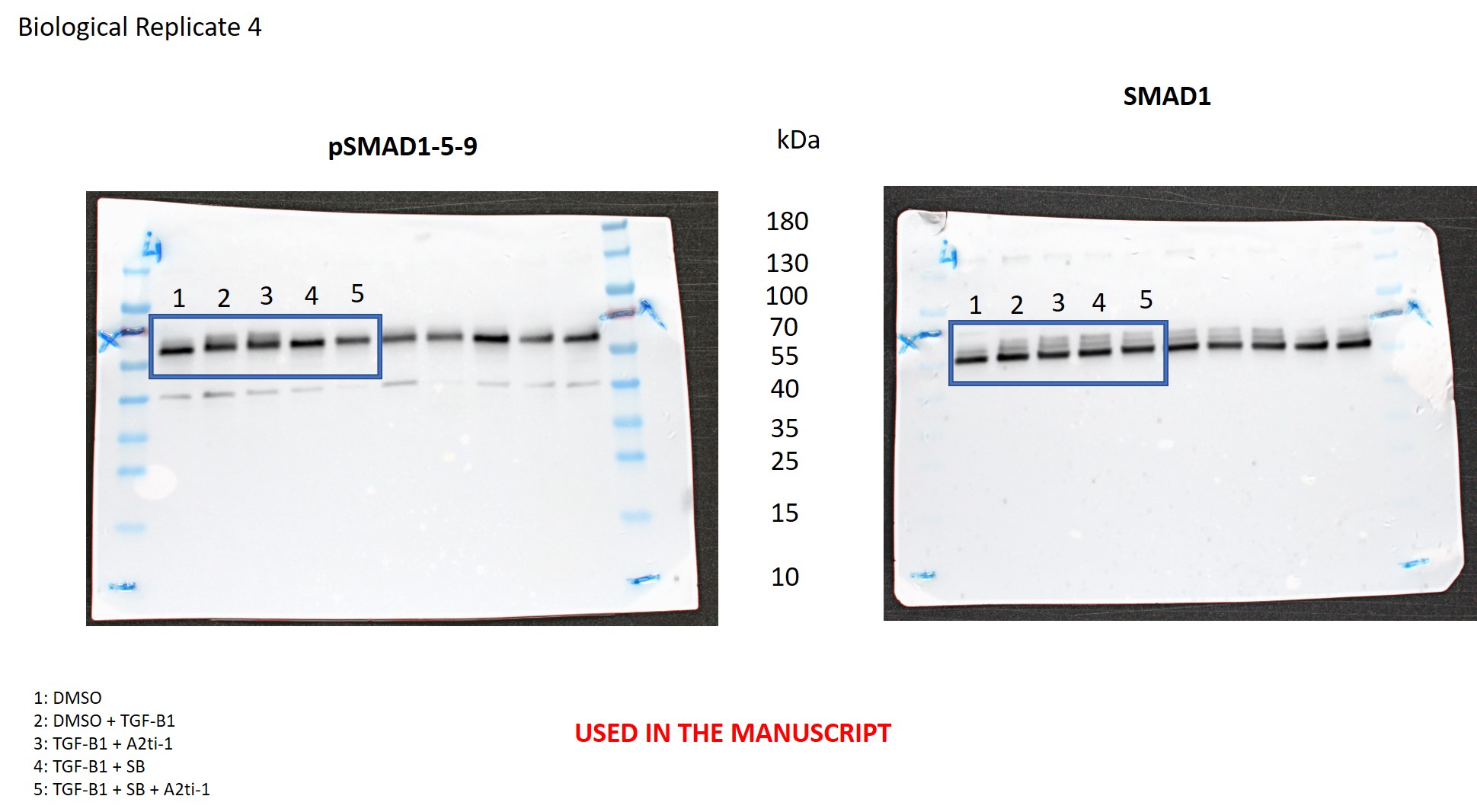

Supplement: Supplementary file 7 — Source data Fig. 5 [file 44321_2026_464_MOESM7_ESM.zip › Figure 5/Figure 5B_5C/Replicate 4 - pSMAD1-SMAD1.jpg]

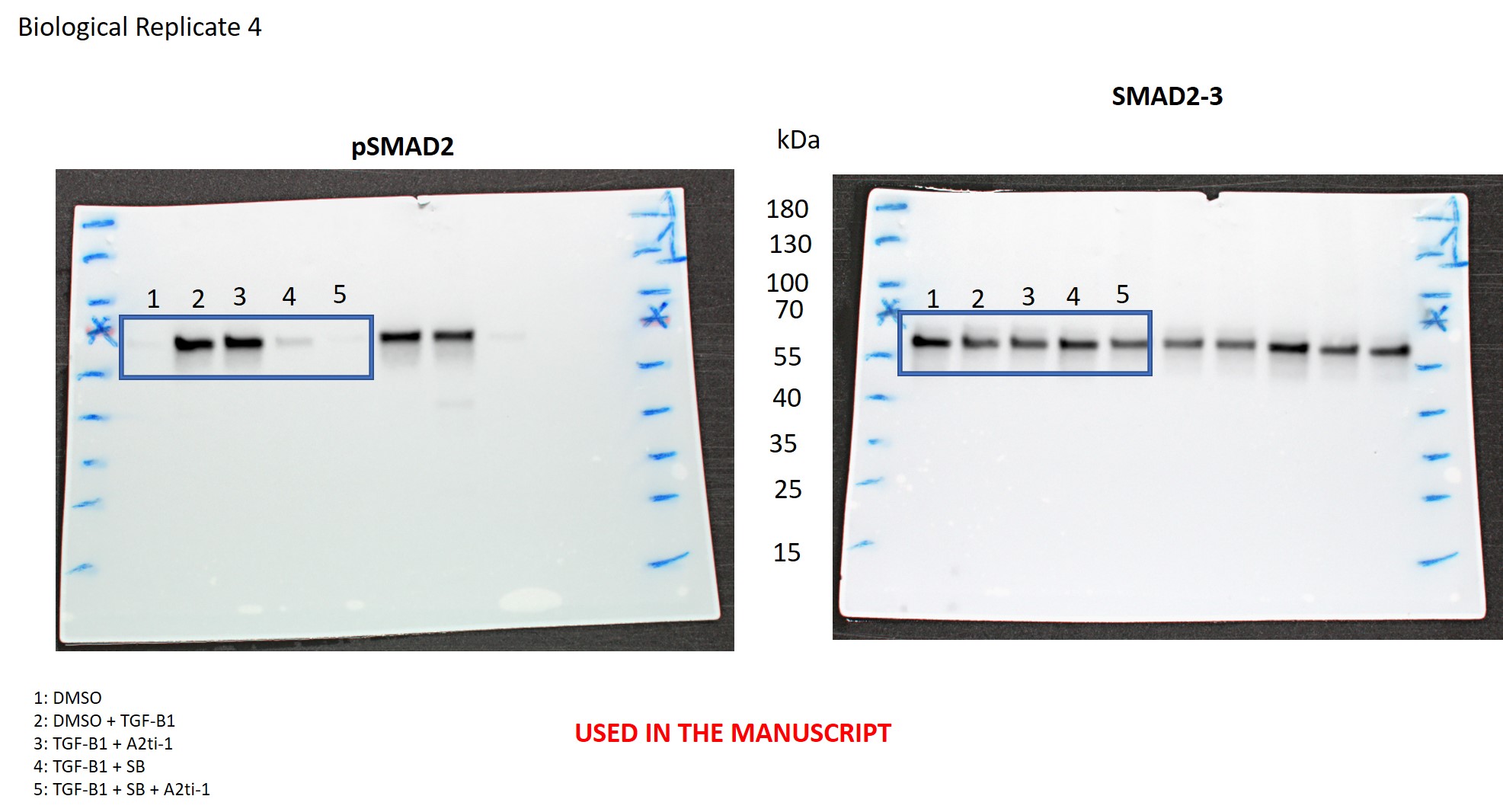

Supplement: Supplementary file 7 — Source data Fig. 5 [file 44321_2026_464_MOESM7_ESM.zip › Figure 5/Figure 5B_5C/Replicate 4 - pSMAD2-SMAD2.jpg]

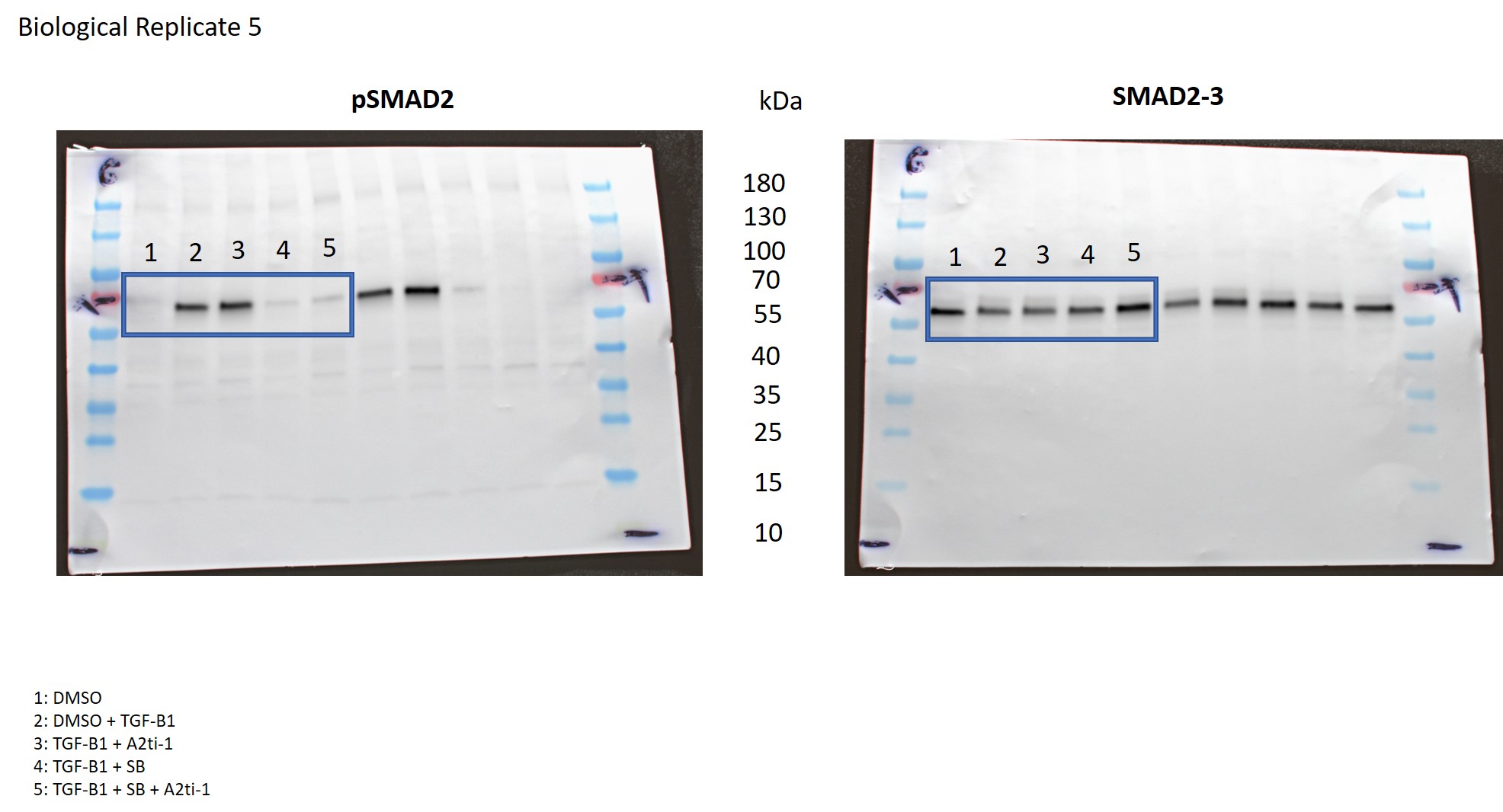

Supplement: Supplementary file 7 — Source data Fig. 5 [file 44321_2026_464_MOESM7_ESM.zip › Figure 5/Figure 5B_5C/Replicate 5 - pSMAD2-SMAD2.jpg]

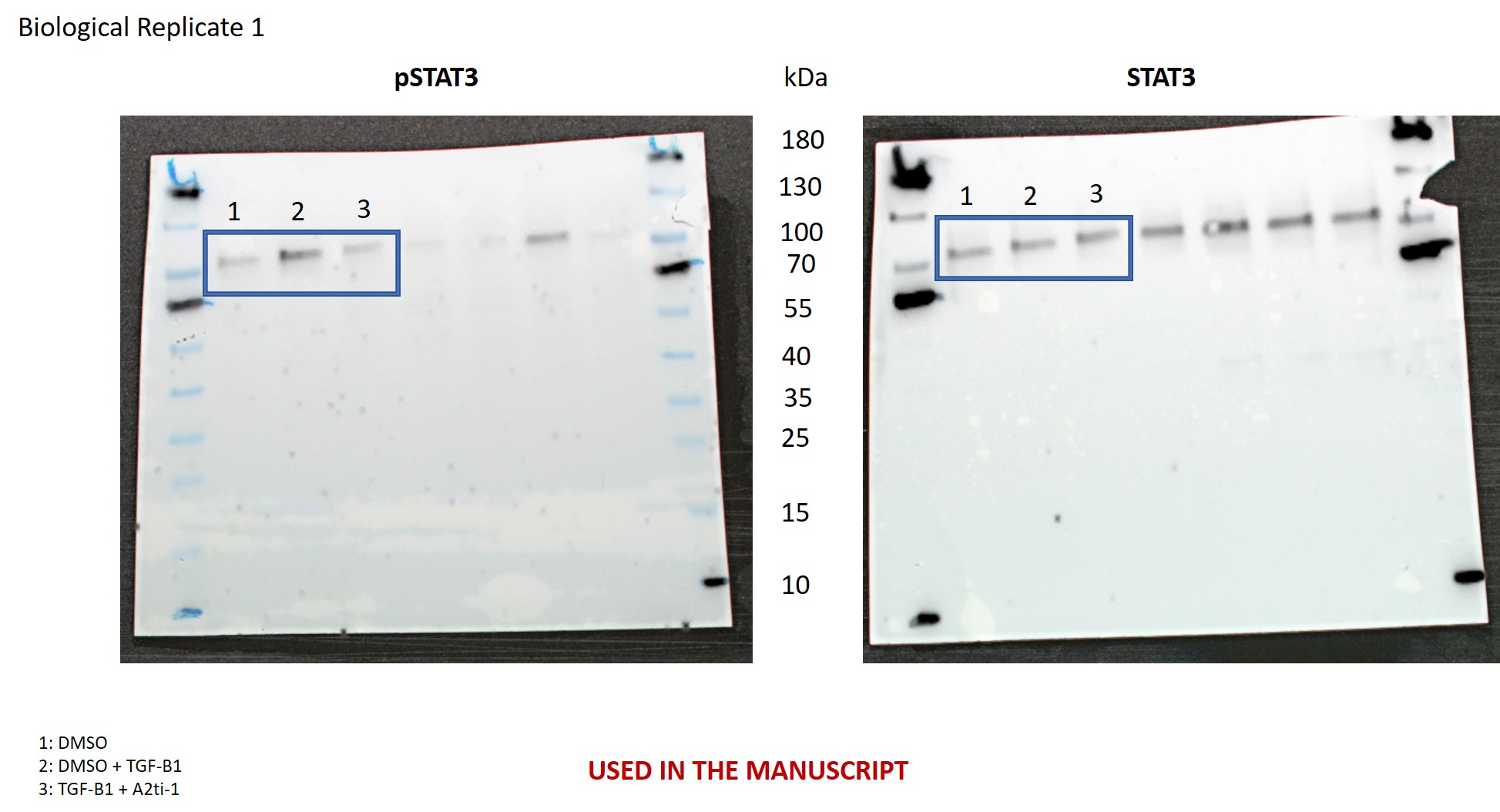

Supplement: Supplementary file 7 — Source data Fig. 5 [file 44321_2026_464_MOESM7_ESM.zip › Figure 5/Figure 5D/Replicate 1.jpg]

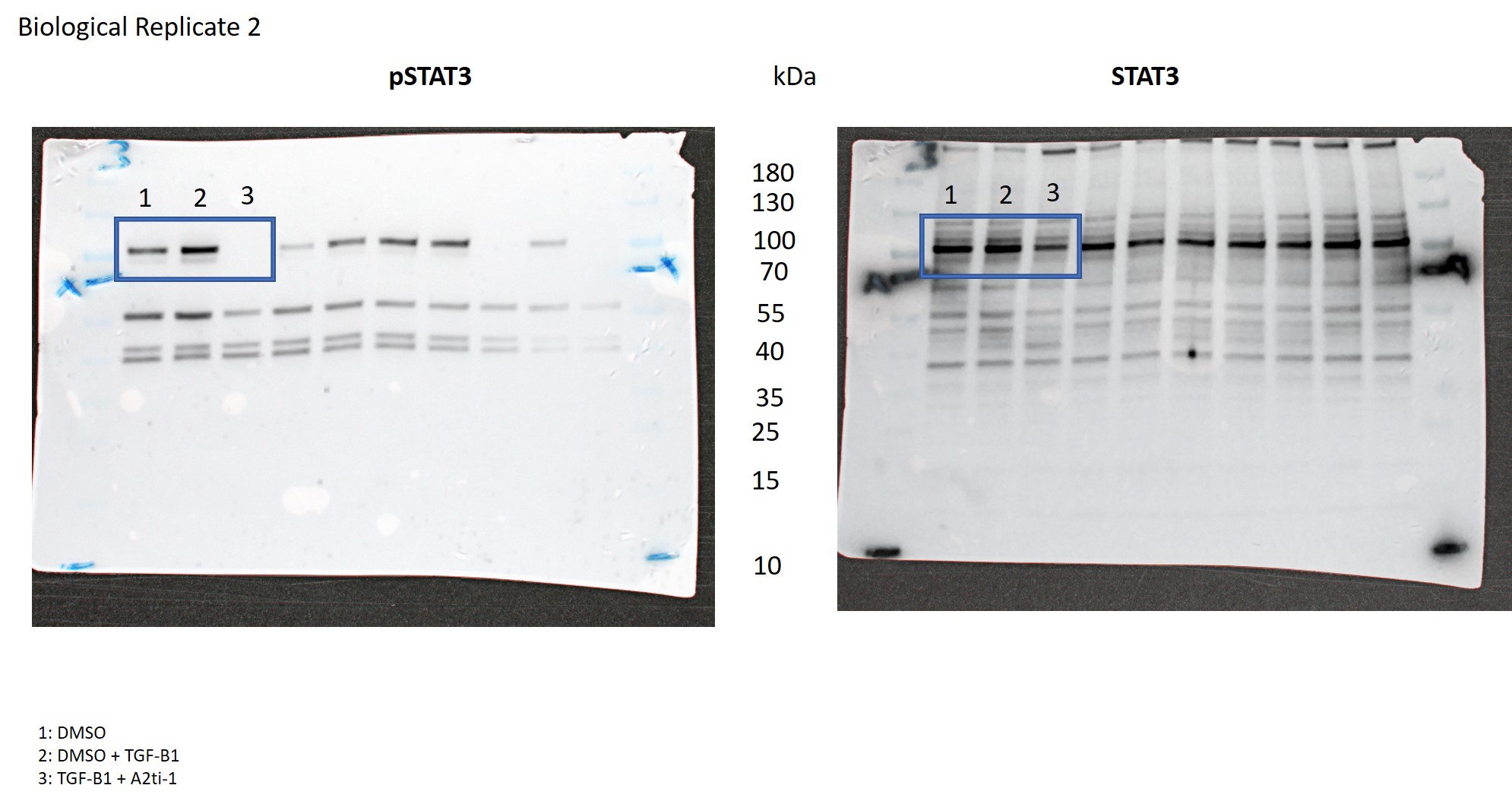

Supplement: Supplementary file 7 — Source data Fig. 5 [file 44321_2026_464_MOESM7_ESM.zip › Figure 5/Figure 5D/Replicate 2.jpg]

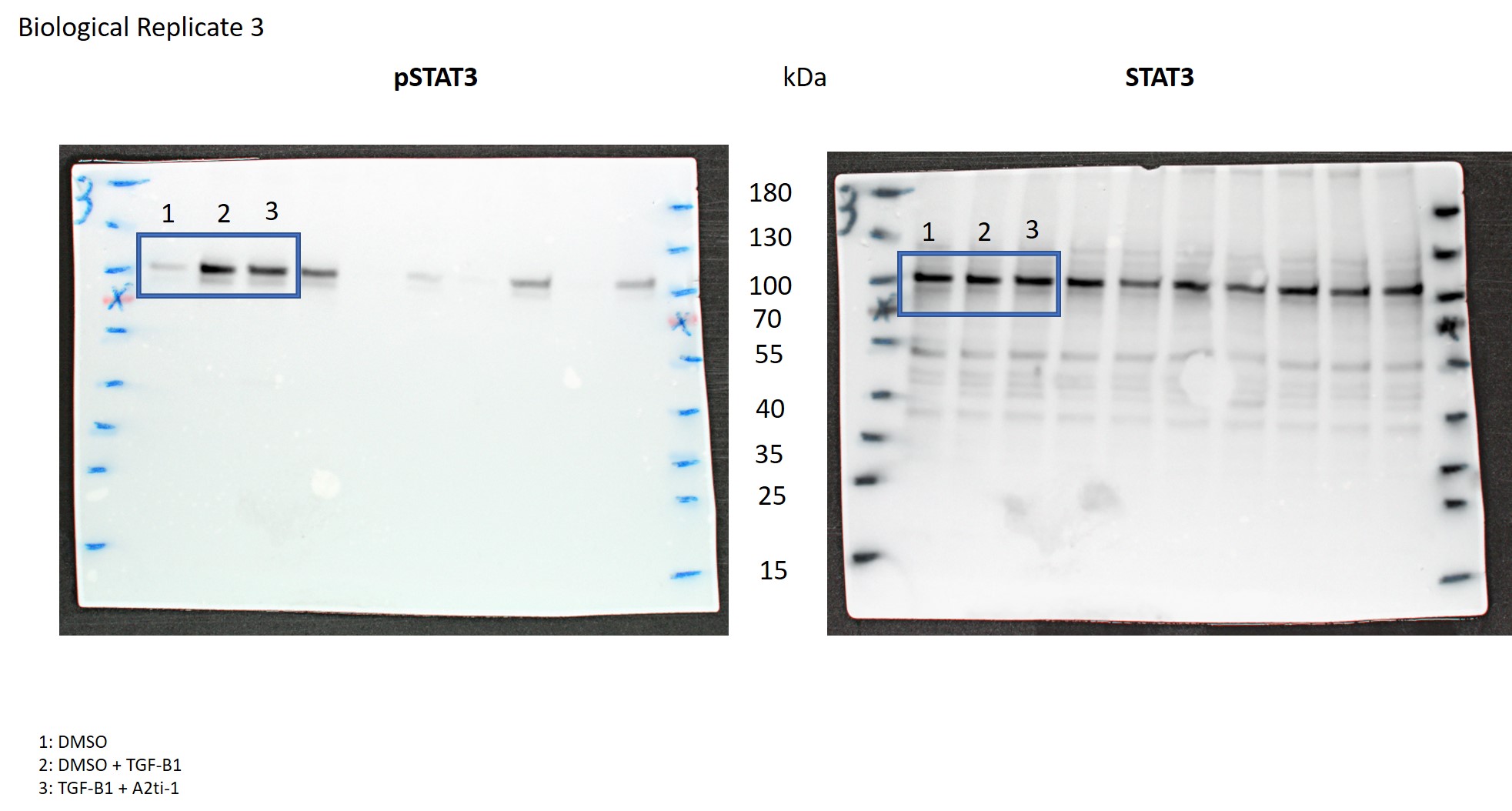

Supplement: Supplementary file 7 — Source data Fig. 5 [file 44321_2026_464_MOESM7_ESM.zip › Figure 5/Figure 5D/Replicate 3.jpg]

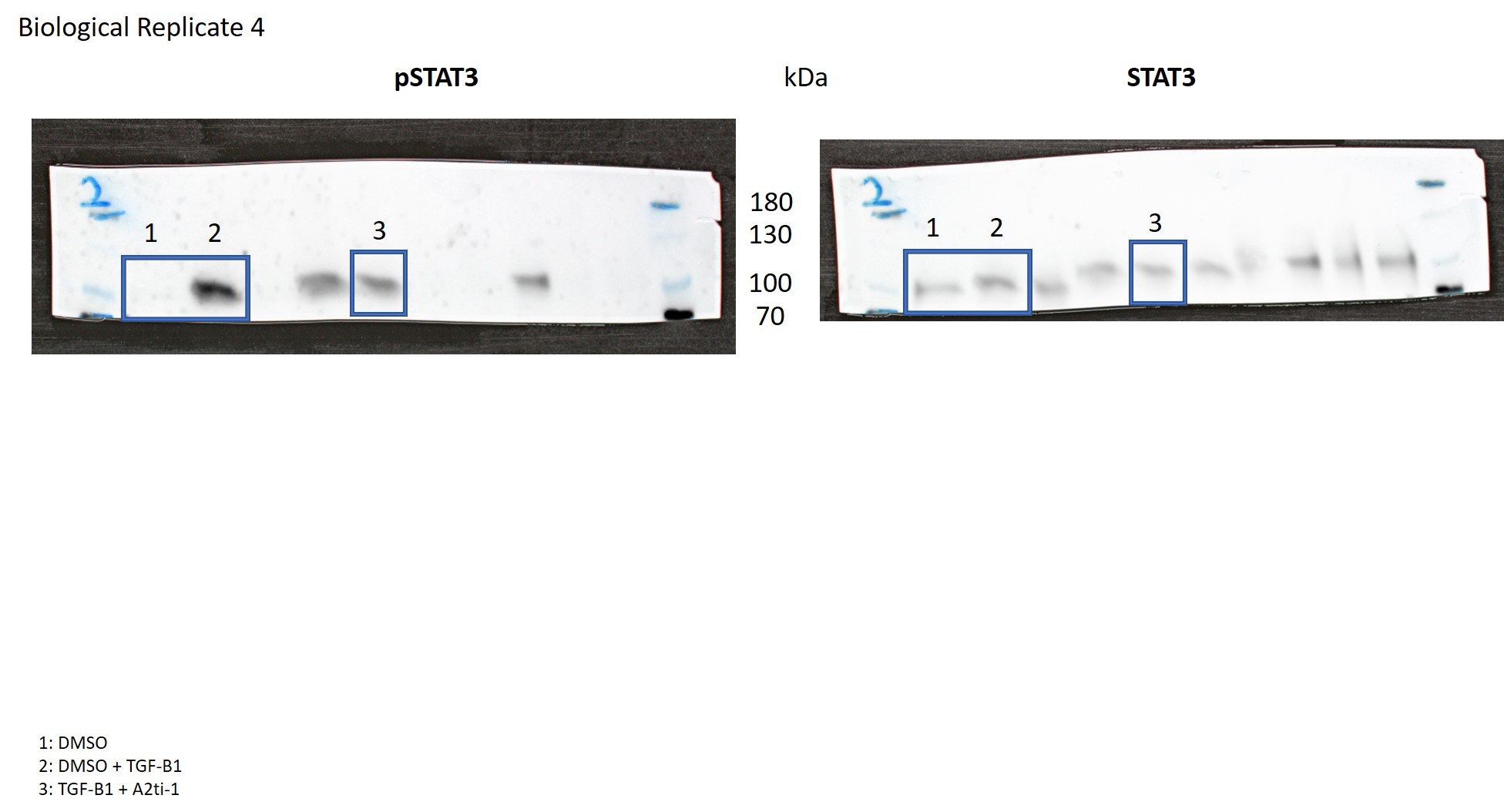

Supplement: Supplementary file 7 — Source data Fig. 5 [file 44321_2026_464_MOESM7_ESM.zip › Figure 5/Figure 5D/Replicate 4.jpg]

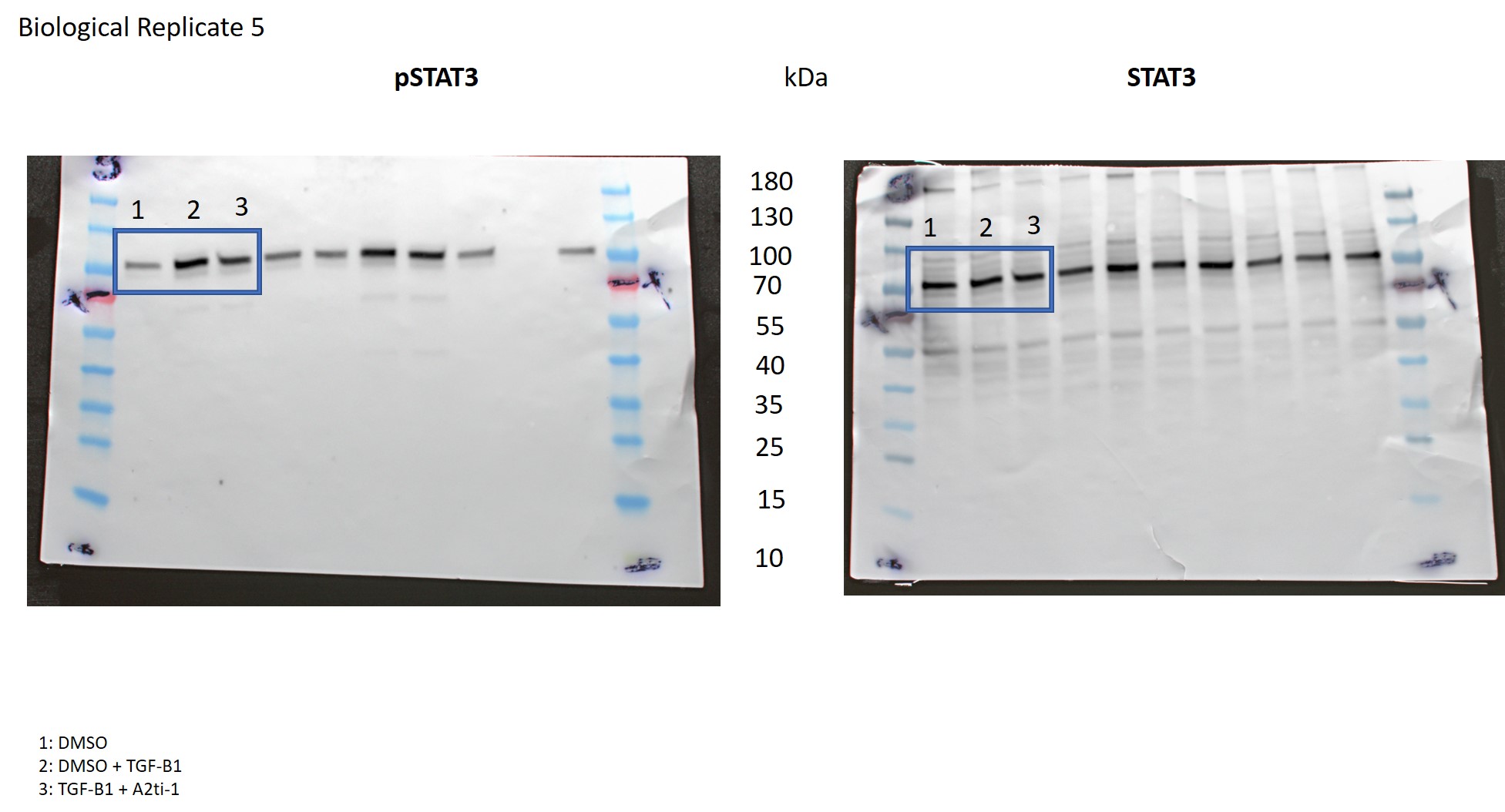

Supplement: Supplementary file 7 — Source data Fig. 5 [file 44321_2026_464_MOESM7_ESM.zip › Figure 5/Figure 5D/Replicate 5.jpg]
