# Supplementary material for: S100A10-ANXA2 tetramer inhibition hampers hepatic stellate cell activation in human MASLD organoids
Source: EMBO Mol Med. 2026 Jun 10;18(7):2920–45. doi: 10.1038/s44321-026-00464-y (PMC13365249; doi:10.1038/s44321-026-00464-y)
Supplement: Supplementary file 9 — Expanded View Figures [file 44321_2026_464_MOESM9_ESM.pdf]

Expanded View Figures

Figure EV1. The expression of ANXA2 and S100A10 in human hepatic stellate cells (HSCs) in vitro and in vivo.

(**A**) ANXA2 and S100A10 expression in primary human HSCs with or without TGF-β stimulation. Violin plots show normalized RNA-seq counts for ANXA2 and S100A10 in primary human HSCs reflecting the relative abundance of ANXA2 and S100A10 transcripts. Human HSCs cultured under solvent (Vehicle) or treated with TGF-β (10 ng/ml) for 24 h (Data ref: GEO GSE253493, Ma et al. 2024). Individual dots represent biological replicates ($n = 3$ per condition). Expression values are shown on a log10 scale. (**B**) Expression of ANXA2 and S100A10 in human hepatic stellate cells in vivo. Dot plots showing expression of ANXA2 and S100A10 in human HSCs across disease conditions (Healthy, MASLD, MASH), derived from single-nucleus RNA-sequencing data (Data ref: GEO GSE244832, 2025 (Kim et al, 2025)). Each dot represents the aggregated expression within the HSC population for a given condition. Dot size indicates the percentage of HSCs expressing the gene, while dot colour reflects average normalized expression level. (**C**) Automatic nuclei counting of LX-2 cells ($n = 3$) treated with increasing concentrations of A2ti-1 (12.5, 25 or 50 µM) for 2 days, measured on ImageXpress. Data are expressed as fold change versus Vehicle condition. Triplicates have been performed for each biological replicate ($n = 3$). (**D**) Representative Western blot (left) and quantification (right) of α-SMA levels in LX-2 cells treated with or without A2ti-1 (50 µM) for 2 days ($n = 6$). Tubulin is used as loading control. (**E**) Representative Western blot (left) and quantification (right) of α-SMA protein levels in LX-2 cells transfected 3 days with siRNA control (siCTL) or siRNA direct against S100A10 (siS100A10) treated with or without A2ti-1 (50 µM) and TGF-β1 (5 ng/ml) for 2 days ($n = 5$). Tubulin is used as loading control. Data are presented as mean ± SD, expressed as fold change relative to the indicated control where applicable. Statistical significance was assessed using Mann–Whitney test or one-way ANOVA followed by Tukey's multiple comparisons test. $^{**}P < 0.01$, $^{***}P < 0.001$, ns=not significant. "n" represents the number of independent experiments, performed at different passages.

© The Author(s)

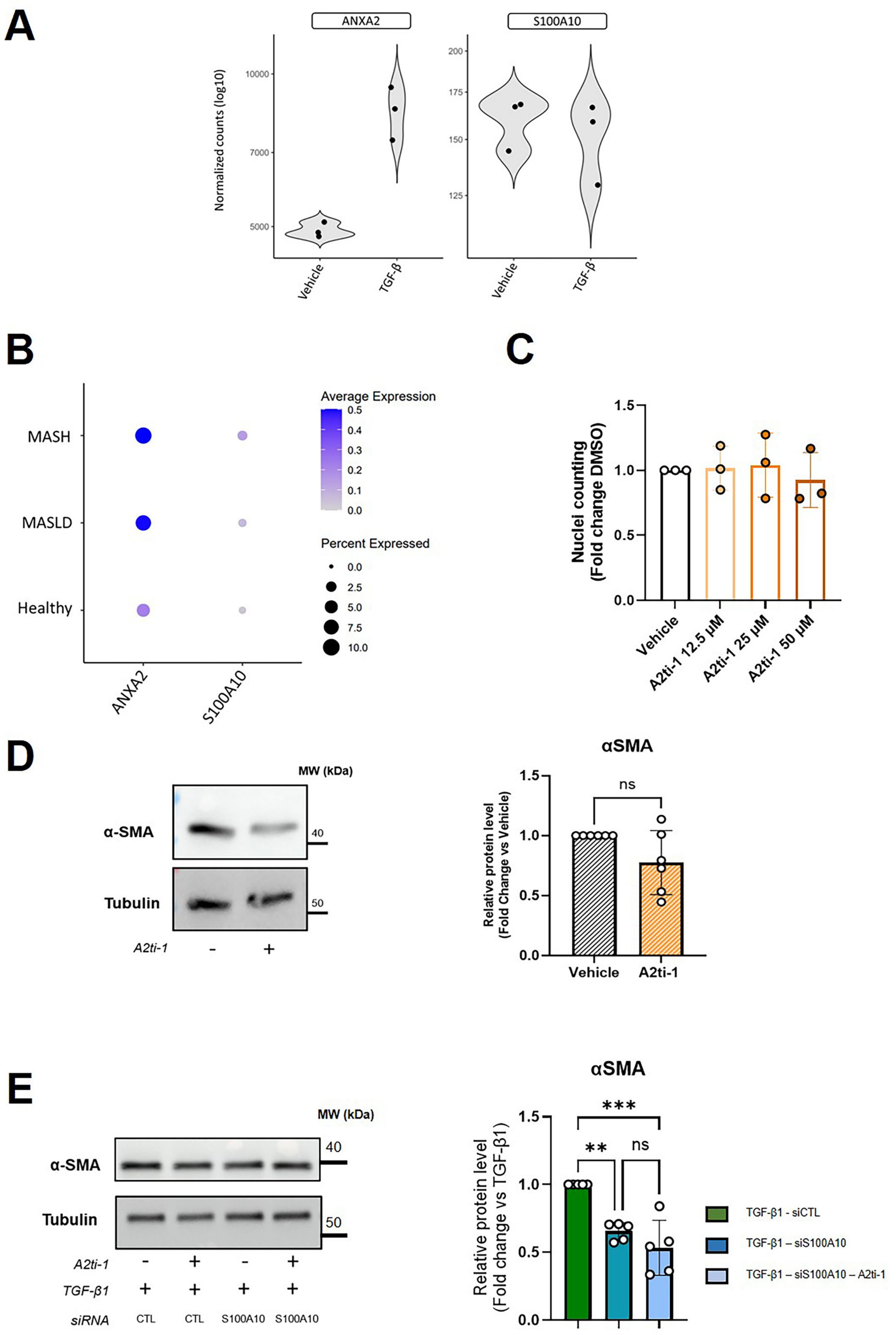
A
ANXA2
S100A10
Normalized counts (log10)
Vehicle
TGF-β
B
Average Expression
Percent Expressed
MASH
MASLD
Healthy
ANXA2
S100A10
C
Nuclei counting (Fold change DMSO)
Vehicle
A2ti-1 12.5 µM
A2ti-1 25 µM
A2ti-1 50 µM
D
MW (kDa)
α-SMA
Tubulin
A2ti-1
αSMA
ns
Relative protein level (Fold Change vs Vehicle)
Vehicle
A2ti-1
E
MW (kDa)
α-SMA
Tubulin
A2ti-1
TGF-β1
siRNA
CTL
S100A10
αSMA
***
**
ns
Relative protein level (Fold change vs TGF-β1)
TGF-β1 - siCTL
TGF-β1 – siS100A10
TGF-β1 – siS100A10 – A2ti-1

 © The Author(s)

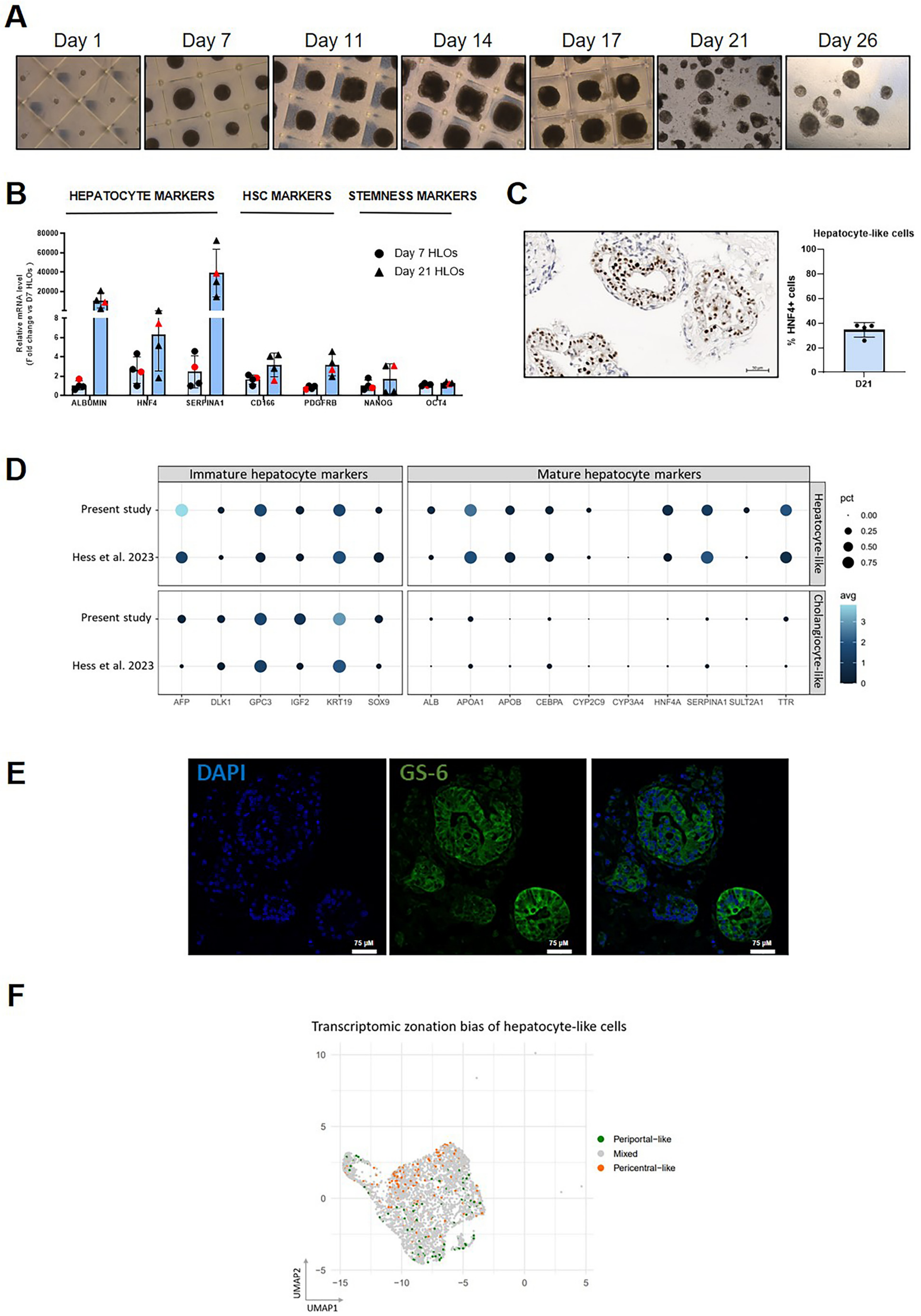
A
Day 1
Day 7
Day 11
Day 14
Day 17
Day 21
Day 26
B
HEPATOCYTE MARKERS
HSC MARKERS
STEMNESS MARKERS
Relative mRNA level (Fold change vs D7 HLOs)
Day 7 HLOs
Day 21 HLOs
ALBUMIN
HNF4
SERPINA1
CD166
PDGFRB
NANOG
OCT4
C
Hepatocyte-like cells
% HNF4+ cells
D21
D
Immature hepatocyte markers
Mature hepatocyte markers
Present study
Hess et al. 2023
Hepatocyte-like
Cholangiocyte-like
pct
avg
AFP
DLK1
GPC3
IGF2
KRT19
SOX9
ALB
APOA1
APOB
CEBPA
CYP2C9
CYP3A4
HNF4A
SERPINA1
SULT2A1
TTR
E
DAPI
GS-6
75 µM
F
Transcriptomic zonation bias of hepatocyte-like cells
Periportal-like
Mixed
Pericentral-like
UMAP1
UMAP2

Figure EV2. Generation and characterization of human liver organoids.

(**A**) Representative bright-field images (5X) showing different stages of HLO differentiation from human embryonic stem cells (hESCs). (**B**) Quantitative PCR (qPCR) analysis of different cell types and progenitor marker gene expression in Day 7 HLOs and Day 21 HLOs ($n = 4$). Expression values are shown as fold change relative to Day 7 HLOs. The red data point indicates the HLO sample used for single-cell RNA sequencing. Cyclophilin A and TBP was used as housekeeping genes. (**C**) Representative picture (left) and quantification (right) of HNF4 staining performed in HLOs after 21 days of differentiation ($n = 4$). Data are expressed as percentage of HNF4-positive cells on total number of cells. Images were taken at ×200 magnification. (**D**) Single-cell transcriptomic comparison of hepatocyte-like cells at day 21. Dot plot comparing the expression of representative immature/hepatoblast (AFP, DLK1, GPC3, IGF2, KRT19, SOX9) and mature hepatocyte markers (ALB, APOA1, APOB, CEBPA, CYP2C9, CYP3A4, HNF4A, SERPINA1, SULT2A1, TTR) in hepatocyte-like clusters from D21 HLOs generated in present study and from a previously published D21 HLO scRNA-seq dataset (Data ref: GEO GSE207889, 2023) (Hess et al, 2023). Cholangiocyte-like cells are included to demonstrate that hepatocyte markers are not ubiquitously expressed in the dataset. Dot size indicates the fraction of cells with detectable expression (expression >0), and colour indicates the mean log-normalized expression across hepatocyte-like cells. Datasets were analysed separately. One limitation of this comparison is that the datasets may not be normalized identically as we used the provided normalized values from Hess et al 2023. (**E**) Representative confocal images of Day 21 HLOs stained for GS-6 (green, pericentral hepatocyte marker) and Hoechst (blue, nuclei). Images were taken with Stellaris confocal microscope (Leica) at ×400 magnification. (**F**) UMAP visualization of hepatocyte-like cells coloured according to transcriptional zonation bias. A continuous porto-central coordinate was computed from single-cell RNA-seq data using module scores for periportal (zone 1) and pericentral (zone 3) gene signatures derived from human liver datasets (MacParland et al, 2018). The zonation axis represents the standardized difference between pericentral and periportal scores. Cells were classified using a stringent threshold (±1.75 SD) into periportal-like (green), pericentral-like (orange), or mixed (grey) populations. Data in (**B**, **C**) are presented as mean ± SD. "*n*" corresponds to an independent human liver organoid (HLO) batch.

 © The Author(s)

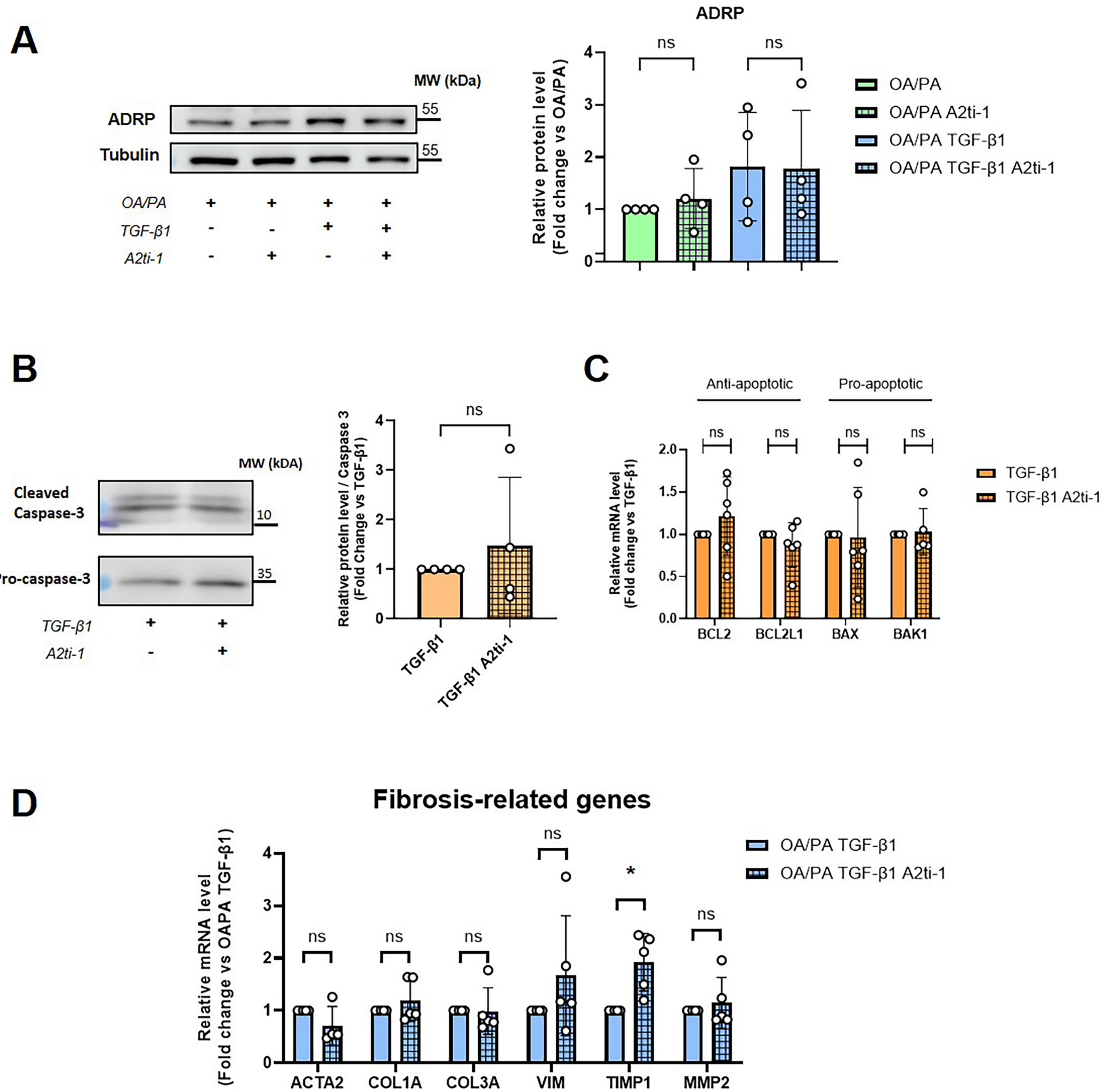

Figure EV3. Effect of A2ti-1 on steatosis and steatohepatitis modelling HLOs.

(**A**) Representative Western blot (left) and quantification (right) of ADRP protein level in HLOs treated with OA/PA (600 µM) ± TGF-β1 (20 ng/ml) and/or A2ti-1 (50 µM). Tubulin was used as a loading control ($n = 4$ for each group). (**B**) Representative Western blot (left) and quantification (right) of cleaved caspase 3 protein level in HLOs treated with TGF-β1 (20 ng/ml) with or without A2ti-1 (50 µM). Data are normalized on total caspase 3 levels ($n = 4$ for each group). (**C**) qPCR analysis of anti-apoptotic (BCL2 and BCL2L1) and pro-apoptotic genes (BAX and BAK1) in TGF-β1 (20 ng/ml) treated HLOs with or without A2ti-1 (50 µM). Cyclophilin A and TBP was used as housekeeping genes ($n = 6$ for each group). (**D**) qPCR analysis of fibrosis-related genes (ACTA2, COL1A1, COL3A1, VIM, TIMP1, MMP2) in OA/PA (600 µM) + TGF-β1 (20 ng/ml) treated HLOs ± A2ti-1 (50 µM). Cyclophilin A and TBP was used as housekeeping genes ($n = 5$ for each group). Technical triplicates have been performed for the qPCR. Data are presented as mean ± SD, expressed as fold change relative to the indicated control when applicable. Statistical significance was assessed using one-way ANOVA followed by Tukey's multiple comparisons test and unpaired *t* test or Mann–Whitney test when data did not pass normality assumptions. $^{**}P < 0.01$, ns = not significant. "*n*" corresponds to an independent human liver organoid (HLO) batch.

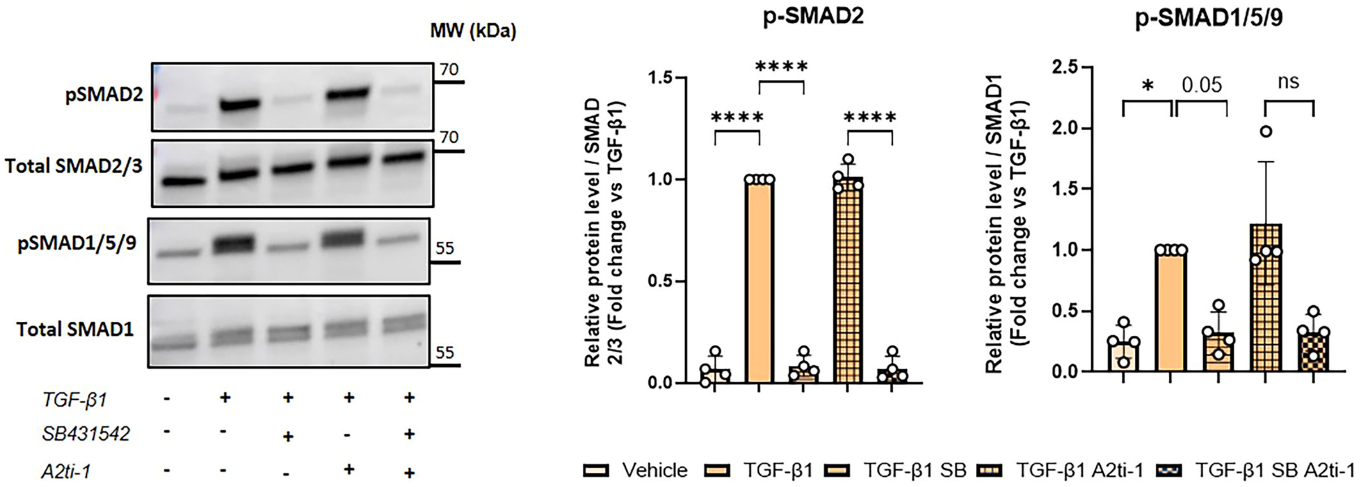

Figure EV4. Phosphorylation of SMAD proteins after 1 h TGF-β1 stimulation.

Western blot images (left) and quantification (right) of phosphorylated SMAD2 (pSMAD2) relative to total SMAD2/3 and phosphorylated SMAD1/5/9 (pSMAD1/5/9) relative to total SMAD1 in LX-2 cells treated with TGF-β1 (5 ng/ml) for 1 h in the presence or absence of A2ti-1 (50 μM) or SB431542 (5 μM). Data are presented as mean ± SD, expressed as fold change relative to the vehicle. Statistical significance was assessed one-way ANOVA followed by Sidak's multiple comparisons test, or Kruskal–Wallis followed by Dunn's multiple comparisons test when data did not pass normality assumptions. $*P < 0.05$, $**P < 0.01$, $****P < 0.0001$, ns = not significant ($n = 4$ for each group, "*n*" represents the number of independent experiments, performed at a different passage).

 © The Author(s)
