# Supplementary material for: S100A10-ANXA2 tetramer inhibition hampers hepatic stellate cell activation in human MASLD organoids
Source: EMBO Mol Med. 2026 Jun 10;18(7):2920–45. doi: 10.1038/s44321-026-00464-y (PMC13365249; doi:10.1038/s44321-026-00464-y)
Supplement: Supplementary file 2 — Peer Review File [file 44321_2026_464_MOESM2_ESM.pdf]

EMBO Molecular Medicine

S100A10-ANXA2 TETRAMER INHIBITION HAMPERS HEPATIC STELLATE CELL ACTIVATION IN HUMAN MASLD ORGANOIDS

Miranda Türkal, Christine Maeder, Marta Correia de Sousa, Margot Fournier, Sanae El-Harane, Monika Gjorgjieva, Michelangelo Foti, Pierre Maechler, and Etienne Delangre

Corresponding authors: Etienne Delangre (etienne.delangre@unige.ch) , Pierre Maechler (pierre.maechler@unige.ch)

Review Timeline:

Submission Date:	19th Dec 25
Editorial Decision:	14th Jan 26
Revision Received:	16th Apr 26
Editorial Decision:	28th Apr 26
Revision Received:	26th May 26
Accepted:	28th May 26

Editor: Zeljko Durdevic

Transaction Report:

(Note: With the exception of the correction of typographical or spelling errors that could be a source of ambiguity, letters and reports are not edited. Depending on transfer agreements, referee reports obtained elsewhere may or may not be included in this compilation. Referee reports are anonymous unless the Referee chooses to sign their reports.)

14th Jan 2026

Dear Dr. Delangre,

Thank you for the submission of your manuscript to EMBO Molecular Medicine. We have now received feedback from the three reviewers who agreed to evaluate your manuscript. All three referees recognize interest of the study but also raise serious concerns that should be addressed in a major revision. If you would like to discuss further the points raised by the referees, I am available to do so via email or video. Let me know if you are interested in this option.

Further consideration of a revision that addresses reviewers' concerns in full will entail a second round of review. EMBO Molecular Medicine encourages a single round of revision only and therefore, acceptance or rejection of the manuscript will depend on the completeness of your responses included in the next, final version of the manuscript. For this reason, and to save you from any frustrations in the end, I would strongly advise against returning an incomplete revision.

We would welcome the submission of a revised version within three months for further consideration. Please let us know if you require longer to complete the revision.

I look forward to receiving your revised manuscript.

Yours sincerely,

Zeljko Durdevic

Zeljko Durdevic
Senior Editor
EMBO Molecular Medicine

*****
When preparing your revised manuscript, please refer to our guidelines: https://link.springer.com/journal/44321/submission-guidelines#cms-Revised-submissions. We perform an initial quality control of all revised manuscripts before re-review; failure to include requested items will delay the evaluation of your revision.

We require:

1) A .docx formatted version of the manuscript text (including legends for main figures, EV figures and tables). Please make sure that the changes are highlighted to be clearly visible.

2) Individual production quality figure files as .eps, .tif, .jpg (one file per figure). For guidance, download the 'Figure Guide PDF': https://media.springernature.com/original/springer-cms/rest/v1/content/27825798/data/v1.

3) A .docx formatted letter INCLUDING the reviewers' reports and your detailed point-by-point responses to their comments. As part of the EMBO Press transparent editorial process, the point-by-point response is part of the Review Process File (RPF), which will be published alongside your paper.

4) A complete author checklist, which you can download from our author guidelines. Please insert information in the checklist that is also reflected in the manuscript. The completed author checklist will also be part of the RPF.

5) Please note that all corresponding authors are required to supply an ORCID ID for their name upon submission of a revised manuscript.

6) It is mandatory to include a 'Data Availability' section after the Materials and Methods. Before submitting your revision, primary datasets produced in this study need to be deposited in an appropriate public database, and the accession numbers and database listed under 'Data Availability'. Please remember to provide a reviewer password if the datasets are not yet public.

In case you have no data that requires deposition in a public database, please state so in this section. Note that the Data Availability Section is restricted to new primary data that are part of this study.

7) For data quantification: please specify the name of the statistical test used to generate error bars and P values, the number (n) of independent experiments (specify technical or biological replicates) underlying each data point and the test used to calculate p-values in each figure legend. The figure legends should contain a basic description of n, P and the test applied. Graphs must include a description of the bars and the error bars (s.d., s.e.m.).

8) At EMBO Press we ask authors to provide source data for the main manuscript figures. You will receive a separate email with instructions for providing source data with your revised manuscript, including how to upload and organize the files.

9) Our journal encourages inclusion of *data citations in the reference list* to directly cite datasets that were re-used and obtained from public databases. Data citations in the article text are distinct from normal bibliographical citations and should directly link to the database records from which the data can be accessed. In the main text, data citations are formatted as follows: "Data ref: Smith et al, 2001" or "Data ref: NCBI Sequence Read Archive PRJNA342805, 2017". In the Reference list, data citations must be labeled with "[DATASET]". A data reference must provide the database name, accession number/identifiers and a resolvable link to the landing page from which the data can be accessed at the end of the reference.

10) We replaced Supplementary Information with Expanded View (EV) Figures and Tables that are collapsible/expandable online. A maximum of 5 EV Figures can be typeset. EV Figures should be cited as 'Figure EV1, Figure EV2" etc... in the text and their respective legends should be included in the main text after the legends of regular figures.

- For the figures that you do NOT wish to display as Expanded View figures, they should be bundled together with their legends in a single PDF file called *Appendix*, which should start with a short Table of Content. Appendix figures should be referred to in the main text as: "Appendix Figure S1, Appendix Figure S2" etc.

- Additional Tables/Datasets should be labeled and referred to as Table EV1, Dataset EV1, etc. Legends have to be provided in a separate tab in case of .xls files. Alternatively, the legend can be supplied as a separate text file (README) and zipped together with the Table/Dataset file.

11) The paper explained: EMBO Molecular Medicine articles are accompanied by a summary of the articles to emphasize the major findings in the paper and their medical implications for the non-specialist reader. Please provide a draft summary of your article highlighting

- the medical issue you are addressing,

- the results obtained and

- their clinical impact.

This may be edited to ensure that readers understand the significance and context of the research. Please refer to any of our published articles for an example.

12) Author contributions: You will be asked to provide CRediT (Contributor Role Taxonomy) terms in the submission system. These replace a narrative author contribution section in the manuscript.

13) A Conflict of Interest statement should be provided in the main text.

14) Every published paper includes a 'Synopsis' to further enhance discoverability. Synopses are displayed on the journal webpage and are freely accessible to all readers. They include a short stand first (maximum of 300 characters, including space) as well as 2-5 one-sentences bullet points that summarizes the paper. Please write the bullet points to summarize the key NEW findings. They should be designed to be complementary to the abstract - i.e. not repeat the same text. We encourage inclusion of key acronyms and quantitative information (maximum of 30 words / bullet point). Please use the passive voice. Please attach these in a separate file or send them by email, we will incorporate them accordingly.

Please also suggest a striking image or visual abstract to illustrate your article as a PNG file 550 px wide x 300-600 px high.

15) Include a Reagents and Tools Table as part of the Methods section, which can be downloaded from our author guidelines.

EMBO Molecular Medicine has a "scooping protection" policy, whereby similar findings that are published by others during review or revision are not a criterion for rejection. Should you decide to submit a revised version, I do ask that you get in touch after three months if you have not completed it, to update us on the status.

***** Reviewer's comments *****

Referee #1 (Comments on Novelty/Model System for Author):

The authors used human cell systems to explore human liver fibrosis

Referee #1 (Remarks for Author):

This manuscript by Türkal et al. highlights the relevance of the Annexin A2-S100A10 tetramer (A2t) in the development of MASLD/MASH and identifies disruption of A2t as a promising anti-fibrotic strategy.

First, I would like to apologize to the authors for the delayed review.

Overall, this is a very well-written manuscript, and I enjoyed reading it. The figures are not labeled (likely due to EMBO formatting requirements), which makes it somewhat challenging to follow all experimental details. Nevertheless, the authors have done an excellent job, and the clarity of the writing strongly highlights the main strengths of the study, namely:

1. The demonstration of an attenuating effect of the S100A10-Annexin A2 tetramer (A2t) on TGF-β1-induced fibrotic activation in hepatic stellate cells.
2. The use of human liver organoids to investigate this phenomenon and explore potential underlying mechanisms.

The study is carefully executed, and I have only two suggestions, neither of which should be considered mandatory. I do not have extensive expertise in the use of liver organoids, and therefore it would not be appropriate to propose an extensive list of additional experiments. However, the authors may wish to consider increasing the number of biological replicates for some experiments that were performed only three or four times, where feasible, to further strengthen the robustness of the conclusions.

In addition, as the authors note, an important next step would be to investigate the anti-fibrotic effects of A2t disruption in vivo. It would be helpful if the authors could clarify what is meant by this in the context of the current study-for example, whether they envision future preclinical models or more translational approaches. Alternatively, the authors might consider whether a relatively straightforward in vivo or ex vivo experiment could be incorporated to further strengthen the manuscript.

Referee #2 (Remarks for Author):

In this manuscript by Turkal et al. titled "S100A10-ANXA2 TETRAMER INHIBITION HAMPERS HEPATIC STELLATE CELL ACTIVATION IN HUMAN MASLD ORGANOIDS", the authors investigated the role of the S100A10-ANXA2 tetramer in driving MASH fibrosis using multilineage human liver organoids (HLO). The authors first reported that the S100A10-ANXA2 heteromerization inhibitor A2Ti-1 inhibited HSC activation in cultured LX2 cells, similar to siRNA knockdown of S100A10. Switching to an HLO system of MASH, the authors shows that induction of MASH in this system with OA/PA + TGFB1 induced ANXA2 expression just like reported in patients. A2Ti-1 treatment of MASH HLO led to reduced HSC activation and fibrosis but not steatosis. Mechanistic exploration in LX2 cells suggest A2Ti-1 inhibit HSC activation by blocking STAT3 phosphorylation activation. Overall this study further advances the use of multilineage HLO to model MASH fibrosis and support S100A10-ANXA2 as a driver of MASH fibrosis which can be inhibited with the A2Ti-1 compound. Although the results are novel and important, there are several major concerns regarding the physiological relevance of the disease model and potential off-target effects of the A2Ti-1 compound that prevents a straightforward interpretation of the results and current conclusions that needs to be addressed.

Major comments:
-Previous reports has evolved around a role of S100A10 and ANXA2 as disease drivers in hepatocytes. It is unclear if S100A10 and ANXA2 are expressed in HSCs in vivo, and how their expression pattern changes with disease. This needs to be addressed either by mining existing single cell/nucleus datasets from mice or patients, or using qPCR/Westernblot from isolated primary HSCs.
-Although A2Ti-1 has shown clear antifibrotic effect in MASH HLOs, it is unclear if the effect is on target. I.e. Is A2Ti-1's effect attributable to its ability to block S100A10-ANXA2 heteromerization? This can be addressed by testing the effect of this drug on MASH HLOs in the presence or absence of S100A10 and/or ANXA2 expression, as has been performed in LX2 cells. Alternatively, the authors can directly show their heteromerization through co-immunoprecipitation experiments.

-What is the toxicity of the A2Ti-1 compound? A proper dose-response study coupled with a cell viability measurements needs to be performed for cell lines and organoids for this compound to rule out cytotoxicity as an alternative explanation for the changes detected.
-The scRNAseq data from 21d HLO is significantly underexplored. As the establishment of MASH-like HLOs (Figures 2 and 3) is a major point of the manuscript, the authors should compare their results to prior MASH-like HLOs, especially the ones that informed the advancements reported in this paper. As achieving mature cells is a major challenge and goal of developing HLOs, how do the maturity of different cell types detected by scRNAseq compare to previously reported for HLOs (doi: 10.15252/embj.2023113898), and adult human liver (many publicly available datasets).
-The authors report expression of zone 3 marker GS in their HLOs. This begs the question whether zone 1 markers are also expressed in these HLOs or is it zone 3 specific?

Minor comment:
-Fig1B, what is the effect of A2Ti-1 on LX2 cells alone? LX2 cells are highly activated already so A2Ti-1 may already have an antifibrotic effect. Or is there only an antifibrotic effect in the presence of TGFb1 stimulation?

Referee #3 (Remarks for Author):

In this manuscript, Miranda et al. demonstrate that the S100A10-ANXA2 heterotetramer plays a critical role in liver fibrosis and provide evidence from in vitro cell lines and liver organoids that the inhibitor A2ti-1 ameliorates fibrotic phenotypes by downregulating α-SMA and pro-fibrotic gene expression. Mechanistic analyses further suggest that A2ti-1 may exert its effects through suppression of the STAT3 signaling pathway. While these findings hold potential clinical implications, several key issues warrant clarification to strengthen the study's rigor and interpretative depth.

1.Figure 1D reveals that siRNA-mediated knockdown of S100A10 leads to increased mRNA levels of VIM and TIMP1 under TGF-β1 stimulation compared to controls, while COL1A1 and COL3A1 expression remains unchanged-findings that contrast with those observed following A2ti-1 treatment (Fig. 1B). This inconsistency implies either the involvement of A2T-independent regulatory functions of S100A10 or the induction of compensatory adaptive responses due to partial gene silencing. To minimize off-target effects and better reflect functional loss, it is advisable to employ CRISPR/Cas9-mediated S100A10 knockout.

2.The authors propose that A2ti-1 exerts anti-fibrotic effects by inhibiting STAT3 phosphorylation. However, the mechanistic link between the S100A10-ANXA2 heterotetramer (A2t) and the STAT3 pathway remains inadequately explored. Specifically, it is unclear whether A2t directly interacts with STAT3 or JAK, or alternatively modulates downstream signaling via regulation of membrane receptor dynamics-such as PDGFRβrecruitment or endocytosis. The absence of direct experimental validation for such interactions undermines the conclusion that the A2t-STAT3-hepatic stellate cell axis constitutes a central regulatory mechanism in liver fibrosis. Further mechanistic investigation is therefore recommended to substantiate this proposed signaling cascade.

3.A hallmark feature of metabolic dysfunction-associated steatohepatitis (MASH) is immune cell-driven inflammation, particularly mediated by macrophages. Notably, the hepatic liver organoids (HLOs) employed in this study appear to lack immune components, a point corroborated by single-cell RNA sequencing data (Fig. 2B). Consequently, the cellular source of IL-6 within the culture system requires clarification: is it derived from stromal cells or injured hepatocytes? Although TGFβand OA/PA robustly induce IL-6 production (Fig. 3J), no reduction in IL-6 levels is observed upon A2ti-1 treatment (Fig. 4F), suggesting an unresolved regulatory mechanism. This discrepancy necessitates further investigation to elucidate the pathways involved.

4.Single-cell RNA sequencing was performed on only one sample derived from a single HLO differentiation batch. Although qPCR confirmed similar marker expression patterns across batches, this supports consistency in differentiation trajectory but does not account for potential batch-to-batch variability in cell type proportions or subpopulation heterogeneity. Thus, extrapolating cluster analysis from a single sample as representative of the "standard" HLO composition may compromise generalizability. Moreover, the omission of an A2ti-1-treated group in scRNA-seq limits the ability to assess drug-induced phenotypic shifts in distinct cell populations. Including such data would significantly enhance mechanistic insights into A2ti-1's mode of action.

5.In Figure 4, A2ti-1 treatment markedly reduces α-SMA protein levels in the OA/PA+TGF-β1 model (Fig. 4C), yet shows no significant effect on ACTA2, COL1A1, or COL3A1 mRNA levels (Supplementary Fig. 2B). In contrast, under TGF-β1-only conditions, A2ti-1 consistently downregulates both α-SMA protein and pro-fibrotic transcripts. This differential response represents a notable inconsistency that is neither acknowledged nor explained in the current text. Clarification regarding the underlying molecular mechanisms responsible for this context-dependent effect is essential.

6.Additionally, the nomenclature for fatty acid mixtures varies throughout the manuscript-including "oleic and palmitic acids (OA/PA)" , "OAPA"-which, while interpretable, lacks terminological consistency. Standardized usage should be adopted across all figures and text. Furthermore, although statistical analysis in Fig. 3K indicates a significant increase in ANXA2 expression, the corresponding Western blot image does not clearly reflect this difference; thus, inclusion of a more representative blot is recommended. Finally, the selection of A2ti-1 concentration lacks justification. It is advised to supplement the study with a dose-response experiment (e.g., 0, 12.5, 25, 50μM) to systematically evaluate the inhibitory efficacy of A2ti-1 on TGF-β1-induced α-SMA expression.

Reviewers’ Comments:

Referee #1:

1) The study is carefully executed, and I have only two suggestions, neither of which should be considered mandatory. I do not have extensive expertise in the use of liver organoids, and therefore it would not be appropriate to propose an extensive list of additional experiments. However, the authors may wish to consider increasing the number of biological replicates for some experiments that were performed only three or four times, where feasible, to further strengthen the robustness of the conclusions.

Following the suggestion of the Reviewer, where feasible, we have increased the number of biological replicates to further strengthen the take home message. Specifically, we increased the sample size for the experiments presented in Figure 1A and Figure 1C. In addition, analyses in Figure 1A have been extended with a dose-response protocol for α-SMA levels, as proposed by Reviewer 3. The additional data are fully consistent with the original findings and reinforce the conclusions drawn from these experiments. These new figures are incorporated in the Revised Manuscript and are presented in the Rebuttal Letter in **Figure R1**.

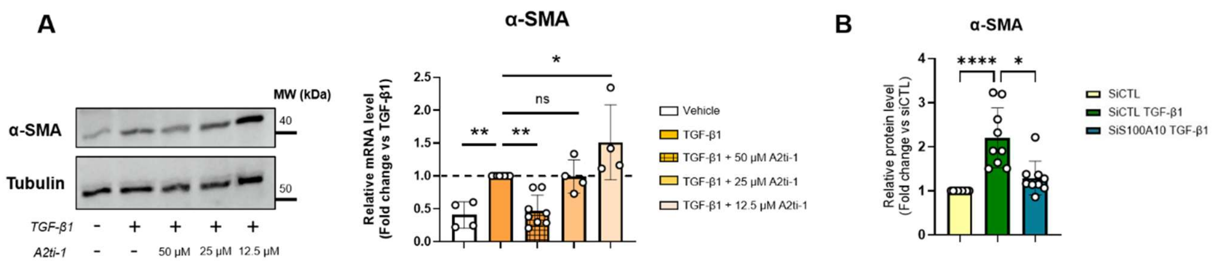

Figure R1: (A) Representative Western blot (left) and quantification (right) of α-SMA protein levels in LX-2 cells treated with TGF-β1 (5ng/ml) for 2 days in the absence or presence of A2ti-1 (12.5 µM to 50µM). Tubulin served as a loading control (n=4 to 8). **(B)** Western blot quantification of α-SMA protein levels in LX-2 cells transfected with control siRNA (siCTL) or S100A10-targeting siRNA (siS100A10) prior TGF-β1 (5 ng/mL) treatment. Tubulin served as loading control (n=9).

2) In addition, as the authors note, an important next step would be to investigate the anti-fibrotic effects of A2t disruption in vivo. It would be helpful if the authors could clarify what is meant by this in the context of the current study-for example, whether they envision future preclinical models or more translational approaches. Alternatively, the authors might consider whether a relatively straightforward in vivo or ex vivo experiment could be incorporated to further strengthen the manuscript.

We thank the Reviewer for this comment and fully agree that investigating the anti-fibrotic effects of A2t disruption in vivo represents an important next step toward its translational evaluation, as mentioned in the Discussion section line 468:

“Nevertheless, further studies should assess the pharmacokinetics, specificity, and safety profile of A2ti-1 and evaluate its therapeutic efficacy in vivo.”

To be fully transparent, we performed a pilot *in vivo* preclinical approach by treating a mouse model with A2ti-1. However, this approach revealed serious technical limitations related to compound solubility/biodistribution, thereby preventing acquisition of reliable conclusions.

Practically, in this *in vivo* protocol, we fed 3-month-old male C57BL/6J mice a choline-deficient, L-amino acid-defined, high-fat diet (CDAHFD) for 7 weeks, a well-established model that induces steatosis, inflammation and fibrosis [1, 2]. A2ti-1 was administered intraperitoneally at 8 mg/kg every other day, based on previously reported dosing regimens [3, 4]. At the experimental endpoint, mice were sacrificed and liver and serum were collected for compound detection. Despite these efforts, high-performance liquid chromatography (HPLC) analysis revealed no detectable levels of administered A2ti-1, neither in liver tissue nor in serum (**Figure R2, for Reviewer only**). We believe this is most likely due to the hydrophobicity of A2ti-1, which might limit its solubility (as grossly observed in the syringes) and thus its systemic distribution and hepatic uptake under the conditions used. As a result, we were unable to achieve sufficient *in vivo* exposure to assess or not anti-fibrotic efficacy in this model.

We believe that future studies using improved formulation strategies (e.g., nanoparticle-based delivery, alternative routes of administration, or chemical-supported solubilization) will be essential to fully evaluate the *in vivo* therapeutic potential of A2t inhibition.

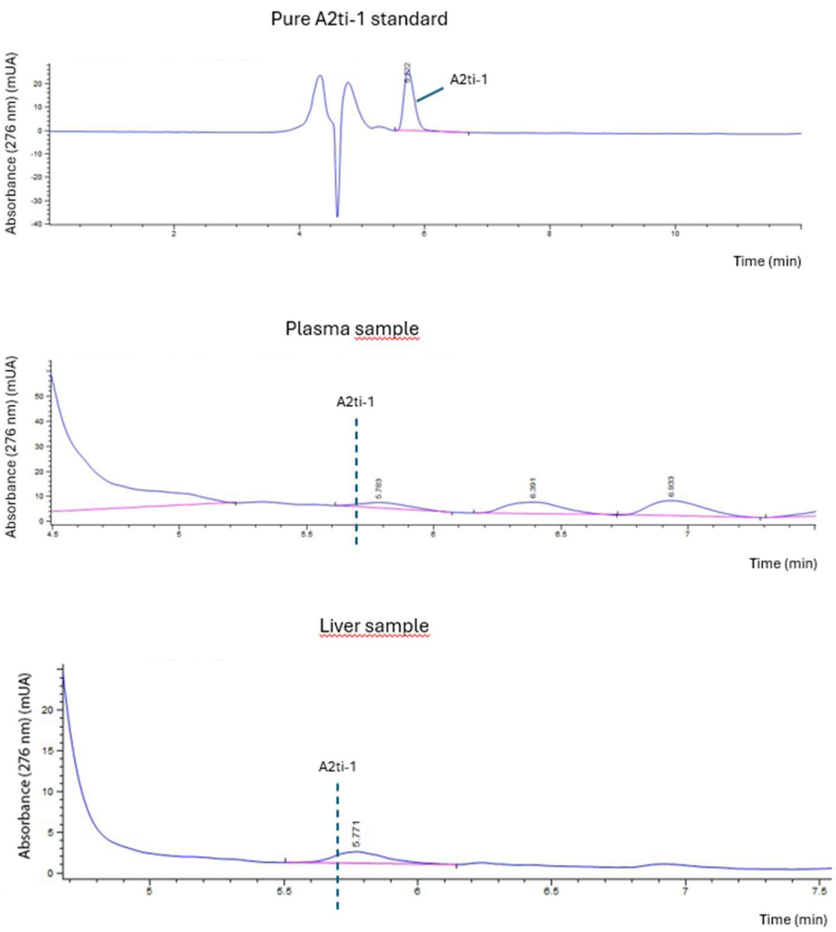

Figure R2: High-performance liquid chromatography (HPLC) analysis of plasma and liver tissue collected from mice treated with A2ti-1. The retention time of the A2ti-1 standard is indicated by the dashed line. No corresponding peak was detected at this retention time in either plasma or liver samples, indicating that A2ti-1 was below the limit of detection under the conditions tested. Additional peaks in A2ti-1 standard between 4 and 5 min belong to its solvent DMSO. Analyses were performed using an Agilent 1100/1260 HPLC system equipped with a quaternary pump, autosampler, and diode array detector.

Referee #2:

Major comments:

1.Previous reports has evolved around a role of S100A10 and ANXA2 as disease drivers in hepatocytes. It is unclear if S100A10 and ANXA2 are expressed in HSCs in vivo, and how their expression pattern changes with disease. This needs to be addressed either by mining existing single cell/nucleus datasets from mice or patients, or using qPCR/Westernblot from isolated primary HSCs.

We concur with the Reviewer that independent implication of S100A10 or ANXA2 in hepatic fibrosis has been described so far through a hepatocyte-dependent mechanism in the

literature. The expression and regulation of S100A10 and ANXA2 in hepatic stellate cells (HSCs) remain indeed poorly characterized.

To directly demonstrate that these genes are expressed in human HSCs and whether their expression changes with disease progression, we further analyzed an independent human single-nucleus RNA-sequencing (snRNA-seq) dataset (Data ref: GEO GSE244832, 2025 [5]), comprising liver samples from healthy subjects and patients suffering from MASLD or MASH. In this dataset, 18 human liver samples have been profiled using snRNA-seq. Consistent with the referenced study, we identified HSC population using canonical gene markers curated from the Panglao database. This analysis confirmed a single cluster with the highest enrichment of canonical HSC markers (including LRAT, RBP1, CYGB, COL1A1, and PDGFRB), which we subsequently defined as the HSC population for downstream analyses.

Within the HSC population, we examined the expression of S100A10 and ANXA2 across disease conditions (Healthy, MASLD and MASH) (**Figure R3A, new Supplementary Figure 1B**). ANXA2 was consistently expressed in human HSCs across all conditions. Average expression levels were modest but showed a slight increase with disease severity. In contrast, S100A10 was detected in a smaller subset of HSCs. Although the expression of S100A10 in HSC is low, it was consistently detectable in both health and disease, supporting its presence in human HSCs in vivo.

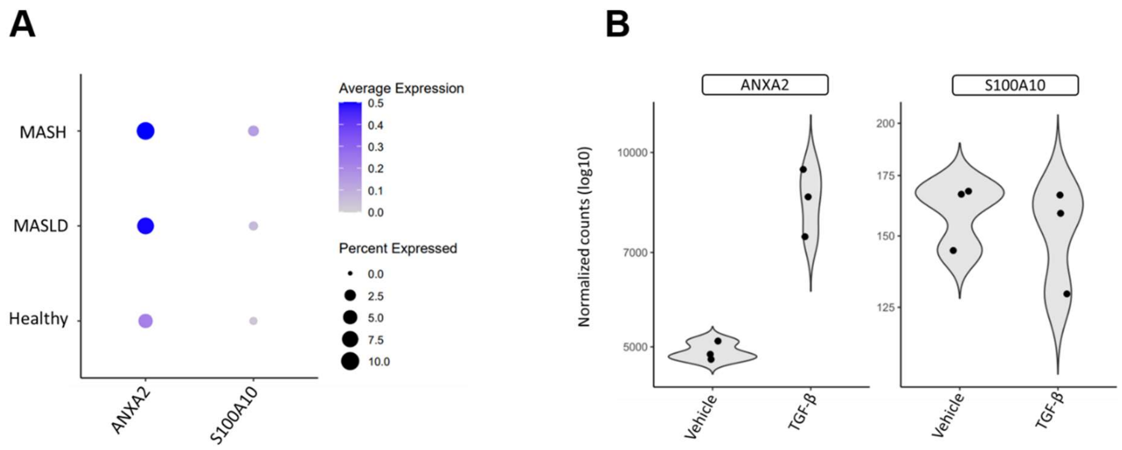

Figure R3: **(A)** Expression of ANXA2 and S100A10 in human hepatic stellate cells in vivo. Dot plots showing expression of ANXA2 and S100A10 in human HSCs across disease conditions (NORMAL, NAFL, NASH), derived from single-nucleus RNA-sequencing data (Data ref: GEO GSE244832, 2025 [5]). Each dot represents the aggregated expression within the HSC population for a given condition. Dot size indicates the percentage of HSCs expressing the gene, while dot colour reflects average normalized expression level. **(B)** Expression of ANXA2 and S100A10 in primary human hepatic stellate cells with or without TGF-β1 stimulation. Violin plots show normalized RNA-seq counts for ANXA2 and S100A10 in primary human HSCs reflecting the relative abundance of ANXA2 and S100A10 transcripts. Human HSCs cultured under solvent (Vehicle) or treated with TGF-β1 (10ng/ml) for 24 hours (Data ref: GEO GSE253493, 2024 [6]). Individual dots represent biological replicates (n = 3 per condition). Expression values are shown on a log10 scale.

To validate these findings, we further analyzed bulk RNA-seq data from primary human HSCs treated with TGF-β1 (10ng/ml) (Data ref: GEO GSE253493, 2024 [6]) (**Figure R3B, new Supplementary Figure 1A**). ANXA2 is robustly expressed in primary human HSCs and its expression largely increased after TGF-β1 stimulation, whereas S100A10 was detected at lower levels and remained consistently expressed following TGF-β1 stimulation. These results are concordant with the results obtained from the snRNA-seq analysis.

Collectively, these independent *in vivo* and *ex vivo* analyses demonstrate that ANXA2 and S100A10 are expressed in human HSCs, at the mRNA level. While ANXA2 expression tends to increase with fibrosis-associated conditions, S100A10 expression is limited to a smaller HSC subset and remains stable across disease states. These findings support the expression of both ANXA2 and S100A10 in human HSCs, extending evidence beyond previous described reports in hepatocytes. Importantly, the mRNA levels do not give solid indication at the protein levels or regarding the formation of the heterotetrametric complex that might be fostered in disease conditions. As a limiting factor, these datasets provide indications in an heterogenous population of activated and non-activated HSC.

Finally, in our study, our data are transposable from HLOs to LX-2 human stellate cell line, demonstrating the key contribution of A2t in HSCs.

This information has been implemented to the Revised version of the Manuscript line 118-135:

"Activated hepatic stellate cells are the principal source of extracellular matrix deposit in the fibrotic liver. S100A10 and ANXA2 expressions in HSCs and their regulation during activation or disease progression remains insufficiently characterized. Here, we re-analyzed a publicly available RNA-seq dataset (Data ref: GEO GSE253493, 2024 [6]) of primary human HSCs cultured under basal conditions or stimulated with TGF-β1. ANXA2 transcripts were abundantly expressed in human primary HSCs and increased upon TGF-β1 activation (Supplementary Figure 1A). In contrast, S100A10 transcripts were detected at low levels and not significantly affected by TGF-β1 stimulation. To assess their expression in vivo, we additionally analyzed a human single-nucleus RNA-sequencing (snRNA-seq) dataset comprising liver samples from healthy individuals and patients suffering from MASLD or MASH (Data ref: GEO GSE244832, 2025 [5]). Here again, ANXA2 was consistently detected in HSCs among all subjects, with modest expression that tended to increase with disease severity (Supplementary Figure 1B). S100A10 was present in a smaller fraction of HSCs, although at lower levels, detectable in both health and disease states, substantiating its expression in human HSCs. Given that both ANXA2 and S100A10 are expressed, we hypothesized that the ANXA2-S100A10 tetramer (A2t) may play a functional role in HSC activation and fibrogenic responses."

2.Although A2Ti-1 has shown clear antifibrotic effect in MASH HLOs, it is unclear if the effect is on target. I.e. Is A2Ti-1's effect attributable to its ability to block S100A10-ANXA2 heteromerization? This can be addressed by testing the effect of this drug on MASH HLOs in the presence or absence of S100A10 and/or ANXA2 expression, as

has been performed in LX2 cells. Alternatively, the authors can directly show their heteromerization through co-immunoprecipitation experiments.

We thank the Reviewer for raising this crucial point regarding the on-target specificity of A2ti-1 in HLOs. We agree that genetic perturbation of S100A10 and/or ANXA2 in A2ti-1-treated HLOs would provide a strong argument regarding target dependency. However, the limited availability of HLOs material and the technical challenges associated with genetic manipulation in this system represent a limitation that cannot be crossed over at this stage.

Nevertheless, convergent evidence support the view that the antifibrotic effects of A2ti-1 are caused by the disruption of the Annexin A2-S100A10 (A2t) complex. A2ti-1 belongs to a class of substituted 1,2,4-triazole inhibitors originally identified through structure-based pharmacophore modelling combined with biochemical screening to disrupt the ANXA2-S100A10 interaction [7]. Because Annexin A2 is known to associate specifically with S100 family members (including S100A6, S100A4, and S100A11), selectivity within this protein family is an important consideration. Notably, these S100 paralogs, although structurally related, display distinct ANXA2-binding interfaces, which may permit selective disruption of the S100A10 complex [8]. Consistent with on-target disruption of the A2t complex, A2ti-1 was shown in a functional infection model to potently inhibit human papillomavirus type 16 (HPV16), which exploits the A2t complex for cellular entry, without detectable cellular toxicity at concentrations up to 100µM [9].

In addition, in our study, knock-down of S100A10 in LX-2 cells phenocopies key antifibrotic effects observed with A2ti-1 treatment (reduction of α-SMA as shown in Figure 1C and 1D), further supporting target achievement.

As a proof of concept, we co-treated LX-2 cells with A2ti-1 and siRNA directed against S100A10 and did not observe significant additional effects on α-SMA protein levels (**Figure R4A, new Supplementary Figure 1E**).

The following sentence has been added to the Revised version of the Manuscript lines 156-159:

"Of note, the on-target effect of A2ti-1 was further assessed by co-treating LX-2 cells with siRNAs and A2ti-1 together, without significant cumulative effects on α-SMA protein levels (Supplementary Figure 1E)."

In addition, although preliminary, we performed co-immunoprecipitation experiment in LX-2 cells transfected with S100A10-FLAG expressing-vectors or empty vectors. We first validated the interaction of ANXA2 with S100A10 (**Figure R4B and R4C, for Reviewer only**), ANXA2

being more enriched in the S100A10-FLAG transfected cells than in the mock transfected cells after immunoprecipitation of the FLAG sequence. Noteworthy, in the IP-FLAG fraction, the levels of ANXA2 were higher than in IgG2a control, even though detectable. Finally, the present assay does not lead to clear-cut conclusion on the effects of A2ti-1. Based on this data set, 1h treatment with A2ti-1 is not sufficient to disrupt A2t complexes (**Figure R4C**). However, 48h treatment, as performed in the main study, seems to reduce ANXA2 levels in the IP FLAG fraction.

Overall, this preliminary qualitative assessment in conjunction with the siRNA strategy strengthen the on-target effect of A2ti-1 in our context.

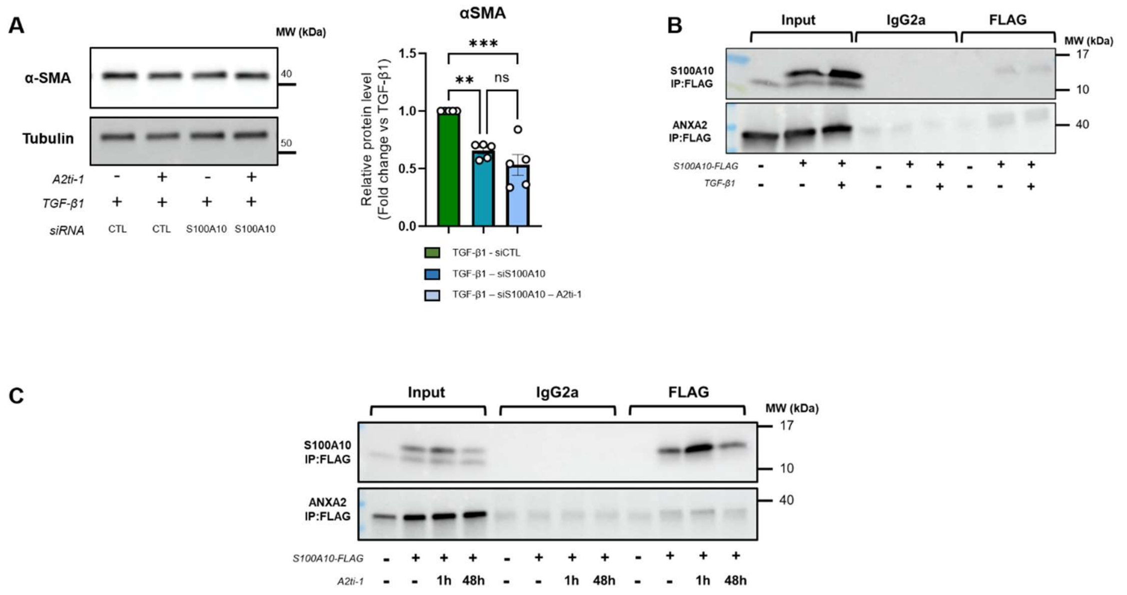

Figure R4: (A) Western blot quantification of α-SMA protein levels in LX-2 cells treated with TGF-β1 (5ng/ml) for 2 days in the absence or presence of A2ti-1 (50µM) and transfected with siRNA control of direct against S100A10. Tubulin served as a loading control (n=5) **(B)** Western blot analysis of S100A10 and ANXA2 following FLAG immunoprecipitation of the cell lysates from LX-2 cells transfected or not with S100A10-FLAG and treated or not with 5 ng/ml TGF-β1 **(C)** Western blot analysis of S100A10 and ANXA2 following FLAG immunoprecipitation of the cell lysates from LX-2 cells transfected or not with S100A10-FLAG and treated or not with 50 µM A2ti-1 for 1h or 48h.

3.What is the toxicity of the A2Ti-1 compound? A proper dose-response study coupled with a cell viability measurement needs to be performed for cell lines and organoids for this compound to rule out cytotoxicity as an alternative explanation for the changes detected.

We agree with the Reviewer that assessing cytotoxicity is important when interpreting the antifibrotic effects of A2ti-1, even though targeted cytotoxicity against activated hepatic stellate cells might be something beneficial in order to treat hepatic fibrosis. In HeLa cells, A2ti was previously shown to exert no substantial cellular toxicity or reduction in cell growth over 3 days of exposure up to 100 µM [9].

Regarding HLOs used in our study, we directly assessed apoptotic responses by measuring cleaved caspase-3 protein level following chronic A2ti-1 treatment. Levels of cleaved caspase-3 were unaffected by 50 µM A2ti-1 (**Figure R5A, new Supplementary Figure 3C**), and the expression of key pro-apoptotic (BAX, BAK1) and anti-apoptotic (BCL2, BCL2L1) genes remained unchanged (**Figure R5B, new Supplementary Figure 3D**). This indicates that A2ti-1 did not induce intrinsic apoptosis in HLOs at 50 µM concentration. Of importance, we performed the analysis on HLOs lysate. Thus, we do not exclude a possible cell-specific effect in activated stellate cells.

The Revised version of the manuscript has been edited with the following sentence line 269: *“without noticeable pro-apoptotic effects (Supplementary Figure 3B and 3C).”*

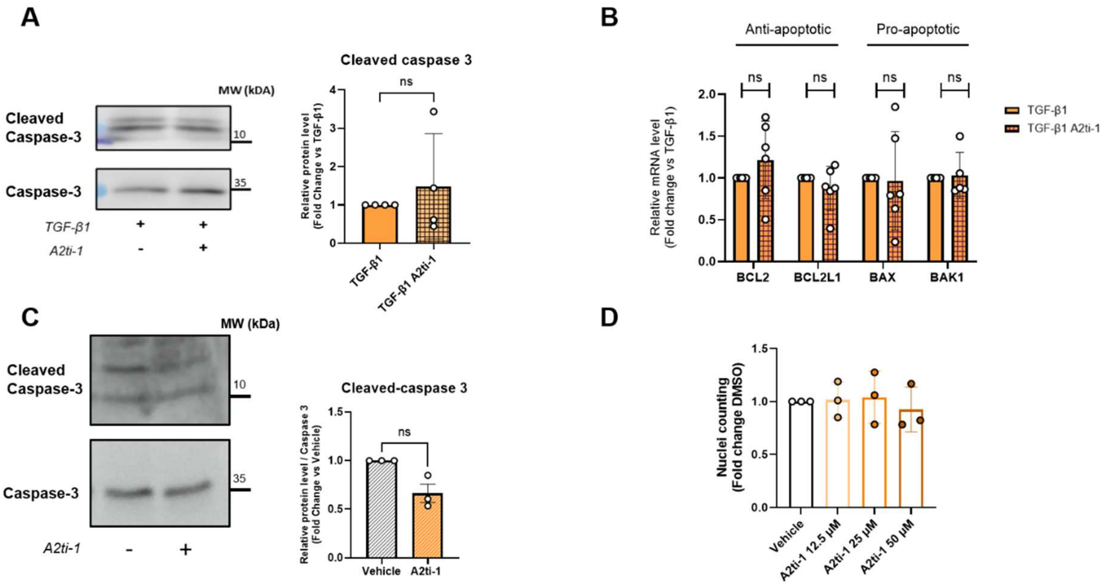

Figure R5: (A) Representative Western blot images (left) and quantification (right) of Cleaved Caspase-3 protein levels in HLOs treated with TGF-β1 (20 ng/ml) with or without A2ti-1 (50 µM, n=4) for 5 days. **(B)** Relative mRNA level of pro- and anti-apoptotic gene expression in HLOs treated with TGF-β1 (20ng/ml) with or without A2ti-1 50µM, n=5) for 5 days. Anti-apoptotic genes (BCL2, BCL2L1) and pro-apoptotic genes (BAX, BAK1) are shown as fold change relative to TGF-β1. Cyclophilin A and TBP were used as housekeeping genes. **(C)** Representative Western blot images (left) and quantification (right) of Cleaved Caspase-3 protein levels in LX-2 cells treated or not with A2ti-1 (50 µM, n=3) for 2 days. **(D)** Automatic nuclei counting of LX-2 cells (n=3) treated with increasing concentrations of A2ti-1 (12.5, 25 or 50 µM) for 2 days, measured on ImageXpress. Data are expressed as fold change versus Vehicle condition. Triplicates have been performed for each biological replicate.

In order to disentangle cell-specific potential cytotoxic effect of A2ti-1 on hepatic stellate cells we performed the same experiment in the LX-2 cell line. A2ti-1 treatment did not affect cleaved caspase 3 levels (**Figure R5C, for Reviewer only**). In addition, dose-response study using increasing concentrations of A2ti-1 did not show major toxicity (**Figure R5D, new Supplementary Figure 1C**). The following sentence has been added to the Result section of the Manuscript line 138-139:

"*Dose-response study was performed for A2ti-1 and showed no significant cytotoxicity (Supplementary Figure 1C).*"

Overall these new data show that A2ti-1 is not cytotoxic for HLOs or LX-2 cells at the tested concentration.

4.The scRNAseq data from 21d HLO is significantly underexplored. As the establishment of MASH-like HLOs (Figures 2 and 3) is a major point of the manuscript, the authors should compare their results to prior MASH-like HLOs, especially the ones that informed the advancements reported in this paper. As achieving mature cells is a major challenge and goal of developing HLOs, how do the maturity of different cell types detected by scRNAseq compare to previously reported for HLOs (doi: 10.15252/embj.2023113898), and adult human liver (many publicly available datasets).

We agree with the Reviewer that scRNA-seq based assessment of cell maturity and comparison to prior MASH-like HLO models is an important consideration. We would like first to clarify the scope of the scRNA-seq data generated in the current study and how it relates to previously published work.

In our study, scRNA-seq analysis was performed exclusively on day 21 (D21) HLOs, prior to MASLD/MASH induction. These HLOs were generated using the same base differentiation protocol described by Ouchi et al. [10], with a minor modification in culture format (use of air-liquid interface plates) in order to foster gas exchanges. This differentiation strategy is consistent with that used in the referenced study (Data ref: GEO GSE207889, 2023), (doi: 10.15252/embj.2023113898) [11]. While we subsequently modelled MASLD/MASH in these HLOs, scRNA-seq data were unfortunately not generated at later disease-modelling time points, and therefore a direct single-cell level comparison of MASLD/MASH-induced states between studies is not feasible.

Given this limitation, we focused our scRNA-seq analysis on the assessment of cellular composition and maturation status of D21 HLOs and benchmarking these data versus the D21 hepatocyte-like populations reported in the reference study. We assessed the similarity of hepatocyte identity by comparing relative expression patterns of established mature and immature hepatocyte marker genes using dot plots of average expression across hepatocyte-like cells.

As shown in **Figure R6**, our data and the previously published D21 HLO scRNA-seq reference dataset exhibit very similar expression patterns of canonical immature/hepatoblast and mature hepatocyte markers in hepatocyte-like population (upper panel). Both datasets display concurrent expression of mature hepatocyte markers (e.g., ALB, HNF4A, APOB) together with

fetal/immature markers (e.g., AFP, IGF2). This is consistent with a shared hepatocyte-like transcriptional state at this differentiation stage. Minor quantitative differences in the fraction of expressing cells and average expression levels are observed, with slightly higher ALB and HNF4A expression in our dataset and more elevated AFP and GPC3 expression levels. These data are now incorporated in the revised version of the manuscript (**Supplementary Figure 2D**), line 187-195:

"Additionally, we compared hepatocyte-like cells from D21 HLOs with those from a previously published D21 HLO scRNA-seq study (Hess et al., 2023 [11]) by examining established immature/hepatoblast and mature hepatocyte marker expression (Supplementary Figure 2D). Both datasets showed concurrent expression of mature markers (e.g., ALB, HNF4A, APOB) and fetal/immature markers (e.g., AFP, IGF2), indicating a comparable intermediate hepatocyte-like maturation state at this stage. While minor quantitative differences in expression levels and in the fraction of expressing cells were observed, the overall transcriptional profiles were highly similar between studies."

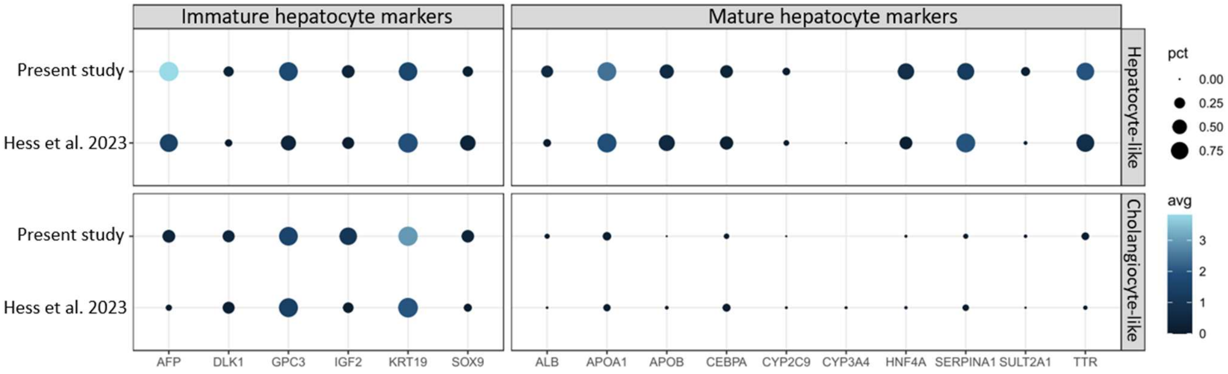

Figure R6: Single-cell transcriptomic comparison of hepatocyte-like cells at day 21. Dot plot comparing the expression of representative immature/hepatoblast (AFP, DLK1, GPC3, IGF2,KRT19, SOX9) and mature hepatocyte markers (ALB, APOA1, APOB, CEBPA, CYP2C9, CYP3A4, HNF4A, SERPINA1, SULT2A1, TTR) in hepatocyte-like clusters from D21 HLOs generated in present study and from a previously published D21 HLO scRNA-seq dataset (Data ref: GEO GSE207889, 2023) [16]. Cholangiocyte-like cells are included to demonstrate that hepatocyte markers are not ubiquitously expressed in the dataset. Dot size indicates the fraction of cells with detectable expression (expression > 0), and colour indicates the mean log-normalized expression across hepatocyte-like cells. Datasets were analysed separately. One limitation of this comparison is that the datasets may not be normalized identically as we used the provided normalized values from Hess et al. 2023.

Regarding comparison with adult human liver, Reviewer's point is well taken. However we would like to emphasize that HLOs generated using current differentiation strategies, including those in the study performed by Hess et al. [11] and Ouchi et al. [10], are well known to retain a fetal or immature hepatocyte-like transcriptional profile. The developmental state of pluripotent stem cell-derived liver organoids, including those generated using the Ouchi et al. [10] methodology on which our system is based, has been systematically evaluated in a transcriptomic meta-analysis study [12]. In that study, CellNet-based gene regulatory network (GRN) analyses compared multiple Pluripotent Stem Cell-derived hepatocyte and liver organoid protocols, including Ouchi-derived HLOs, versus primary human hepatocytes (PHH) and whole liver tissue. While Ouchi-derived HLOs exhibited robust liver GRN scores relative

to other Pluripotent Stem Cell-based organoid systems, their GRN scores remained consistently lower than those of cryopreserved and freshly isolated adult PHH, as well as whole liver tissue [12]. These findings indicate that Ouchi-based HLOs recapitulate key liver regulatory programs but do not fully reach adult hepatocyte identity, consistent with a developmentally immature (fetal-like) hepatocyte state.

Overall, the goal of the present study was not to claim adult-like maturation, but rather to establish a robust and reproducible human HLO platform suitable for MASLD/MASH modelling and mechanistic interrogation. Notably, despite their fetal-like transcriptional profile, hepatocyte-like cells in our system exhibit adult liver functions, including glycogen storage and urea secretion (**Figure 2F-G of the Manuscript**). In order to be fully transparent and trigger the attention of the reader on this point, we added the following sentence in the Revised Manuscript (lines 195-198):

"Although HLOs express mature hepatocyte-specific genes, residual expression of the progenitor-associated marker KRT19 indicates that hepatocyte-like cells retain features of developmental immaturity at day 21."

5.The authors report expression of zone 3 marker GS in their HLOs. This begs the question whether zone 1 markers are also expressed in these HLOs or is it zone 3 specific?

Whether our HLOs express zone 1 markers was indeed an unexplored point beyond the scope of our study. Nevertheless, to address whether hepatocyte-like cells display pericentral (zone 3) or periportal (zone 1) gene signatures, we performed a systematic single-cell transcriptomic assessment of canonical periportal and pericentral gene programs.

We compiled extensive periportal (zone 1) and pericentral (zone 3) gene signatures derived from published human liver single-cell datasets [13]. To quantify zonation bias, we calculated module scores for periportal and pericentral gene sets for each cell, and derived a continuous porto-central coordinate defined as the standardized difference between pericentral and periportal scores. This continuous measure was subsequently used to classify cells into periportal-like, pericentral-like, or mixed states using a data-driven threshold. Under these conservative criteria, the vast majority of hepatocyte-like cells fell within the intermediate range ("mixed"), while only small subsets exhibited clear periportal-like or pericentral-like profiles (**Figure R7, new Supplementary Figure 2F)**. This indicates that, even if most hepatocyte-like cells express pericentral Glutamine Synthase, they lack a strong transcriptional program toward either periportal or pericentral programs, consistent with incomplete zonation rather than mature spatial segregation. Despite the absence of a fully defined periportal

transcriptional program, the HLOs exhibited robust urea secretion (Figure 2G of the Manuscript), consistent with functional activation of the urea cycle.

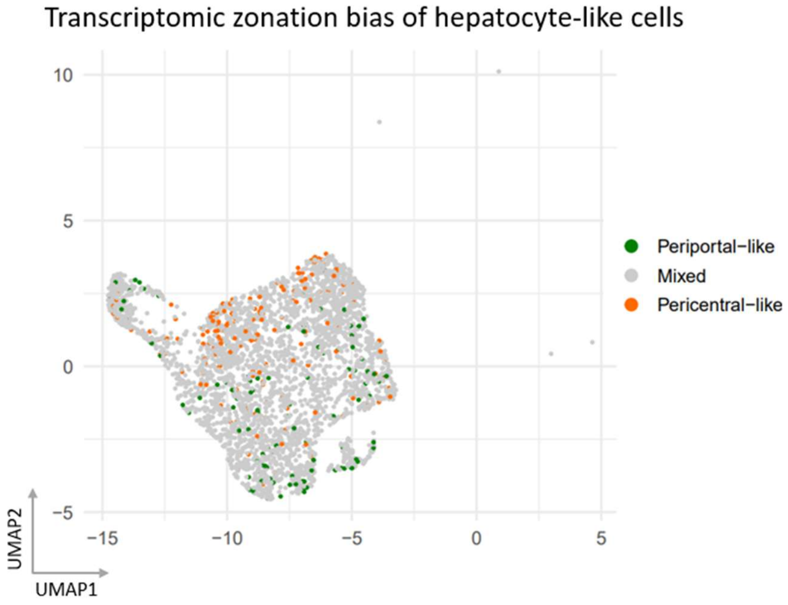

Figure R7: Transcriptomic zonation bias of hepatocyte-like cells. UMAP visualization of hepatocyte-like cells coloured according to transcriptional zonation bias. A continuous porto-central coordinate was computed from single-cell RNA-seq data using module scores for periportal (zone 1) and pericentral (zone 3) gene signatures derived from human liver datasets [64]. The zonation axis represents the standardized difference between pericentral and periportal scores. Cells were classified using a stringent threshold (±1.75 SD) into periportal-like (green), pericentral-like (orange), or mixed (grey) populations.

The Result section of the manuscript has been updated as follow (line 202-206):

"Despite detectable GS expression, hepatocyte-like cells predominantly exhibited a mixed transcriptional pattern (Supplementary Figure 2F). This indicates that most hepatocyte-like cells lack a strong transcriptional program toward either periportal or pericentral programs, consistent with incomplete zonation rather than mature spatial segregation."

The Material and Methods section of the manuscript has been updated as follow (line 714-726):

"To assess hepatocyte spatial zonation, a continuous porto-central zonation axis was computed based on established periportal and pericentral gene expression signatures from [64]. For each hepatocyte, module scores for periportal and pericentral programs were calculated using Seurat's AddModuleScore function, which computes the average expression of a gene set relative to control genes matched for expression level. A continuous zonation coordinate was then defined as the standardized difference between pericentral and periportal module scores:

Zonation axis = scale(Pericentral score − Periportal score)

To categorize cells into periportal-like, pericentral-like, or intermediate states, a strict data-driven threshold was applied to the continuous zonation axis. The optimal threshold (±1.75 standard deviations) was selected empirically to capture only cells exhibiting strong

transcriptional bias toward either program while minimizing misclassification of intermediate cells."

Minor comment:

1.Fig1B, what is the effect of A2Ti-1 on LX2 cells alone? LX2 cells are highly activated already so A2Ti-1 may already have an antifibrotic effect. Or is there only an antifibrotic effect in the presence of TGFb1 stimulation?

As mentioned by the Reviewer, LX-2 cells are, to some extent, already activated. This raises the question of A2ti-1 effects in the absence of TGF-β1 stimulation. Noteworthy, LX-2 cells are cultivated in 2% FBS which contains TGF-β1. In addition, LX-2 cells most probably secrete TGF-β1 themselves. Both contributions might explain the basal activation level of these cells. In that regard, in the absence of TGF-β1 supplementation, we observe a trend for α-SMA protein level reduction in A2ti-1-treated LX-2 cells (**Figure R8, new Supplementary Figure 1D**). Whether the effect is TGF-β1 independent or contributed by endogenous levels of TGF-β1 in the medium cannot be assessed. The following sentence has been added to the Result section of the Revised Manuscript lines 144-146:

"Background induction of α-SMA in LX-2 cells, without addition of exogenous TGF-β1, also tended to be reduced by 50 µM A2ti-1 (Supplementary Figure 1D)"

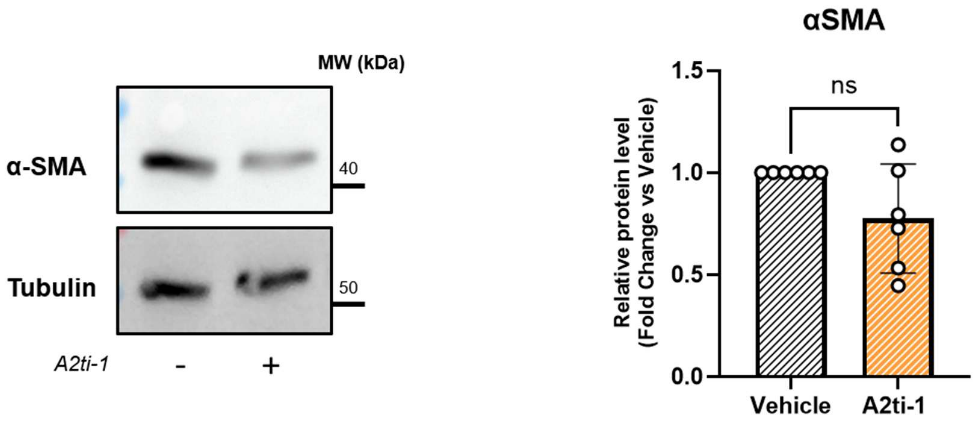

Figure R8: Representative Western blot images (left) and quantification (right) of α-SMA protein levels in LX-2 cells treated or not with A2ti-1 (50 µM, n=6) for 2 days.

Referee #3:

Major Comments;

1.Figure 1D reveals that siRNA-mediated knockdown of S100A10 leads to increased mRNA levels of VIM and TIMP1 under TGF-β1 stimulation compared to controls, while

COL1A1 and COL3A1 expression remains unchanged-findings that contrast with those observed following A2ti-1 treatment (Fig. 1B). This inconsistency implies either the involvement of A2T-independent regulatory functions of S100A10 or the induction of compensatory adaptive responses due to partial gene silencing. To minimize off-target effects and better reflect functional loss, it is advisable to employ CRISPR/Cas9-mediated S100A10 knockout.

It is correct that S100A10 knockdown and pharmacological inhibition of the A2t complex do not fully phenocopy each other at the transcriptional level, particularly for COL1A1 and COL3A1. However, several lines of evidence listed below support that these differences do not preclude the take home message.

Both approaches consistently suppressed hepatic stellate cell activation, as evidenced by significant ACTA2 downregulation at the mRNA level and robust α-SMA protein reduction (Fig. 1A, 1C of the Manuscript). This indicates that interfering with S100A10 alone or A2t complex reproducibly suppress hepatic stellate cell activation.

Second, siRNA-mediated knockdown resulted in ~80% reduction of S100A10 protein level, leaving a residual pool that may still support partial A2t formation and background stimulation. Along the same line, A2ti-1 may not fully disrupt the whole pool of A2t complex. Thus, incomplete genetic depletion of S100A10 may leave residual A2t activity to sustain collagen transcription, while pharmacological blockade more effectively suppresses this pathway, even though this does not represent the major hypothesis.

Another explanation for this experimental paradigm could be the differences in the kinetics. A2ti-1 is expected to rapidly interfere with A2t formation, whereas siRNA treatment requires prolonged exposure (3 days to reach 80% knockdown) to achieve maximal knockdown. Given that collagen transcription represents a downstream fibrogenic program following hepatic stellate cell activation, it is likely that differences in time line contribute to the observed differences in COL1A1 and COL3A1 regulation.

The increased VIM and TIMP1 expression observed upon S100A10 knockdown but not upon A2ti-1 treatment may reflect adaptive or compensatory transcriptional responses to partial loss of S100A10, consistent with the Reviewer's suggestion of non-A2t-dependent functions. In addition, siRNA-mediated depletion requires lipid-based transfection, which is known to elicit transient cellular stress responses. Such stress-associated effects, together with sustained partial loss of S100A10, may contribute to adaptive transcriptional responses, including the regulation of VIM and TIMP1.

Lastly, we fully acknowledge that an additional CRISPR/Cas9-mediated approach to achieve S100A10 knockout would further strengthen the delineation of the role of A2t. Establishing a stable knockout model would be a valuable direction for future studies.

In order to trigger the attention of the readers to these limitations, the following sentence has been added to the Revised version of the Manuscript (lines 151-156):

"qPCR analysis confirmed reduced ACTA2 and MMP2 mRNA levels upon S100A10 knockdown, while COL1A1 and COL3A1 were unchanged (Figure 1D). These results reveal slight differences between S100A10 gene silencing on the one hand and A2t pharmacological disruption on the other, none of them resulting in complete ablation of the A2t complex resulting in residual background activation."

2.The authors propose that A2ti-1 exerts anti-fibrotic effects by inhibiting STAT3 phosphorylation. However, the mechanistic link between the S100A10-ANXA2 heterotetramer (A2t) and the STAT3 pathway remains inadequately explored. Specifically, it is unclear whether A2t directly interacts with STAT3 or JAK, or alternatively modulates downstream signaling via regulation of membrane receptor dynamics-such as PDGFRβrecruitment or endocytosis. The absence of direct experimental validation for such interactions undermines the conclusion that the A2t-STAT3-hepatic stellate cell axis constitutes a central regulatory mechanism in liver fibrosis. Further mechanistic investigation is therefore recommended to substantiate this proposed signaling cascade.

Although our study clearly shows the implication of STAT3, we agree that further mechanistic explorations are needed to fully understand the exact position of A2t in the signaling pathway involving STAT3, which goes beyond the scope of the present study.

Indeed, A2t complex has been previously linked with endosomal dynamics through a calcium dependent effect [14]. In the context of our study, several receptors including PDGFR or others such as IL-6 receptors might be under the control of ANXA2/S100A10, although this view remains hypothetical at this stage.

To gain more insights into the link between STAT3 and S100A10/ANXA2, we took advantage of a pilot co-immunoprecipitation experiment performed to assess the interaction of S100A10 with ANXA2, and checked the presence of STAT3 (**Figure R9, for Reviewer only**). In the fraction immunoprecipitated with the FLAG antibody, STAT3 was not detected. This preliminary result neither demonstrate nor rule out direct (and possibly transient) STAT3 interaction with A2t, which should be investigated in future studies.

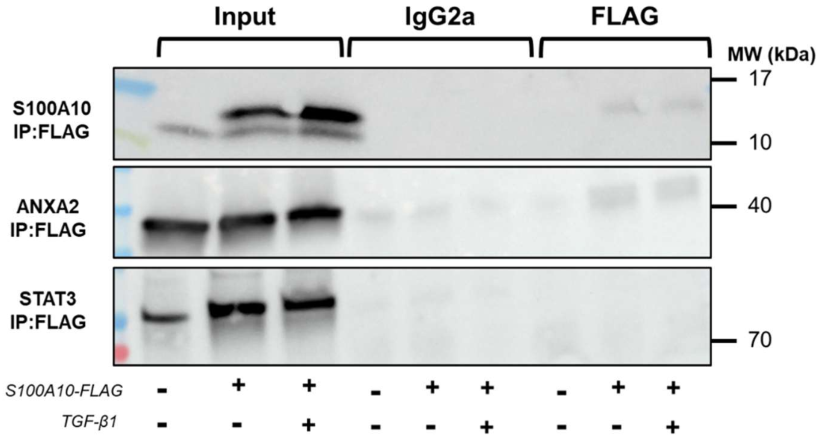

Figure 9: Western blot analysis of S100A10, ANXA2 *and STAT3* following FLAG immunoprecipitation of the cell lysates from LX-2 cells transfected or not with S100A10-FLAG and treated or not with 5 ng/ml TGF-β1.

3.A hallmark feature of metabolic dysfunction-associated steatohepatitis (MASH) is immune cell-driven inflammation, particularly mediated by macrophages. Notably, the hepatic liver organoids (HLOs) employed in this study appear to lack immune components, a point corroborated by single-cell RNA sequencing data (Fig. 2B). Consequently, the cellular source of IL-6 within the culture system requires clarification: is it derived from stromal cells or injured hepatocytes? Although TGFβand OA/PA robustly induce IL-6 production (Fig. 3J), no reduction in IL-6 levels is observed upon A2ti-1 treatment (Fig. 4F), suggesting an unresolved regulatory mechanism. This discrepancy necessitates further investigation to elucidate the pathways involved.

As pointed out by the Reviewer, HLOs used in this study lack immune cell population. This represents an intrinsic limitation of current HLOs systems, hepatocytes deriving from endoderm, whereas hepatic stellate cells originate from mesodermal mesenchyme and Kupffer cells from mesoderm-derived hematopoietic progenitors [15]. Recapitulating all these lineages within a single differentiation protocol remains an unmet goal, and several groups are actively testing strategies to incorporate Kupffer cells by mimicking fetal liver hematopoiesis or by mixing macrophages to HLOs at latter stages.

Given the absence of immune cells, IL-6 detected in our system cannot originate from macrophages or other immune cells and consequently is produced by non-immune compartments within the organoids. Both hepatic stellate cells and damaged hepatocytes are known to produce IL-6 in response to stress signals, such as TGF-β1 or lipotoxic route, supporting a parenchymal or stromal origin in this context (reviewed in [16]). In our study, while

TGF-β1 and OA/PA robustly induced IL-6 secretion (Fig. 3J of the Manuscript), A2ti-1 treatment did not significantly reduce IL-6 levels (Fig. 4F of the Manuscript).

This suggests that IL-6 secretion in this immune-free system is independent of A2t activity. In contrast, A2ti-1 consistently suppressed hepatic stellate cell activation and fibrotic markers, indicating that its primary action is anti-fibrotic rather than broadly anti-inflammatory. This indicates that hepatic stellate cells may not be the source of IL-6 in our system. Therefore, injured-hepatocytes in response to TGF-β1 or OA/PA could be the source. We performed immunostainings in HLOs for HNF4 (hepatocytes) and cleaved caspase 3 (apoptosis) and observed no differences in the proportion of apoptotic hepatocytes in control versus TGF-β1 treated HLOs (**Figure R10, for Reviewer only**), the few apoptotic cells being non-hepatocytes (white arrows). Therefore, IL-6 found in the medium would not be a passive release secondary to apoptosis.

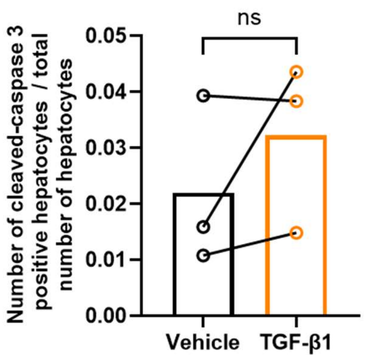

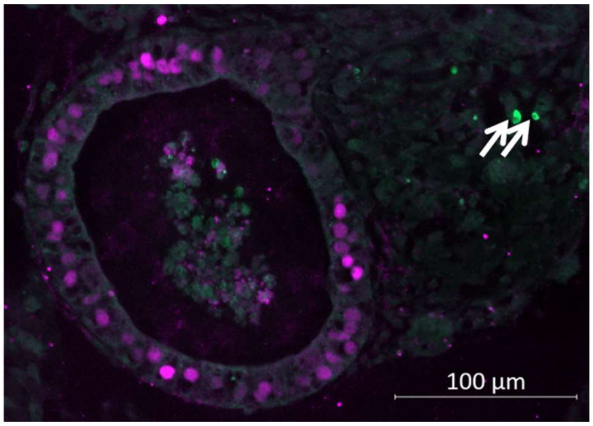

Figure R10: Representative picture (right) and quantification (right) of immunostaining for HNF4 (pink, hepatocytes) and cleaved-caspase 3 (green) in day 26 HLOs treated or not with 20 ng/ml TGF-β1 for 5 days.

Interestingly, in our study we observed that A2ti-1 hampered STAT3 phosphorylation without affecting the canonical TGF-β1 SMAD dependent pathway. Our hypothesis is that IL-6 secretion would be more dependent on SMAD pathway rather than STAT3, arguing for an A2t independent effect. This requires further investigations to be undertaken in future studies.

Of importance, once secreted IL-6 is known to initiate signaling pathways relying on JAK/STAT [17]. Therefore, even if the amount of secreted IL-6 is unchanged in response to A2ti-1 treatment, downstream deleterious signaling pathways would be reduced.

Together, these observations support a model in which A2ti-1 selectively targets fibrogenic pathways in HSCs, while IL-6 production in HLOs reflects ongoing non- immune stress.

The following text has been implemented to the Revised version of the Manuscript lines 437-446:

"Of note, the HLOs produced in this study lack resident immune cells. IL-6 production in this system likely reflects stress responses by non-immune cells. In this context, A2ti-1 selectively suppresses fibrogenic HSC activation without broadly attenuating stress-associated cytokine production IL-6. Here, we observed that A2ti-1 hampered STAT3 phosphorylation without affecting the canonical TGF-β1 SMAD-dependent pathway. Our hypothesis is that IL-6 secretion would be more dependent on SMAD pathway rather than STAT3, arguing for an A2t independent effect. Once secreted, IL-6, initiates signaling pathways relying on JAK/STAT [55] . Therefore, even if the amount of secreted IL-6 is unchanged upon A2ti-1 treatment, downstream deleterious signaling should be reduced."

4.Single-cell RNA sequencing was performed on only one sample derived from a single HLO differentiation batch. Although qPCR confirmed similar marker expression patterns across batches, this supports consistency in differentiation trajectory but does not account for potential batch-to-batch variability in cell type proportions or subpopulation heterogeneity. Thus, extrapolating cluster analysis from a single sample as representative of the "standard" HLO composition may compromise generalizability. Moreover, the omission of an A2ti-1-treated group in scRNA-seq limits the ability to assess drug-induced phenotypic shifts in distinct cell populations. Including such data would significantly enhance mechanistic insights into A2ti-1's mode of action.

We concur with the Reviewer regarding the limitations of performing the single-cell RNA sequencing on only one batch of HLOs differentiation. However, the qPCR analysis shown on Supplemental Figure 1 provides reliable information regarding the batch-to-batch reproducibility regarding both differentiation status and cell type distribution. Indeed, significant differences in cell proportion would affect relative mRNA levels detected from whole HLOs lysate.

In addition, addressing Reviewer's comment, we documented in four different batches the proportion of hepatocytes (based on HNF4 immunostaining, **Figure R11**). We observed in these 4 batches in average of 36% of hepatocyte-like cells, as shown by the single-cell RNA-seq (Figure 2B of the Manuscript). This data strengthens the extrapolation performed in the single-cell RNA-seq (Figure 2B) and has been added to the Revised version of the Manuscript as **Supplementary Figure 2C**. The following sentence has been added line 184-186:

"HNF4α staining of D21 HLOs in four independent batches also underscores the batch to batch reproducibility regarding the hepatocyte-like population (Supplementary Figure 2C)."

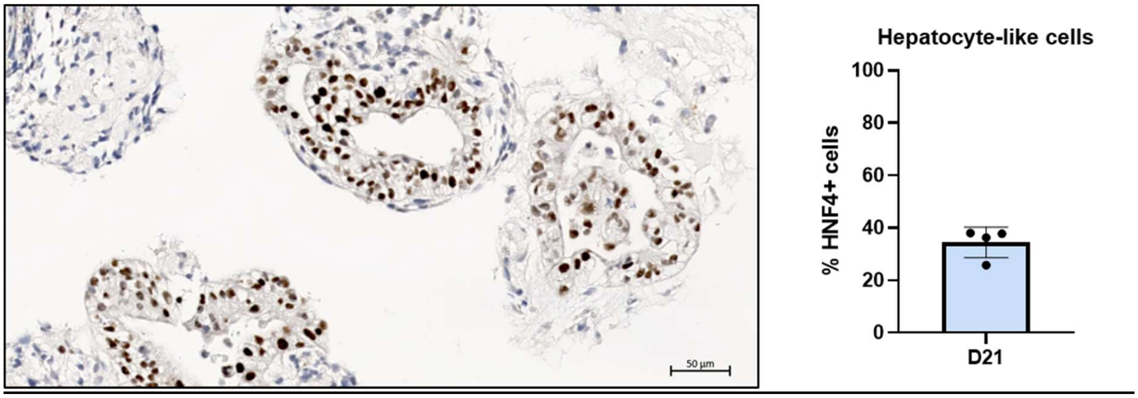

Figure R11: Representative picture (left) and quantification (right) of HNF4 immunohistochemistry performed in mature HLOs at day 21 of the differentiation process. HLOs were counterstained with hemalum. Results are expressed as percentage of HNF4 positive cells. At least 20 HLOs per condition have been stained.

Finally, we did not perform sc-RNAseq on HLOs treated with A2ti-1 because such analysis was conducted at day 21 of differentiation, i.e. at the time point where various treatments (including A2ti-1) would be initiated until final day 26. The RNAseq is a snapshot view of the differentiation state when HLOs are being exposed to fatty acids or TGF-β1 (without or with A2ti-1) and was not aimed at exploring potential phenotypic shifts. This might indeed represent an interesting aspect to explore in future studies.

5.In Figure 4, A2ti-1 treatment markedly reduces α-SMA protein levels in the OA/PA+TGF-β1 model (Fig. 4C), yet shows no significant effect on ACTA2, COL1A1, or COL3A1 mRNA levels (Supplementary Fig. 2B). In contrast, under TGF-β1-only conditions, A2ti-1 consistently downregulates both α-SMA protein and pro-fibrotic transcripts. This differential response represents a notable inconsistency that is neither acknowledged nor explained in the current text. Clarification regarding the underlying molecular mechanisms responsible for this context-dependent effect is essential.

We thank the Reviewer for highlighting the apparent discrepancy between α-SMA protein levels and pro-fibrotic transcript responses to A2ti-1 under TGF-β1 versus OA/PA + TGF-β1 conditions. We agree that this context-dependent effect warranted clarification, and we have now addressed this point both experimentally and conceptually.

To better understand the baseline transcriptional state induced by OA/PA + TGF-β1, we performed additional analyses of fibrosis-related gene expression in the absence of A2ti-1 (**Figure R12**). These new data (new **Figure 3J** of the Manuscript) demonstrate that in OA/PA + TGF-β1 conditions, unlike TGF-β1 alone, ACTA2/α-SMA is the only fibrotic marker that was significantly induced at the transcriptional level, whereas canonical extracellular matrix genes

such as COL1A1 and COL3A1 were not upregulated by the combined treatment. TIMP1 and MMP2 displayed modest upward trends. These results indicate a partial fibrotic transcriptional response rather than a fully activated program of extracellular matrix production under OA/PA + TGF-β1 treatment compared to TGF-β1 alone. Accordingly, A2ti-1 cannot produce robust effects on these transcripts poorly / not upregulated in this context.

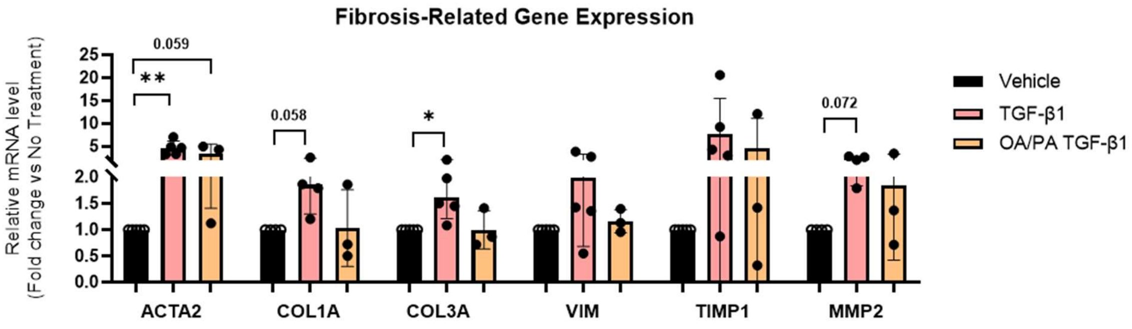

Figure R12: Relative mRNA level of fibrosis-related gene expression in HLOs treated with TGF-β1 (20ng/ml) (n=5) or with TGF-β1 (20ng/ml) + OAPA (400μM/200μM) (n=3) for 5 days. Fibrosis related genes are shown as fold change relative to vehicle (n=5). Cyclophilin A and TBP were used as housekeeping genes.

This finding contrasts with the TGF-β1 only condition, in which a coordinated transcriptional fibrotic program is activated and is consistently suppressed by A2ti-1 at both the mRNA and protein levels. Thus, the apparent discrepancy reflects distinct baseline fibrotic states, rather than an inconsistency in A2ti-1 action.

Importantly, our observations are consistent with recent single-cell transcriptomic analyses of HLOs reported by Hess et al.[11] showing that oleic acid (OA, 500μM) treatment alone reduces COL1A1 expression and collagen deposition, whereas palmitic acid (PA, 500μM) primarily induces inflammatory rather than fibrotic transcriptional programs. In this study, authors showed that OA exerts anti-fibrotic effects even in the presence of metabolic stress, aligning with evidence showing that OA-rich lipid environments attenuate fibrosis progression [18, 19].

In our experimental design, OA/PA was applied in combination at a ratio (400μM OA / 200μM PA) that likely preserves the anti-fibrotic effect of OA while limiting PA-driven fibrotic gene induction. This provides a plausible explanation for why OA/PA + TGF-β1 selectively induces α-SMA/ACTA2, reflecting contractile activation, without fully engaging a collagen-producing transcriptional program. Under these conditions, A2ti-1 can still reduce α-SMA protein levels, while exerting limited effects on collagen gene expression.

We have now explicitly discussed this context-dependent response in the revised manuscript and clarified that OA/PA modifies the fibrotic baseline, thereby shaping the apparent transcriptional sensitivity to A2ti-1.

We have updated the Revised version of the Manuscript as follow:

Result section, line 241-247: *“Interestingly, under OA/PA TGF-β1 conditions, ACTA2/α-SMA was the only fibrotic marker induced at the transcriptional level, whereas canonical extracellular matrix genes such as COL1A1 and COL3A1 were not upregulated (Figure 3J). TIMP1 and MMP2 displayed modest upward trends without reaching significance. This difference in the responses is substantiated by the weaker Masson’s trichrome coloration in the OA/PA TGF-β1 condition as compared to the TGF-β1 condition.”*

Result section, line 275-277: *“Conversely, in HLOs steatohepatitis modelling, A2ti-1 treatment did not significantly affect expression of most of the profibrotic genes, except for an increase in TIMP1 expression (Supplementary Figure 3D)”*

Discussion section, line 377-395: *“Noteworthy, we observed a difference in the A2ti-1 mediated repression of fibrosis related genes under either TGF-β1 or OA/PA TGF-β1 treatments, the latter being less responsive to A2ti-1. In the absence of A2ti-1, baseline transcriptional state of fibrosis-related genes showed a partial fibrotic transcriptional response under combined OA/PA TGF-β1 treatment compared to TGF-β1 alone that induced a fully activated program of extracellular matrix production. Thus, the apparent discrepancy reflects distinct baseline fibrotic states. These observations are consistent with recent single-cell transcriptomic analyses of HLOs reported by Hess et al. [22] which showed that OA (500µM) treatment alone reduces COL1A1 expression and collagen deposition, whereas PA (500µM) primarily induces inflammatory rather than fibrotic transcriptional programs. Hess et al. showed that OA exerts anti-fibrotic effects even in the presence of metabolic stress, aligning with evidence showing that OA-rich lipid environments attenuate fibrosis progression [44, 45]. In our experimental design, the OA/PA mix was applied at a ratio (400µM OA / 200µM PA) that likely preserved the anti-fibrotic influence of unsaturated OA while limiting PA-driven fibrotic gene induction. This might explain why OA/PA TGF-β1 selectively induces α-SMA/ACTA2, reflecting cytoskeletal activation, without fully engaging a collagen-producing transcriptional program. Under these conditions, A2ti-1 can still reduce α-SMA protein levels, while exerting limited effects on collagen gene expression.”*

6.Additionally, the nomenclature for fatty acid mixtures varies throughout the manuscript-including "oleic and palmitic acids (OA/PA)", "OAPA"-which, while interpretable, lacks terminological consistency. Standardized usage should be adopted across all figures and text.

Regarding nomenclature, we have revised the manuscript to ensure consistent terminology throughout the text and figures. The fatty acid mixture is now uniformly referred to as oleic and palmitic acids (OA/PA) across all figures, figure legends, and the main text.

Furthermore, although statistical analysis in Fig. 3K indicates a significant increase in ANXA2 expression, the corresponding Western blot image does not clearly reflect this difference; thus, inclusion of a more representative blot is recommended.

With respect to the ANXA2 Western blot in Fig. 3K (now Fig. 3L), we checked whether a more visually representative blot could be obtained. Of note, the ~1.4-fold difference in protein level might not be readily distinguishable by visual inspection. However, we rely on unbiased quantitative densitometric analysis shown in Fig. 3L. Based on the point raised by the Reviewer, we tempered the description of this effect in the revised Result section of the manuscript (line 250).

Finally, the selection of A2ti-1 concentration lacks justification. It is advised to supplement the study with a dose-response experiment (e.g., 0, 12.5, 25, 50μM) to systematically evaluate the inhibitory efficacy of A2ti-1 on TGF-β1-induced α-SMA expression.

This point is indeed of highly relevant. We would like to highlight the fact that the concentration has been chosen based on existing literature [4, 9]. In that regard, to make it clear for the reader, this information has been added to the Material and Methods section lines 517-519:

"The concentration of A2ti-1 used in this study was selected based on published data indicating effective biological activity without reported cytotoxicity in cultured cells at concentrations up to 100μM [4, 9]."

In addition, we followed up on the suggestion raised by the Reviewer about the dose-response experiment and treated LX-2 cells with different concentrations of A2ti-1 (0, 12.5, 25 and 50 μM). We observed a significant effect of A2ti-1 only at the dose of 50 μM (**Figure R13, new Figure 1A of the Revised Manuscript**). Of interest, the lowest dose 12.5 μM showed an increase in α-SMA levels. The following sentence has been added to the Result section line 141-144:

"As witnessed by the reduction of α-SMA protein levels, 50 μM A2ti-1 significantly attenuated TGF-β1-driven hepatic stellate cell activation. Lower doses of A2ti-1 did not alter the phenotype, 12.5 μM showing an unexpected enhancement of α-SMA levels (Figure 1A)"

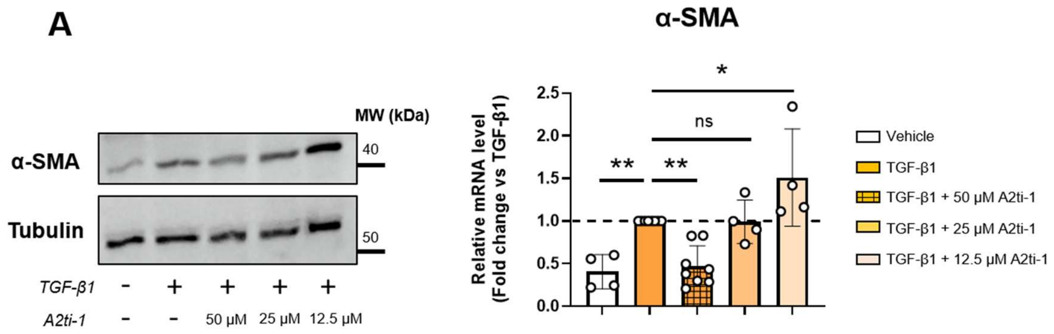

Figure R33: Representative Western blot (left) and quantification (right) of α-SMA protein levels in LX-2 cells treated with TGF-β1 (5ng/ml) for 2 days in the absence or presence of A2ti-1 (12.5 µM to 50µM). Tubulin served as a loading control (n=4 to 8).

REFERENCES

1. Yang, J., et al., *A CDAHFD-induced mouse model mimicking human NASH in the metabolism of hepatic phosphatidylcholines and acyl carnitines.* Food Funct, 2024. **15**(6): p. 2982-2995.
2. Pedersen, H.D., et al., *NASH-inducing Diets in Gottingen Minipigs.* J Clin Exp Hepatol, 2020. **10**(3): p. 211-221.
3. Chen, Z., et al., *Multi-omics analysis identifies S100a10/Anxa2 complex within proximal tubule aggravates acute kidney injury through p-Stat3/Spp1 signaling.* Transl Res, 2025. **284**: p. 57-68.
4. Weng, M., et al., *Pseudorabies Virus Regulates the Extracellular Translocation of Annexin A2 To Promote Its Proliferation.* J Virol, 2023. **97**(3): p. e0154522.
5. Kim, H.Y., et al., *Multi-modal analysis of human hepatic stellate cells identifies novel therapeutic targets for metabolic dysfunction-associated steatotic liver disease.* J Hepatol, 2025. **82**(5): p. 882-897.
6. Ma, Y., et al., *An Integrative Transcriptome Subtraction Strategy to Identify Human lncRNAs That Specifically Play a Role in Activation of Human Hepatic Stellate Cells.* Noncoding RNA, 2024. **10**(3).
7. Reddy, T.R.K., et al., *Three-Dimensional Pharmacophore Design and Biochemical Screening Identifies Substituted 1,2,4-Triazoles as Inhibitors of the Annexin A2-S100A10 Protein Interaction.* Chemmedchem, 2012. **7**(8): p. 1435-1446.
8. Liu, Y., H.K. Myrvang, and L.V. Dekker, *Annexin A2 complexes with S100 proteins: structure, function and pharmacological manipulation.* Br J Pharmacol, 2015. **172**(7): p. 1664-76.
9. Woodham, A.W., et al., *Small molecule inhibitors of the annexin A2 heterotetramer prevent human papillomavirus type 16 infection.* J Antimicrob Chemother, 2015. **70**(6): p. 1686-90.
10. Ouchi, R., et al., *Modeling Steatohepatitis in Humans with Pluripotent Stem Cell-Derived Organoids.* Cell Metab, 2019. **30**(2): p. 374-384 e6.
11. Hess, A., et al., *Single-cell transcriptomics stratifies organoid models of metabolic dysfunction-associated steatotic liver disease.* EMBO J, 2023. **42**(24): p. e113898.
12. Velazquez, J.J., et al., *Gene Regulatory Network Analysis and Engineering Directs Development and Vascularization of Multilineage Human Liver Organoids.* Cell Syst, 2021. **12**(1): p. 41-55 e11.
13. MacParland, S.A., et al., *Single cell RNA sequencing of human liver reveals distinct intrahepatic macrophage populations.* Nat Commun, 2018. **9**(1): p. 4383.
14. Drust, D.S. and C.E. Creutz, *Aggregation of chromaffin granules by calpactin at micromolar levels of calcium.* Nature, 1988. **331**(6151): p. 88-91.

15. Lotto, J., T.L. Stephan, and P.A. Hoodless, *Fetal liver development and implications for liver disease pathogenesis.* Nat Rev Gastroenterol Hepatol, 2023. **20**(9): p. 561-581.
16. Schmidt-Arras, D. and S. Rose-John, *IL-6 pathway in the liver: From physiopathology to therapy.* J Hepatol, 2016. **64**(6): p. 1403-15.
17. Kishimoto, T., *Interleukin-6: from basic science to medicine--40 years in immunology.* Annu Rev Immunol, 2005. **23**: p. 1-21.
18. Hong, Y., et al., *In vitro inhibition of hepatic stellate cell activation by the autophagy-related lipid droplet protein ATG2A.* Sci Rep, 2018. **8**(1): p. 9232.
19. Frendi, S., et al., *Protective role of oleic acid against palmitic acid-induced pancreatic fibrosis.* J Transl Med, 2025. **23**(1): p. 416.

28th Apr 2026

Dear Dr. Delangre,

Thank you for submitting your revised manuscript to EMBO Molecular Medicine. Your manuscript will be ready for acceptance pending the following final amendments:

1) Figures:
- The resolution of the submitted figures is too low for blot and microscopy images. This reduction in resolution is commonly caused by converting original 16-bit TIFF files to RGB format for publication. While this is not inherently problematic, it can raise concerns about image integrity for critical readers. To avoid any misunderstanding and to meet EMBO Press standards, we kindly ask that you resubmit the complete figure set at the captured original data resolution.
- Please rename supplementary figures to Figure EV1 etc. and update their callouts in the manuscript text.
- In supplementary figure 4 remove A for panel designation as there is only one panel and update its callouts in the manuscript text.
2) Please provide source data and completed source data checklist.
3) In the main manuscript file, please do the following:
- Please address all comments suggested by our data editors listed below:
o Figure legends:
1. Please note that the exact p values are not provided in the legends of figures 1A-D; 3C, D, E, F, I, J, K, L; 4C, D, E, H; 5 C-E; S1 E, S3D, S4 A.
2. Please note that information related to n is missing in the legends of figures S2 B, C
3. Please note that the error bars are not defined in the legends of figures S2B, C.
4. Please note that scale bar and its definition are missing for figures S2A, E.
- Remove "Abbreviations" and incoporate them into the manuscript text.
- Remove BioRender reference from legends and add a dedicated section to the Methods.
Graphics:
(some of the... OR Figure #... OR synopsis) Graphics were created with BioRender.com.
- Please include structured Methods section that includes a Reagents and Tools Table (should be uploaded as a separate file) followed by a Methods and Protocols section. More information on how to adhere to this format as well as downloadable templates (.docx) for the Reagents and Tools Table can be found in our author guidelines:
https://www.embopress.org/page/journal/17574684/authorguide#structuredmethods
An example of a paper with Structured Methods can be found here:
https://www.embopress.org/doi/full/10.1038/s44320-024-00037-6#sec-4
- Remove the table with primer sequences and add them to the reagents and tools table.
- Indicate in legends number and nature of replicates and exact p= values, not a range, along with the statistical test used. To keep the figures "clear" some authors found providing an Appendix table Sx with all exact p-values preferable. You are welcome to do this if you want to.
- Author contributions: Please remove it from the manuscript and specify author contributions in our submission system. CRediT has replaced the traditional author contributions section because it offers a systematic machine-readable author contributions format that allows for more effective research assessment. You are encouraged to use the free text boxes beneath each contributing author's name to add specific details on the author's contribution. More information is available in our guide to authors:
https://www.embopress.org/page/journal/17574684/authorguide#authorshipguidelines
- Correct the reference citation in the text and reference list. In the text, a reference should be cited by author and year of publication. Include a space between a word and the opening parenthesis of the reference that follows. In the reference list, citations should be listed in alphabetical order. Where there are more than 10 authors on a paper, 10 will be listed, followed by "et al.". Please check "Author Guidelines" for more information.
https://www.embopress.org/page/journal/17574684/authorguide#referencesformat
4) Funding: Please merge it with "Acknowledgments" and add Novartis Foundation project number in the manuscript.
5) Tables: Please rename suppl. excel file to Dataset EV1, remove the legend from manuscript text and add to the excel file, in a separate tab/worksheet. Also, update its callout in the text.
6) The Paper Explained: Please provide "The Paper Explained" and add it to the main manuscript text. Please check "Author Guidelines" for more information. https://www.embopress.org/page/journal/17574684/authorguide#researcharticleguide
7) Synopsis: Every published paper now includes a 'Synopsis' to further enhance discoverability. Synopses are displayed on the journal webpage and are freely accessible to all readers. They include separate synopsis image and synopsis text.
- Synopsis image: Please provide a visual abstract as a high-resolution jpeg file 550 px-wide x 300-600 pixels high to illustrate your article.
- Synopsis text: Please provide a short standfirst (maximum of 300 characters, including space) as well as 2-5 one sentence bullet points that summarise the paper as a .doc file. Please write the bullet points to summarise the key NEW findings. They should be designed to be complementary to the abstract - i.e. not repeat the same text. We encourage inclusion of key acronyms and quantitative information (maximum of 30 words / bullet point). Please use the passive voice.

- Please check your synopsis text and image before submission with your revised manuscript. Please be aware that in the proof stage minor corrections only are allowed (e.g., typos).
8) As part of the EMBO Publications transparent editorial process (see our Editorial at http://embomolmed.embopress.org/content/2/9/329), EMBO Molecular Medicine will publish online a Review Process File (RPF) to accompany accepted manuscripts. This file will be published in conjunction with your paper and will include the anonymous referee reports, your point-by-point response and all pertinent correspondence relating to the manuscript. Let us know if you want to remove or not any figures from it prior to publication. Please note that the Authors checklist will be published at the end of the RPF.
9) Please provide a point-by-point letter INCLUDING my comments as well as the reviewer's reports and your detailed responses (as Word file).

I look forward to reading a new revised version of your manuscript as soon as possible.

Yours sincerely,

Zeljko Durdevic

Zeljko Durdevic
Senior Editor
EMBO Molecular Medicine

*** Instructions to submit your revised manuscript ***

*** PLEASE NOTE *** As part of the EMBO Publications transparent editorial process initiative (see our Editorial at https://www.embopress.org/doi/pdf/10.1002/emmm.201000094), EMBO Molecular Medicine will publish online a Review Process File to accompany accepted manuscripts.

In the event of acceptance, this file will be published in conjunction with your paper and will include the anonymous referee reports, your point-by-point response and all pertinent correspondence relating to the manuscript. If you do NOT want this file to be published, please inform the editorial office at contact@embomolmed.org.

*****

When preparing your revised manuscript, please refer to our guidelines: https://link.springer.com/journal/44321/submission-guidelines#cms-Revised-submissions. We perform an initial quality control of all revised manuscripts before re-review; failure to include requested items will delay the evaluation of your revision.

We require:

1) A .docx formatted version of the manuscript text (including legends for main figures, EV figures and tables). Please make sure that the changes are highlighted to be clearly visible.

2) Individual production quality figure files as .eps, .tif, .jpg (one file per figure). For guidance, download the 'Figure Guide PDF': https://media.springernature.com/original/springer-cms/rest/v1/content/27825798/data/v1.

3) A .docx formatted letter INCLUDING the reviewers' reports and your detailed point-by-point responses to their comments. As part of the EMBO Press transparent editorial process, the point-by-point response is part of the Review Process File (RPF), which will be published alongside your paper.

4) A complete author checklist, which you can download from our author guidelines. Please insert information in the checklist that is also reflected in the manuscript. The completed author checklist will also be part of the RPF.

5) Please note that all corresponding authors are required to supply an ORCID ID for their name upon submission of a revised manuscript.

6) It is mandatory to include a 'Data Availability' section after the Materials and Methods. Before submitting your revision, primary datasets produced in this study need to be deposited in an appropriate public database, and the accession numbers and database listed under 'Data Availability'. Please remember to provide a reviewer password if the datasets are not yet public.

In case you have no data that requires deposition in a public database, please state so in this section. Note that the Data Availability Section is restricted to new primary data that are part of this study.

7) For data quantification: please specify the name of the statistical test used to generate error bars and P values, the number (n) of independent experiments (specify technical or biological replicates) underlying each data point and the test used to calculate p-values in each figure legend. The figure legends should contain a basic description of n, P and the test applied. Graphs must include a description of the bars and the error bars (s.d., s.e.m.).

8) At EMBO Press we ask authors to provide source data for the main manuscript figures. You will receive a separate email with instructions for providing source data with your revised manuscript, including how to upload and organize the files.

9) Our journal encourages inclusion of *data citations in the reference list* to directly cite datasets that were re-used and obtained from public databases. Data citations in the article text are distinct from normal bibliographical citations and should directly link to the database records from which the data can be accessed. In the main text, data citations are formatted as follows: "Data ref: Smith et al, 2001" or "Data ref: NCBI Sequence Read Archive PRJNA342805, 2017". In the Reference list, data citations must be labeled with "[DATASET]". A data reference must provide the database name, accession number/identifiers and a resolvable link to the landing page from which the data can be accessed at the end of the reference.

10) We replaced Supplementary Information with Expanded View (EV) Figures and Tables that are collapsible/expandable online. A maximum of 5 EV Figures can be typeset. EV Figures should be cited as 'Figure EV1, Figure EV2" etc... in the text and their respective legends should be included in the main text after the legends of regular figures.

- For the figures that you do NOT wish to display as Expanded View figures, they should be bundled together with their legends in a single PDF file called *Appendix*, which should start with a short Table of Content. Appendix figures should be referred to in the main text as: "Appendix Figure S1, Appendix Figure S2" etc.

- Additional Tables/Datasets should be labeled and referred to as Table EV1, Dataset EV1, etc. Legends have to be provided in a separate tab in case of .xls files. Alternatively, the legend can be supplied as a separate text file (README) and zipped together with the Table/Dataset file.

11) The paper explained: EMBO Molecular Medicine articles are accompanied by a summary of the articles to emphasize the major findings in the paper and their medical implications for the non-specialist reader. Please provide a draft summary of your article highlighting

- the medical issue you are addressing,

- the results obtained and

- their clinical impact.

This may be edited to ensure that readers understand the significance and context of the research. Please refer to any of our published articles for an example.

12) Author contributions: You will be asked to provide CRediT (Contributor Role Taxonomy) terms in the submission system. These replace a narrative author contribution section in the manuscript.

13) A Conflict of Interest statement should be provided in the main text.

14) Every published paper includes a 'Synopsis' to further enhance discoverability. Synopses are displayed on the journal webpage and are freely accessible to all readers. They include a short stand first (maximum of 300 characters, including space) as well as 2-5 one-sentences bullet points that summarizes the paper. Please write the bullet points to summarize the key NEW findings. They should be designed to be complementary to the abstract - i.e. not repeat the same text. We encourage inclusion of key acronyms and quantitative information (maximum of 30 words / bullet point). Please use the passive voice. Please attach these in a separate file or send them by email, we will incorporate them accordingly.

Please also suggest a striking image or visual abstract to illustrate your article as a PNG file 550 px wide x 300 px high.

15) Include a Reagents and Tools Table as part of the Methods section, which can be downloaded from our author guidelines.

EMBO Molecular Medicine has a "scooping protection" policy, whereby similar findings that are published by others during review or revision are not a criterion for rejection. Should you decide to submit a revised version, I do ask that you get in touch after three months if you have not completed it, to update us on the status.

*Additional important information regarding Figures

Each figure should be given in a separate file and should have the following resolution:
Graphs 800-1,200 DPI
Photos 400-800 DPI
Colour (only CMYK) 300-400 DPI"

Figures are not edited by the production team. All lettering should be the same size and style; figure panels should be indicated by capital letters (A, B, C etc). Gridlines are not allowed except for log plots. Figures should be numbered in the order of their appearance in the text with Arabic numerals. Each Figure must have a separate legend and a caption is needed for each panel.

*Additional important information regarding figures and illustrations can be found at
https://media.springernature.com/original/springer-cms/rest/v1/content/27825798/data/v1

***** Reviewer's comments *****

Referee #2 (Comments on Novelty/Model System for Author):

The authors used human cell systems to explore human liver fibrosis

Referee #2 (Remarks for Author):

The authors has satisfactorily addressed my previous comments.

Referee #3 (Remarks for Author):

The revised manuscript has already addressed all the concerns.

The authors addressed the remaining editorial issues.

28th May 2026

Dear Dr. Delangre,

We are pleased to inform you that your manuscript is accepted for publication and is now being sent to our publisher to be included in the next available issue of EMBO Molecular Medicine.

Your manuscript will be processed for publication by EMBO Press. It will be copy edited and you will receive page proofs prior to publication. Please note that you will be contacted by Springer Nature Author Services to complete licensing and payment information.

You may qualify for financial assistance for your publication charges - either via a Springer Nature fully open access agreement or an EMBO initiative. Check your eligibility: https://link.springer.com/journal/44321/how-to-publish-with-us

Should you be planning a Press Release on your article, please notify the production team via this form in order to coordinate publication and release dates: https://forms.cloud.microsoft/e/eH4A8JVP6g

If you have any questions, please do not hesitate to contact the Editorial Office. Thank you for your contribution to EMBO Molecular Medicine.

Yours sincerely,
Zeljko Durdevic

Zeljko Durdevic
Senior Editor
EMBO Molecular Medicine

>>> Please note that it is EMBO Molecular Medicine policy for the transcript of the editorial process (containing referee reports and your response letter) to be published as an online supplement to each paper. If you do NOT want this, you will need to inform the Editorial Office via email immediately. More information is available here: https://link.springer.com/partners/embo-press/editorial-policies#Peer%20review

EMBO Press Author Checklist

Corresponding Author Name: Etienne Delangre and Pierre Maechler	
Journal Submitted to: EMBO Molecular Medicine	
Manuscript Number: 2025-23178-T	

USEFUL LINKS FOR COMPLETING THIS FORM
The EMBO Journal - Author Guidelines
EMBO Reports - Author Guidelines
Molecular Systems Biology - Author Guidelines
EMBO Molecular Medicine - Author Guidelines

Reporting Checklist for Life Science Articles (updated January
This checklist is adapted from Materials Design Analysis Reporting (MDAR) Checklist for Authors. MDAR establishes a minimum set of requirements in transparent reporting in the life sciences (see Statement of Task: 10.31222/osf.io/9sm4x). Please follow the journal's guidelines in preparing your
Please note that a copy of this checklist will be published alongside your article.

Abridged guidelines for figures
1. Data
The data shown in figures should satisfy the following conditions:
- the data were obtained and processed according to the field’s best practice and are presented to reflect the results of the experiments in an accurate and unbiased manner.
- ideally, figure panels should include only measurements that are directly comparable to each other and obtained with the same assay.
- plots include clearly labeled error bars for independent experiments and sample sizes. Unless justified, error bars should not be shown for technical
- if n<5, the individual data points from each experiment should be plotted. Any statistical test employed should be justified.
- Source Data should be included to report the data underlying figures according to the guidelines set out in the authorship guidelines on Data

2. Captions
Each figure caption should contain the following information, for each panel where they are relevant:
- a specification of the experimental system investigated (eg cell line, species name).
- the assay(s) and method(s) used to carry out the reported observations and measurements.
- an explicit mention of the biological and chemical entity(ies) that are being measured.
- an explicit mention of the biological and chemical entity(ies) that are altered/varied/perturbed in a controlled manner.
- the exact sample size (n) for each experimental group/condition, given as a number, not a range;
- a description of the sample collection allowing the reader to understand whether the samples represent technical or biological replicates (including how many animals, litters, cultures, etc.).
- a statement of how many times the experiment shown was independently replicated in the laboratory.
- definitions of statistical methods and measures:
  - common tests, such as t-test (please specify whether paired vs. unpaired), simple χ2 tests, Wilcoxon and Mann-Whitney tests, can be unambiguously identified by name only, but more complex techniques should be described in the methods section;
  - are tests one-sided or two-sided?
  - are there adjustments for multiple comparisons?
  - exact statistical test results, e.g., P values = x but not P values < x;
  - definition of ‘center values’ as median or average;
  - definition of error bars as s.d. or s.e.m.

Please complete ALL of the questions below.
Select "Not Applicable" only when the requested information is not relevant for your study.

Materials

Newly Created Materials	Information included in the manuscript?	In which section is the information available? (Reagents and Tools Table, Materials and Methods, Figures, Data Availability Section)
New materials and reagents need to be available; do any restrictions apply?	Not Applicable	N/A

Antibodies	Information included in the manuscript?	In which section is the information available? (Reagents and Tools Table, Materials and Methods, Figures, Data Availability Section)
For **antibodies** provide the following information:<br>- Commercial antibodies: RRID (if possible) or supplier name, catalogue number and or/clone number<br>- Non-commercial: RRID or citation	Yes	Reagents and Tools table

DNA and RNA sequences	Information included in the manuscript?	In which section is the information available? (Reagents and Tools Table, Materials and Methods, Figures, Data Availability Section)
**Short novel DNA or RNA including primers, probes**: provide the sequences.	Yes	Appendix Table S1

Cell materials	Information included in the manuscript?	In which section is the information available? (Reagents and Tools Table, Materials and Methods, Figures, Data Availability Section)
**Cell lines:** Provide species information, strain. Provide accession number in repository **OR** supplier name, catalog number, clone number, and/**OR** RRID.	Yes	Reagents and Tools table
**Primary cultures:** Provide species, strain, sex of origin, genetic modification status.	Not Applicable	
Report if the cell lines were recently **authenticated** (e.g., by STR profiling) and tested for mycoplasma contamination.	Yes	Material and Methods

Experimental animals	Information included in the manuscript?	In which section is the information available? (Reagents and Tools Table, Materials and Methods, Figures, Data Availability Section)
**Laboratory animals or Model organisms:** Provide species, strain, sex, age, genetic modification status. Provide accession number in repository **OR** supplier name, catalog number, clone number, **OR** RRID.	Not Applicable	N/A
**Animal observed in or captured from the field:** Provide species, sex, and age where possible.	Not Applicable	N/A
Please detail **housing and husbandry conditions**.	Not Applicable	N/A

Plants and microbes	Information included in the manuscript?	In which section is the information available? (Reagents and Tools Table, Materials and Methods, Figures, Data Availability Section)
**Plants:** provide species and strain, ecotype and cultivar where relevant, unique accession number if available, and source (including location for collected wild specimens).	Not Applicable	N/A
**Microbes:** provide species and strain, unique accession number if available, and source.	Not Applicable	N/A

Human research participants	Information included in the manuscript?	In which section is the information available? (Reagents and Tools Table, Materials and Methods, Figures, Data Availability Section)
If collected and within the bounds of privacy constraints report on age, sex and gender or ethnicity for all study participants.	Not Applicable	N/A

Core facilities	Information included in the manuscript?	In which section is the information available? (Reagents and Tools Table, Materials and Methods, Figures, Data Availability Section)
If your work benefited from core facilities, was their service mentioned in the acknowledgments section?	Yes	Acknowledgments

Design

Study protocol	Information included in the manuscript?	In which section is the information available? (Reagents and Tools Table, Materials and Methods, Figures, Data Availability Section)
If study protocol has been **pre-registered, provide DOI in the manuscript**. For clinical trials, provide the trial registration number **OR** cite DOI.	Not Applicable	N/A
Report the **clinical trial registration number** (at ClinicalTrials.gov or equivalent), where applicable.	Not Applicable	N/A

Laboratory protocol	Information included in the manuscript?	In which section is the information available? (Reagents and Tools Table, Materials and Methods, Figures, Data Availability Section)
Provide DOI OR other citation details if **external detailed step-by-step protocols** are available.	Not Applicable	N/A

Experimental study design and statistics	Information included in the manuscript?	In which section is the information available? (Reagents and Tools Table, Materials and Methods, Figures, Data Availability Section)
Include a statement about **sample size** estimate even if no statistical methods were used.	Yes	Material and Methods - Figure legends
Were any steps taken to minimize the effects of subjective bias when allocating animals/samples to treatment (e.g. **randomization procedure**)? If yes, have they been described?	Not Applicable	N/A
Include a statement about **blinding** even if no blinding was done.	Yes	Material and Methods
Describe **inclusion/exclusion criteria** if samples or animals were excluded from the analysis. Were the criteria pre-established?<br><br>If sample or data points were omitted from analysis, report if this was due to attrition or intentional exclusion and provide justification.	Yes	Material and Methods
For every figure, are **statistical tests** justified as appropriate? Do the data meet the assumptions of the tests (e.g., normal distribution)? Describe any methods used to assess it. Is there an estimate of variation within each group of data? Is the variance similar between the groups that are being statistically compared?	Yes	Material and Methods - Figure Legends - Appendix Table S2

Sample definition and in-laboratory replication	Information included in the manuscript?	In which section is the information available? (Reagents and Tools Table, Materials and Methods, Figures, Data Availability Section)
In the figure legends: state number of times the experiment was **replicated** in laboratory.	Yes	Material and Methods
In the figure legends: define whether data describe **technical or biological replicates**.	Yes	Material and Methods

Ethics

Ethics	Information included in the manuscript?	In which section is the information available? (Reagents and Tools Table, Materials and Methods, Figures, Data Availability Section)
Studies involving **human participants**: State details of **authority granting ethics approval** (IRB or equivalent committee(s), provide reference number for approval.	Yes	Material and Methods and Ethic statement
Studies involving **human participants**: Include a statement confirming that **informed consent** was obtained from all subjects and that the experiments conformed to the principles set out in the WMA Declaration of Helsinki and the Department of Health and Human Services Belmont Report.	Not Applicable	
Studies involving **human participants: For publication of patient photos**, include a statement confirming that consent to publish was obtained.	Not Applicable	
Studies involving experimental **animals**: State details of **authority granting ethics approval** (IRB or equivalent committee(s), provide reference number for approval. Include a statement of compliance with ethical regulations.	Not Applicable	
Studies involving **specimen and field samples:** State if relevant **permits** obtained, provide details of authority approving study; if none were required, explain why.	Not Applicable	

Dual Use Research of Concern (DURC)	Information included in the manuscript?	In which section is the information available? (Reagents and Tools Table, Materials and Methods, Figures, Data Availability Section)
Could your study fall under dual use research restrictions? Please check biosecurity documents and list of **select agents and toxins** (CDC): https://www.selectagents.gov/sat/list.htm	Not Applicable	
If you used a select agent, is the security level of the lab appropriate and reported in the manuscript?	Not Applicable	
If a study is subject to dual use research of concern regulations, is the name of the **authority granting approval and reference number** for the regulatory approval provided in the manuscript?	Not Applicable	

Reporting

The MDAR framework recommends adoption of discipline-specific guidelines, established and endorsed through community initiatives. Journals have their own policy about requiring specific guidelines and recommendations to complement MDAR.

Adherence to community standards	Information included in the manuscript?	In which section is the information available? (Reagents and Tools Table, Materials and Methods, Figures, Data Availability Section)
State if relevant guidelines or checklists (e.g., **ICMJE, MIBBI, ARRIVE, PRISMA**) have been followed or provided.	Not Applicable	
For **tumor marker prognostic studies,** we recommend that you follow the **REMARK** reporting guidelines (see link list at top right). See author guidelines, under 'Reporting Guidelines'. Please confirm you have followed these guidelines.	Not Applicable	
For **phase II and III randomized controlled trials**, please refer to the **CONSORT** flow diagram (see link list at top right) and submit the CONSORT checklist (see link list at top right) with your submission. See author guidelines, under 'Reporting Guidelines'. Please confirm you have submitted this list.	Not Applicable	

Data Availability

Data availability	Information included in the manuscript?	In which section is the information available? (Reagents and Tools Table, Materials and Methods, Figures, Data Availability Section)
Have **primary datasets** been deposited according to the journal's guidelines (see 'Data Deposition' section) and the respective accession numbers provided in the Data Availability Section?	Yes	Data Availability Section
Were **human clinical and genomic datasets** deposited in a public access-controlled repository in accordance to ethical obligations to the patients and to the applicable consent agreement?	Not Applicable	
Are **computational models** that are central and integral to a study available without restrictions in a machine-readable form? Were the relevant accession numbers or links provided?	Not Applicable	
If publicly available data were reused, provide the respective **data citations in the reference list**.	Yes	Material and Methods - Result section - Reference section
